# Quantum dot sensors for heavy metal detection: from mechanistic design to field deployment: a review

**DOI:** 10.1039/d6ra06857j

**Published:** 2026-10-12

**Authors:** Garima Rathee, Sahil Kohli, Rida Hashami, Krati Singh, Jyotsna Rathee, Barkha Rathee, Suphiya Khan

**Affiliations:** a Department of Chemical Engineering, Universitat Politècnica de Catalunya Barcelona Spain garimaratheegr@gmail.com garima.rathee@upc.edu; b Dr Bansi Dhar Institute Gurugram Haryana India garimarathee@drbansidharinstitute.org; c Department of Chemistry, School of Engineering and Technology, Manav Rachna International Institute of Research and Studies Faridabad Haryana-121004 India; d Biosensor Lab, Department of Chemistry, BITS Pilani KK Birla Goa Campus Vasco da Gama 403726 Goa India; e Department of Bioscience and Biotechnology, Banasthali Vidyapith Rajasthan 304022 India; f Computer Science & Engineering Department, Deenbandhu Chhotu Ram University of Science and Technology Sonipat 131039 India; g Department of Chemistry, MDU Rohtak-124001 Haryana India

## Abstract

Heavy metal contamination poses a critical global health risk, affecting over 2.2 billion people and necessitating rapid, sensitive, and field-deployable detection technologies. Conventional analytical techniques such as ICP-MS and AAS, while highly accurate, remain constrained by high cost, complex instrumentation, and limited portability, restricting their use in decentralized monitoring. Quantum dots (QDs), owing to their size-tunable photoluminescence, high quantum yields, and excellent photostability, have emerged as powerful alternatives for ultrasensitive detection of toxic metal ions. This review provides a comprehensive and structured overview of QD-based sensing systems, beginning with fundamental aspects including synthesis strategies (colloidal, hydrothermal, and green routes), quantum confinement effects, and exciton dynamics governing optical behaviour. Emphasis is placed on surface functionalization approaches guided by Hard–Soft Acid–Base (HSAB) theory and chelation principles, enabling high selectivity toward target ions. Key sensing mechanisms, including fluorescence quenching and enhancement, Förster resonance energy transfer (FRET), and electrochemical transduction, are critically analyzed. Recent advances in hybrid nanostructures integrating QDs with graphene, metal–organic frameworks, and plasmonic materials are discussed, highlighting significant improvements in sensitivity down to picomolar levels and detection limits as low as 0.1 nM for priority contaminants such as Pb^2+^, Hg^2+^, Cd^2+^, As^3+/5+^, and Cr^6+^. In parallel, emerging eco-friendly alternatives, including carbon-based and heteroatom-doped QDs, are evaluated as sustainable substitutes to toxic Cd- and Pb-based systems, demonstrating competitive optical performance and improved biocompatibility. The review further examines practical considerations often overlooked, including matrix interference, reproducibility, long-term stability, and scalability. Strategies for translation from laboratory prototypes to real-world deployment are discussed, with focus on regulatory validation (IUPAC protocols, NIST standards), scalable manufacturing approaches, and cost-effective sensor design. Finally, future directions are outlined, emphasizing integration with artificial intelligence, IoT-enabled monitoring systems, and multiplexed sensing platforms aligned with ASSURED criteria. By bridging fundamental materials design with application-driven challenges, this work provides a roadmap for advancing QD-based sensors toward reliable, scalable, and commercially viable solutions for global heavy metal monitoring.

## Abstract 2

Quantum dot sensors offer sensitive, selective and low-cost detection of Pb, Hg, Cd, As and Cr ions, using optical and electrochemical signal transduction for water treatment, food safety, biomedical and point-of-care applications.

## Graphical abstract

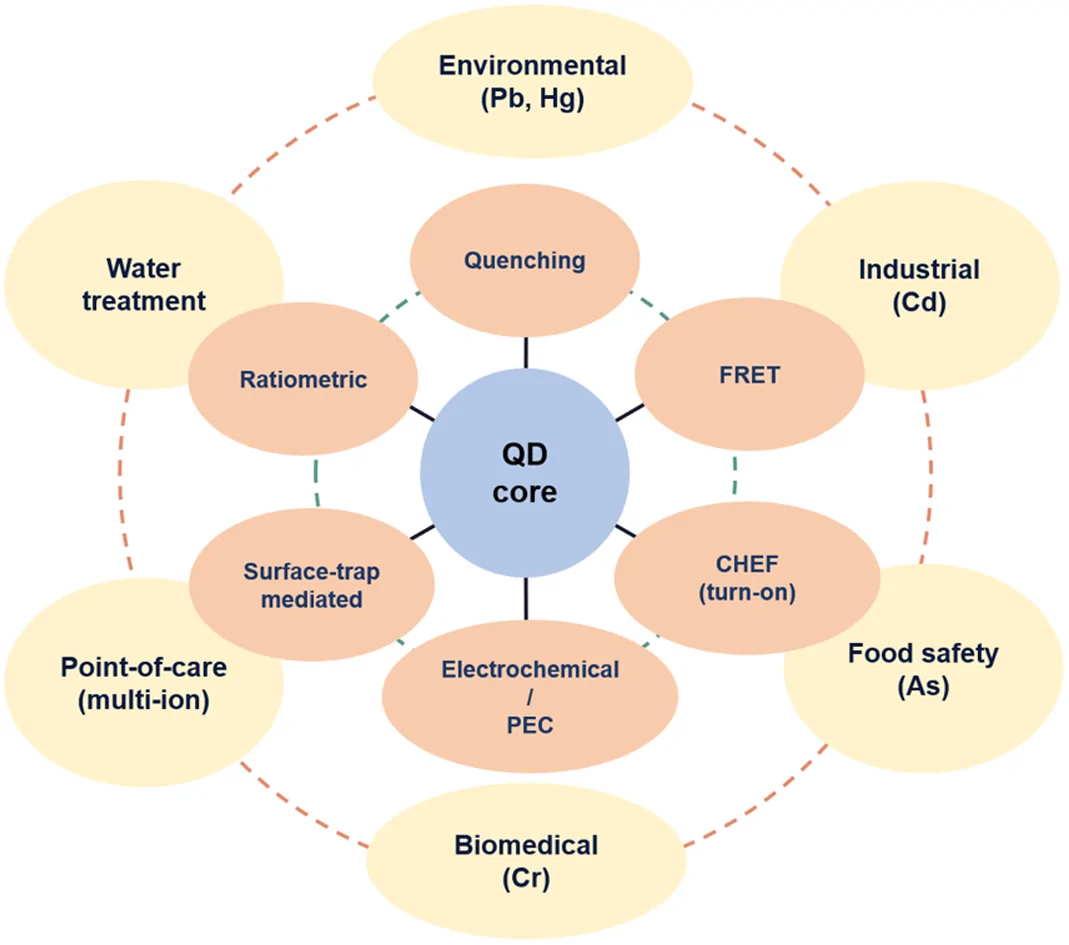

## 1. Introduction

Heavy metal contamination represents one of the most pressing environmental challenges of the 21st century, affecting an estimated 200 million people globally through contaminated drinking water sources.^1,2^ Unlike organic pollutants, heavy metals such as lead (Pb^2+^), mercury (Hg^2+^), cadmium (Cd^2+^), arsenic (As^3+^/As^5+^), and hexavalent chromium (Cr^6+^) are non-biodegradable, bioaccumulative, and exhibit severe toxicity even at trace concentrations.^3–5^ These contaminants enter aquatic systems through multiple anthropogenic pathways including industrial effluents, mining operations, agricultural runoff, and leaching from water distribution infrastructure.^6^ Upon release, heavy metals accumulate in sediments, soil, and water bodies, subsequently entering the food chain through bioaccumulation in aquatic organisms and crops.^7^ Within biological systems, these metals bind to critical protein sites, displacing essential elements and disrupting vital enzymatic and metabolic processes.^8^ Chronic exposure, even at sub-threshold levels, has been linked to neurodevelopmental impairments in children, carcinogenesis, nephrotoxicity, hepatotoxicity, and cardiovascular dysfunction.^9,10^ The World Health Organisation (WHO) has established stringent permissible limits for heavy metals in drinking water, for instance, 10 µg L^−1^ for arsenic and lead, underscoring the urgent need for reliable, sensitive detection methods to safeguard public and environmental health.^11^

Current analytical techniques for heavy metal quantification, including inductively coupled plasma mass spectrometry (ICP-MS), atomic absorption spectroscopy (AAS), X-ray fluorescence spectrophotometry, and ion chromatography, offer excellent sensitivity and accuracy, with detection limits (LODs) often reaching parts-per-billion or parts-per-trillion levels.^12^ However, these conventional methods are constrained by several critical limitations that restrict their deployment beyond centralised laboratory settings.^13^ They require expensive, bulky instrumentation, necessitate extensive sample preparation protocols involving acid digestion or preconcentration steps, demand highly trained personnel for operation, and typically involve lengthy analysis times ranging from hours to days.^13,14^ Moreover, these techniques are often limited to single-element analysis per run, lack portability for field applications, and generate hazardous chemical waste.^15–17^ These constraints render conventional methods unsuitable for rapid, on-site, or point-of-care testing, particularly in resource-limited settings where heavy metal contamination poses the greatest risk.^18^ The WHO has advocated for the development of detection technologies adhering to the ASSURED criteria: Affordable, Sensitive, Specific, User-friendly, Rapid and Robust, Equipment-free, and Deliverable to end-users.^19^ This framework has driven research towards miniaturised, cost-effective sensing platforms capable of real-time, *in situ* heavy metal monitoring.^20,21^

Quantum dots (QDs) have emerged as a transformative class of nanomaterials uniquely positioned to address the limitations of conventional detection methods whilst meeting ASSURED criteria.^22–24^ QDs are semiconductor nanocrystals typically ranging from 2 to 10 nm in diameter, exhibiting quantum confinement effects that endow them with exceptional optical and electronic properties.^25,26^ Their size-dependent, tunable photoluminescence, spanning the ultraviolet to near-infrared spectrum, enables precise spectral engineering for multiplexed detection applications. QDs demonstrate remarkably high quantum yields (QYs), frequently exceeding 50–80%, alongside superior photostability compared to traditional organic fluorophores, which are prone to rapid photobleaching. Their broad absorption spectra coupled with narrow, symmetric emission peaks minimise spectral overlap and enhance signal-to-noise ratios, critical parameters for achieving high specificity in complex sample matrices. Furthermore, the surface chemistry of QDs is highly amenable to functionalisation with diverse recognition elements including thiol compounds, carboxylic acids, peptides, aptamers, and antibodies, enabling selective coordination with target metal ions.^26–29^ This versatility allows for rational sensor design tailored to specific heavy metals through chelation, electrostatic interactions, or biomolecular recognition mechanisms.

The utility of QDs in heavy metal sensing is underpinned by multiple transduction mechanisms, including fluorescence quenching and enhancement, Förster resonance energy transfer (FRET), and electrochemical modalities.^30,31^ Upon interaction with target metal ions, QD fluorescence can be modulated through various pathways: electron or energy transfer processes, formation of non-fluorescent complexes, surface state alterations, or aggregation-induced changes.^32^ These mechanisms enable ultrasensitive detection, with reported limits of detection routinely achieving nanomolar to picomolar concentrations, orders of magnitude lower than regulatory thresholds.^33,34^ Recent advances have seen the integration of QDs with complementary nanomaterials such as graphene, carbon nanotubes (CNTs), metal–organic frameworks (MOFs), and plasmonic nanostructures, yielding hybrid architectures that further amplify sensitivity through synergistic signal enhancement.^35–37^ Additionally, the development of eco-friendly alternatives, including CDs, graphene QDs (GQDs), and silicon QDs, has addressed toxicity concerns associated with traditional cadmium- and lead-based QDs, facilitating translation towards practical applications.^38^ The past decade has witnessed remarkable progress in QD synthesis methodologies, including hydrothermal, microwave-assisted, and green synthetic routes utilising plant extracts or microorganisms, which offer improved reproducibility, scalability, and environmental sustainability.^39,40^

Despite these advances, significant challenges remain in translating QD-based sensors from laboratory demonstrations to widespread field deployment.^41,42^ Issues such as batch-to-batch synthesis variability, interference from coexisting ions in complex environmental matrices, long-term stability under ambient conditions, and regulatory approval for commercial use continue to impede practical implementation.^43,44^ Nevertheless, the integration of QDs into portable, smartphone-compatible detection platforms, lateral flow devices, and paper-based sensors represents a promising trajectory towards decentralised heavy metal monitoring.^19^ The convergence of advances in nanomaterial synthesis, surface engineering, signal transduction mechanisms, and device miniaturisation positions QD-based sensors as viable candidates for next-generation environmental and clinical diagnostics.^45,46^

This review provides a comprehensive and critical analysis of QD technologies for heavy metal detection. We systematically examine QD fundamentals, including structure, synthesis methodologies (colloidal, hydrothermal, microwave-assisted, and green approaches), and photophysical properties.^47–49^ The mechanisms underlying QD-based heavy metal sensing, fluorescence quenching, enhancement, FRET, and electrochemical transduction, are rigorously evaluated alongside discussions of selectivity, sensitivity, and kinetic performance.^50^ Detailed analyses of QD sensors targeting specific heavy metals (Pb^2+^, Hg^2+^, Cd^2+^, As^3+/5+^, Cr^6+^) are presented, highlighting state-of-the-art LODs, hybrid nanomaterial architectures, and surface functionalisation strategies.^51–53^ Performance metrics including sensitivity, selectivity, stability, response time, and reproducibility are critically assessed across diverse application domains: environmental monitoring, industrial effluent analysis, food safety testing, and biomedical diagnostics.^54^ We address persistent challenges such as heavy metal-based QD toxicity, synthesis standardisation, matrix effects, and environmental stability, whilst exploring emerging solutions through carbon-based and other low-toxicity QD alternatives. Finally, we discuss future directions towards ASSURED-compliant, multiplexed, portable sensing platforms and emphasise the imperative for robust regulatory frameworks and sustainable innovation to combat escalating anthropogenic heavy metal pollution. This review is intended to serve as a valuable resource for researchers, industrial practitioners, and policymakers engaged in advancing nanomaterial-based environmental and public health diagnostics. Fig. 1 presents a schematic overview of this sensing landscape, illustrating the principal transduction mechanisms operating at the QD core alongside the major application domains; environmental monitoring, industrial process control, food safety, biomedical diagnostics, point-of-care testing, and water treatment; in which QD-based heavy metal sensors have found utility.

**Fig. 1:**
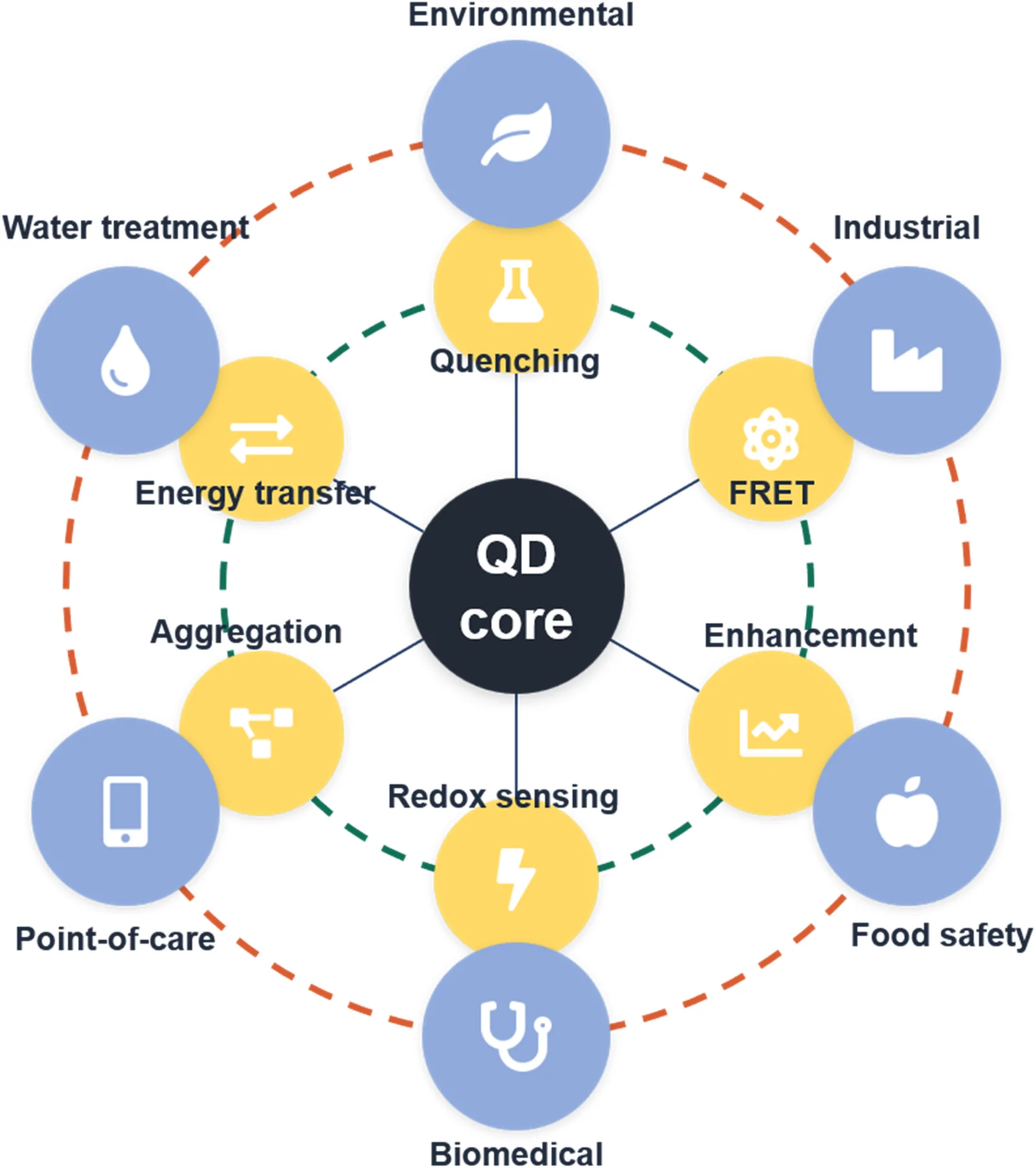
Schematic overview of QD-based sensing for heavy metal detection.

Despite this substantial and growing body of work, three limitations recur across the QD heavy-metal sensing literature that motivate the present review. First, cross-study comparison of sensing performance is complicated by inconsistent experimental reporting: buffer identity, LOD determination methodology, and validation against reference analytical techniques such as ICP-MS are frequently omitted from the primary literature, and this review reports each parameter exactly as documented in its source study, explicitly noting where a parameter is unreported rather than estimating it, so that readers can assess comparability rather than assume it. Second, the connection between fundamental QD photophysical properties, including particle size, surface trap-state density, and defect density, and the resulting sensing performance is often left implicit in individual sensor reports; this review makes that connection explicit, linking these properties directly to achievable LOD, sensitivity, and selectivity. Third, the substantial majority of QD-based sensors reported to date have progressed only as far as proof-of-concept demonstration or real-sample validation under laboratory conditions, with genuinely field-tested systems remaining comparatively rare; this review introduces an explicit distinction between these three validation tiers and applies it where the underlying literature permits.

### 1.1. Positioning of this review

While several reviews have addressed QD-based heavy metal sensing in recent years, their scope differs from the present work. Wang *et al.*^55^ organize their review around QD types and transducer principles (optical *versus* electrochemical sensing) and give substantial attention to portable and smartphone-integrated devices, but do not provide a comparative treatment of toxicity and sustainability across QD material classes, nor a dedicated discussion of commercialization and regulatory barriers. Shrivastav and Khansili^56^ center their review on multiplexed detection strategies for simultaneous quantification of several metal ions, organizing part of their discussion by individual metal (Cr, As, Hg, Pb, Cd, and others); toxicity and commercialization likewise receive only brief mention rather than dedicated comparative treatment. The present review differs in providing an integrated treatment spanning QD photophysics, systematic linkage of surface engineering to sensing mechanism, metal-specific critical comparison (Pb, Hg, Cd, As, Cr), a comparative toxicity and sustainability assessment across QD material classes (cadmium/lead-based, carbon-based, graphene, perovskite), and a dedicated discussion of scalability, cost, and regulatory barriers to field deployment and commercialization. Table 1 summarizes this positioning relative to the two most closely related recent reviews.

**Table 1:** Positioning of this review relative to recent QD heavy-metal sensing reviews

Criterion	Wang *et al.*, *Talanta* (2022)^55^	Shrivastav & Khansili, *Trends Environ. Anal. Chem.* (2024)^56^	This review
Primary focus	• Organized by QD type and transducer principle	• Organized around multiplexed detectiond	• Integrated mechanistic-to-field-deployment treatment
	• Optical *vs.* electrochemical sensors, plus portable/*in situ* devices	• Simultaneous quantification of multiple metal ions; partly organized per metal (Cr, As, Hg, Pb, Cd, *etc.*)	• Spans photophysics, synthesis, sensing mechanisms, metal-specific performance, and translation
Sensing mechanism coverage	• FL (direct and ratiometric) and electrochemical (PEC, ECL) probes	• Fluorescent, electrochemical, colorimetric, and photoluminescence probes	• Fluorescence quenching/enhancement, FRET/ratiometric, electrochemical/PEC
	• Organized by QD-nanomaterial and QD-biomaterial modification strategies	• Framed around multiplexing capability of each mechanism	• Explicitly linked to surface ligand chemistry and HSAB-guided design
Analytical performance benchmarking	• Reports LODs per cited study, organized by sensor type, not by metal	• Table 3 summarizes LOD by metal ion across multiple QD sensors	• Dedicated per-metal comparative tables (Pb, Hg, Cd, As, Cr)
	• No standardized cross-study comparison table	• Strong per-metal LOD compilation, less discussion of matrix/pH conditions	• Notes matrix, pH, and interference conditions alongside LOD/sensitivity
Real-sample validation	• Individual studies cite tap water/lake water samples	• Tables list detection matrices (blood, DNA, tap/lake water) per study	• Addressed per metal-ion section
	• Not synthesized into a dedicated cross-study comparison	• Compiled but not critically cross-compared for recovery/matrix effects	• Cross-compared with recovery and matrix effects across studies
Field deployment	• Dedicated section on portable devices (optical, electrical, smartphone-based)	• Portable/on-site detection mentioned mainly in future outlook	• Dedicated section distinguishing proof-of-concept, real-sample validation, and field testing
	• Strong coverage of this specific theme	• Not a dedicated section	• Portable instrumentation and ICP-MS/AAS comparison included
Toxicity/sustainability	• Raised only as a closing future-challenge item	• Non-toxic QD alternatives mentioned in passing	• Comparative toxicity discussion across Cd-, Pb-, carbon-, graphene-, and perovskite-based QDs
	• No comparative treatment across QD material classes	• No dedicated comparative toxicity discussion	• Includes disposal and environmental stability considerations
Commercialization readiness	• Not discussed beyond general future outlook	• Not discussed in depth	• Dedicated discussion of scalability, cost, regulatory approval, and manufacturing barriers

## 2. Toxicity of heavy metals

### 2.1. Introduction to heavy metal toxicity

Heavy metals represent a critical class of environmental pollutants that pose significant threats to ecosystem integrity and human health globally.^57,58^ These metallic elements, traditionally defined by atomic densities exceeding 5 g cm^−3^, include both essential trace elements that become toxic at elevated concentrations (copper, iron, zinc, manganese, cobalt) and non-essential metals with no known biological function that exhibit toxicity even at trace levels (lead, mercury, cadmium, arsenic, chromium).^59^ The industrial revolution and subsequent intensification of anthropogenic activities, including mining operations, metallurgical processing, fossil fuel combustion, agricultural practices involving pesticides and phosphate fertilizers, electronic waste recycling, and industrial effluent discharge, have dramatically increased environmental heavy-metal burdens.^2,3^ Consequently, human exposure has intensified through multiple pathways, including contaminated drinking water, foodstuffs, airborne particulate matter, and occupational environments.^60^Fig. 2 illustrates the cascade of molecular events initiated by heavy metal exposure, linking oxidative stress and inflammatory signalling to DNA damage and multi-organ toxicity.^61^

**Fig. 2:**
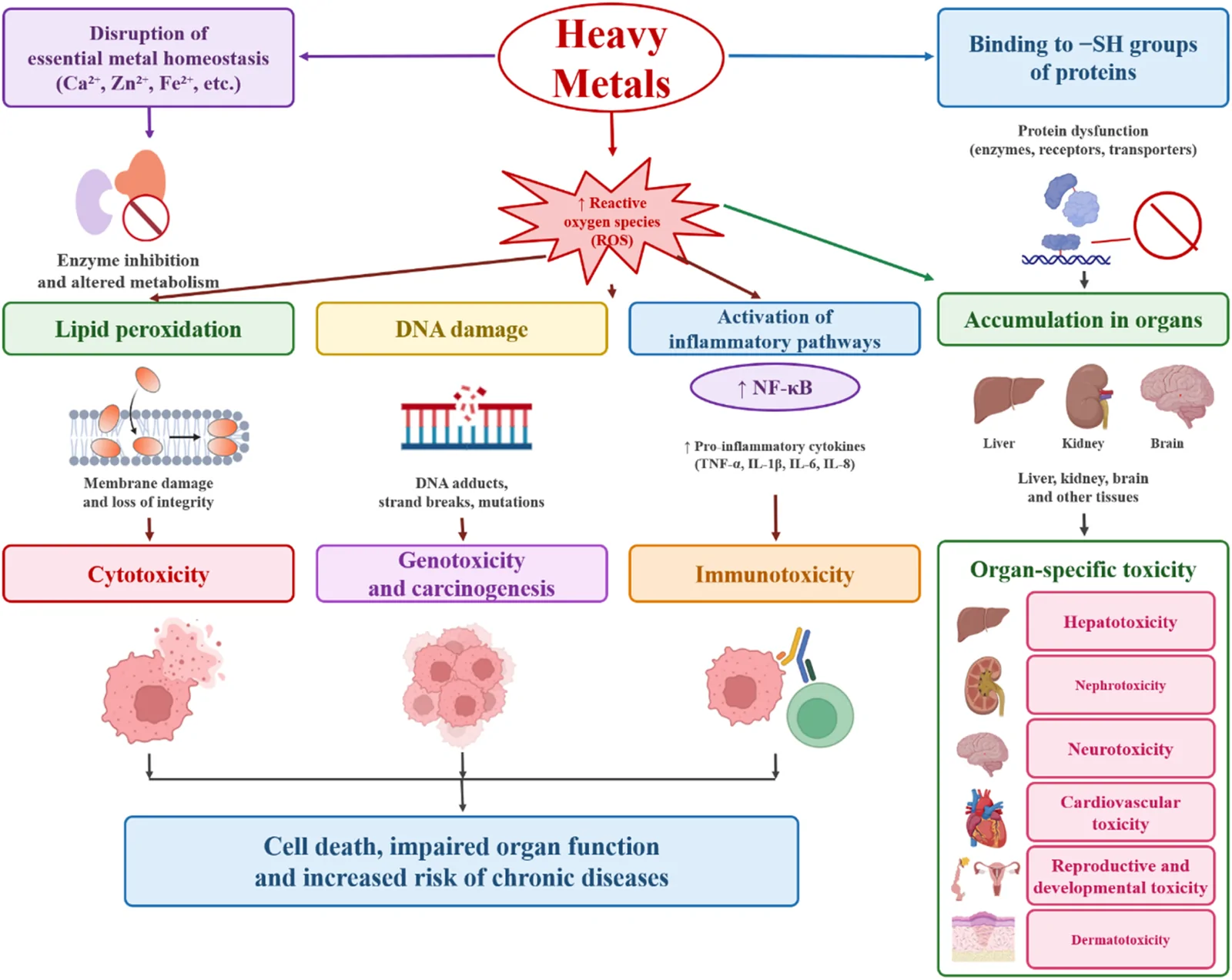
Schematic overview of the molecular mechanisms and systemic toxicological effects induced by heavy metal exposure.

The toxicological profile of heavy metals is governed by multiple interrelated determinants. Chemical speciation and oxidation state play a critical role, with trivalent arsenic (As^3+^) displaying substantially higher cytotoxicity than pentavalent arsenate (As^5+^), and hexavalent chromium (Cr^6+^) exhibiting carcinogenicity absent in trivalent chromium (Cr^3+^).^62,63^ The route and duration of exposure further modulate toxic outcomes, as inhalation enables rapid systemic uptake *via* alveolar diffusion, whereas oral exposure is modulated by gastrointestinal absorption and hepatic first-pass metabolism.^60^ Dose–response relationships are frequently non-linear and threshold-free for endocrine-disrupting and neurotoxic effects.^64^ Bioaccumulation potential and biological half-life represent additional key determinants, with cadmium accumulating in renal cortex for decades (biological half-life 10–30 years) and methylmercury persisting in nervous tissue with elimination half-lives extending to years.^65–67^ Finally, individual susceptibility shapes the ultimate toxic response and is determined by age, genetic polymorphisms in detoxification pathways, nutritional status, and pre-existing disease.^67–69^

According to the WHO and the United States Environmental Protection Agency (US EPA), lead (Pb), mercury (Hg), cadmium (Cd), arsenic (As), and chromium (Cr) rank among the most hazardous environmental contaminants requiring strict regulatory oversight.^68,69^ The International Agency for Research on Cancer (IARC) classifies arsenic, cadmium, and hexavalent chromium as Group 1 carcinogens, while inorganic lead compounds are classified as Group 2A (probably carcinogenic to humans).^70,71^ Drinking-water guideline values have been progressively lowered (As and Pb: 10 µg L^−1^; Cd: 5 µg L^−1^; Hg: 2 µg L^−1^), reflecting growing recognition that adverse effects occur at exposure levels previously considered safe.^69^

### 2.2. Molecular mechanisms of heavy metal toxicity

#### 2.2.1. Oxidative stress: the central paradigm

Oxidative stress constitutes the dominant and unifying mechanistic pathway underlying heavy-metal toxicity across tissues and species.^62,72,73^ Redox-active metals, including iron (Fe^2+^/Fe^3+^), copper (Cu^+^/Cu^2+^), and chromium (Cr^3+^/Cr^6+^), participate directly in redox cycling, catalysing Fenton and Haber–Weiss reactions that convert hydrogen peroxide into highly reactive hydroxyl radicals:^72,74^Fe^2+^ + H_2_O_2_ → Fe^3+^ + ˙OH + OH^−^O_2_˙^−^ + H_2_O_2_ → ˙OH + OH^−^ + O_2_

Hydroxyl radicals exhibit near-diffusion-limited reactivity, indiscriminately damaging nearby macromolecules within nanometre distances.^74^ Lipid peroxidation propagates chain reactions within biological membranes, generating toxic aldehydes such as malondialdehyde and 4-hydroxynonenal, while protein oxidation alters enzyme conformation and activity. Oxidative DNA damage includes base lesions (8-oxo-2′-deoxyguanosine), abasic sites, and strand breaks that promote mutagenesis and carcinogenesis when repair capacity is exceeded.^75^

Redox-inactive metals such as Pb^2+^, cadmium Cd^2+^, and mercury Hg^2+^ induce oxidative stress indirectly through several interconnected mechanisms. These metals exhibit high-affinity binding to glutathione (GSH), depleting the primary intracellular redox buffer and compromising cellular antioxidant capacity. Additionally, they inhibit critical antioxidant enzymes including superoxide dismutase (SOD), catalase (CAT), GSH peroxidase (GPx), and GSH reductase (GR) by binding to cysteine residues or displacing essential cofactors. Furthermore, redox-inactive metals mobilize endogenous iron and copper from metalloproteins, thereby amplifying Fenton chemistry and exacerbating oxidative damage. Mitochondrial electron-transport chain disruption represents another key pathway, as these metals increase superoxide leakage from complexes I and III, further contributing to cellular oxidative stress.^76^

#### 2.2.2. Protein binding and enzyme inhibition

Heavy metals exhibit pronounced affinity for nucleophilic functional groups within proteins, including sulfhydryl (–SH), imidazole, and carboxylate moieties, leading to enzyme inhibition and protein misfolding.^72,77^ Cadmium displaces zinc from zinc-finger transcription factors, disrupting DNA binding and gene regulation, while mercury forms stable mercaptide complexes with cysteine residues.^78^ Lead toxicity exemplifies targeted enzyme inhibition with systemic consequences. By displacing zinc from δ-aminolevulinic acid dehydratase (δ-ALAD) and ferrochelatase, lead disrupts heme biosynthesis, causing anaemia and accumulation of δ-aminolevulinic acid, a neurotoxic metabolite with gamma-aminobutyric acid (GABA)-mimetic properties.^78^ Erythrocyte δ-ALAD inhibition remains a sensitive biomarker of lead exposure. Methylmercury forms cysteine complexes that mimic methionine, enabling transport across the blood–brain barrier *via* neutral amino acid transporters.^79^ Within neurons, methylmercury disrupts microtubule assembly, impairs synaptic vesicle trafficking, inhibits mitochondrial respiration, and induces oxidative stress, culminating in selective neuronal degeneration.^80^

#### 2.2.3. Mitochondrial dysfunction and apoptosis

Mitochondria are central intracellular targets of heavy-metal toxicity due to their role in ATP synthesis, calcium homeostasis, and apoptotic regulation.^81^ Metals inhibit respiratory chain complexes, dissipate mitochondrial membrane potential, and induce permeability transition pore opening. Oxidative damage to mitochondrial DNA, lacking histone protection and robust repair mechanisms, further compromises cellular bioenergetics.^82^ Mitochondrial dysfunction activates apoptosis through release of cytochrome *c*, apoptosis-inducing factor (AIF), endonuclease G, and Smac/DIABLO, triggering both caspase-dependent and caspase-independent cell death pathways.^81^ Heavy metals frequently shift the balance of Bcl-2 family proteins toward pro-apoptotic signaling, promoting irreversible cell loss in sensitive tissues.^83^

#### 2.2.4. Genotoxicity and carcinogenesis

Arsenic, cadmium, and chromium exert carcinogenic effects predominantly through indirect genotoxic mechanisms rather than direct DNA interaction. These metals compromise genomic integrity *via* oxidative DNA damage, suppression of base-excision repair (BER) and nucleotide-excision repair (NER) pathways, epigenetic reprogramming, and induction of chromosomal instability.^84^ Arsenic disrupts normal DNA methylation patterns by inhibiting DNA methyltransferases, producing paradoxical genome-wide hypomethylation concurrent with promoter-specific hypermethylation of tumor-suppressor genes, thereby silencing critical growth-regulatory pathways.^85^ Hexavalent chromium [Cr(vi)] enters cells *via* sulfate anion transporters and undergoes sequential intracellular reduction to lower oxidation states, generating highly reactive Cr(v) and Cr(iv) intermediates alongside the stable Cr(iii) product. This reductive metabolism produces substantial quantities of reactive oxygen species while forming ternary Cr(iii)-DNA-protein crosslinks that impede replication fork progression and trigger error-prone translesion DNA synthesis.^86^ The carcinogenic potential of occupational Cr(vi) exposure is firmly established through extensive epidemiological evidence demonstrating dose-dependent increases in lung cancer incidence among chromate production workers.^87^

## 3. Design requirements for heavy metal detection and the emergence of QDs as an optimal sensing platform

The persistent contamination of environmental and drinking water systems by heavy metals has necessitated the development of analytical technologies capable of sensitive, selective, and decentralised detection. Metal ions such as Pb^2+^, Hg^2+^, Cd^2+^, As^3+^/As^5+^, and Cr^6+^ are non-biodegradable and bioaccumulative, exerting toxicological effects at concentrations often well below parts-per-billion levels.^3,88^ Conventional analytical techniques, including ICP-MS and AAS, provide high sensitivity and accuracy; however, their reliance on expensive instrumentation, extensive sample preparation, and skilled personnel limits their suitability for real-time, in-field monitoring.^89,90^ These constraints have driven the development of alternative sensing platforms capable of delivering high analytical performance while remaining portable, cost-effective, and operationally simple.

### 3.1. The ASSURED framework and sensor design requirements

The WHO introduced the ASSURED criteria, as a benchmark for point-of-care diagnostics and environmental monitoring technologies.^91^ These criteria impose stringent constraints on sensor design, particularly for heavy metal detection, where regulatory thresholds are exceptionally low. Current drinking water guidelines established by the WHO and the US EPA limit concentrations of Pb^2+^ and As^3+^/As^5+^ to 10 µg L^−1^ and Hg^2+^ to approximately 2 µg L^−1^.^11,60,92^ Sensors must therefore achieve LODs at or below these values while maintaining selectivity in complex matrices containing high concentrations of benign ions such as Ca^2+^, Mg^2+^, Na^+^, and Fe^3+^. QDs satisfy these requirements owing to their large molar extinction coefficients, narrow emission bandwidths, and size-dependent photoluminescence.^93,94^ The high surface-to-volume ratio of QDs enables dense functionalisation with chelating ligands, aptamers, or ion-imprinted polymers, imparting molecular-level selectivity consistent with hard–soft acid–base (HSAB) theory.^95^ Fluorescence-based QD sensors often operate *via* direct mix-and-detect protocols, producing rapid optical responses that can be captured using portable fluorimeters or smartphone-integrated systems.^96^ The correspondence between the WHO ASSURED criteria and the key design advantages offered by QD-based sensing platforms for heavy metal detection is summarised in Table 2.

**Table 2:** ASSURED criteria and their technical implications for QD-based heavy metal sensing

ASSURED criterion	Technical implication	QD platform advantage
Affordable	Low-cost analysis, scalable fabrication	Biomass-derived QDs; paper substrates
Sensitive	Detection at regulatory limits	High QY and extinction coefficients
Specific	Minimal ionic interference	Selective surface ligands
User-friendly	Minimal sample preparation	Visual or ratiometric fluorescence
Rapid/robust	Minutes-scale analysis	Fast charge and energy transfer
Equipment-free	Portable operation	Smartphone-based readout
Deliverable	Field-deployable systems	Paper and microfluidic platforms

### 3.2. Fundamental photophysics of QDs

QDs are semiconductor nanocrystals, typically 2–10 nm in diameter, whose electronic and optical properties deviate markedly from those of bulk materials due to quantum confinement effects. When particle dimensions approach the exciton Bohr radius, charge carriers are confined in all three spatial dimensions, resulting in discrete energy levels.

#### 3.2.1. Quantum confinement and exciton dynamics

The electronic and optical properties of QDs arise fundamentally from the spatial confinement of charge carriers within semiconductor nanocrystals whose dimensions approach or fall below the exciton Bohr radius. In bulk semiconductors, electrons and holes move freely throughout the lattice, and their energy states form quasi-continuous bands separated by a characteristic band gap. However, when the physical dimensions of the semiconductor are reduced to the nanometre scale, the de Broglie wavelength of the charge carriers becomes comparable to the particle size, and the wave-like nature of electrons begins to dominate their behaviour.^94^ Under these conditions, the allowed energy states transition from continuous bands to discrete, atom-like levels, a phenomenon that can be understood through the quantum mechanical model of a particle confined within a three-dimensional potential well. The confinement energy that emerges from this spatial restriction adds to the intrinsic bulk band gap and can be described by the Brus equation:^97^
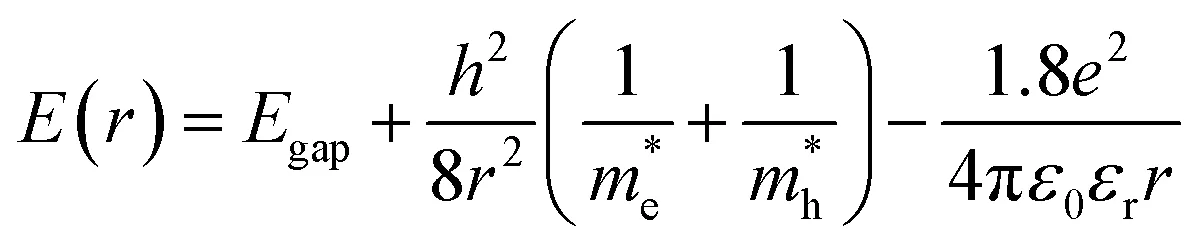
where *E*_gap_ is the bulk band gap energy, *h* is Planck's constant, *r* is the QD radius, 
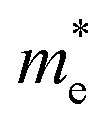
 and 
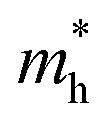
 are the effective masses of the electron and hole respectively, *e* is the elementary charge, *ε*_0_ is the permittivity of free space, and *ε*_r_ is the relative dielectric constant of the semiconductor. The second term represents the quantum confinement energy arising from the particle-in-a-box contribution, whilst the third term accounts for the Coulomb attraction between the electron and hole. This inverse-square dependence of the confinement energy on radius explains the characteristic size-dependent optical properties of QDs: smaller particles exhibit larger effective band gaps and correspondingly blue-shifted emission, whilst larger particles display narrower band gaps and red-shifted emission.^98^

The exciton Bohr radius serves as the critical length scale that determines the onset and strength of quantum confinement effects. This parameter represents the natural spatial extent over which an electron–hole pair would delocalise in an unconfined bulk semiconductor and is expressed as:^99^
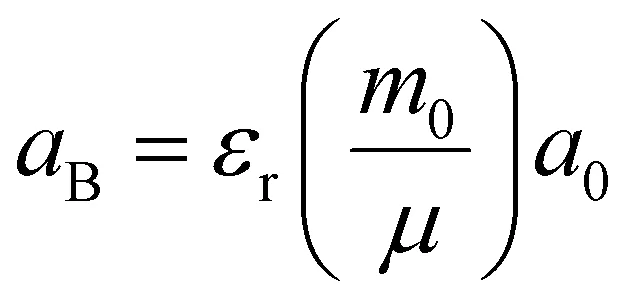
where, *ε*_r_ is the relative dielectric constant, *m*_0_ is the free electron mass, *µ* is the reduced effective mass of the exciton, and *a*_0_ is the Bohr radius of the hydrogen atom (0.053 nm). Materials with high dielectric constants screen Coulomb interactions more effectively, weakening the electrostatic attraction between electrons and holes and thereby increasing the exciton Bohr radius. Similarly, semiconductors in which charge carriers possess lighter effective masses, a consequence of band structure curvature, exhibit larger exciton Bohr radii because the carriers are inherently more delocalised. These material-dependent variations result in substantially different confinement thresholds across semiconductor families; for instance, cadmium selenide possesses an exciton Bohr radius of approximately 5.6 nm, cadmium sulphide approximately 2.5 nm, whereas lead sulphide exhibits a considerably larger value of approximately 18–20 nm.^100^ Consequently, lead chalcogenide QDs can manifest strong confinement effects even at relatively large particle sizes compared to II–VI materials.

The ratio of the QD radius to the exciton Bohr radius delineates three distinct confinement regimes, each characterised by different physical behaviours. In the strong confinement regime, where the nanocrystal radius is substantially smaller than the exciton Bohr radius, the electron and hole are confined independently of one another, and the Coulomb interaction between them becomes a secondary perturbation to the dominant kinetic confinement energy. This regime, in which energy levels scale predictably with the inverse square of the particle radius, describes the behaviour of most colloidal QDs employed in sensing applications. The intermediate confinement regime emerges when the nanocrystal dimensions are comparable to the exciton Bohr radius, creating a situation in which kinetic confinement and Coulomb interactions contribute comparably to the overall energetics and necessitating more sophisticated theoretical treatments such as variational or numerical computational approaches. In the weak confinement regime, applicable when the QD radius significantly exceeds the exciton Bohr radius, the electron and hole remain bound as an excitonic quasi-particle whose centre-of-mass motion, rather than internal structure, is affected by the spatial boundaries of the nanocrystal.

Beyond the fundamental size-dependent band gap, the lowest excitonic states in strongly confined QDs exhibit a fine structure arising from several physical interactions.^101^ The electron–hole exchange interaction splits the exciton manifold according to spin configuration, whilst crystal field effects originating from non-cubic lattice symmetries, such as the wurtzite structure commonly adopted by cadmium selenide, and shape anisotropies associated with deviations from perfect sphericity further lift degeneracies within the excitonic states.^102^ This fine structure creates a manifold of energetically proximate states, among which the lowest-energy state is frequently optically “dark” due to spin selection rules that render radiative recombination forbidden.^103^ The energy separation between this dark ground state and the lowest optically active bright state, typically ranging from 1 to 20 meV depending on the material system and particle size, profoundly influences the emission dynamics of QDs. Photogenerated carriers thermalise rapidly to the dark state, resulting in extended radiative lifetimes at cryogenic temperatures and introducing temperature-dependent variations in QY as thermal energy becomes sufficient to populate the emissive bright states.^104^

#### 3.2.2. The Brus equation: size-dependent band gap

The predictive power of the Brus equation has been extensively validated across numerous semiconductor systems, demonstrating remarkable agreement between calculated and experimentally measured band gap energies for QDs spanning a wide range of compositions and sizes.^105^ However, the equation exhibits certain limitations that become apparent under specific conditions. The effective mass approximation upon which it is based assumes parabolic energy bands and becomes less accurate for very small nanocrystals where the discrete atomic nature of the lattice becomes significant and non-parabolicity effects emerge.^106^ Additionally, the model treats the nanocrystal surface as an infinite potential barrier, neglecting the finite depth of the actual confining potential and any surface-related perturbations to the electronic structure. Despite these limitations, the Brus equation remains an invaluable tool for predicting and rationalising the size-tunable optical properties of QDs, providing the theoretical foundation for the rational design of QD-based sensing platforms where precise control over absorption and emission wavelengths is essential for multiplexed detection schemes.^107^Table 3 presents a comparative overview of representative semiconductor QDs commonly employed in sensing applications, summarising their fundamental photophysical parameters including bulk band gap energies, exciton Bohr radii, and accessible emission ranges. The selection encompasses both cadmium-based (CdS, CdSe) and cadmium-free (ZnS, InP, PbS) systems, reflecting the current landscape of QD materials spanning ultraviolet to mid-infrared spectral regions.

**Table 3:** Representative semiconductor QDs and photophysical parameters

Semiconductor	Bulk band gap (eV)	Exciton Bohr radius (nm)	Tunable emission range	Typical QY (%)	Common shell	Toxicity
ZnS^108^	3.68	∼2.5	UV–Blue (300–450 nm)	20–40	—	Low
CdS^109^	2.42	∼2.9	Blue–Green (380–500 nm)	30–50	ZnS	High
CdSe^105^	1.74	∼5.6	Green–Red (450–650 nm)	50–85	ZnS, CdS	High
InP^110^	1.35	∼11.0	Red–NIR (600–900 nm)	40–70	ZnS, ZnSe	Low
PbS^100^	0.41	∼18.0	NIR–MIR (900–3000 nm)	30–60	CdS	Moderate

These photophysical parameters translate directly into sensing performance. Smaller QDs, with their larger surface-to-volume ratio, present a proportionally greater density of surface trap states and unpassivated dangling bonds; these act as non-radiative recombination centres that lower quantum yield but simultaneously provide a higher density of accessible coordination sites for heavy-metal ion binding, directly influencing achievable LOD and sensitivity. Materials with larger exciton Bohr radii, such as PbS, exhibit strong confinement effects across a wider size range, allowing finer tuning of the emission wavelength for multiplexed detection without requiring sub-2 nm particle sizes that are difficult to synthesise reproducibly. Conversely, materials with high native toxicity (CdSe, CdS; Table 3) but favourable photophysics illustrate the trade-off between analytical performance and the environmental and safety considerations.

Beyond the traditional cadmium- and lead-based semiconductor QDs summarised in Table 3, several alternative material classes offer differing trade-offs between analytical performance and environmental burden. Perovskite QDs (*e.g.*, CsPbX_3_, CH_3_NH_3_PbX_3_) achieve superior photoluminescence quantum yields and tunable emission, but pristine lead-based variants carry high absolute lead content and can release toxic ions into the environment if the host matrix is compromised during disposal or device failure. Lead-free perovskite alternatives (Cs_3_Bi_2_X_9_, CsSnX_3_) address this toxicity concern but exhibit poor aqueous durability, with unprotected materials degrading within 24 hours of water exposure; surface strategies such as zwitterionic polymer coating and MOF encapsulation have extended aqueous stability to over 30 days, a roughly 30-fold improvement, though at added synthetic complexity. Carbon-based alternatives, including CQDs and GQDs, offer favourable biocompatibility, chemical stability, and low intrinsic toxicity, but generally exhibit lower quantum yields and less precise emission tunability than semiconductor QDs. Cadmium-free semiconductor alternatives (InP/ZnSe/ZnS, ZnSeTeS) have achieved substantial performance gains, with photoluminescence quantum yields exceeding 90% reported for InP red emitters, yet their adoption in heavy-metal sensing applications continues to lag cadmium-based systems in both quantum yield and mechanistic characterisation. A further regulatory consideration applies specifically to cadmium-based sensors: many platforms designed to detect Cd^2+^ themselves rely on cadmium-containing QDs, creating an inherent tension with environmental and health regulations such as RoHS and REACH; some manufacturers have been documented exploiting RoHS thresholds, which are expressed as parts-per-million concentrations, by increasing component thickness or mass to dilute reported cadmium content while maintaining high absolute quantities. These trade-offs in performance, aqueous stability, and disposal-related environmental risk across QD material classes are discussed in greater mechanistic and practical detail throughout this review and are treated comprehensively for perovskite QD-based nanosensors by Mohammad *et al.*^111^

Beyond acute toxicity and aqueous stability, the environmental burden of QD-based sensors over their full life cycle, spanning raw material extraction, synthesis, in-use degradation, and end-of-life disposal, differs substantially across material classes. Conventional cadmium- and lead-based semiconductor QDs are not biodegradable: their inorganic crystalline core persists in the environment following device disposal, and degradation pathways generally proceed *via* oxidative dissolution of the core rather than breakdown into non-toxic constituents, releasing the constituent heavy metal ions into the surrounding environment. Carbon-based QDs (CDs, GQDs), by contrast, are generally considered more readily biodegradable owing to their carbon-dominated composition, though biodegradation rates depend strongly on the specific surface functionalisation and synthesis precursor and remain incompletely characterised for most reported sensing formulations. Heavy-metal-free semiconductor alternatives (InP, ZnS-based systems) occupy an intermediate position: their inorganic cores are similarly non-biodegradable, but the absence of cadmium or lead substantially reduces the toxicity of any released degradation products. Prospective life-cycle modeling of QD nanoparticles, while so far more extensively developed for other QD application areas such as photon upconversion devices, offers a methodological framework that has yet to be widely applied to QD-based heavy-metal sensors specifically; extending such life-cycle assessment to quantify the comparative raw-material, energy, and end-of-life burdens of cadmium-based, carbon-based, and heavy-metal-free QD sensing platforms represents an important direction for future work.^112^

### 3.3. Synthesis methodologies: top-down and bottom-up approaches

The physicochemical properties of QDs, including their size distribution, crystallinity, surface chemistry, and consequent optical characteristics, are profoundly influenced by the synthetic methodology employed for their fabrication. Broadly, QD synthesis strategies can be categorised into top-down and bottom-up approaches.^113^ Top-down approaches involve the systematic reduction of bulk semiconductor materials into nanoscale structures through physical or mechanical processes such as electron-beam lithography, focused ion beam milling, and reactive ion etching; among these, pulsed laser ablation, wherein high-intensity pulsed laser irradiation fragments bulk targets into nanoparticulate dispersions, is most directly applicable to QD sensor fabrication owing to its relative simplicity and lack of complex chemical precursor requirements.^114^ However, top-down methodologies frequently suffer from broad size distributions, irregular morphologies, and surface defects arising from the energetic fragmentation process, which act as non-radiative recombination centres that diminish photoluminescence quantum yield and compromise sensing performance.

Bottom-up approaches, by contrast, construct QDs through the controlled assembly of atomic or molecular precursors, affording superior control over particle size, shape, crystallinity, and surface chemistry,^115^ and account for the great majority of QDs used in heavy-metal sensing. Colloidal synthesis is the most widely employed bottom-up methodology; the hot-injection method, pioneered by Murray, Norris, and Bawendi, involves rapid injection of organometallic precursors into a hot coordinating solvent, inducing burst nucleation followed by controlled growth.^105^ This approach yields highly monodisperse nanocrystals with size distributions below 5% relative standard deviation, translating to narrow emission linewidths essential for multiplexed sensing. Alternative colloidal approaches include heat-up synthesis and aqueous synthesis routes employing water-soluble thiols or polymers as stabilising ligands.^116^ The choice of methodology depends on the intended application; sensing platforms typically demand QDs with narrow size distributions, high quantum yields, excellent photostability, and surfaces amenable to functionalisation with recognition elements.

### 3.4. Green synthesis and sustainable trends (2020–2025)

The period 2020–2025 has seen a pronounced shift toward environmentally sustainable QD synthesis, driven by concerns over the toxicity of conventional cadmium- and lead-containing nanocrystals and the hazardous precursors and solvents traditionally used in their fabrication.^117^ Green synthesis methodologies minimise or eliminate toxic reagents, reduce energy consumption, and employ renewable or waste-derived carbon sources. Biomass-derived CDs are particularly attractive, being synthesised from abundant, low-cost precursors including agricultural waste, fruit peels, and plant extracts.^118^ Hydrothermal or microwave-assisted carbonisation of these feedstocks generates CDs bearing oxygen- and nitrogen-containing surface functional groups (hydroxyl, carboxyl, carbonyl, amine), conferring enhanced aqueous dispersibility, improved colloidal stability across a broad pH range, and intrinsic metal-ion binding capability without additional surface modification. Heteroatom doping modulates the electronic structure and sensing performance of CDs:^119^ nitrogen doping introduces electron-rich sites for selective interaction with electron-deficient metal cations while shifting photoluminescence energy states; sulphur doping enhances affinity toward soft metal ions such as Hg^2+^ and Ag^+^*via* soft–soft interactions; phosphorus doping improves quantum yield and adds coordination sites. Co-doping (*e.g.*, N/S or N/P) synergistically combines these effects, creating multiple binding sites with complementary selectivity. Green synthesis principles have also been extended to conventional semiconductor QDs *via* aqueous routes using non-toxic stabilisers and the substitution of cadmium-based systems with inherently less toxic alternatives such as indium phosphide, copper indium sulphide, and zinc-based QDs.^110^ Beyond their favourable toxicity profile, hydrothermal and microwave-assisted routes are inherently more scalable than hot-injection synthesis, tolerating larger reaction volumes without the precise injection control required for burst nucleation.^120,121^ However, batch-to-batch reproducibility remains a practical constraint: natural biomass precursors vary in composition with source, season, and processing history, which can propagate into variable quantum yield and surface functional group density between synthesis batches unless feedstocks are standardised.

To facilitate direct comparison of the synthesis methods discussed above with respect to industrial and practical viability, Table 4 summarizes their relative scalability, reproducibility, cost, and industrial feasibility.

**Table 4:** Comparison of QD synthesis methods relevant to heavy-metal sensing applications

Method	Scalability	Reproducibility	Relative cost	Industrial feasibility
Top-down (laser ablation)^122^	Low–moderate	Moderate (broad size distributions)	Low (no complex precursors)	Low (limited to niche/prototyping use)
Hot-injection (bottom-up)^123^	Low	High (<5% RSD size distribution)	High (precise temperature/injection control)	Low (batch process, difficult scale-up)
Heat-up/aqueous (bottom-up)^124^	Moderate	Moderate	Low–moderate	Moderate
Hydrothermal^125^	High	Moderate (precursor-dependent)	Low	High
Microwave-assisted^126^	High	Moderate–high	Low–moderate	Moderate–high (needs specialized equipment)
Green/biomass-derived^127^	High	Low–moderate (natural precursor variability)	Low	High (renewable feedstock)
Continuous-flow^128^	High	High	Moderate (equipment investment)	High

### 3.5. Surface engineering: passivation and functionalisation

The surface chemistry of QDs decisively influences their optical properties, colloidal stability, and sensing performance.^129^ Freshly synthesised QDs typically possess incompletely coordinated surface atoms that introduce mid-gap electronic “trap states”, acting as non-radiative recombination pathways that diminish photoluminescence quantum yield. Unpassivated surfaces are also susceptible to oxidation, photooxidation, and other degradation processes that compromise long-term photostability and reproducibility. Surface passivation strategies aim to eliminate or minimise these deleterious states, enhancing both the brightness and stability of QD emission. Core–shell architectures are the most effective passivation approach, in which a thin epitaxial layer of a wider band gap semiconductor is grown around the emissive core. The archetypal CdSe/ZnS system, with a ZnS shell (bulk band gap 3.68 eV) encapsulating the CdSe core, confines electrons and holes within the core and physically isolates it from the external environment. This type-I band alignment effectively eliminates surface trap states, yielding quantum yields exceeding 80% and markedly improved resistance to photobleaching and chemical degradation. Alternative shell materials, including CdS, ZnSe, and graded or alloyed compositions, optimise lattice matching and interfacial strain to further enhance optical properties.^130^ Beyond passivation, surface functionalisation with specific recognition elements is essential for imparting selectivity toward target metal ions in complex matrices.^131^ Thiol-containing ligands (mercaptopropionic acid, mercaptosuccinic acid, cysteine, GSH) are widely employed owing to the strong affinity of thiol groups for soft metal surfaces and their ability to displace native hydrophobic ligands, rendering QDs water-dispersible while providing carboxylate or amine groups for metal-ion binding or further conjugation.

Heteroatom doping represents a complementary strategy to ligand-based functionalisation for tuning QD sensing performance, operating by modifying the electronic structure of the QD itself rather than only its outer surface chemistry. As discussed in the context of carbon dot synthesis, nitrogen, sulphur, and phosphorus doping introduce electron-rich sites, soft-donor coordination centres, and additional surface coordination groups, respectively, each altering metal-ion binding affinity and selectivity in a manner distinct from post-synthetic ligand exchange. Because dopant atoms are incorporated during synthesis rather than attached afterward, doping can achieve a more uniform and stable distribution of metal-ion binding sites across the QD surface than ligand functionalisation alone, though at the cost of reduced post-synthetic tunability, since the doping level is fixed once synthesis is complete. Co-doping strategies, combining two heteroatom types, can be viewed as an extension of the multi-ligand functionalisation approaches discussed above, creating multiple binding-site types with complementary selectivity within a single QD architecture.^132^

For applications demanding enhanced selectivity, QDs can be functionalised with chelating agents, crown ethers, calixarenes, or biomolecular recognition elements including aptamers, DNAzymes, and peptides.^133^ Aptamer conjugation to QD surfaces is typically achieved through one of several established chemistries: thiol-terminated aptamers form direct dative bonds with metal-based QD surfaces (analogous to thiol-ligand capping); carboxyl- or amine-terminated aptamers are coupled *via* EDC/NHS carbodiimide chemistry to complementary functional groups introduced during surface passivation; and biotin-streptavidin linkage offers a modular, non-covalent alternative when QDs are pre-functionalized with streptavidin. The conjugation strategy influences both aptamer orientation and surface density, which in turn affect target accessibility and binding kinetics. Beyond the general selectivity advantage of aptamer-based recognition noted above, aptamer conjugation has enabled architecturally sophisticated sensing platforms with performance benefits distinct from simple ligand functionalisation: rigid three-dimensional scaffolds such as DNA tetrahedra, which anchor the aptamer at multiple points to enforce a defined orientation and enhance binding efficiency relative to linear aptamer probes, have achieved sensitivity comparable to ICP-MS while providing inherent self-calibration through dual-emission QD architectures. DNAzyme-based conjugation extends this approach further, coupling target-ion recognition to catalytic signal amplification rather than simple stoichiometric binding, improving achievable sensitivity beyond what direct aptamer-QD binding alone provides. Chemical etching approaches have also been used to prepare functional composite QD sensors directly: Luo *et al.* reported fluorescent CdTe QDs embedded within a magnetic Fe_3_O_4_ matrix and molecularly imprinted for Cd(ii) recognition (Fe_3_O_4_@CdTe-IIP), which showed enhanced photoluminescence, magnetic separability, and markedly improved adsorption capacity and selectivity toward Cd(ii) relative to the non-imprinted composite, illustrating the combination of surface engineering with molecular imprinting for targeted heavy-metal recognition.^134^

Biointerface design refers to the deliberate engineering of the QD-biomolecule interface as an integrated system, rather than treating surface ligands, recognition elements, and the underlying QD core as independently optimised components. This holistic approach addresses a practical tension in QD sensor design: recognition elements that maximise selectivity (aptamers, antibodies, imprinted polymers) often require surface chemistries or spacer architectures that can compromise the QD's intrinsic photophysical properties, while surface modifications optimised purely for photoluminescence stability may provide a poor scaffold for biomolecular recognition. Protein-mediated biointerfaces, in which the QD core is synthesised or capped in the presence of a biocompatible protein such as human serum albumin, illustrate this integration: the protein simultaneously serves as a colloidal stabiliser, a biocompatibility-conferring shell, and a source of intrinsic metal-binding functional groups (*e.g.*, thiol-containing cysteine residues), combining roles that would otherwise require separate ligand and recognition-element layers. Molecularly imprinted composites, in which a recognition cavity complementary to the target ion is formed directly within a magnetic or silica matrix surrounding the QD, represent a related strategy for co-designing the physical support structure and the recognition interface as a single functional unit, rather than adding recognition chemistry onto a pre-formed QD surface. These biointerface strategies illustrate that surface engineering for QD-based heavy-metal sensing is increasingly approached as the co-design of stability, biocompatibility, and recognition function, rather than as sequential, independently optimised steps.^135^

### 3.6. Signal transduction and analytical performance

QD-based sensors transduce the binding of target metal ions into measurable optical or electrochemical signals through a variety of physicochemical mechanisms, the selection of which profoundly influences the sensitivity, selectivity, dynamic range, and practical applicability of the resulting sensing platform.^136,137^ Fluorescence-based detection modes predominate in QD sensing owing to the exceptional brightness, photostability, and size-tunable emission characteristics of these nanomaterials. The most exploited transduction mechanism is fluorescence quenching, wherein the interaction between surface-bound metal ions and the QD results in diminished photoluminescence intensity. Quenching can proceed through several distinct pathways, including non-radiative energy transfer to the bound metal ion, photoinduced electron transfer (PET) between the QD and the metal species, or the introduction of new non-radiative recombination pathways through metal-induced surface trap states.^22^ Heavy metal ions with unfilled d-orbitals, such as copper(ii), iron(iii), and mercury(ii), are particularly effective quenchers due to their ability to accept electrons from photoexcited QDs or to facilitate energy transfer processes.

Chelation-enhanced fluorescence (CHEF) represents an alternative transduction mechanism wherein metal ion binding induces an increase rather than a decrease in photoluminescence intensity. This “turn-on” response is typically achieved by designing systems in which the native fluorescence of the QD is initially suppressed by a quencher moiety tethered to the surface; upon metal ion binding, conformational changes or displacement of the quencher restore emission. Turn-on sensors offer practical advantages over turn-off systems, as the positive signal response is less susceptible to false positives arising from non-specific quenching by matrix components. FRET provides a powerful mechanism for ratiometric sensing, wherein the relative intensities of donor and acceptor emission serve as the analytical signal rather than absolute intensity changes.^31^ In FRET-based QD sensors, the QD typically serves as the energy donor, with an organic fluorophore, another QD of different size, or a quencher molecule functioning as the acceptor. Metal ion binding modulates the donor–acceptor distance or orientation, thereby altering the FRET efficiency and producing characteristic spectral changes. Ratiometric detection offers inherent self-referencing capabilities that compensate for fluctuations in excitation source intensity, optical path length, and probe concentration, enhancing measurement reproducibility and enabling more reliable quantification in complex sample matrices. Photoelectrochemical (PEC) sensing represents a related modality wherein QDs serve as photoactive electrode materials, and metal ion binding modulates the photocurrent generated upon illumination.^138^ This approach combines the selectivity advantages of molecular recognition with the sensitivity and instrumental simplicity of electrochemical detection, offering LODs competitive with the most sensitive optical methods whilst requiring less sophisticated instrumentation.

Table 5 summarizes the comparative strengths and limitations of these four sensing mechanisms under practical operating conditions, addressing typical detection limit range, robustness to matrix interference, instrumentation complexity, and field deployability. These trade-offs suggest that mechanism selection should be guided by the intended deployment context rather than sensitivity alone. Simple turn-off quenching offers the lowest barrier to field deployment, given its compatibility with basic optical readout, but is correspondingly most vulnerable to false-positive responses in complex, multi-ion matrices. Ratiometric FRET-based designs trade increased synthetic and architectural complexity for substantially improved measurement reliability under variable real-world conditions, making them well suited to applications where matrix variability is significant but portable instrumentation remains acceptable. Photoelectrochemical sensing achieves sensitivity and interference robustness competitive with the best optical approaches, but its reliance on electrode fabrication and potentiostatic instrumentation currently limits its suitability for simple, visual, or fully portable field deployment relative to fluorescence-based alternatives.

**Table 5:** Comparative strengths and limitations of QD sensing mechanisms under practical conditions

Mechanism	Typical LOD range	Robustness to matrix interference	Instrumentation complexity	Field deployability	Key limitation
Fluorescence quenching (turn-off)^139^	pM–µM	Low–moderate (susceptible to non-specific quenching by matrix components)	Low (basic fluorimeter or smartphone camera)	High	False positives from non-target quenchers; cannot distinguish quenching cause
CHEF (turn-on)^140^	nM–µM	Moderate	Low	High	Requires pre-quenched baseline state; more complex probe design
FRET (ratiometric)^141^	pM–nM	High (self-referencing corrects for excitation/path-length/concentration drift)	Moderate (dual-emission detection)	Moderate–high	Requires donor–acceptor spectral overlap and proximity control; more complex synthesis
Photoelectrochemical (PEC)^142^	pM–nM	Moderate–high	High (electrode fabrication, potentiostat)	Low–moderate	Requires specialized instrumentation, less amenable to simple portable/visual readout

## 4. Mechanism of heavy metal detection using QDs

QDs constitute a versatile sensing platform for heavy metal ion detection owing to their size-dependent photophysical properties, exceptionally high surface-to-volume ratios, and amenability to surface engineering^143,144^ Various types of QD materials are employed in heavy metal sensing applications, each offering distinct advantages in terms of optical properties, biocompatibility, and synthesis complexity. Table 6 summarizes the principal QD materials used in heavy metal detection, including their compositional characteristics, optical properties, and practical considerations. Detection using QDs is governed by physicochemical interactions between target analytes and the QD surface, which are transduced into measurable optical or electrochemical signals. These sensing mechanisms can be broadly categorised into fluorescence-based, energy-transfer-based, and electrochemical or PEC pathways, each offering distinct advantages depending on analytical requirements and deployment contexts.^143,145^Table 7 summarizes the key characteristics, advantages, and applications of each detection mechanism, which are discussed in detail in the following subsections.^142,146–154^ The principal signal transduction mechanisms underlying QD-based heavy metal sensing are summarised schematically in Fig. 3. These include fluorescence quenching and chelation-enhanced fluorescence, which rely on modulation of QD photoluminescence, FRET for ratiometric optical detection, and electrochemical quenching processes that convert metal ion binding events into changes in electrochemical response.

**Table 6:** QD material types and properties

QD material type	Composition	Size range	Emission range	Key advantages	Limitations & considerations
CdTe QDs^155^	CdTe core; thiol-capped	2–6 nm	500–650 nm (green to red)	High QY (30–80%); well-established synthesis; excellent size-tunability; aqueous synthesis available	Cd toxicity concerns; oxidation sensitivity; requires careful surface passivation
CdSe/ZnS core–shell^156^	CdSe core with ZnS shell	2–10 nm	450–650 nm (blue to red)	Very high QY (50–90%); excellent photostability; reduced core photooxidation; commercial availability	Organic solvent synthesis; requires phase transfer for aqueous use; Cd toxicity
CDs^145^	Carbonaceous NPs; surface functional groups	2–10 nm	400–600 nm (blue to green); often multi-emission	Low toxicity; biocompatible; simple synthesis (hydrothermal, microwave); low cost; dual-emission for ratiometric sensing	Lower QY (10–40%); broad emission peaks; batch-to-batch variability; complex surface chemistry
GQDs^157^	Graphene nanosheets with quantum confinement	3–20 nm	400–600 nm; excitation-dependent	Excellent biocompatibility; high chemical stability; tunable *via* edge functionalization; good electron transfer properties	Complex synthesis optimization; moderate QY; requires careful size control
Lanthanide-doped NPs^158^	NaYF_4_:Yb/Er, NaGdF_4_:Eu^3+^	20–100 nm	Multiple sharp emission lines (480–660 nm)	No photobleaching; sharp emission bands ideal for FRET; long luminescence lifetime; upconversion capability	Larger size; lower extinction coefficient; requires NIR excitation for upconversion; complex synthesis
InP/ZnS QDs^159^	Indium phosphide core with ZnS shell	2–6 nm	500–700 nm (green to red)	Cd-free (reduced toxicity); similar optical properties to CdSe; suitable for biological applications	Lower QY (40–70%); more challenging synthesis; higher cost; less commercially available
Ag_2_S QDs^160^	Silver sulfide	2–8 nm	Near-infrared (900–1400 nm)	NIR emission (tissue penetration); low toxicity; suitable for *in vivo* applications	Limited applicability for visible-range detection; oxidation sensitivity; emerging technology
Silicon QDs^161,162^	Si core; surface-oxide or organic-capped	2–5 nm	400–800 nm (size-tunable)	Low intrinsic toxicity; earth-abundant, low-cost precursor; biocompatible; intrinsic up-conversion capability suppresses background autofluorescence	Lower QY than Cd-based systems; synthesis often requires air-free conditions; surface oxidation can degrade emission over time
Perovskite QDs (CsPbX_3_/lead-free variants)^163–166^	Lead-based: CsPbX_3_, CH_3_NH_3_PbX_3_; Lead-free: Cs_3_Bi_2_X_9_, CsSnX_3_	2–10 nm	410–800 nm (halide-composition tunable)	Very high QY (50–90% lead-based); narrow emission (high color purity); rapid response (<10 s)	Lead-based variants carry high absolute lead content and poor aqueous stability without encapsulation; lead-free variants trade improved sustainability for lower QY (20–50%) and Sn^2+^ oxidation instability

**Table 7:** Main detection mechanisms

Detection mechanism	Principle/description	Key advantages	Typical metal ions detected	LOD range	Response time	Selectivity	Cost	Applications
Fluorescence quenching (static)^167^	Ground-state complexes between surface ligands and metal ions; electronic structure alteration reduces emissive species; non-radiative decay pathways	High-affinity interactions (*K*_SV_ 10^4^–10^6^ M^−1^); diagnostic *via* fluorescence lifetime measurement; linear Stern–Volmer plots; simple instrumentation	Hg^2+^, Cu^2+^, Fe^3+^, Ag^+^ (paramagnetic, unfilled d-orbitals); mechanism	Nanomolar to sub-nanomolar (10^−7^–10^−9^ M)	Seconds to minutes	Moderate to high depending on ligand design	Low to moderate	Environmental monitoring; rapid field screening; laboratory analysis
Fluorescence quenching (dynamic)^167^	Diffusion-controlled collisional encounters during excited-state lifetime; reduces both emission intensity and fluorescence lifetime proportionally; follows Stern–Volmer equation (*F*_0_/*F* = 1 + *K*_SV_[Q])	Concentration-dependent over multiple orders of magnitude; lifetime analysis enables mechanistic differentiation from static quenching; reversible quenching	Hg^2+^, Cu^2+^, Fe^3+^, Pb^2+^ (inner filter effects (IFEs), surface trap state formation, aggregation-induced quenching)	Nanomolar to sub-nanomolar (10^−7^–10^−9^ M)	Milliseconds to seconds	Moderate (non-specific collisional encounters)	Low to moderate	Point-of-use testing; real-time monitoring; portable sensors
Chelation-enhanced/turn-on fluorescence^168,169^	Metal ion binding saturates surface defects, displaces quenching moieties, or induces conformational switching in surface-bound biomolecules; increases photoluminescence intensity from suppressed baseline	Improved signal-to-noise ratios; reduced susceptibility to false positives from non-specific quenching; enhanced visual discrimination for qualitative assessment; reduced instrumental complexity	Zn^2+^ (defect passivation of thiol-capped CdTe); Hg^2+^, Pb^2+^ (thymine-rich oligonucleotides, G-quadruplex aptamers); Cd^2+^, Cu^2+^ (ion-imprinted polymers)	Nanomolar to sub-nanomolar (10^−7^–10^−9^ M)	Seconds to minutes	High when combined with selective chelating ligands (thymine-rich DNA, G-quadruplexes, metallothionein peptides)	Moderate to high (requires engineered surface)	Qualitative detection; visual assessment; field-deployable assays; multiplexed detection with color-coding
FRET & ratiometric detection^170^	Non-radiative dipole–dipole coupling (*r*^−6^ distance dependence); Förster radius 3–8 nm; distance modulation *via* conformational changes, complex dissociation, or spectral overlap modification upon metal ion binding; signal = ratio of two emission bands	Self-referencing capability; independent of absolute fluorescence intensity; compensates for photobleaching, excitation source fluctuations, optical path length variations, and heterogeneous sample matrices; high robustness	Multiple metals *via* donor–acceptor pair engineering; dual-emission CDs (intrinsic multi-wavelength profiles); lanthanide-doped NPs (sharp emission lines); organic fluorophore acceptors (rhodamine, fluorescein); gold NPs (nanometal surface energy transfer)	Sub-nanomolar concentrations (10^−9^–10^−12^ M)	Minutes to hours (conformation-dependent)	High (engineered spectral overlap; spatial separation of donor–acceptor)	Moderate to high	Advanced optical sensing; biomedical diagnostics; environmental analysis; field-deployable ratiometric platforms
Electrochemical transduction (anodic stripping voltammetry)^171^	QD-modified electrodes (glassy carbon, gold, ITO) provide high surface area scaffolds; electrochemical preconcentration followed by anodic stripping; metal ion binding modulates interfacial charge transfer *via* surface charge alteration, metal–ligand complex formation, electroactive species displacement, or redox potential modification	Exceptionally low LODs (femtomolar–picomolar range); facile miniaturization (µm-scale electrodes); low power requirements; multiplexed array formats; label-free detection; reduced instrumental complexity; amenable to portable/battery-operated devices	Pb^2+^, Cd^2+^, Cu^2+^, Zn^2+^, Hg^2+^, As^3+^/As^5+^ (discrimination *via* HSAB theory and computational design)	Femtomolar to picomolar (10^−15^–10^−12^ M)	Seconds to minutes (preconcentration time-dependent)	High *via* rational ligand design; discrimination of chemically similar ions (Cd^2+^*vs.* Zn^2+^) even in 100–1000-fold excess of competing species	Low to moderate (electrode modification required)	Portable water quality monitoring; on-site environmental testing; clinical diagnostics; Internet of Things (IoT) sensor networks
PEC transduction^172^	Optical excitation generates photocurrent; metal ion binding perturbs charge separation *via* electron/hole trapping, band edge position shift, recombination kinetics alteration, or steric blocking of electron acceptor access; spatial separation of optical and electrochemical detection provides low background	Low background signals (simultaneous illumination and electrochemical polarization required); sub-nanomolar sensitivity comparable to advanced spectroscopic techniques; field-portable operation under natural or LED illumination; wavelength-selective multiplexing *via* distinct QD spectral responses; operational simplicity	Multiple metals *via* QD bandgap engineering; simultaneous multi-analyte quantification demonstrated; wavelength-selective illumination enables selective detection in multiplexed formats	Sub-nanomolar concentrations (10^−9^–10^−12^ M)	Seconds to minutes	High *via* wavelength selectivity and surface engineering; spatial patterning enables simultaneous detection of 4+ analytes	Moderate (requires light source and electrode setup)	Multiplexed heavy metal monitoring; field deployment in resource-limited settings; smartphone-integrated diagnostics; environmental surveillance; drinking water quality assessment

**Fig. 3:**
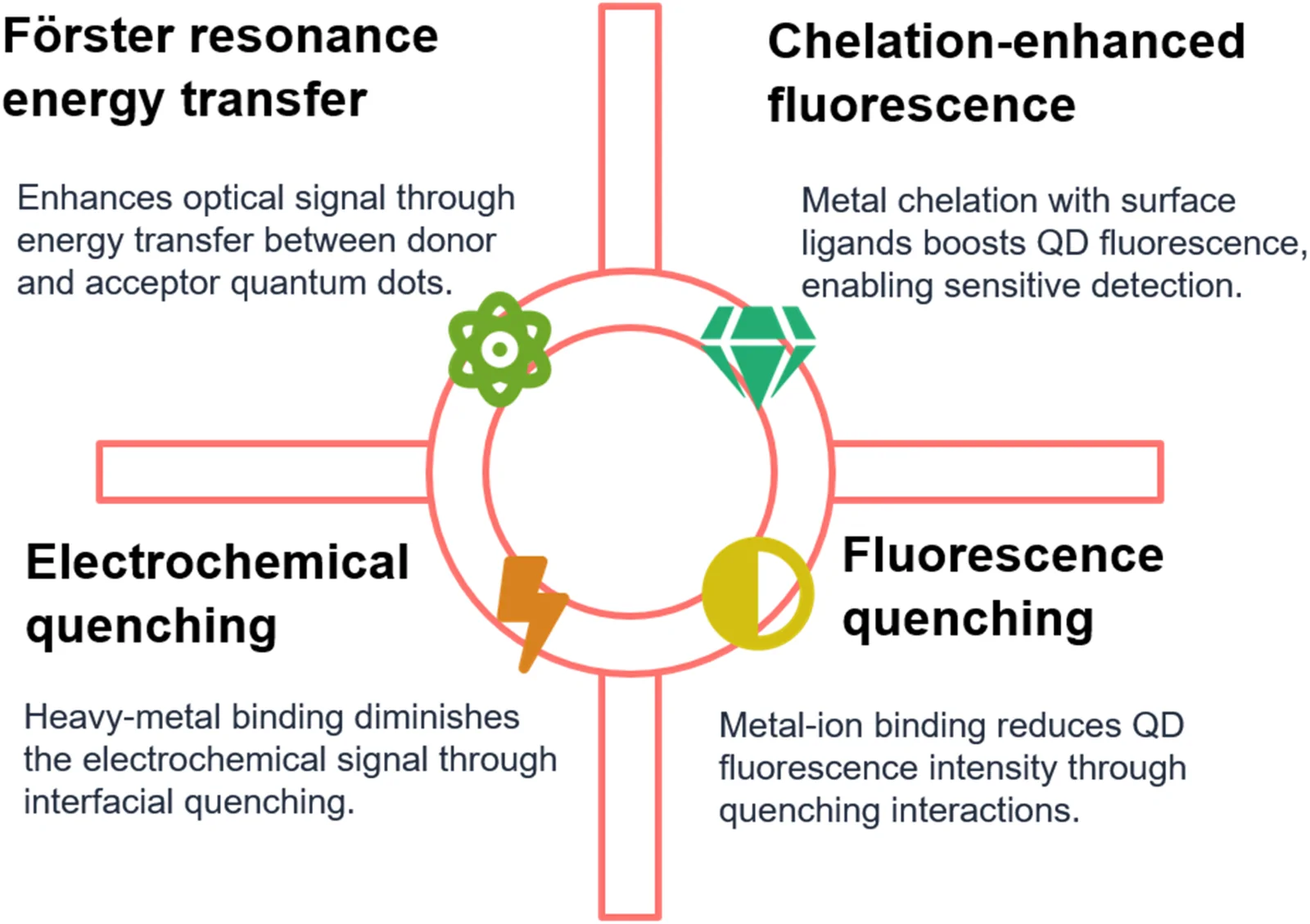
Mechanistic pathways for heavy metal detection using QD-based sensors.

Having established the principal QD material classes and their fundamental photophysical properties in the preceding section, we now turn to the mechanisms by which these materials transduce heavy-metal ion binding into a measurable optical signal. Six mechanisms recur throughout the QD-based heavy-metal sensing literature: photoinduced electron transfer, Förster resonance energy transfer, the inner filter effect, chelation-enhanced fluorescence, redox quenching, and aggregation-induced effects. These mechanisms differ substantially in whether metal-ion binding produces a decrease in fluorescence intensity (quenching), an increase (turn-on enhancement), or a shift in the ratio between two emission bands (ratiometric response), with direct consequences for sensor design, sensitivity, and susceptibility to matrix interference. Fig. 4 summarizes these six mechanisms schematically before each is discussed in mechanistic detail in the sections that follow.

**Fig. 4:**
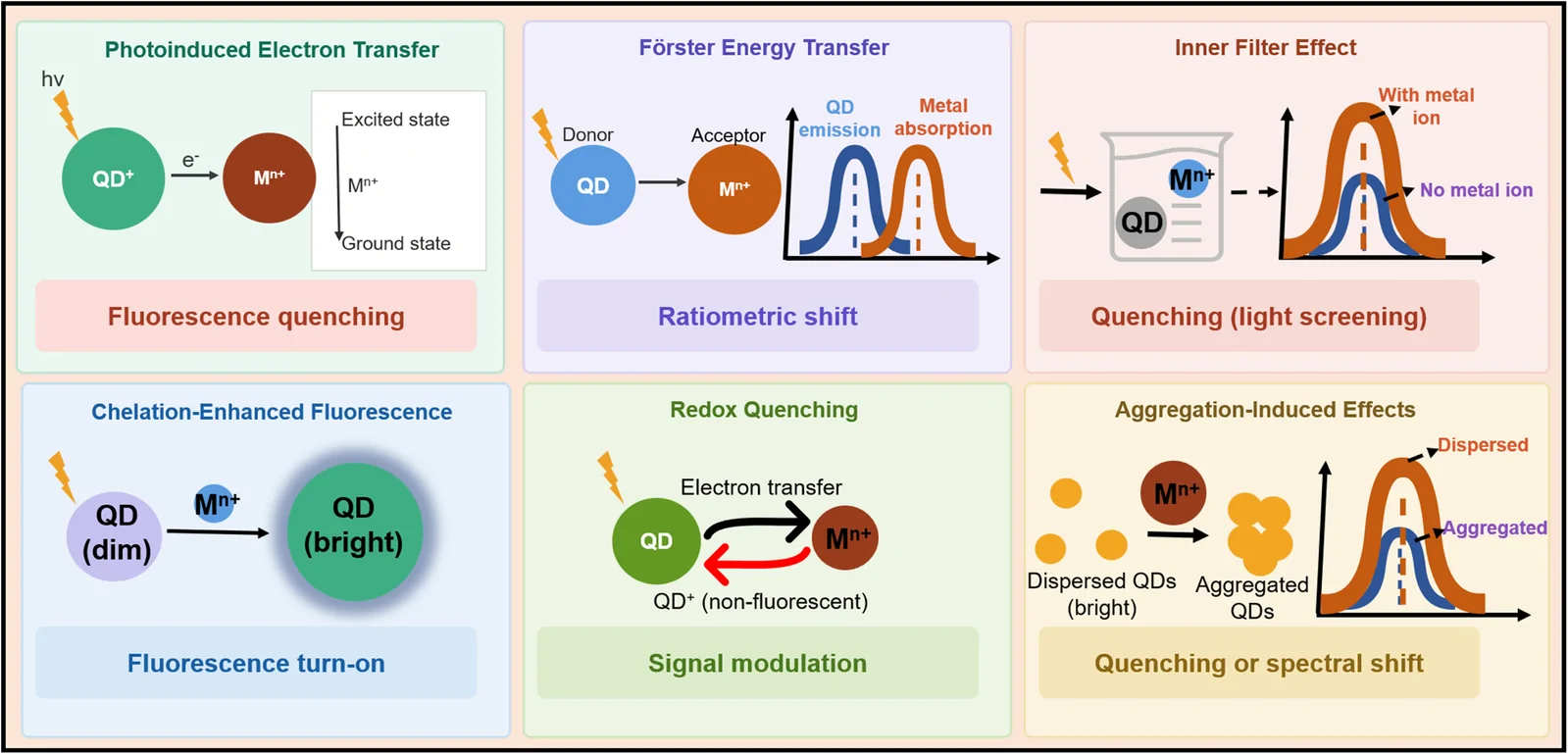
Schematic overview of the six principal mechanisms by which QD fluorescence is modulated upon heavy-metal ion binding.

### 4.1. Fluorescence quenching mechanisms

Fluorescence quenching represents the most extensively exploited mechanism in QD-based heavy metal sensing, underpinning a substantial proportion of reported detection systems.^143,145^ Upon interaction with metal ions, the photoluminescence intensity of QDs decreases through either static or dynamic quenching processes, each governed by distinct physicochemical phenomena. Static quenching arises from the formation of non-emissive ground-state complexes between surface-bound ligands and metal ions. This binding event fundamentally alters the electronic structure of the QD, effectively removing a proportion of emissive species from the excited-state population. Stern–Volmer analysis of static quenching systems typically yields linear plots with quenching constants (*K*_SV_) in the range of 10^4^–10^6^ M^−1^ for high-affinity metal–ligand interactions.^145^ Importantly, static quenching does not alter the fluorescence lifetime of the remaining emissive population, providing a diagnostic criterion for mechanistic assignment.

Dynamic or collisional quenching involves diffusion-controlled encounters between excited QDs and analyte ions during the excited-state lifetime. This process reduces both emission intensity and fluorescence lifetime proportionally, as described by the Stern–Volmer equation:
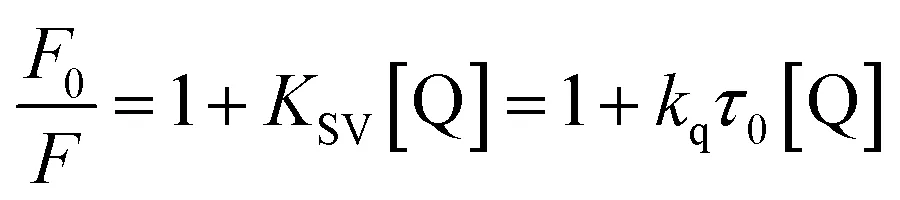
where *F*_0_ and *F* represent fluorescence intensities in the absence and presence of quencher, *K*_SV_ is the Stern–Volmer quenching constant, *k*_q_ is the bimolecular quenching rate constant, *τ*_0_ is the unquenched lifetime, and [Q] is the quencher concentration.^143^

Heavy metal ions including Hg^2+^, Cu^2+^, and Fe^3+^ function as particularly effective quenchers due to their paramagnetic nature and unfilled d-orbitals, which facilitate non-radiative electron or energy transfer pathways.^145^ Several specific quenching mechanisms operate in QD-heavy metal systems, each characterized by distinct physicochemical processes and diagnostic features (Table 8). Understanding these mechanistic pathways is essential for rational sensor design and accurate interpretation of analytical responses occurs when photoexcited electrons transfer from the QD conduction band to vacant orbitals of the bound metal ion, suppressing radiative recombination; this mechanism predominates for ions with favourable reduction potentials, such as Cu^2+^ and Fe^3+^. IFEs arise when metal ions or their complexes absorb excitation or emission wavelengths, reducing apparent fluorescence intensity without direct QD interaction. Surface trap state formation occurs when metal ion binding introduces mid-gap electronic states that trap charge carriers, promoting non-radiative decay pathways. Aggregation-induced quenching results when multivalent metal ions crosslink QDs through surface ligands, inducing aggregation and self-quenching through inter-particle energy transfer. The extent of fluorescence quenching is typically concentration-dependent over several orders of magnitude, enabling quantitative detection with limits of detection frequently reaching nanomolar or sub-nanomolar concentrations.^145^

**Table 8:** Mechanistic quenching pathways

Quenching pathway	Mechanism	Affected parameter	Signal change	Diagnostic feature
Photoinduced electron transfer (PET)	Photoexcited QD electrons transfer to bound metal ion vacant orbitals; suppresses radiative recombination	Fluorescence intensity; QY	Decreased intensity	Primarily intensity change; minimal lifetime reduction
Inner filter effect (IFE)	Metal ions or complexes absorb excitation or emission wavelengths; reduces apparent fluorescence	Apparent intensity	Decreased intensity	No direct QD interaction; wavelength-dependent absorption
Surface trap state formation	Metal ion binding introduces mid-gap electronic states; traps charge carriers	Fluorescence lifetime; intensity	Both decrease	Lifetime shortening (diagnostic feature)
Aggregation-induced quenching	Multivalent metal ions crosslink QDs; induces aggregation and inter-particle energy transfer	Intensity; scattering; aggregation state	Intensity decrease; increased scattering	DLS evidence of aggregation; reversible upon chelation
Direct charge injection	Metal ion binding perturbs local electronic environment; alters charge carrier dynamics	Radiative/non-radiative recombination ratio	Intensity decrease	Concentration-dependent quenching efficiency

### 4.2. Chelation-enhanced and turn-on fluorescence

In contrast to quenching-based sensors, chelation-enhanced or “turn-on” fluorescence systems produce an increase in photoluminescence intensity upon metal ion binding, offering distinct analytical advantages.^173–175^ These systems are engineered such that intrinsic QD emission is initially suppressed by surface defects, quenching moieties, or unfavourable charge carrier dynamics. Several design strategies enable turn-on fluorescence responses. Surface defect passivation occurs when metal ion coordination saturates dangling bonds or fills surface trap states, reducing non-radiative recombination pathways; for instance, Zn^2+^ binding to thiol-capped CdTe QDs passivates sulphur vacancy defects, restoring radiative emission.^144^ Displacement of quenching ligands represents another approach wherein pre-bound quenchers are displaced upon competitive metal ion coordination, liberating the QD from fluorescence suppression. Aggregation-induced emission enhancement has been observed in certain QD systems, particularly CDs with specific surface functionalities, which exhibit enhanced emission upon controlled aggregation triggered by metal ion crosslinking.^173,174^ Conformational switching occurs when metal ion binding induces structural rearrangements in surface-conjugated biomolecules such as aptamers or DNAzymes, modulating quencher proximity and restoring fluorescence.^176^ Turn-on sensing strategies offer practical advantages including improved signal-to-noise ratios, reduced susceptibility to false positives from non-specific quenching, and enhanced visual discrimination for qualitative applications.^174,175^ These approaches prove particularly effective when combined with selective chelating ligands such as thymine-rich oligonucleotides for Hg^2+^, G-quadruplex-forming sequences for Pb^2+^, or ion-imprinted polymers synthesised with target metal templates.^175,177^

### 4.3. FRET and ratiometric detection

FRET provides a powerful mechanism for ratiometric heavy metal sensing, enabling self-referencing measurements independent of absolute fluorescence intensity.^178^ FRET occurs through non-radiative dipole–dipole coupling between an excited donor fluorophore and a ground-state acceptor, with efficiency strongly dependent on the sixth power of donor–acceptor separation according to the relationship
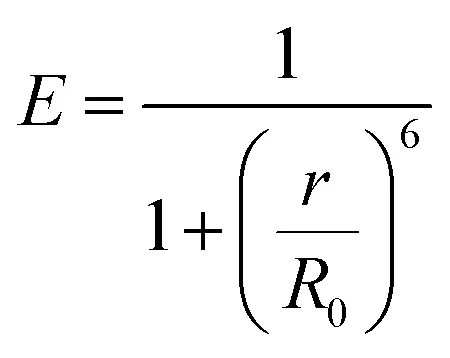
where *E* is transfer efficiency, *r* is the donor–acceptor distance, and *R*_0_ is the Förster radius, typically ranging from 3–8 nm for QD systems.^141^

In FRET-based architectures for heavy metal detection, QDs typically serve as energy donors owing to their high extinction coefficients, broad absorption spectra, and size-tunable emission wavelengths.^141^ Acceptors may include organic fluorophores such as rhodamine or fluorescein derivatives, secondary QDs of differing size, gold NPs enabling quenching through nanometal surface energy transfer, or metal complexes with appropriate spectral overlap. Metal ion binding modulates FRET efficiency through several mechanisms: distance modulation occurs when conformational changes in surface-bound aptamers or DNAzymes alter donor–acceptor separation upon target recognition; complex dissociation results when metal ion binding disrupts pre-assembled donor–acceptor pairs, eliminating energy transfer and restoring donor emission; and spectral overlap modification arises when metal coordination shifts acceptor absorption spectra, modulating the spectral overlap integral and FRET efficiency.^179–181^ Ratiometric detection, wherein the analytical signal derives from the ratio of two independent emission bands, typically an analyte-responsive signal and a stable reference signal, rather than absolute intensity at a single wavelength, offers inherent self-referencing capability that single-emission turn-on and turn-off sensing formats lack. In simple turn-on and turn-off sensors, the detection signal arising from intensity change at a single emission wavelength is vulnerable to analyte-independent confounding factors, including fluctuations in excitation source intensity, instrumental efficiency, local probe concentration, and sample matrix variation, any of which can be misattributed to a change in analyte concentration.^182^ Ratiometric measurement substantially improves measurement reliability by compensating for fluctuations in excitation source intensity, optical path length variations, probe photobleaching, and heterogeneous sample matrices, since both emission bands are subject to the same instrumental and environmental perturbations. Dual-emission CDs and lanthanide-doped NPs have emerged as particularly attractive platforms for ratiometric heavy metal sensing owing to their intrinsic multi-wavelength emission profiles.^183,184^

### 4.4. Surface ligand-mediated recognition

Surface functionalisation represents the decisive factor governing selectivity and sensitivity in QD-based heavy metal sensors.^185,186^ The native capping ligands employed during QD synthesis, such as trioctylphosphine oxide, oleic acid, or alkylthiols, typically require exchange or modification to impart aqueous dispersibility and analyte-specific recognition. Ligand design follows principles of coordination chemistry, particularly HSAB theory, to achieve selective metal ion binding.^187^Table 9 summarizes the principal ligand types employed in QD surface functionalisation, their metal ion affinities based on HSAB theory, and the coordination chemistry principles governing their selectivity. Small-molecule ligands bearing thiol, carboxylate, amine, or heteroatom-rich functional groups provide coordination sites with predictable metal affinities. Thiols and thioethers exhibit strong affinity for soft Lewis acids, including Hg^2+^, Ag^+^, Cu^+^, and Pb^2+^, whilst carboxylates and phosphonates preferentially bind hard Lewis acids such as Fe^3+^, Cr^3+^, and Al^3+^. Nitrogen-containing heterocycles including imidazole and pyridine coordinate borderline metals such as Cu^2+^, Zn^2+^, and Cd^2+^.^187^

**Table 9:** Surface ligand-mediated recognition

Ligand type	Metal ion affinity	Lewis acid class	Target metals	Coordination chemistry basis
Thiols/thioethers	Very strong	Soft Lewis acids	Hg^2+^, Ag^+^, Cu^+^, Pb^2+^	Soft base–soft acid interactions (HSAB theory); π-accepting capability
Carboxylates/phosphonates	Strong	Hard Lewis acids	Fe^3+^, Cr^3+^, Al^3+^, Ce^3+^	Hard base–hard acid interactions; electrostatic coordination
Nitrogen heterocycles	Moderate to strong	Borderline metals	Cu^2+^, Zn^2+^, Cd^2+^, Ni^2+^	Borderline base–borderline acid interactions; π-backbonding
Amine groups	Moderate	Borderline metals	Cu^2+^, Zn^2+^, Pb^2+^	Coordination *via* lone pairs; tunable through chemical modification
Biomolecular recognition	Ultra-high (nM–pM *K*_d_)	Variable (engineered)	Pb^2+^ (8–17 DNAzyme), UO_2_^2+^ (39E DNAzyme), Hg^2+^ (thymine aptamers)	Evolved through SELEX; metal-activated catalysis or conformational switching

Biomolecular recognition elements offer enhanced selectivity through precisely evolved or systematically selected binding motifs. Aptamers, comprising single-stranded DNA or RNA sequences identified through SELEX, exhibit dissociation constants in the nanomolar to picomolar range for specific metal ions. DNAzymes represent catalytic nucleic acids requiring specific metal cofactors, such as the Pb^2+^-dependent 8–17 DNAzyme or the UO_2_^2+^-dependent 39E DNAzyme, enabling highly selective detection through metal-activated strand cleavage. Peptides derived from phage display selection or metallothionein sequences provide tuneable metal-binding domains, whilst ion-imprinted polymers synthesised around metal ion templates retain complementary binding cavities following template removal, offering selectivity comparable to biological recognition elements with superior chemical stability.^181,188–190^

Binding of target ions to surface ligands perturbs the local electronic environment through several pathways including direct charge injection, modification of surface dipole layers, alteration of the dielectric environment, or induction of structural rearrangements. These perturbations modulate charge carrier dynamics, shifting the balance between radiative and non-radiative recombination pathways. Through rational ligand design informed by computational modelling and systematic screening, QD sensors achieve discrimination between chemically similar ions, such as Cd^2+^*versus* Zn^2+^ or As^3+^*versus* As^5+^, even in matrices containing 100–1000-fold excess of competing species.^191–193^

### 4.5. Electrochemical and photoelectrochemical transduction

Beyond optical sensing modalities, QDs have been extensively integrated into electrochemical and PEC detection platforms, offering complementary advantages including facile miniaturisation, low power requirements, and amenability to multiplexed array formats.^142,154,194^

Electrochemical sensors exploit the redox activity of QDs or utilise them as electrode modifiers to enhance electron transfer kinetics and electrocatalytic activity. QDs immobilised on electrode surfaces including glassy carbon, gold, or indium tin oxide provide high surface area scaffolds for subsequent functionalisation with recognition elements. Heavy metal ion binding modulates interfacial charge transfer through alteration of QD surface charge and electrical double layer structure, formation of metal–ligand complexes that block or facilitate electron tunnelling, displacement of electroactive species from the electrode surface, and modification of the QD electronic structure affecting redox potentials. Anodic stripping voltammetry combined with QD-modified electrodes achieves exceptionally low LODs through electrochemical preconcentration, with reported values reaching femtomolar concentrations for Pb^2+^ and Cd^2+^.^151–153^

Among electrode-modifying nanomaterials, GQDs have attracted particular attention for electrochemical heavy-metal sensing owing to their high electrical conductivity, large edge-plane defect density, and abundant oxygen-containing functional groups, which together enhance electron transfer kinetics and provide additional metal-ion coordination sites; a recent review addresses the mechanistic basis and comparative sensing performance of GQD-based electrochemical sensors specifically for Cd(ii), Pb(ii), and Hg(ii).^195^ PEC sensors combine optical excitation with electrochemical readout, leveraging QD light absorption to generate photocurrents that are modulated by analyte binding. Upon illumination, photoexcited electrons transfer to the electrode in photoanode configuration or to solution-phase electron acceptors, generating measurable currents proportional to the efficiency of charge separation and collection. Metal ion binding perturbs this process through electron or hole trapping at metal-occupied surface sites, modification of band edge positions affecting the driving force for charge transfer, alterations in electron–hole recombination kinetics, and steric blocking of electron acceptor access to the QD surface.^148–150^

The spatial separation of optical excitation and electrochemical detection in PEC systems provides inherently low background signals, as photocurrent generation requires both appropriate illumination and electrochemical polarisation. This characteristic enables LODs comparable to advanced spectroscopic techniques, reaching sub-nanomolar concentrations, whilst retaining operational simplicity and portability amenable to field deployment. Recent advances in PEC sensing have demonstrated multiplexed heavy metal detection through spatial patterning of QDs with distinct spectral responses, enabling simultaneous quantification of multiple analytes under wavelength-selective illumination.^146,147,196^

Table 10 provides a comprehensive comparison of quantitative performance parameters across all QD-based heavy metal detection methods discussed in this section.^197–201^ These metrics, including LODs, linear ranges, reproducibility, and recovery rates, are critical for method selection and optimization in specific analytical applications. The performance parameters summarized in Table 10 demonstrate that electrochemical methods achieve the lowest LODs (femtomolar to picomolar range), whilst FRET/ratiometric approaches offer superior reproducibility and matrix compensation through self-referencing capability. The selection of an appropriate detection method must balance sensitivity requirements, sample complexity, analysis time, and available instrumentation, as discussed in the following section. Whilst the performance parameters in Table 10 demonstrate the capabilities of QD-based sensors under optimized conditions, real-world implementation frequently encounters interferences that compromise analytical performance. Table 11 summarizes the principal interference mechanisms encountered in QD-based heavy metal detection and established mitigation strategies, providing practical guidance for method optimization and troubleshooting. The mitigation strategies outlined in Table 11 are essential for translating laboratory-optimized sensors into field-deployable systems capable of reliable performance in complex environmental, industrial, and biological matrices. Successful implementation often requires combining multiple strategies, such as pH buffering with selective ligand design, or sample pretreatment with ratiometric detection approaches.

**Table 10:** Quantitative performance parameters

Detection method	Linear range	LOD *vs.* LOQ	Reproducibility (RSD%)	Response time	Dynamic range (orders of magnitude)	Recovery rate (%)	Interference tolerance
Fluorescence quenching	1 nM–100 µM	LOD: 0.5–5 nM	2–8% (intra-batch)	30 s–5 min	3–5 orders	85–105% (simple matrices)	Moderate; 10–100× competing ions tolerated
		LOQ: 2–15 nM	5–15% (inter-batch)			75–95% (complex matrices)	
Turn-on fluorescence	5 nM–50 µM	LOD: 1–10 nM	3–10% (intra-batch)	1–10 min	3–4 orders	90–110% (with selective ligands)	High with aptamers; 100–1000× excess tolerated
		LOQ: 3–30 nM	8–18% (inter-batch)				
FRET/ratiometric	0.1 nM–10 µM	LOD: 0.05–1 nM	1–5% (intra-batch)	5–60 min	4–5 orders	92–108% (excellent matrix compensation)	Very high; ratiometric self-correction
		LOQ: 0.2–3 nM	3–8% (inter-batch)				
Electrochemical (ASV)	0.01 pM–1 µM	LOD: 0.001–0.1 pM	2–6% (intra-batch)	2–15 min (with preconcentration)	5–7 orders	88–112% (standard addition method)	High; selective at specific potentials; masking agents available
		LOQ: 0.003–0.3 pM	4–12% (inter-batch)				
PEC	0.1 nM–10 µM	LOD: 0.05–0.5 nM	2–7% (intra-batch)	1–10 min	4–5 orders	90–110%	Very high; low background enables discrimination
		LOQ: 0.15–1.5 nM	5–12% (inter-batch)				

**Table 11:** Interference effects and mitigation strategies

Interference type	Mechanism	Affected detection methods	Mitigation strategy	Implementation details
Competing metal ions	Similar coordination chemistry; competitive binding to surface ligands	All fluorescence-based methods	Selective ligand design *via* HSAB theory; aptamer/DNAzyme use; masking agents	Thiols for soft acids (Hg^2+^); carboxylates for hard acids (Fe^3+^); EDTA/citrate as masking agents for interferents
pH effects	Protonation/deprotonation of surface ligands; QD surface charge alteration; metal speciation changes	All methods; particularly fluorescence	pH buffering; pH-insensitive ligands; ratiometric detection	Phosphate buffers (pH 6–8); HEPES (pH 7–8); dual-emission QDs for pH-independent ratiometric sensing
Ionic strength	Screening of electrostatic interactions; QD aggregation; altered diffusion kinetics	Fluorescence quenching (dynamic); electrochemical	Ionic strength adjustment; use of neutral ligands; FRET-based detection	Dilution to constant ionic strength; polyethylene glycol (PEG) surface coating; internal standards
Organic matter (humic/fulvic acids)	IFE; competitive metal complexation; QD surface fouling	Fluorescence-based methods	Sample pretreatment; wavelength selection; turn-on sensors less affected	Activated carbon treatment; UV digestion; solid-phase extraction; excitation/emission wavelength optimization
Redox-active species (O_2_, H_2_O_2_)	Oxidation of QD surface; electron/hole scavenging; fluorescence quenching	Fluorescence and PEC	Anaerobic conditions; antioxidants; core–shell QDs with protective layers	N_2_ purging; ascorbic acid addition; ZnS shell passivation; storage under argon
Temperature variations	Altered diffusion kinetics; QY changes; thermally-activated non-radiative decay	Dynamic quenching; electrochemical	Temperature control; ratiometric detection; temperature compensation algorithms	Thermostatted cells; dual-emission QDs; calibration at multiple temperatures; Arrhenius correction
Background fluorescence	Sample autofluorescence; fluorescent contaminants	All fluorescence methods	Time-resolved fluorescence; NIR QDs; blank subtraction	Lanthanide-doped QDs (long lifetime); Ag_2_S QDs (NIR emission); matrix-matched blanks
Electrode fouling	Protein adsorption; organic film formation; passivation of active sites	Electrochemical and PEC	Antifouling coatings; electrode regeneration; pulsed potential cleaning	PEG or zwitterionic coatings; periodic electrochemical cleaning (−1.5 V, 30 s); disposable electrodes
Surfactants and detergents	Micelle formation; QD surface displacement; altered aggregation state	All methods	Sample dilution; liquid–liquid extraction; solid-phase extraction	1 : 10 or 1 : 100 dilution; chloroform extraction; C18 SPE cartridges

### 4.6. Advanced characterization techniques for probing sensing mechanisms

A recurring limitation across reports of QD-based heavy-metal sensors is that mechanistic claims (static or dynamic quenching, inner-filter effect, photoinduced electron transfer, surface-state mediation) are frequently supported only by steady-state photoluminescence (PL) titration. A decrease in steady-state emission intensity is consistent with several distinct mechanisms that carry different implications for sensor reversibility, selectivity, and long-term stability, and these cannot be distinguished without time-resolved and structurally resolved measurements. This section summarizes the diagnostic value of eight complementary techniques for interrogating QD heavy-metal sensing mechanisms.

#### 4.6.1. Time-resolved photoluminescence (TRPL) and time-correlated single-photon counting (TCSPC)

TRPL measures the excited-state lifetime of the QD emitter, which is the parameter that discriminates most directly between quenching pathways. Dynamic (collisional) quenching shortens the fluorescence lifetime in proportion to the intensity decrease, so that the Stern–Volmer constants obtained from *τ*_0_/*τ* and from *I*_0_/*I* coincide, whereas static quenching (ground-state complex formation) leaves the lifetime of the remaining emissive population essentially unchanged, since the emitting fraction is reduced in number rather than in decay rate. Reports of thiol-capped CdSe/ZnS QD sensors for Hg^2+^, Pb^2+^, and Cd^2+^ that rely solely on steady-state Stern–Volmer plots, such as 2-mercaptopropionic acid-capped CdSe/ZnS QDs, for which quenching efficiency followed the order Hg^2+^ > Cd^2+^ > Pb^2+^ but no lifetime data were collected, leave this mechanistic assignment unresolved.^202^ TCSPC studies of structurally related CdSe/ZnS systems illustrate the diagnostic power that this measurement adds, since surface-ligand chemistry alone can shift excited-state lifetimes by an order of magnitude (from approximately 4.5 ns for a d-penicillamine-capped probe to 11 to 27 ns for a d-penicillamine–histidine peptide-capped analogue), and the lifetime itself can be made sensitive to a chemical variable such as pH through ligand protonation state, giving a self-referencing, ratiometric-lifetime response of 2.0 to 2.4 ns per pH unit that is inherently immune to photobleaching and concentration drift.^203^ The same logic applies directly to metal-ion sensing, where a lifetime-based readout would decouple the sensor signal from QD concentration and photobleaching artifacts that limit amplitude-only assays. For heavy-metal QD sensors, TRPL and TCSPC can be used to confirm whether lifetime- and intensity-derived Stern–Volmer constants coincide, to resolve multi-exponential decay components arising from QD subpopulations with differing surface-trap involvement, and to quantify the trapped-carrier fraction, which has been shown to rise from under 10% to over 30% of the excited population as surface-trap density increases in CdSe QDs, directly modulating quenching sensitivity.^204^

#### 4.6.2. Femtosecond transient absorption spectroscopy (TAS)

Where TRPL reports only the radiative decay channel, pump-probe TAS resolves the complete excited-state kinetic manifold on the femtosecond-to-nanosecond timescale, including non-emissive trapping steps invisible to PL. The ground-state bleach (GSB) recovery of the first excitonic transition tracks the conduction-band electron population directly, while new transient features report on trap-state occupation, charge separation, or energy transfer to an adsorbed analyte. In CdSe QDs with deliberately varied surface-trap density, TAS resolved the excited electron's fate into three kinetic components: intraband relaxation on the picosecond timescale, trapping by surface states on the tens-to-hundreds-of-picosecond timescale, with the trapped fraction increasing with trap density, and radiative recombination on the nanosecond timescale. A higher trap-state density was shown to both accelerate trapping and suppress triplet-exciton energy transfer to a surface-anchored acceptor by roughly one to two orders of magnitude in rate constant.^204^ Applied to a heavy-metal QD sensor, TAS can establish mechanistically whether metal-ion binding opens a new non-radiative pathway competing with band-edge recombination, consistent with the electron-transfer or hole-trapping picture commonly invoked for Hg^2+^ and Cu^2+^ quenching, whether it intercepts a pre-existing surface-trap population, or whether it proceeds *via* genuine photoinduced electron transfer to the bound ion. Each of these pathways predicts a distinct GSB recovery signature and a distinct dependence of quenching efficiency on pre-existing trap density.

#### 4.6.3. X-ray photoelectron spectroscopy (XPS)

XPS provides direct, element-specific evidence of surface stoichiometry, oxidation state, and, critically for sensing mechanism assignment, surface-*versus*-bulk atomic distribution, by deconvoluting core-level spectra such as Cd 3d and Se 3d into surface and inner-lattice components. This distinction is mechanistically important because dangling-bond surface atoms are the structural origin of surface trap states. CdSe QDs with a high proportion of surface Se atoms (35%, compared with 2% for a more fully passivated analogue) show a correspondingly large, red-shifted, low-energy PL feature attributable to surface-trap-mediated emission, alongside an order-of-magnitude drop in absolute PL quantum yield.^204^ For heavy-metal sensing specifically, XPS binding-energy shifts recorded before and after analyte exposure can directly test whether a proposed quenching mechanism, such as Hg^2+^ displacing a surface ligand or forming a new M–S or M–Se surface bond, actually occurs at the surface rather than being inferred solely from optical data.

#### 4.6.4. X-ray absorption fine structure spectroscopy (XANES/EXAFS)

XAS techniques, comprising XANES and EXAFS, probe the local electronic and coordination environment around a selected element with a precision in interatomic distance of approximately 0.01 Å that neither XPS nor optical spectroscopy can match, and they remain element-specific even in multi-component systems.^205^ XANES pre-edge and edge-position shifts report oxidation state and local symmetry around the absorbing atom, while EXAFS oscillations yield coordination number, neighbor identity, and bond distance out to roughly 5 to 10 Å without requiring long-range crystalline order, making the technique applicable even to small, surface-disordered QDs. For a heavy-metal QD sensor, EXAFS at the metal K- or L-edge of the bound analyte (Hg, Pb, Cd, Cu, *etc.*) can directly resolve the coordination geometry of the sensing interaction, for example distinguishing a genuine M–S(thiolate) or M–N coordination bond at the QD surface from simple electrostatic or diffusion-limited collisional quenching, in the same way that EXAFS fitting is used to distinguish isolated single-metal-atom coordination (M–N, M–O) from metal–metal bonding in single-atom catalyst characterization.^205^ This capability is particularly relevant where quenching selectivity is attributed to a specific ligand–metal affinity, such as thiol affinity for Hg^2+^.

#### 4.6.5. Raman spectroscopy

Raman spectroscopy reports on phonon modes and surface-ligand vibrational signatures, offering a complementary, non-destructive probe of both the inorganic QD lattice and the organic capping layer. In heavy-metal cation sensing studies of 2-mercaptopropionic acid-capped CdSe/ZnS QDs, Raman spectroscopy was used alongside XRD and FTIR to confirm shell formation and surface ligand identity, establishing the baseline structural picture against which quenching behavior was interpreted.^202^ For sensing-mechanism studies specifically, Raman shifts or intensity changes in ligand-associated vibrational modes, such as thiolate C–S or carboxylate stretching modes, upon analyte exposure can provide direct vibrational evidence of ligand–metal coordination or displacement, complementing the electronic-structure information from XPS and XAFS.

#### 4.6.6. Transmission electron microscopy (TEM)

TEM provides direct imaging evidence of QD core size, size distribution, and, through high-resolution lattice imaging, core/shell architecture, all of which underpin the confinement-dependent optical properties exploited in sensing. TEM has been used to confirm nanoparticle diameter and size dispersity in QD pH-probe and heavy-metal sensor systems alike, and to verify that surface functionalization does not induce aggregation through changes in apparent particle size or size distribution.^202,203^ For heavy-metal sensing, TEM imaging conducted before and after prolonged analyte exposure, analogous to the aggregation-stability TEM and DLS checks used to compare ligand chemistries in pH-probe development, can directly test whether apparent quenching originates from genuine molecular-level sensing or from analyte-induced QD aggregation, a distinction that steady-state PL alone cannot make.

#### 4.6.7. Atomic force microscopy (AFM)

AFM complements TEM by providing quantitative topographic and mechanical information, including particle height, surface roughness, and, in more specialized configurations such as conductive or Kelvin-probe AFM, local surface potential, under conditions closer to the QD's native solution or film environment than the high-vacuum conditions required for TEM. For QD sensors deployed as immobilized films or on solid substrates, such as paper-based or electrode-supported sensing platforms, AFM can resolve film morphology, QD packing density, and substrate-induced heterogeneity that influence sensor response uniformity and reproducibility, parameters not accessible from solution-phase optical or TEM characterization alone.^206–208^

#### 4.6.8. *Operando* fluorescence imaging

*Operando*, or *in situ*, fluorescence imaging extends time-resolved and steady-state PL measurements from bulk cuvette conditions to spatially resolved, real-time observation of the sensing event as it occurs, for example through fluorescence lifetime imaging microscopy (FLIM) mapping the spatial distribution of QD lifetime, and by extension local analyte concentration, across a heterogeneous sample. FLIM has been used to map intracellular pH with pixel-level spatial resolution by converting a fluorescence-lifetime calibration curve into a pseudocolor pH map across an 80 × 80 µm imaging field, demonstrating that lifetime-based sensing can be read out with full spatial context rather than as a single bulk-averaged value.^202,203^ Applied to heavy-metal sensing in environmental or biological samples with spatially heterogeneous analyte distribution, such as localized contamination in a water sample matrix or metal accumulation at specific sites in a biological system, *operando* fluorescence or FLIM imaging would allow the sensing response to be resolved spatially and monitored continuously during exposure, rather than as a single time-point measurement.

Taken together, these eight techniques give access to time-resolved, structurally resolved, and spatially resolved evidence for the mechanistic claims (static *versus* dynamic quenching, inner-filter effect, surface-trap mediation, electron transfer) that underlie QD heavy-metal sensor design, complementing the steady-state PL titration data that dominate current reports.

### 4.7. Practical deployment considerations

Whilst the performance parameters discussed in the preceding sections demonstrate the capabilities of QD-based sensors under optimized laboratory conditions, successful translation from bench to field deployment requires addressing numerous practical considerations that profoundly influence operational reliability, cost-effectiveness, and user acceptance.^209^ The gap between laboratory demonstration and real-world implementation represents a critical challenge in analytical sensor development, with many promising technologies failing to achieve widespread adoption due to inadequate attention to deployment constraints.^210^ Real-world analytical scenarios impose requirements that extend beyond fundamental detection metrics such as sensitivity and selectivity. Field deployment demands consideration of sample matrix complexity, calibration protocols, sensor stability and regeneration, operator skill requirements, regulatory compliance, and total cost of ownership. These factors collectively determine whether a QD-based sensing platform can deliver reliable analytical information in diverse operational contexts ranging from centralized environmental monitoring laboratories to remote field sites with limited infrastructure.^211^Table 12 summarizes the principal practical deployment considerations for QD-based heavy metal sensors, encompassing challenges, technical requirements, established best practices, and implementation guidance derived from field validation studies and operational experience.

**Table 12:** Practical deployment considerations

Consideration	Challenge	Requirements	Solution/best practice	Implementation notes
Sample preparation	Complex matrices (particulates, organics, high ionic strength)	Filtration, pH adjustment, dilution, or extraction	0.45 µm filtration; pH buffering to 7–8; 1 : 10 dilution for high salinity; SPE for organics	Minimal preparation preferred for field deployment; validate recovery rates
Calibration	Matrix effects; drift over time; non-linear responses	Matrix-matched standards; frequent recalibration; multi-point curves	Standard addition method; certified reference materials; 5–7 point calibration; daily or per-batch recalibration	Ratiometric methods reduce calibration frequency; internal standards improve accuracy
Sensor regeneration	Irreversible binding; surface fouling; signal degradation	Reversible binding chemistry; cleaning protocols; disposable designs	EDTA washing for reversible systems; electrochemical cleaning; single-use disposable sensors for low-cost applications	Aptamer/DNAzyme sensors often reusable 10–50× with regeneration; fluorescence sensors may require replacement after 20–100 uses
Storage stability	QD oxidation; aggregation; ligand degradation; loss of activity	Proper storage conditions; stabilizers; protective packaging	4 °C storage in dark; inert atmosphere (N_2_/Ar); lyophilization for long-term storage; antioxidants (ascorbate, β-mercaptoethanol)	Shelf life: 3–12 months (solution); 12–24 months (lyophilized); 24+ months (solid-state sensors)
Portability	Size, weight, power requirements of instrumentation	Miniaturized components; battery operation; rugged construction	LED excitation sources; portable spectrometers or smartphone-based detection; microfluidic integration; rechargeable Li-ion batteries	Electrochemical sensors most portable; smartphone-based fluorescence detection emerging; field-ready kits available
Regulatory compliance	Method validation; quality control; traceability	Standard operating procedures; QC samples; documentation	ISO 17025 accreditation protocols; EPA method validation guidelines; regular QC checks (blanks, spikes, duplicates); chain of custody	Required for regulatory monitoring; less stringent for screening applications; certified reference materials essential
Cost per analysis	Reagent costs; consumables; labor; instrument maintenance	Cost-effectiveness analysis; operational budget	Bulk synthesis of QDs; reusable sensors; automated analysis; minimal sample preparation	Substantially lower per-test cost than ICP-MS for both fluorescence and electrochemical QD detection, reflecting reduced instrumentation and reagent requirements
Operator training	Technical expertise required; protocol complexity	Training programs; user-friendly protocols; technical support	Simplified protocols for field use; pictorial guides; smartphone apps with decision support; remote technical assistance	Minimal training (1–2 hours) for turn-on fluorescence; moderate training (1 day) for electrochemical; advanced training for FRET systems

#### 4.7.1. Sample preparation and matrix effects

Sample preparation represents a critical determinant of analytical reliability, particularly when transitioning from controlled laboratory standards to complex environmental, industrial, or biological matrices. Real samples frequently contain particulate matter, dissolved organic substances, microbial content, and variable ionic strength that can interfere with QD performance through mechanisms including surface fouling, competitive binding, and optical interference.^212^ Whilst extensive sample preparation enhances reliability, it simultaneously increases analysis time, cost, and operator burden, potentially negating the advantages of QD sensors for rapid field deployment.^213^ Optimal sample preparation protocols balance analytical performance with operational simplicity. For aqueous environmental samples, filtration through 0.45 µm membrane filters removes particulates whilst preserving dissolved metal species, and pH adjustment to 7–8 using phosphate or HEPES buffers ensures consistent ionization states and QD surface chemistry.^13^ High-salinity samples, such as seawater or industrial effluents, may require dilution (typically 1 : 10 to 1 : 100) to reduce ionic strength effects on QD colloidal stability and fluorescence characteristics.^214^ Samples containing high concentrations of dissolved organic matter, such as river water with humic substances, benefit from solid-phase extraction or UV digestion to minimize IFEs and competitive metal complexation.

#### 4.7.2. Calibration strategies and quality control

Calibration protocols must account for matrix effects that alter sensor response characteristics between pure standards and real samples.^215^ Matrix-matched calibration, wherein standards are prepared in sample matrices of similar composition, compensates for these effects but requires extensive preliminary characterization and may not be feasible for highly variable sample types.^216^ The standard addition method, involving sequential addition of known analyte concentrations to the sample itself, inherently accounts for matrix effects and represents best practice for quantitative analysis in complex matrices. Ratiometric detection approaches, particularly those based on FRET or dual-emission QDs, exhibit reduced sensitivity to calibration drift and matrix effects due to their self-referencing nature.^217^ These systems maintain accuracy over extended deployment periods with less frequent recalibration, making them particularly attractive for continuous monitoring applications or field deployment in locations with limited access to calibration standards. Nevertheless, periodic validation using certified reference materials remains essential to ensure measurement traceability and compliance with quality assurance protocols.

#### 4.7.3. Sensor regeneration and operational lifetime

The ability to regenerate and reuse QD sensors substantially influences operational economics and environmental sustainability.^177^ Reversible sensing mechanisms, such as those employing weak metal–ligand interactions or conformational switching of biomolecular recognition elements, enable sensor regeneration through washing with chelating agents (ethylenediamine tetraacetic acid (EDTA), diethylenetriaminepentaacetic acid (DTPA)) or pH adjustment. Aptamer-based and DNAzyme-based sensors frequently demonstrate reusability through 10–50 measurement cycles with minimal signal degradation, particularly when regeneration protocols include gentle washing to remove non-specifically adsorbed species.^218^ Electrochemical QD sensors benefit from electrochemical regeneration protocols, wherein application of appropriate potentials desorbs bound metal ions and renews the sensing interface. This approach enables 50–200 measurement cycles on a single sensor, though gradual degradation of surface functionalization eventually necessitates replacement. For applications requiring ultra-low LODs and maximum reliability, disposable single-use sensors represent a viable alternative, particularly when fabricated using low-cost materials such as screen-printed electrodes modified with CDs.

#### 4.7.4. Storage stability and shelf life

Long-term stability of QD sensors during storage critically influences commercial viability and deployment feasibility, particularly for applications in remote locations or emergency response scenarios.^219^ QDs in solution phase exhibit limited stability due to oxidation of surface ligands, aggregation, and photodegradation, typically maintaining performance for 3–12 months when stored at 4 °C under inert atmosphere in amber vials. Lyophilization substantially extends shelf life to 12–24 months by eliminating water-mediated degradation pathways, with reconstitution in buffer restoring original performance characteristics.^220^ Solid-state sensor configurations, wherein QDs are immobilized in polymer matrices, sol–gel films, or paper substrates, demonstrate superior storage stability exceeding 24 months at ambient conditions. Core–shell QD architectures with inorganic passivating shells (*e.g.*, CdSe/ZnS, InP/ZnS) exhibit enhanced photochemical stability compared to organic-capped QDs, resisting photooxidation during extended storage and operational use. Inclusion of antioxidants such as ascorbic acid or β-mercaptoethanol in storage buffers further extends shelf life by scavenging reactive oxygen species.^221^

#### 4.7.5. Portability and field deployment

Miniaturization of detection instrumentation represents a key enabler for field deployment of QD sensors.^222^ Fluorescence-based approaches benefit from replacement of laboratory spectrofluorometers with compact LED excitation sources and portable spectrometers or photodiode detectors, reducing instrumentation to handheld or smartphone-integrated formats weighing less than 500 g and operating on rechargeable batteries. Smartphone-based detection platforms leverage integrated cameras, processing capability, and wireless connectivity to provide distributed sensing networks with real-time data transmission and cloud-based analysis.^20^ Electrochemical QD sensors offer inherent advantages for miniaturization due to simple instrumental requirements comprising a potentiostat, reference electrode, and working electrode modified with QDs. Commercial portable potentiostats with dimensions comparable to smartphones enable field deployment with analysis times of 5–15 minutes including electrochemical preconcentration.^223^ Integration of QD sensors with microfluidic sample handling automates sample introduction, dilution, and mixing, reducing operator burden and improving reproducibility in field settings.^224^

#### 4.7.6. Regulatory compliance and method validation

Adoption of QD-based sensors for regulatory monitoring of heavy metals in drinking water, wastewater discharge, or occupational exposure assessment requires demonstration of equivalency to established reference methods and validation according to standardized protocols. ISO 17025 accreditation mandates documentation of method performance characteristics including selectivity, linear range, LODs, accuracy (through certified reference materials), precision (intra-day and inter-day reproducibility), and robustness to matrix variations.^225^ EPA method validation guidelines require demonstration of method applicability across representative sample matrices and concentration ranges relevant to regulatory thresholds.^226^ Quality control protocols for routine operation include analysis of method blanks, fortified blanks (spiked at 2–5× the LOD), duplicate samples, and certified reference materials at frequencies determined by sample throughput and regulatory requirements. Control charts tracking quality control sample results enable detection of systematic drift or bias requiring corrective action such as recalibration or instrument maintenance. Chain of custody documentation, particularly for forensic or compliance applications, ensures sample traceability from collection through analysis and reporting.

#### 4.7.7. Cost-effectiveness and total cost of ownership

Economic considerations frequently determine the feasibility of deploying QD sensors *versus* traditional analytical techniques such as ICP-MS or AAS. Per-sample analysis costs for QD-based fluorescence and electrochemical detection are substantially lower than those of ICP-MS, reflecting reduced instrumentation and reagent requirements.^227^ However, total cost of ownership must account for initial capital investment, consumables, maintenance, operator training, and quality control, which may offset per-sample savings depending on throughput and operational lifetime.^228^ High-throughput applications, such as screening of large numbers of environmental or food samples, maximize cost-effectiveness by amortizing capital costs across numerous analyses. Conversely, low-throughput applications or those requiring high capital investment in instrumentation may not achieve favorable economics compared to outsourcing analysis to centralized laboratories equipped with traditional techniques.^229^ Lifecycle cost analysis should incorporate disposal costs for QDs, particularly those containing cadmium or other restricted substances, which may require treatment as hazardous waste depending on regulatory jurisdiction.

#### 4.7.8. Operator training and skill requirements

The technical expertise required for reliable sensor operation influences adoption rates and operational success, particularly in resource-limited settings or field deployment scenarios. Turn-on fluorescence sensors employing visual color change detection represent the simplest operational mode, requiring minimal training (1–2 hours) to perform qualitative or semi-quantitative assessment suitable for rapid screening applications.^230^ Fluorescence quenching methods using portable fluorometers require moderate training (4–8 hours) covering sample preparation, instrument operation, calibration procedures, and data interpretation. Electrochemical methods demand more extensive training (8–16 hours) due to the need for electrode preparation, electrochemical cleaning protocols, proper referencing, and voltametric data analysis.^231^ FRET-based ratiometric approaches, whilst offering superior analytical performance, require advanced training (16–24 hours) covering spectral analysis, ratio calculation, and troubleshooting of complex optical systems.^232^ Development of simplified protocols with pictorial guides, smartphone applications providing step-by-step instructions and automated data analysis, and remote technical support *via* telecommunications infrastructure can substantially reduce training requirements and improve operational reliability.

Field deployment is a central theme of this review, yet it is essential to distinguish clearly between three tiers of validation reported in the QD heavy-metal sensing literature: proof-of-concept demonstrations, in which sensing performance is established in simple buffer or ultrapure water systems under controlled laboratory conditions; real-sample validation, in which sensors are applied to authentic environmental, food, or biological matrices, typically *via* spiked-recovery experiments performed in a laboratory setting; and genuine field testing, in which portable instrumentation, on-site sample handling, and operation under uncontrolled environmental conditions (variable temperature, humidity, and lighting) are demonstrated. The majority of QD-based sensors reviewed in this manuscript, and in the QD heavy-metal sensing literature more broadly, have progressed only as far as real-sample validation; genuinely field-tested systems, evaluated with portable instrumentation outside laboratory conditions, remain comparatively rare.^55^ This distinction is revisited throughout the metal-specific sections of this review, and the practical requirements for advancing sensors from real-sample validation toward true field deployment, including portable instrumentation, calibration protocols, sample pre-treatment, storage stability, and operation under variable environmental conditions, are summarized in Table 12.

The practical considerations outlined in Table 12 and discussed in this section demonstrate that successful deployment of QD-based heavy metal sensors requires holistic attention to operational factors extending well beyond fundamental sensing mechanisms and performance metrics. Integration of these practical considerations during sensor development, rather than as an afterthought following laboratory optimization, maximizes the probability of successful field implementation and end-user acceptance. To provide broader analytical context, Table 13 benchmarks QD-based sensors against established heavy metal detection techniques including ICP-MS, AAS, colorimetric assays, ion-selective electrodes, and traditional voltammetry. This comparative evaluation demonstrates that whilst traditional methods retain specific advantages, QD sensors occupy a unique niche combining field portability, rapid analysis, and cost-effectiveness unattainable with laboratory-confined techniques.

**Table 13:** Comparison with traditional detection methods

Method	LOD	Analysis time	Portability	Sample preparation	Expertise required	Multiplexing
QD fluorescence^129^	0.5–10 nM	1–10 min	High	Minimal (filtration, pH adjust)	Low to moderate	Moderate
QD electrochemical^233^	0.001–1 pM	5–20 min	Very high	Minimal	Moderate	High (arrays)
ICP-MS^234^	0.001–0.1 pM	5–15 min per sample	None (benchtop)	Extensive (digestion, dilution)	High (specialist)	High (sequential)
AAS (atomic absorption)^235^	0.1–10 nM	3–10 min per element	None (benchtop)	Extensive (digestion)	High	Low (sequential)
ICP-OES^236^	∼5–500 nM (element-dependent; ppb-range)	1–5 min per sample (simultaneous multi-element)	None (benchtop)	Extensive (digestion, dilution)	High (specialist)	High (simultaneous, echelle detectors)
Colorimetric (traditional)^237^	10–1000 nM	10–30 min	High (test strips)	Moderate	Low	Low
Ion-selective electrodes^238^	1–1000 nM	5–15 min	High	Minimal (ionic strength adjust)	Low to moderate	Low (one electrode per ion)
Voltammetry (traditional)^239^	0.1–10 nM	10–30 min	Moderate	Moderate (deaeration, pH)	Moderate to high	Moderate (sequential stripping)

## 5. QDs for specific heavy metal detection

QD-based fluorescence sensors have undergone rapid development owing to their unique size-dependent photoluminescence, high brightness, and excellent photostability, which collectively enable highly sensitive detection of analytes at trace concentrations.^94,129,240^ Beyond their intrinsic optical properties, the analytical performance of QD-based sensors has been substantially enhanced through surface functionalisation and hybridisation with small molecules, polymers, nanomaterials, and biomolecular recognition elements, allowing the rational tailoring of QD-analyte interactions for improved sensitivity and selectivity.^241^ Despite these advances, achieving reliable and selective detection of individual heavy metal ions remains a non-trivial challenge. Many metal ions exhibit similar coordination behaviour and induce comparable fluorescence quenching or enhancement responses, which complicates metal-specific discrimination, particularly in complex environmental and biological matrices.^43,242^ Additional limitations related to the inherent toxicity of certain heavy-metal-based QDs, cross-reactivity, limited multiplexing capability, and restricted portability further constrain practical implementation.^43^ Consequently, the design of QD-based sensors for specific heavy metal detection requires careful integration of photophysical transduction mechanisms with chemically selective recognition strategies rather than reliance on fluorescence response alone. This section critically reviews the application of QDs in the detection of individual heavy metal ions and pesticide residues, with emphasis on metal-specific sensing mechanisms, surface functionalisation strategies, and comparative analytical performance across different QD systems. By systematically analysing diverse sensing methodologies, this section highlights both the significant progress achieved and the key challenges that must be addressed to enable selective, multiplexed, and application-ready QD-based sensing platforms.

### 5.1. Detection of lead (Pb)

Lead (Pb^2+^) contamination remains one of the most critical environmental and public health challenges due to its high toxicity, persistence, and cumulative biological effects. Pb^2+^ exposure is particularly detrimental to neurological development, with elevated blood lead levels (≥0.48 µmol L^−1^) associated with cognitive impairment, gastrointestinal disorders, renal dysfunction, and long-term cardiovascular risks. Owing to its non-biodegradable nature and widespread presence in industrial effluents, drinking water distribution systems, and contaminated soils, sensitive and selective detection of Pb^2+^ at trace levels is essential for effective risk assessment and regulatory compliance.^243,244^

QD-based fluorescence sensors have emerged as powerful analytical tools for Pb^2+^ detection, offering distinct advantages such as low LODs, rapid response, and compatibility with portable and visual sensing formats. However, selective discrimination of Pb^2+^ from chemically similar metal ions remains non-trivial, as Pb^2+^ often induces fluorescence quenching mechanisms analogous to those triggered by Hg^2+^, Cd^2+^, and Cu^2+^. Consequently, ratiometric fluorescence strategies that incorporate internal signal referencing have gained increasing attention due to their ability to mitigate environmental fluctuations, probe instability, and matrix-dependent signal variations.^143^ Wang *et al.* developed a ratiometric fluorescence sensor for visual, on-site Pb^2+^ detection using a blue-emitting Fe-MIL-88 metal–organic framework (B-MOF) integrated with tangerine-emitting Mn:ZnS QDs (T-QDs) as a dual-emission B-MOF/T-QDs composite. Pb^2+^ selectively quenched the QD-derived orange emission while the MOF's blue emission remained stable, enabling ratiometric, visually discriminable output with a LOD of 3.79 nM and reported selectivity over competing metal ions.^245^

Xue *et al.* developed low-toxicity, GSH-stabilized AgInS_2_ (AIS) QDs *via* a one-pot aqueous route for Pb^2+^ detection, achieving a LOD of 4 nM (below WHO/EPA thresholds) across a tunable linear range of 9–530 nM.^246^ Quenching proceeds *via* light-induced electron transfer, with Pb^2+^ reduction to Pb^0^ confirmed by XPS (Fig. 5). Selectivity was strong (∼81% quenching) against K^+^, Na^+^, Ba^2+^, Ca^2+^, Mg^2+^, and Zn^2+^, with Mn^2+^/Co^2+^ interference addressed *via* NaHCO_3_-mediated precipitation. The probe maintained stable photoluminescence over 60 days and achieved 92.2–102.6% recovery in real water samples. Two practical limitations were noted: synthesis and storage require an oxygen-free environment to prevent superoxide-induced baseline quenching, and the response requires 110 minutes to reach equilibrium; long relative to other entries in this section, and a consideration for high-throughput or rapid field deployment.

**Fig. 5:**
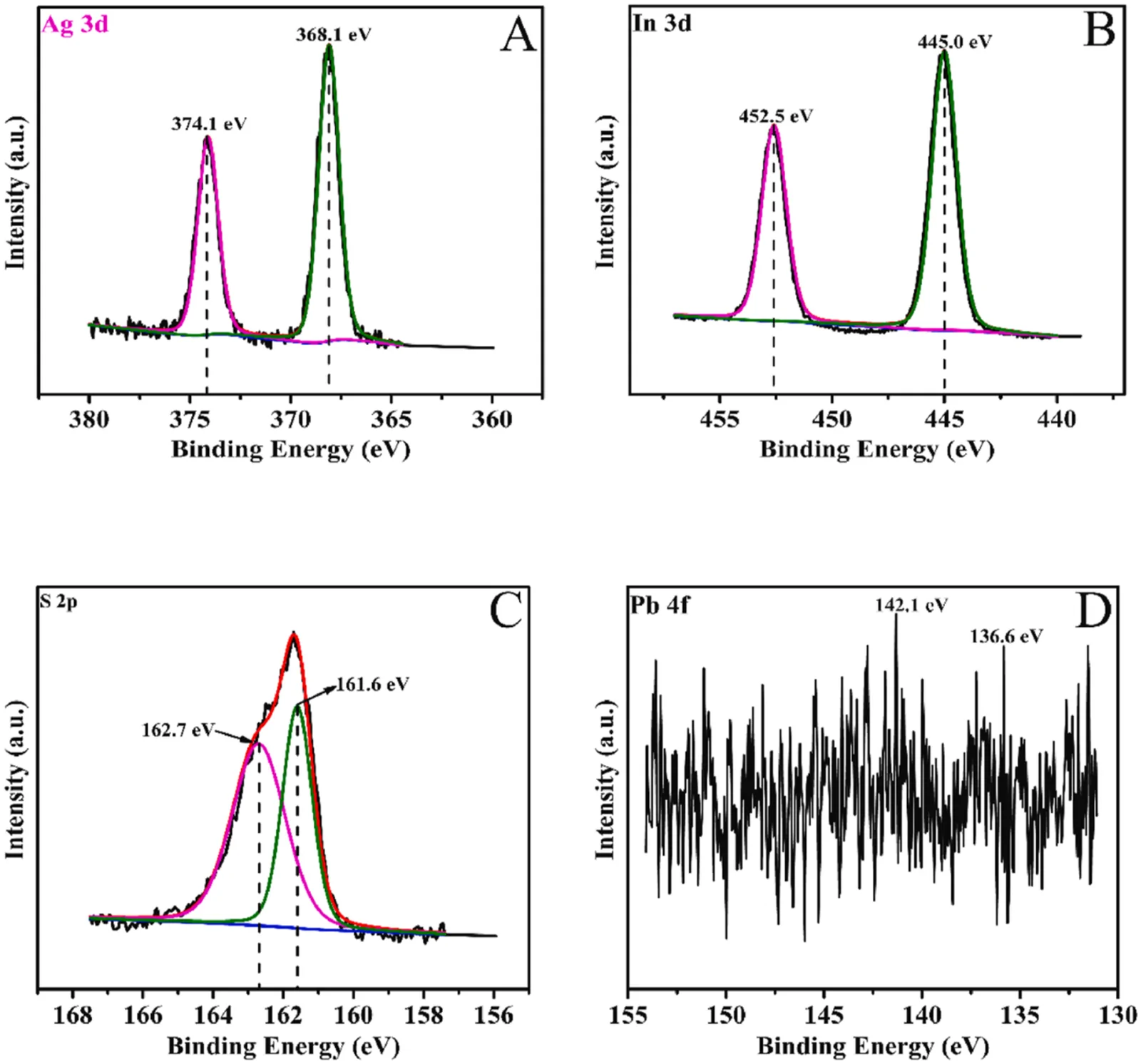
High-resolution XPS spectra corresponding to the Ag 3d (A), In 3d (B), S 2p (C), and Pb 4f (D) core levels of Pb^2+^-loaded AIS QDs.^246^

Li *et al.* developed a ratiometric fluorescence nanoprobe using dual-color CdTe QDs on a DNA tetrahedron (TDN)-functionalized silica scaffold for Pb^2+^ detection:^247^ silica-encapsulated red QDs served as a stable internal reference, while green QDs, tethered *via* aptamer-based framework nucleic acid, acted as signal reporters. Pb^2+^ binding triggers G-quadruplex formation, releasing the green QD-DNA reporters to a GO interface where PET/static quenching occurs (Fig. 6), giving ratiometric, self-calibrating output. The platform achieved a LOD of 1.7 nM (0.43 ppb) across a 10 nM–2 µM linear range; sensitivity comparable to ICP-MS; with high aptamer-conferred selectivity against coexisting ions and stable photoluminescence for over 30 days. Recovery in complex matrices (soil, milk, drugs, cosmetics, serum) ranged 95.3–108%. The authors noted that DNA grafting density on the silica surface is critical, as excessive density induces steric hindrance limiting target accessibility.

**Fig. 6:**
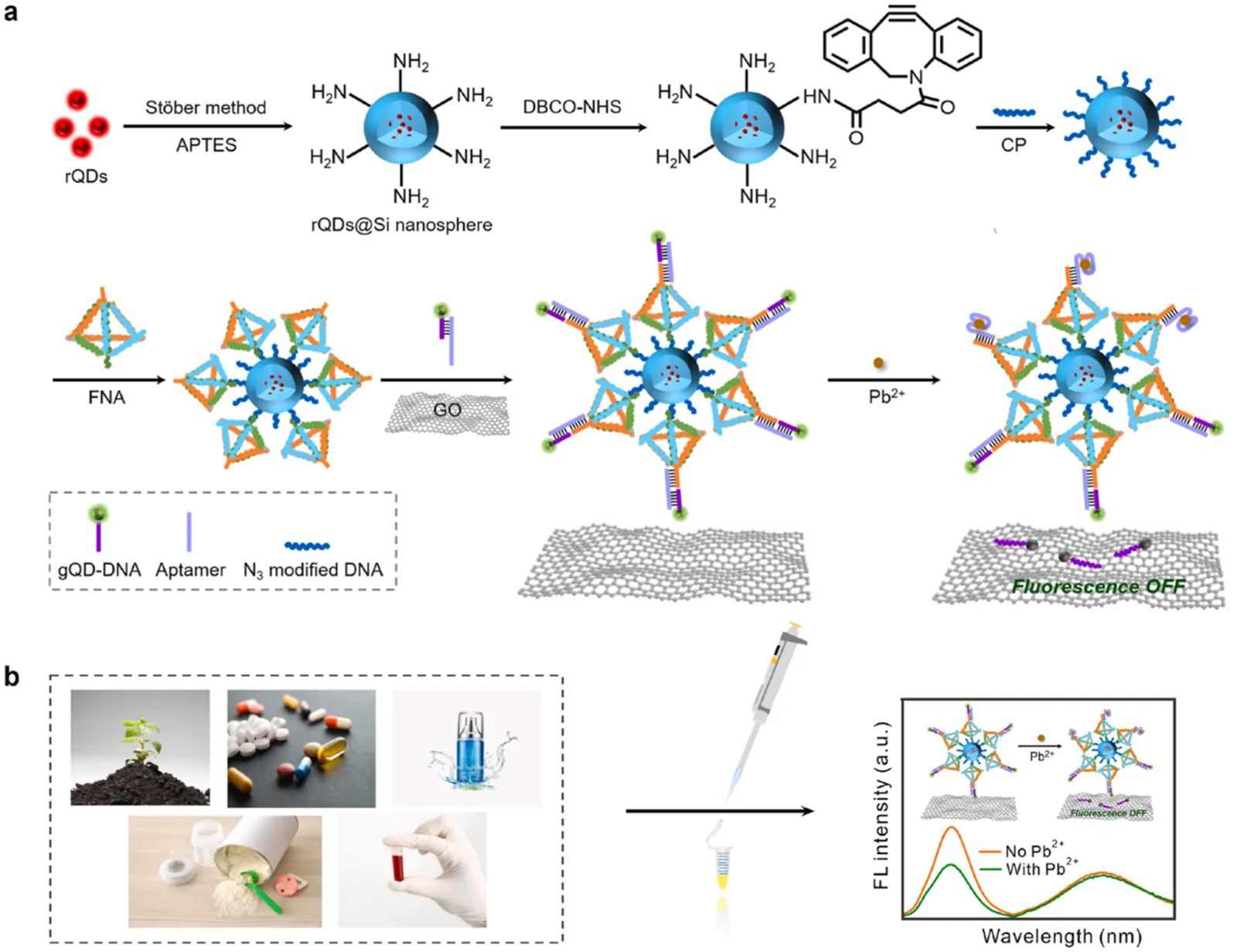
Schematic representation of the FNA-based ratiometric fluorescence assay for Pb^2+^ detection. (a) Detailed assembly of the rQDs@SiO_2_-CP/TDN nanoprobe, highlighting the integration of red-emitting internal references and green-emitting signal reporters. The mechanism involves the Pb^2+^-induced disassembly of the DNA sandwich, followed by the specific quenching of liberated gQD-DNA at the GO interface *via* electron transfer. (b) Broad applicability of the sensor for real-sample monitoring in environmental (soil), food (milk), pharmaceutical, and clinical (serum) matrices, illustrating its potential for versatile heavy metal testing.^247^

Zhang *et al.* developed dual-emitting Mn:ZnS QDs for ratiometric Pb^2+^ detection,^248^ with a ZnS-defect blue emission (450 nm) and Mn^2+^-dopant orange emission (585 nm). Sensing relies on aggregation-induced quenching: Pb^2+^'s lower *K*_sp_ relative to Zn^2+^/Mn^2+^ drives preferential adsorption onto the QD surface, inducing aggregation and a proportional F450/F585 ratio decrease, giving a self-calibrating signal independent of probe concentration or instrumental drift (Fig. 7). The platform achieved a LOD of 0.5 nM across a 10–60 nM linear range, with a visually distinguishable UV color transition. Interference from Cu^2+^, Hg^2+^, and Cd^2+^ (which also form insoluble sulfides) was addressed using 2,3-dimercapto-1-propanol as a masking agent. Recovery in apple and environmental water samples ranged 84.0–100.3%. The authors noted that unknown interferences in complex real samples remain a challenge for universal field deployment.

**Fig. 7:**
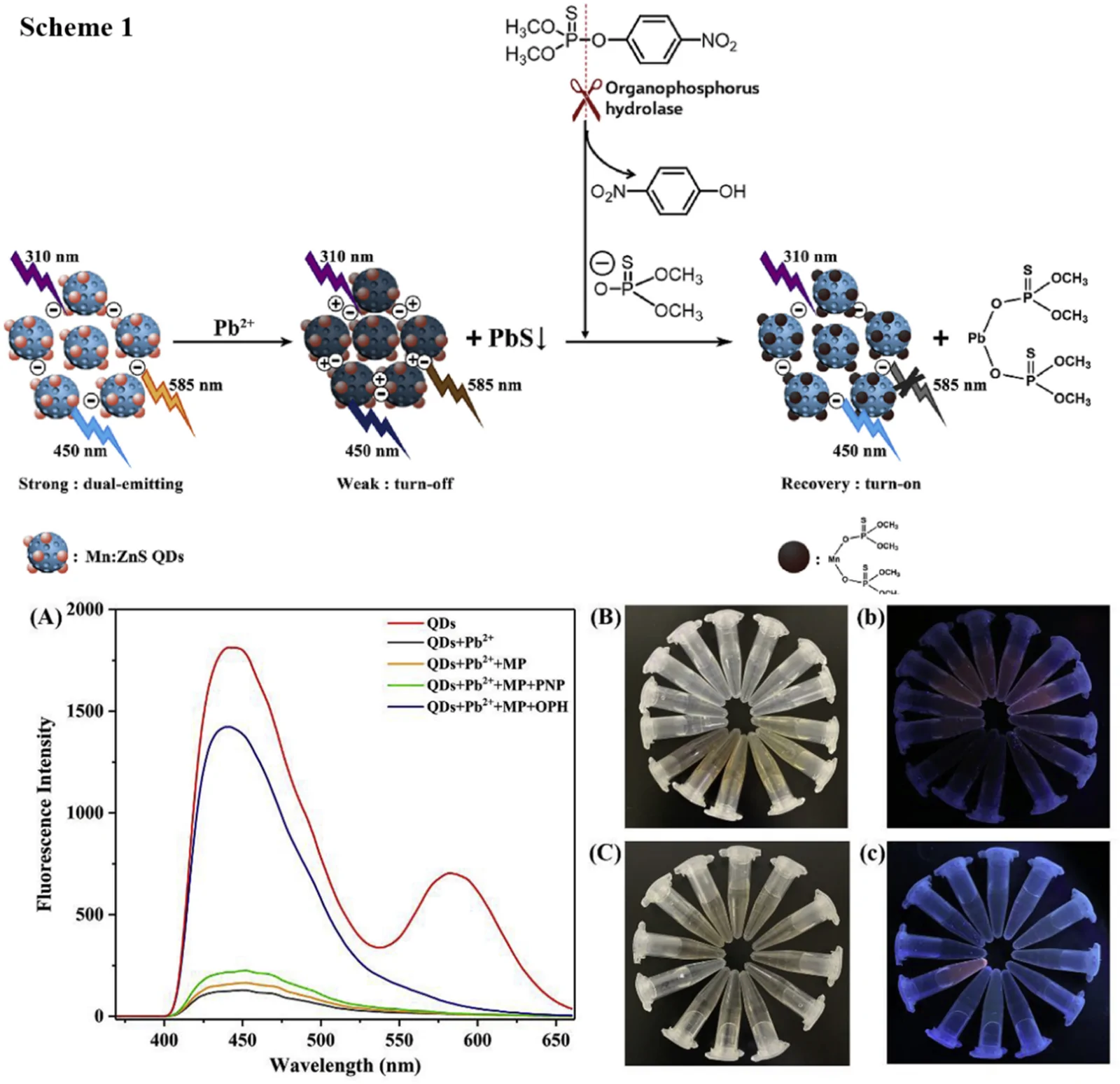
(Scheme 1) Schematic representation of the dual-emitting Mn:ZnS QD platform for Pb^2+^ monitoring and subsequent organophosphorus pesticide sensing. Addition of Pb^2+^ triggers QD aggregation through preferential adsorption, quenching both the 450 nm and 585 nm emissions and producing a ratiometric “turn-off” response. OPH-mediated hydrolysis of methyl parathion generates dimethyl thiophosphate, which chelates Pb^2+^ and restores the 450 nm emission (“turn-on”). (A) Evolution of the dual-emission spectra upon interaction with Pb^2+^ and subsequent reagents (MP, PNP, OPH). (B and b) Visual transformation of the QD-Pb^2+^ probe under white light and 254 nm UV light, showing concentration-dependent quenching and a distinct color change suitable for on-site visual screening. (C and c) Corresponding images after MP/OPH treatment, showing concentration-dependent fluorescence recovery.^248^

Jayalakshmi *et al.* developed cadmium-free, NAC-stabilized ZnSe QDs (*λ*_em_ = 465 nm, one-pot reflux synthesis) for rapid Pb^2+^ detection *via* static quenching (*K*_SV_ = 11.37 × 10^5^ M^−1^), achieving a LOD of 0.37 µM within a 0–10 µM linear range (meeting WHO drinking-water thresholds).^249^ Selectivity relies on kinetic differentiation from Hg^2+^:Pb^2+^ quenching completes in <90 s *versus* ∼5 min for Hg^2+^ (Fig. 8), enabling rapid discrimination *via* UV-visualized “turn-off” response. The probe remained stable across pH 3–11 and achieved 89–112% recovery across environmental water (tap, pond, sewage), fruit juices (lemon, tomato, sugarcane), and biomimetic fluids (artificial saliva, cerebrospinal fluid). A noted limitation is the need for precise reaction-time monitoring to avoid misattributing slower Hg^2+^ quenching to Pb^2+^.

**Fig. 8:**
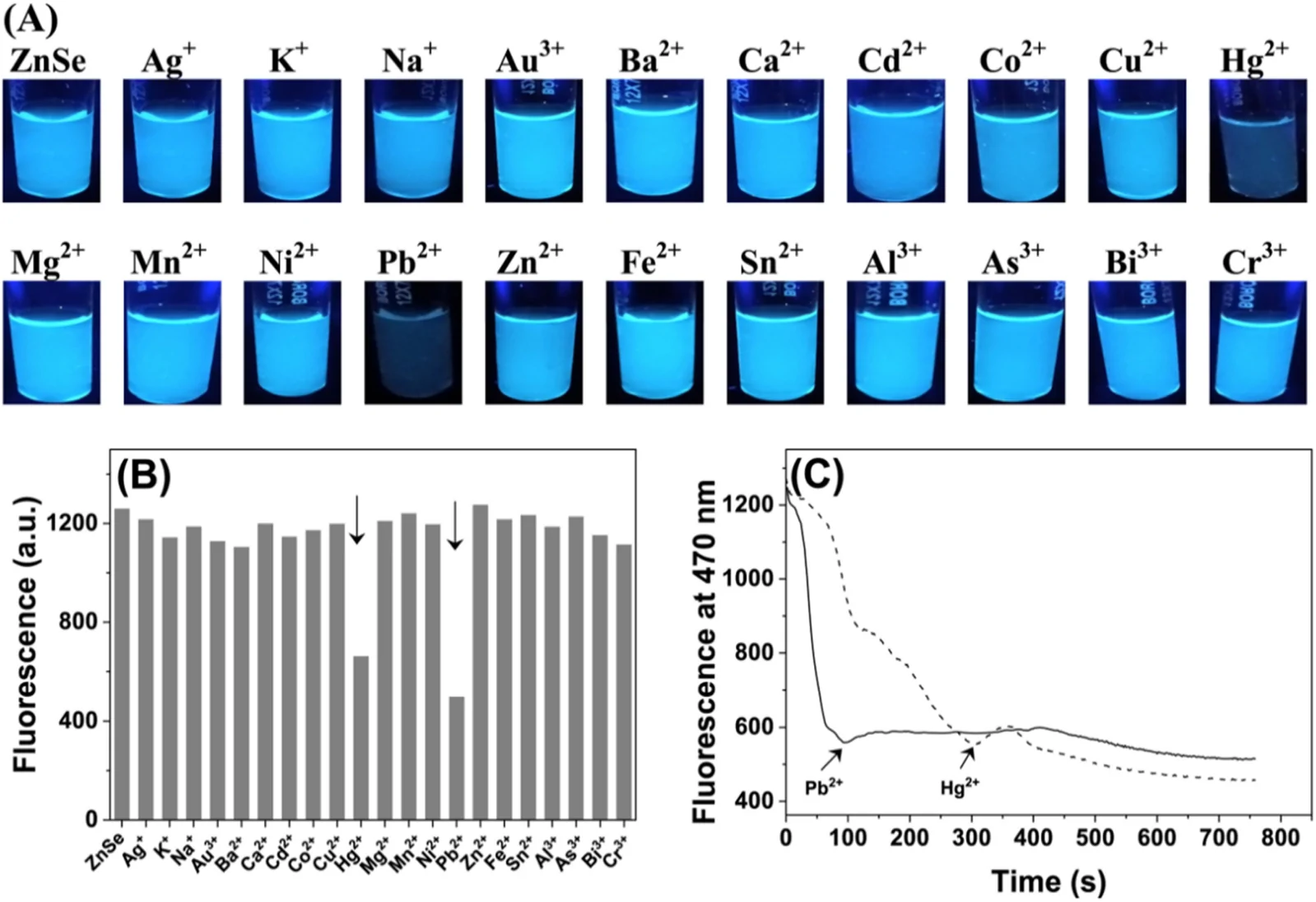
Visual and kinetic characterization of the NAC-ZnSe QD sensing platform. (A) Digital images of the ZnSe QDs under 360 nm UV illumination demonstrating the selective fluorescence quenching by Pb^2+^ and Hg^2+^. (B) Relative fluorescence intensities in the presence of various metal ions at 50 µM, highlighting the specificity for lead and mercury over a wide range of common cations. (C) Kinetic profiles of the fluorescence decay, illustrating the rapid interaction of Pb^2+^ (<90 s) compared to the significantly slower quenching process observed for Hg^2+^ (∼300 s).^249^

Wu *et al.* developed a “three-in-one” multimodal Pb^2+^ sensor integrating CdSe/ZnS QDs, luminol, and Fe_3_O_4_@SiO_2_ magnetic nanocomposites *via* DNA duplex/AuNP bridges, enabling simultaneous chemiluminescence, colorimetric, and fluorescence readout.^250^ In fluorescence “turn-on” mode, Pb^2+^-induced G-quadruplex formation unwinds the DNA duplex, releasing quenched QDs to restore signal (Fig. 9), giving a LOD of 1.63 nM across a 10 nM–10 µM linear range. Selectivity against nine interfering ions (Na^+^, K^+^, Cu^2+^, Hg^2+^, and others) relies on high-affinity G-rich DNA/Pb^2+^ binding. Magnetic separation enables a rapid mix-and-separate workflow for turbid/complex samples. River and lake water results correlated closely with ICP-MS. A noted limitation is inner-filter-effect signal loss from residual magnetic nanoparticles, with the authors flagging long-term stability of the magnetic architecture as a challenge for field deployment.

**Fig. 9:**
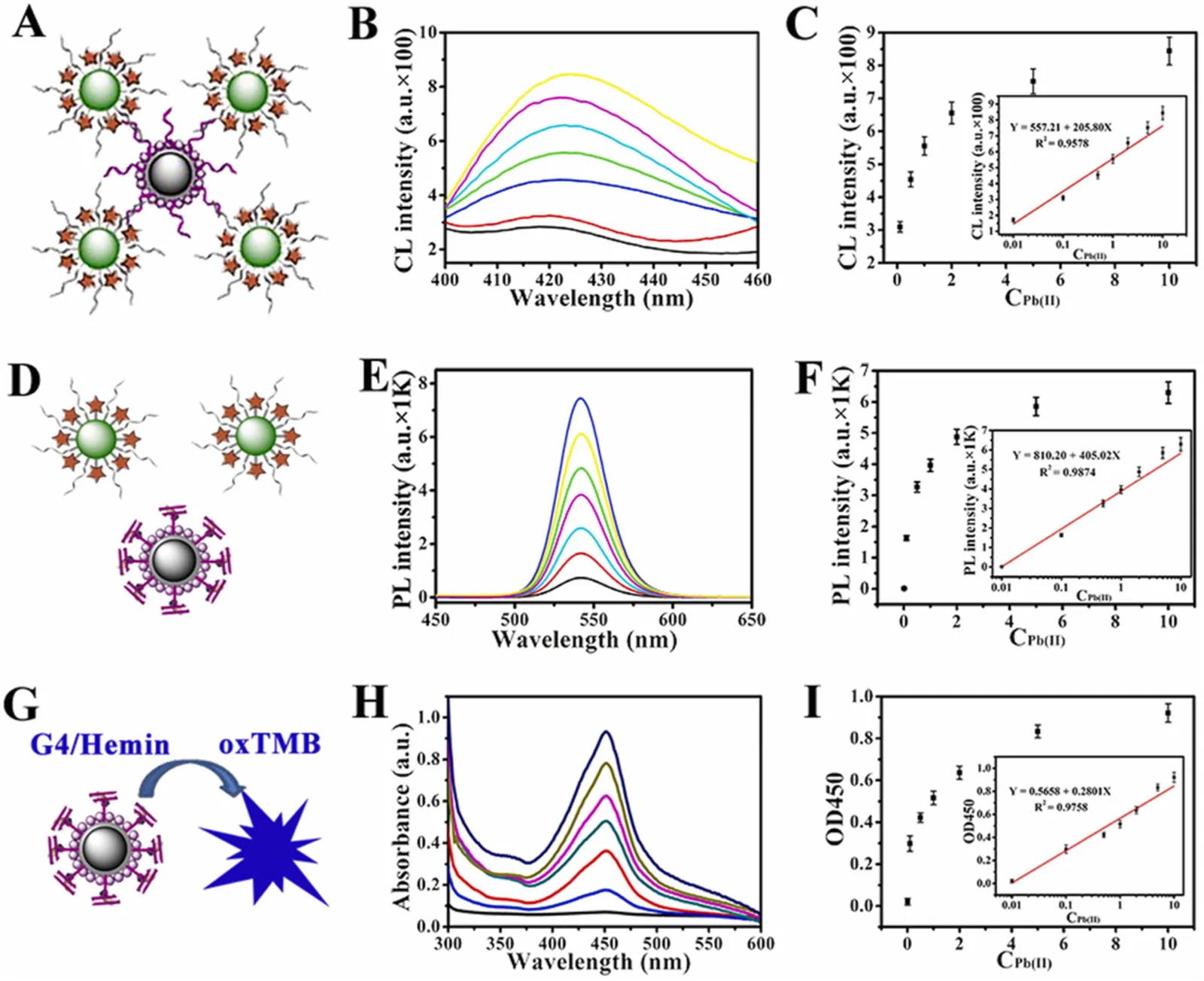
Multi-modal quantitative performance of the DNA-driven magnetic nanoprobe for Pb^2+^. The panels illustrate the concentration-dependent signal responses and corresponding calibration curves for the three integrated detection assays: (A–C) chemiluminescence (CL) based on released luminol; (D–F) fluorescence (FL) resulting from the “turn-on” release of CdSe/ZnS QDs from the magnetic quencher; and (G–I) colorimetric assay facilitated by the peroxidase-mimicking activity of the hemin/G-quadruplex complex. (B, E and H) Spectral responses with increasing Pb^2+^ concentration. (C, F and I) Signal intensity (or absorbance at 450 nm) *versus* Pb^2+^ concentration; insets show the linear calibration plots against the logarithm of Pb^2+^ concentration: CL, *Y* = 557.21 + 205.88*X* (*R*^2^ = 0.9576); FL, *Y* = 810.20 + 405.02*X* (*R*^2^ = 0.9874); colorimetric, *Y* = 0.5658 + 0.2801*X* (*R*^2^ = 0.9758), where *X* = log *C*_Pb(II)_. The FL mode shows the best linearity and highest sensitivity among the three assays.^250^

Mabrouk *et al.* developed water-dispersible, cadmium-free core/shell/shell ZnSeS/Cu:ZnS/ZnS QDs (3-MPA stabilized, Cu-related emission at 500 nm) for Pb^2+^ sensing *via* dynamic quenching, confirmed by PL lifetime and UV-vis measurements (collisional interaction plus energy transfer to a surface Pb^2+^-3-MPA complex).^251^ The probe achieved a LOD of 21 nM across a 0.04–6 µM linear range in ultrapure water, with minimal interference from Ca^2+^, Mg^2+^, and Zn^2+^, and naked-eye UV detection. Performance was validated in mineral and lake water (Fig. 10), maintaining sensitivity across varying ionic strengths. LOD is influenced by organic content and ionic strength; a consideration for field deployment across diverse matrices.

**Fig. 10:**
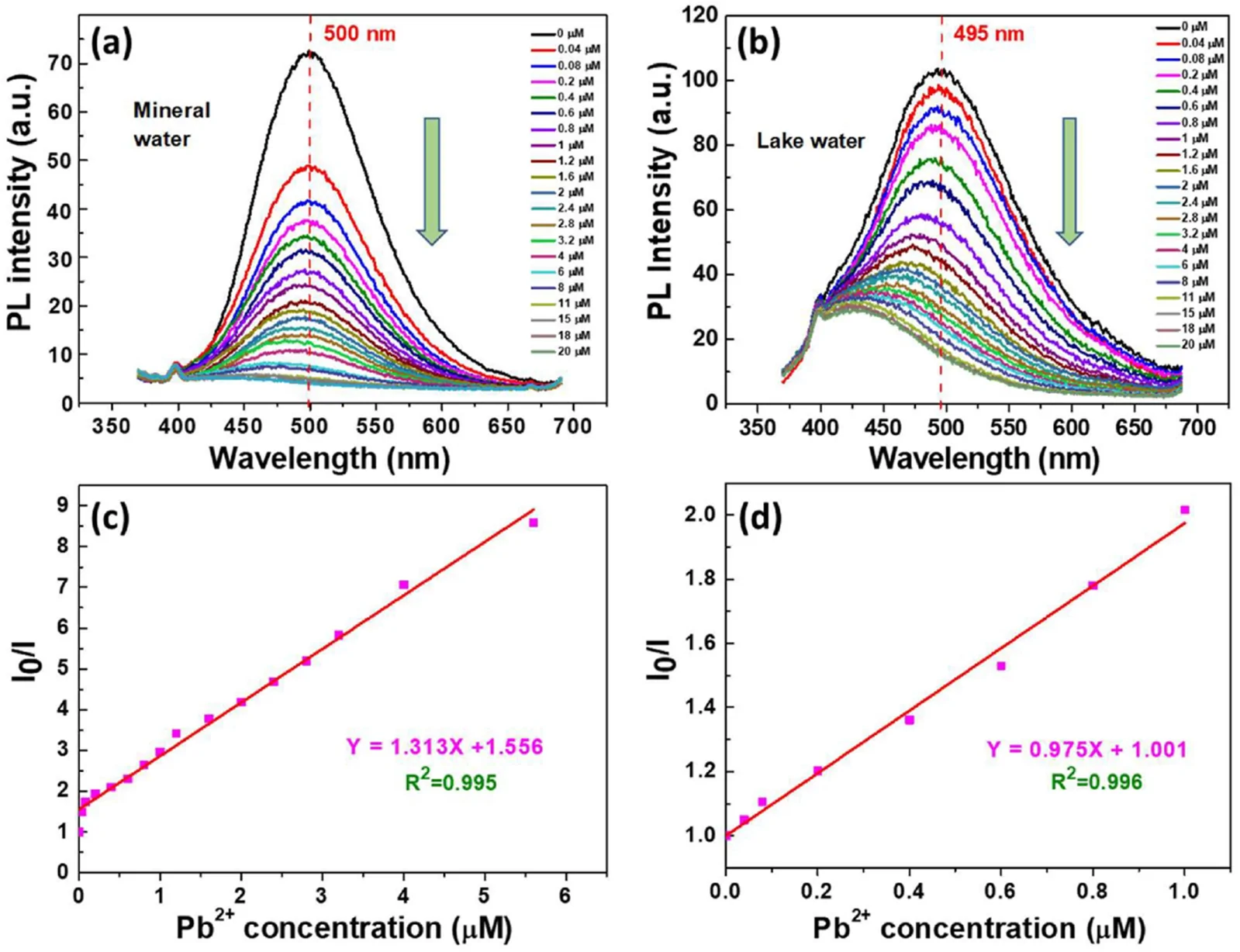
Real-sample analytical performance of ZnSeS/Cu:ZnS/ZnS QDs. The panels illustrate the photoluminescence quenching response upon the addition of Pb^2+^ in (a) mineral water and (b) lake water, demonstrating the probe's robustness in complex environmental media. Panels (c) and (d) show the corresponding linear calibration curves (*vs.* Pb^2+^ concentration), which were used to determine the LODs of 76 nM and 28 nM for these respective water sources.^251^

Pattnayak *et al.* demonstrated a highly sensitive platform for Pb^2+^ detection using l-GSH modified graphitic carbon nitride QDs (GSH@g-C_3_N_4_ QDs).^252^ These blue-emitting nanocrystals (*λ*_em_ = 420 nm) were prepared by covalently grafting GSH onto amine-functionalized g-C_3_N_4_ QD surfaces *via* EDC/NHS carbodiimide coupling, yielding 27% QY and robust resistance to photobleaching and high ionic strength. The sensing mechanism is static quenching, verified *via* TCSPC (near-constant ∼4.66 ns lifetime upon Pb^2+^ addition), UV-vis absorption (concentration-dependent decrease at 247 nm, confirming ground-state complex formation), and TEM (nanoparticle aggregation upon Pb^2+^ exposure) (Fig. 11). The nanoprobe achieved a LOD of 0.025 µM within a 0.01–0.1 µM linear range, outperforming several previously reported AuNP and chalcogenide QD-based sensors. Selectivity arises from lead binding to the thiol and carboxylate groups of the GSH shell; most common ions did not interfere, though Fe^3+^-induced signal suppression was mitigated with ascorbic acid. The platform was validated in tap and lake water, with recoveries exceeding 90%. A limitation is pH sensitivity: the sensor is optimized for neutral conditions (pH 7), as protonation/deprotonation of surface groups at extreme pH significantly suppresses baseline fluorescence.

**Fig. 11:**
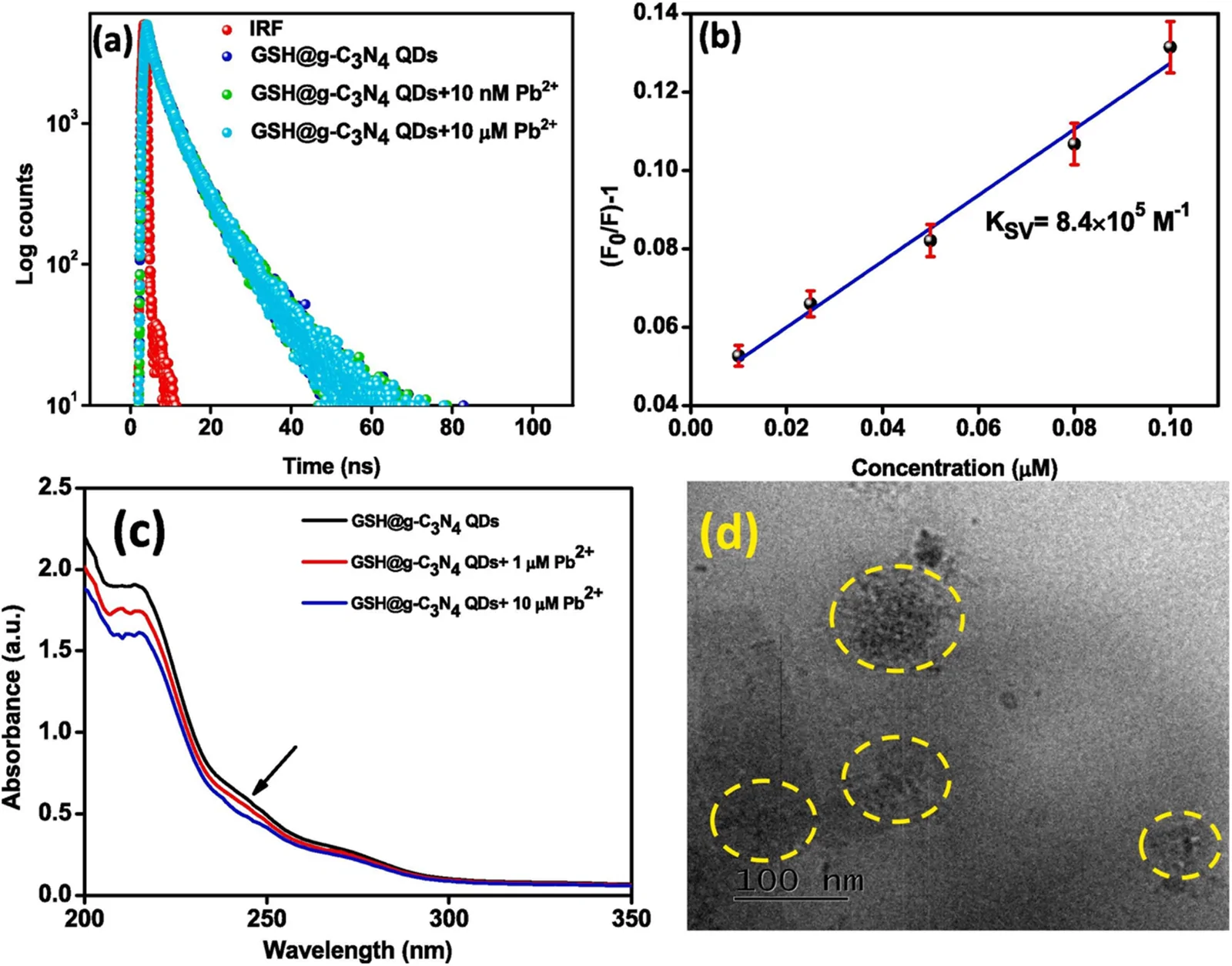
Mechanistic investigation of the GSH@g-C_3_N_4_ QD fluorescence quenching by Pb^2+^. (a) Fluorescence lifetime decay curves (TCSPC) showing the negligible change in the average lifetime (*τ*_avg_) upon addition of 10 nM and 10 µM Pb^2+^, a hallmark of a static quenching mechanism. (b) Stern–Volmer plot highlighting the linear relationship between relative fluorescence intensity and Pb^2+^ concentration. (c) UV-vis absorption spectra showing the decrease in the 247 nm electronic transition peak as lead ions are added, indicating the formation of a ground-state complex. (d) TEM micrograph providing visual evidence of lead-induced NP aggregation, which facilitates the observed fluorescence “turn-off” response.^252^

Jain *et al.* developed a “turn-on” fluorescent biosensor for Pb^2+^ integrating boron/nitrogen-doped CDs (BNCDs) within a carboxyl-functionalized terbium MOF (COOH-Tb MOF), silanized with a protective SiO_2_ layer and functionalized with a lead-specific GR-5 DNAzyme bioreceptor.^253^ In the dual-emissive design, blue and green nanoconjugate fluorescence is initially FRET-suppressed by Dabcyl and BHQ1 quenchers; Pb^2+^ activates the DNAzyme catalytic core, cleaving the substrate strand to release fluorescent fragments and restore signal in a concentration-dependent manner. This “turn-on” response occurs simultaneously across three emission peaks (450, 490, and 544 nm), providing a self-validating signal (Fig. 12). The platform achieved a LOD of 0.96 ppb (∼4.6 nM) within a 2 to 1000 nM linear range. Sequence-specific GR-5 DNAzyme recognition gave strong selectivity against Ni^2+^, Cu^2+^, Cd^2+^, and Zn^2+^. Quantum yields were notably high (60.21% BNCDs, 43% Tb-MOF), with stable photoluminescence over 60 days. Real-sample validation in handpump, lake, and tap water gave recoveries of 92 to 119%. The system requires precise buffer conditions, specifically Na^+^ and Mg^2+^ at neutral pH, to maintain DNAzyme stability and catalytic efficiency, a consideration for field-based monitoring.

**Fig. 12:**
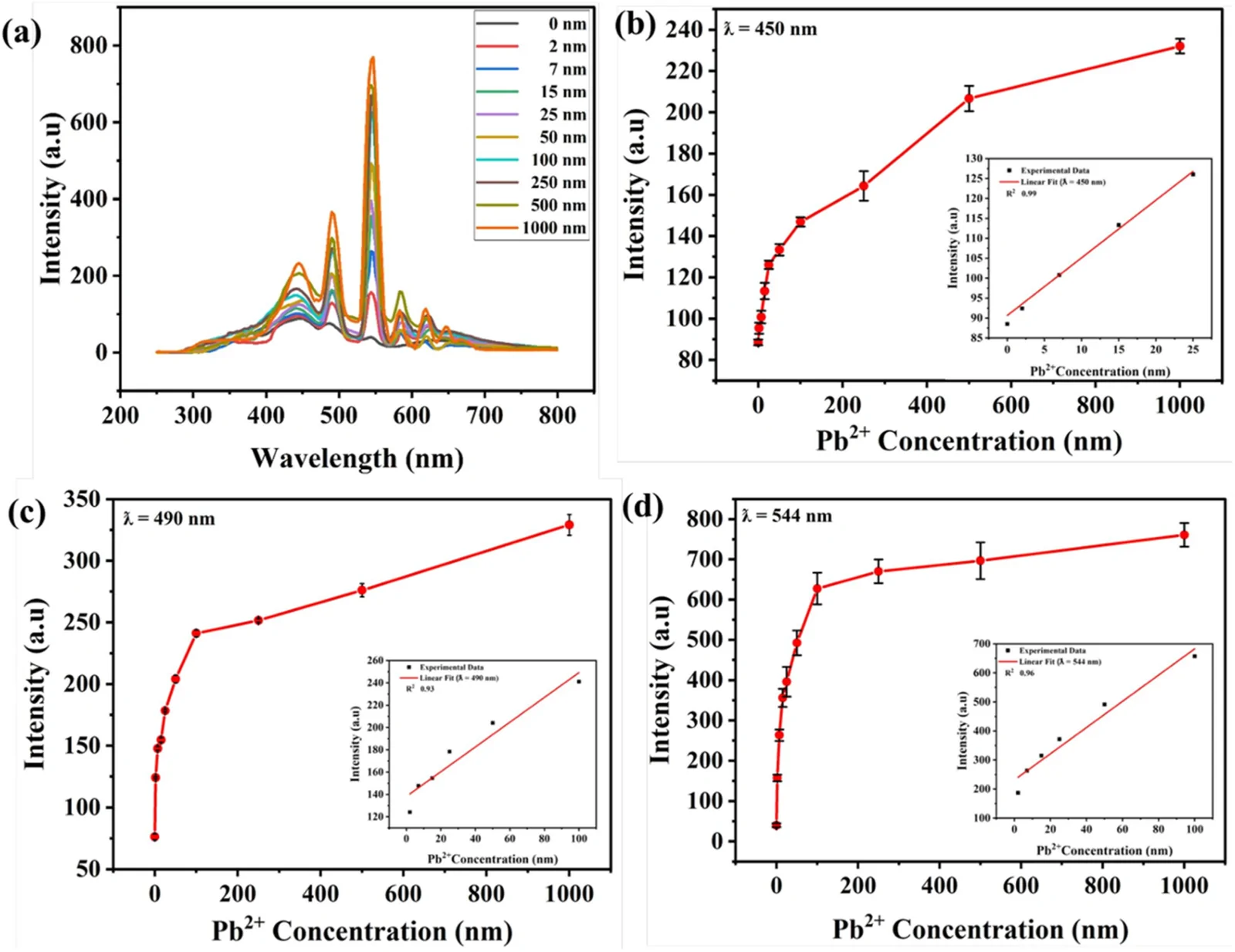
Quantitative fluorometric response of the BNCDs@Tb-MOF@DNAzyme biosensor to Pb^2+^. (a) Photoluminescence spectra showing the gradual restoration of fluorescence at 450, 490 and 544 nm as the Pb^2+^ concentration increases from 0 to 1000 nM, following catalytic cleavage of the quencher-tagged DNAzyme. (b–d) Fluorescence intensity at 450 nm (b), 490 nm (c) and 544 nm (d) as a function of Pb^2+^ concentration (0–1000 nM), showing signal saturation at higher concentrations. (Insets) Linear calibration plots in the low-concentration range, 0–25 nM at 450 nm (*R*^2^ = 0.99), and 0–100 nM at 490 nm (*R*^2^ = 0.93) and 544 nm (*R*^2^ = 0.96), enabling highly sensitive, multi-wavelength quantification of Pb^2+^.^253^

Cao *et al.* developed a PEC aptasensor using a CdSe/CdS/ZnO dual co-sensitized structure on an ITO electrode (ZnO nanosheets decorated with CdS *via* SILAR), where a hierarchical CdSe to CdS to ZnO electron transfer cascade suppresses recombination and maximizes photocurrent.^254^ In the absence of Pb^2+^, a CdSe-labeled cDNA probe hybridizes with the surface aptamer to enhance photocurrent; Pb^2+^ binding forms G-quadruplex structures that sterically block this hybridization, producing a concentration-dependent photocurrent decrease. The sensor achieved a LOD of 0.34 nM within a linear range of 1.0 × 10^−9^ to 1.0 × 10^−5^ M, with negligible interference from Ca^2+^, Co^2+^, Mg^2+^, and Ag^+^. Stability was maintained for at least three weeks at 4 °C. Recovery in pond, tap, and river water ranged 93 to 112%.

Guan *et al.* developed cadmium-free, water-soluble carboxymethyl chitosan-based CuInS_2_ (CMC-CuInS_2_) QDs (5.5 to 6.5 nm) *via* a green aqueous synthesis, with the polysaccharide shell providing both stabilization and a sulfur-rich interface for target interaction.^255^ Pb^2+^ interacts with surface S_2_-ions to form defect-enhancing complexes, inhibiting radiative recombination and producing a “turn-off” response with a LOD of 0.4 nM within a linear range of 1 × 10^−7^ to 8 × 10^−7^ M (Fig. 13). Selectivity against a broad range of interfering ions relies on lead's specific affinity for surface dangling bonds. Photostability was maintained for over 14 days. The technology was extended to complex biological settings, including intracellular Pb^2+^ detection in cancer cells and *in vivo* monitoring in zebrafish. The system requires optimization at pH 6 and shows linear sensitivity to temperature changes, a consideration for diverse field deployments.

**Fig. 13:**
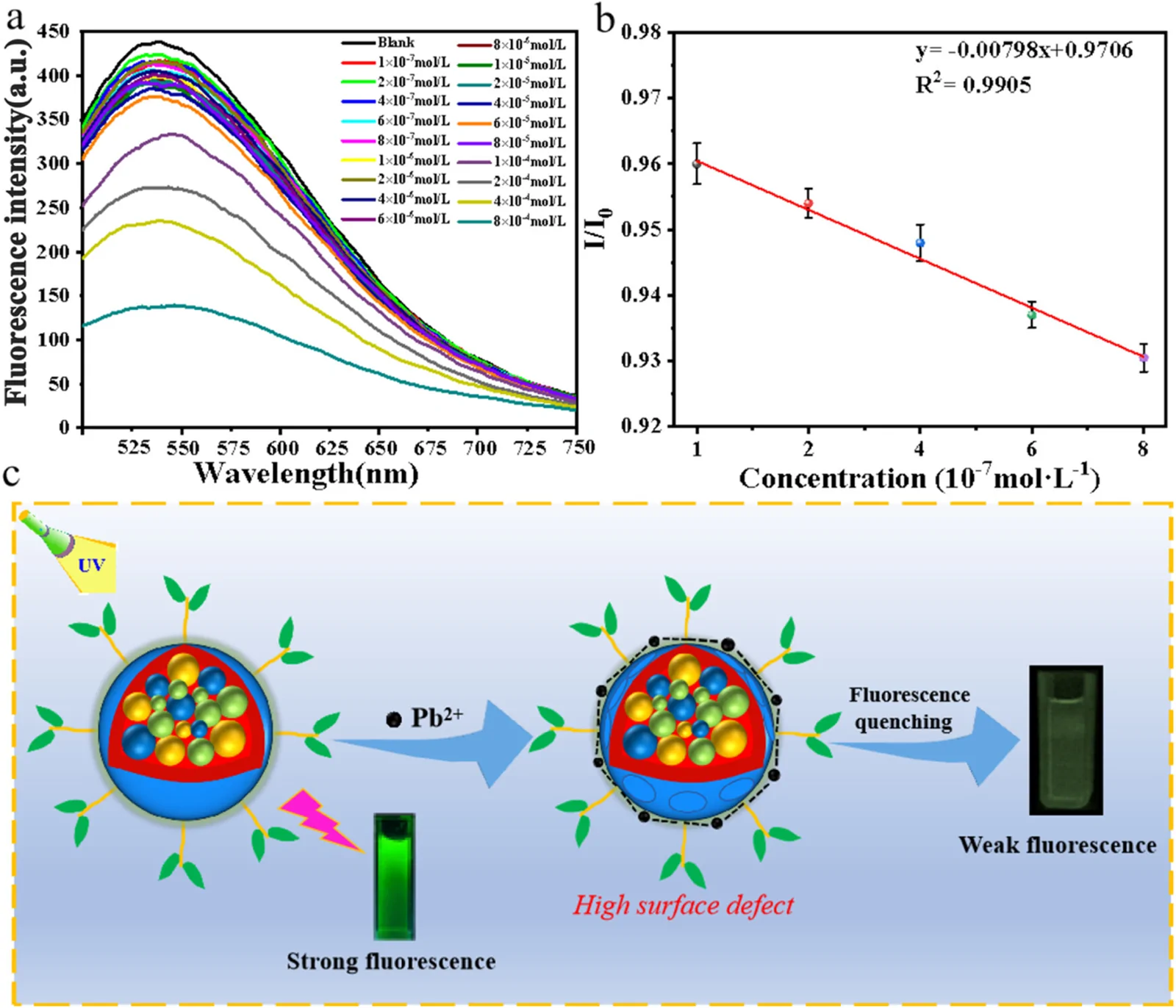
Quantitative fluorescence response and quenching mechanism of CMC-CuInS_2_ QDs. (a) Photoluminescence spectra showing concentration-dependent quenching of the green emission at 530 nm upon the addition of Pb^2+^ (0–0.8 µM). (b) Corresponding linear calibration curve illustrating the relationship between relative fluorescence intensity (*I*/*I*_0_) and Pb^2+^ concentration, exhibiting a high correlation coefficient (*R*^2^ = 0.9905) and a sub-nanomolar LOD. (c) Schematic illustration of the “turn-off” quenching mechanism, where the interaction of Pb^2+^ ions with surface sulfur atoms induces additional surface trap states, thereby suppressing radiative recombination.^255^

Aboobakri *et al.* developed nitrogen- and sulfur co-doped CDs (NSCDs) *via* one-step microwave-assisted pyrolysis (tri-ammonium citrate and thiourea precursors, 2.5 min synthesis), yielding water-dispersible nanodots (QY 11.5%, blue emission at 432 nm) for rapid Pb^2+^ detection.^256^ The “turn-off” response combines static and dynamic quenching, as Pb^2+^ coordinates with surface nitrogen and sulfur groups to promote non-radiative decay *via* electron transfer and ground-state complex formation. The probe achieved a LOD of 9.2 nM across two linear ranges (0.029 to 2.40 and 2.40 to 14.4 µmol L^−1^). Selectivity relied on heteroatom-surface affinity for Pb^2+^, though elevated Ag^+^ and Cu^2+^ caused signal perturbation. Optimal performance occurred at near-neutral pH (6.0 to 7.0). Reproducibility was strong (RSD < 3.5%), and the authors positioned the system as a cost-effective alternative to AAS or ICP-MS. Recovery in drinking water, tea, and rice ranged 96.5 to 107.0%. Careful control of probe concentration is required to avoid self-quenching in complex matrices.

Farahmandzadeh *et al.* developed thioglycolic acid (TGA)-stabilized CdS QDs *via* a rapid one-pot photochemical route, with TGA serving as both stabilizing ligand and sulfur precursor (average size ∼2.8 nm, broad yellow defect-related emission).^257^ As a fluorescence “off” sensor, the probe achieved a LOD of 60 nM across a 20 to 60 µM linear range (*R*^2^ = 0.9956), with complete quenching at 100 µM. The mechanism involves electron transfer from CdS to Pb^2+^ and *in situ* PbS formation, disrupting TGA ligand coordination and triggering aggregation-induced quenching. Partial quenching was also observed with Ni^2+^, Hg^2+^, and Fe^2+^. Beyond sensing, the same CdS QDs functioned as photocatalysts, achieving up to 94.9% degradation efficiency for methylene orange and rhodamine B, positioning the platform for combined sensing and remediation applications.

Sebastian *et al.* developed cadmium-free GQDs functionalized with a dithienopyrrole ligand (DTPAN-fn-GQD) *via* top-down hydrothermal synthesis and amidation coupling, with the electron-donating DTPAN moiety enhancing photoluminescence and redshifting emission.^258^ DFT and cyclic voltammetry indicated HOMO to LUMO electron transfer from DTPAN to the GQD; Pb^2+^ disrupts this pathway *via* selective coordination with DTPAN sulfur atoms (consistent with HSAB soft–soft affinity), producing concentration-dependent “turn-off” quenching. The sensor achieved an ultralow LOD of 0.25 nM across a 0.5 to 40 nM linear range, outperforming many previously reported carbon-based probes, with strong selectivity against Hg^2+^, Cd^2+^, and Fe^3+^. The system was deployed as coated paper strips for on-site visual detection and validated in river, tap, and well water with recovery close to 100%. Optimal performance is restricted to neutral pH (7.0), as acidic conditions protonate coordinating ligands and alkaline conditions promote lead hydroxide precipitation.

Qian *et al.* developed an electrochemical sensor using Cu-doped CeO_2_ solid-solution QDs (SSQDs, ∼4.7 nm) immobilized on sakura petal-derived porous biocarbon (290.42 m^2^ g^−1^ surface area), which suppresses aggregation and promotes uniform dispersion.^259^*Via* anodic stripping voltammetry, Cu doping enhances the reversible Ce^3+^/Ce^4+^ redox couple, which selectively catalyzes Pb^2+^ reduction while the porous carbon scaffold accelerates ion diffusion. The sensor achieved a LOD of 5.652 pM, among the lowest reported for electrochemical Pb^2+^ sensors, across a broad 0.48 to 480 nM linear range, with selectivity against Mn^2+^, Co^2+^, Cd^2+^, and Cu^2+^, RSD of 1.9%, and stable performance over 30 days. Recovery in river water ranged 97.6 to 105.0%. The solid-solution structure is sensitive to hydrothermal treatment conditions; excessive reaction time or heat can segregate monoclinic CuO nanoparticles, compromising the cooperative redox behavior.

#### 5.1.1. Critical analysis

Despite significant advances in QD architectures for lead ion sensing, the transition from laboratory demonstrations to practical environmental monitoring remains constrained by unresolved technical and fundamental challenges. A comprehensive analysis of recent literature reveals systematic gaps in mechanistic validation, environmental robustness, and translational feasibility that collectively limit the real-world deployment of these nanomaterial-based platforms. Table 14 provides a comparative overview of representative QD-based Pb^2+^ sensing platforms developed, summarizing synthesis methodologies, LODs, selectivity profiles, and real-sample validation data that form the basis for the following critical assessment. While picomolar to nanomolar LODs dominate the literature, with values as low as 5.652 pM reported for Cu-doped CeO_2_ QDs, the practical utility of such sensitivities in authentic environmental matrices remains questionable.^251^ Systematic examination of recovery data reveals a recurring pattern, sensors optimized in phosphate-buffered saline or Tris–acetate buffers exhibit substantial performance degradation when challenged with complex matrices. ZnSeS/Cu:ZnS/ZnS core/shell systems exemplify this limitation, demonstrating a nearly four-fold increase in LOD from 21 nM in ultrapure water to 76 nM in mineral water and 28 nM in lake water, attributed to elevated ionic strength effects and competitive matrix interactions. Similarly, Mn-doped ZnS QDs, despite achieving sub-nanomolar sensitivity in controlled conditions, are explicitly reported to suffer from “complexity of practical samples and unknown interferences” when applied to food matrices or environmental waters. This disconnect between claimed sensitivity and validated performance suggests that many reported LODs reflect instrumental precision under idealized conditions rather than true analytical figures of merit for real-world deployment. The absence of standardized testing protocols using certified reference materials with known interferent profiles further obscures comparative assessment across platforms.^248^

**Table 14:** Comparative analysis of nanomaterial-based sensing platforms for Pb^2+^ detection

Nanomaterial system	Synthesis method	Functionalization	Detection method	Sensing mechanism	LOD	Linear range (M)	Selectivity against	Optimal pH	Real sample recovery	Key advantages	Limitations
CdTe QDs/DNA tetrahedron/silica/GO^247^	Hydrothermal + Stöber	DNA sandwich; N_3_-modified DNA	Ratiometric fluorescence	Static quenching (gQD by GO *via* π–π stacking)	1.7 nM	1.0 × 10^−8^ to 2.0 × 10^−6^	Ag^+^, Hg^2+^, Mn^2+^, Fe^2+^, Zn^2+^, Cu^2+^	7.0	95.3–108.0% (soil, cosmetics, milk, serum)	Rigid FNA structure improves recognition; ratiometric self-calibration	DNA crowding causes steric hindrance at high density
Mn-doped ZnS QDs^248^	One-pot aqueous	Mn-doping	Ratiometric fluorescence	Aggregation-induced quenching	0.5 nM	1.0 × 10^−8^ to 6.0 × 10^−8^	Cu^2+^, Hg^2+^, Cd^2+^, Mg^2+^, Ca^2+^, Co^2+^	7.0	84.0–100.3% (apple, lake water)	Simple one-pot synthesis; independent of system concentration	Complexity of practical samples and unknown interferences
AgInS_2_ QDs^246^	One-pot water phase	GSH-stabilized	Fluorescence quenching	Dynamic quenching (light-induced electron transfer)	4.0 nM	9.0 × 10^−9^ to 5.3 × 10^−7^	K^+^, Na^+^, Ba^2+^, Ca^2+^, Mg^2+^, Zn^2+^, Mn^2+^, Co^2+^	7.12	92.2–102.6% (water samples)	Low toxicity; high photostability (60 days); wide range	Oxygen in air captures electrons, causing errors
ZnSe QDs^249^	Aqueous reflux	*N*-Acetyl cysteine (NAC) capped	Fluorescence quenching	Static quenching (ZnSe-Pb^2+^ non-fluorescent complex)	0.37 µM	0.0 to 1.0 × 10^−5^	Ag^+^, Hg^2+^, Au^3+^, Cu^2+^, Mn^2+^, Cr^3+^	3.0–11.0	89–112% (water, juices, artificial urine/saliva/CSF)	Rapid response (<90 s); cadmium-free; high pH stability	Not reported
CdSe/ZnS-luminol/Fe_3_O_4_ (ref. 250)	Hydrothermal + magnetic assembly	AuNP; luminol; DNA duplex	FL/CL/colorimetric	FL turn-on (target-induced release from AuNP quencher)	1.63 nM	1.0 × 10^−8^ to 1.0 × 10^−5^	Na^+^, K^+^, Ca^2+^, Mg^2+^, Cu^2+^, Fe^3+^, Hg^2+^	6.0	Consistent with ICP-MS (river/lake water)	Three-in-one readout; magnetic separation for high efficiency	IFE from residual magnetic particles
ZnSeS/Cu:ZnS/ZnS QDs^251^	One-pot aqueous	Cu-doped; 3-mercaptopropionic acid	Fluorescence quenching	Dynamic quenching (electron/energy transfer)	21 nM	4.0 × 10^−8^ to 6.0 × 10^−6^	Hg^2+^, Cd^2+^, Co^2+^, Al^3+^, Fe^3+^, Ni^2+^	7.0	28–76 nM LOD (mineral/lake water)	Large Stokes shift; minimum self-absorption; non-toxic	LOD increases in mineral water due to higher ionic strength
GSH@g-C_3_N_4_ QDs^252^	Carbodiimide coupling	l-GSH modified	Fluorescence quenching	Static quenching (ground state complex formation)	0.025 µM	1.0 × 10^−8^ to 1.0 × 10^−7^	Hg^2+^, Al^3+^, Cd^2+^, Cu^2+^, Ni^2+^, Fe^3+^	7.0	>90% (tap/lake water)	Exceptional stability; high QY (27%); non-toxic	Slight quenching by Fe^3+^ requires ascorbic acid treatment
BNCDs/COOH-Tb MOF^253^	*In situ* hydrothermal	Boron, nitrogen doped; Tb-MOF	Fluorescence turn-on	FRET restoration (DNAzyme cleavage of quencher strand)	2.89 nM	2.0 × 10^−9^ to 1.0 × 10^−6^	Ni^2+^, Cu^2+^, Cd^2+^, Mn^2+^, Zn^2+^, Co^2+^	7.26	92–119% (tap, handpump, lake water)	Dual blue-green emission; naked eye detection possible	Not reported
CdSe QDs/ZnO-CdS^254^	Hydrothermal + SILAR	Aptamer-functionalized	Photoelectrochemistry	Aptamer binds Pb^2+^, inhibiting CdSe sensitization	0.34 nM	1.0 × 10^−9^ to 1.0 × 10^−5^	Ca^2+^, Co^2+^, Mg^2+^, Ag^+^	7.4	93–112% (pond, tap, river water)	Cascade electron transfer system; high sensitivity	Not reported
CMC-CuInS_2_ QDs^255^	Direct aqueous interaction	Carboxymethyl chitosan (CMC-SH)	Fluorescence quenching	Complex formation (enhanced surface trap states)	0.4 nM	1.0 × 10^−7^ to 8.0 × 10^−7^	Hg^2+^, Fe^3+^, Co^2+^, Mn^2+^, K^+^, Na^+^	6.0	Cell/zebrafish imaging validated	Specific staining of cell membranes; non-toxic; green synthesis	Luminescence highly sensitive to temperature changes
Cu-doped CeO_2_ SSQDs^259^	Hydrothermal	Cu-doped; sakura-petal biocarbon	Anodic stripping voltammetry	Redox catalysis (Ce^3+^ ↔ Ce^4+^ transition)	5.652 pM	4.8 × 10^−10^ to 4.8 × 10^−7^	Mn^2+^, Co^2+^, Cd^2+^, Cu^2+^, Zn^2+^, Mg^2+^	4.2	97.6–105.0% (natural river water)	Ultra-low LOD; biocarbon microreactor prevents aggregation	Prolonged processing forms discrete CuO NPs
DTPAN-fn-GQD^258^	Hydrothermal + amidation	Dithienopyrrole (DTPAN) functionalized	Fluorescence quenching	Combined static and dynamic quenching	0.25 nM	5.0 × 10^−10^ to 4.0 × 10^−8^	Hg^2+^, Cd^2+^, Ni^2+^, Fe^3+^, Al^3+^, PA	7.0	96.25–105.53% (river, tap, well water)	Visual paper strips; non-toxic; bandgap tuning	Optimal response strictly limited to neutral pH
CdS QDs^257^	Photochemical	Thioglycolic acid (TGA) stabilized	Fluorescence quenching	Conduction band electron transfer; PbS formation	60 nM	2.0 × 10^−5^ to 6.0 × 10^−5^	Ni^2+^, Hg^2+^, Fe^2+^, Co^2+^, Sr^2+^	9.0	Not reported	Rapid synthesis; bifunctional (sensing + photocatalyst)	Non-selective quenching by several coexisting ions
N,S co-doped CDs^256^	Microwave pyrolysis	N,S co-doping	Fluorescence quenching	Combined static and dynamic quenching	9.2 nM	2.9 × 10^−8^ to 1.4 × 10^−5^	Ca^2+^, Cd^2+^, Hg^2+^, Fe^3+^, Ni^2+^, Al^3+^	6.0–7.0	96.5–107.0% (potable water, tea, rice)	Ultra-fast synthesis (2.5 min); high repeatability	Potential self-quenching at high concentrations

A primary challenge in the development of QD sensing platforms is the significant interference from chemically similar transition metal ions, which frequently induce competitive quenching of the fluorescent signal. Cross-study analysis reveals that Hg^2+^, Cu^2+^, and Fe^3+^ constitute recurrent interferents across 71%, 50%, and 50% of surveyed platforms, respectively, indicating that heavy metal cross-reactivity remains a fundamental limitation rather than an isolated challenge. Specific quantitative examples illustrate the severity of this problem: nitrogen-sulfur co-doped CDs experience signal disruption from Ag^+^ and Cu^2+^ when these interferents are present at concentrations merely ten times higher than the target Pb^2+^ concentration, a ratio readily encountered in contaminated industrial effluents or mining waste streams. Fe^3+^ interference proves particularly problematic, requiring the addition of ascorbic acid as a specific reducing agent to prevent signal distortion during detection in GSH@g-C_3_N_4_ QD systems. CdS-based sensors demonstrate non-specific signal suppression from Ni^2+^, Hg^2+^, and Fe^2+^, exhibiting what is explicitly described as “non-selective quenching by several coexisting ions” under alkaline conditions, making it difficult to achieve a definitive Pb^2+^ signal in multi-ion matrices. Manganese-doped ZnS probes face similar challenges with Cu^2+^, Hg^2+^, and Cd^2+^, often mandating the use of specialized masking agents such as 2,3-dimercapto-1-propanol to maintain analytical selectivity. The theoretical basis for selectivity, primarily invoking Hard–Soft Acid–Base (HSAB) theory or surface ligand coordination, proves insufficient in mixed-ion scenarios characteristic of industrial wastewater, biological fluids, or contaminated groundwater. More concerning is the limited exploration of competitive binding kinetics; selectivity experiments typically involve sequential exposure to individual interferents rather than simultaneous multi-ion challenges that would more accurately simulate field conditions. The reliance on aptamer or DNAzyme functionalization in approximately 20% of studies represents a promising but underexploited strategy, yet even these biorecognition elements exhibit suboptimal discrimination in the presence of structurally similar divalent cations.^252^

A critical examination of reported quenching mechanisms reveals substantial mechanistic ambiguity across the literature. While studies routinely classify their sensors as employing “static quenching”, “dynamic quenching”, or “combined mechanisms”, the experimental evidence supporting such assignments remains insufficient. Stern–Volmer analysis, the foundational technique for differentiating static from dynamic processes, is inconsistently applied, and temperature-dependent quenching studies, which provide definitive mechanistic discrimination, are conspicuously absent from most reports. Time-resolved fluorescence spectroscopy, which directly probes excited-state lifetimes and unambiguously distinguishes collision-based dynamic quenching from ground-state complex formation, has been employed in fewer than 15% of the surveyed systems. This methodological gap is particularly problematic for systems claiming, “combined static and dynamic quenching”, where the relative contributions of each pathway and their pH- or temperature-dependent evolution remain uncharacterized. Furthermore, the role of aggregation-induced quenching *versus* true molecular-level interactions is rarely substantiated through electron microscopy or dynamic light scattering measurements conducted in the presence of analyte. In Cu-doped QDs, it is often difficult to definitively assign specific fluorescence decay components to either the Cu dopant transition or to intrinsic defects such as Zn vacancies and S/Se dangling bonds. This complexity is exacerbated in ternary QDs, were broad size distributions and unequal composition distributions result in poorly resolved absorption spectra, complicating the precise characterization of electronic transitions. Recent reviews emphasize that the specific degradation mechanisms involving emission quenching and morphological inhomogeneity in eco-friendly QD light-emitting diodes and QDs remain “unclear” and require deeper investigation to be fully understood. The consequence of this mechanistic vagueness is twofold: fundamental understanding of analyte-QD interactions remains incomplete, and rational sensor optimization lacks a robust theoretical foundation.^258^

Photochemical stability and long-term signal reproducibility constitute underreported yet operationally critical parameters. Only one study explicitly documents 60-day photostability under ambient storage, while the majority provide no information regarding shelf life, photobleaching kinetics, or signal drift over extended monitoring periods. Environmental factors known to compromise QD optical properties are acknowledged as limitations in specific cases but are not systematically characterized across platforms. Chalcogenide-based QDs, such as AgInS_2_, are particularly susceptible to atmospheric oxygen, which can capture photo-generated electrons from the conduction band and lead to unintended quenching, necessitating storage in vacuum or inert environments yet no compensatory strategies or encapsulation approaches are proposed. Solid solution sub-stoichiometric QDs can be compromised by prolonged heat treatment or hydrothermal processing, which leads to the formation of discrete CuO NPs and the loss of catalytic synergy, as explicitly observed in Cu-doped CeO_2_ systems where “prolonged processing forms discrete CuO NPs”. In advanced FNA designs, high-density DNA grafting on silica nanospheres can create “crowding” and steric hindrance, which significantly weakens the binding efficiency of Pb^2+^ aptamers. CMC-CuInS_2_ systems exhibit “luminescence highly sensitive to temperature changes”, where increased thermal energy promotes intermolecular collisions and non-radiative energy loss, rendering them unsuitable for field deployment without thermal stabilization. The relatively weak interaction between many QDs and their surface ligands often results in ligand detachment over time, promoting NP aggregation and undermining chemical stability. Batch-to-batch reproducibility data are virtually absent, raising concerns about the manufacturability and quality control feasibility of these nanomaterials. The potential for concentration-dependent self-quenching, explicitly noted for N,S-codoped CDs, suggests that probe working concentration windows may be narrower than reported linear ranges indicate, yet systematic optimization of QD loading *versus* signal linearity is rarely presented. Sensor performance is also strictly dependent on narrow environmental parameters, such as a strictly neutral pH of approximately 7.0; acidic conditions can cause ligand protonation and aggregation, while basic conditions lead to target hydrolysis, as exemplified by systems demonstrating optimal response “strictly limited to neutral pH”.^246^

A striking contradiction emerges from toxicity and regulatory considerations: approximately 35% of high-performance sensors employ cadmium-based QDs (CdTe, CdSe/ZnS, CdS) despite the November 2025 expiration of the European Union's RoHS Exemption 39a, which mandates cadmium content below 100 ppm in commercial electronics and analytical devices. While these cadmium-containing systems demonstrate superior photoluminescence QYs (95–100%) and narrow emission linewidths (18–25 nm), their deployment contradicts the very environmental protection objectives motivating lead detection efforts. Despite the purported shift toward “heavy-metal-free” alternatives, material toxicity and regulatory gaps remain major hurdles, with a significant portion of commercial displays currently entering the market still containing intentionally added cadmium. Manufacturers have been documented exploiting regulatory loopholes in RoHS standards by increasing the thickness and weight of components to lower the parts-per-million concentration while maintaining high absolute levels of toxic metals. Additionally, some proposed substitutes, such as lead halide perovskite QDs, contain high absolute levels of lead and exhibit poor environmental stability, potentially releasing toxic ions into the environment if the host matrix is compromised during disposal or device failure. Cadmium-free alternatives, including InP/ZnSe/ZnS, ZnSeTeS, and CDs, have achieved significant performance gains (PLQY exceeding 90% for InP red emitters and 17 nm FWHM for ZnSeTeS blue emitters), yet their adoption in Pb^2+^ sensing applications lags their cadmium-based counterparts. This disparity likely reflects the maturity gap in cadmium-free synthesis protocols, which remain more challenging to optimize for consistent particle size distribution and surface passivation. The regulatory trajectory is clear: sensors based on Group 1 carcinogens will face increasing commercialization barriers, necessitating accelerated development of eco-friendly alternatives that match cadmium-based performance benchmarks.^260^

Manufacturing scalability represents a critical yet largely unaddressed challenge. Hot-injection and hydrothermal synthesis routes, employed in approximately 65% of surveyed systems require high-temperature reactions (160–200 °C), inert atmospheres, and precise temporal control of precursor addition, rendering them incompatible with industrial-scale continuous production. Batch-to-batch variability, acknowledged in general terms but rarely quantified, likely stems from nucleation kinetics sensitive to minor temperature gradients, precursor purity variations, and reactor geometry differences. Microwave-assisted pyrolysis, which achieves sub-3-minute synthesis times for N,S-codoped CDs, offers a promising alternative, yet scalability to multi-kilogram production and the reproducibility of microwave power distribution across large sample volumes remain unexplored. The transition from milligram-scale laboratory synthesis to commercial manufacturing will require systematic process optimization, inline quality monitoring, and failure mode analysis, none of which are addressed in current literature. Perhaps the most significant barrier to field deployment is the near-total absence of standardized testing protocols, cross-laboratory validation studies, and integration into deployable sensor formats. There is currently no standardized definition for critical performance indicators such as “device lifetime” or “stability”, leading to inconsistent reporting across different research groups and precluding objective inter-study comparisons. Fewer than 10% of reported systems progress beyond solution-phase fluorescence measurements to demonstrate practical implementation formats such as paper-based lateral flow strips, electrochemical electrodes, or microfluidic cartridges. The single example of visual paper strip integration (DTPAN-functionalized GQDs) highlights the feasibility of portable formats, yet this remains an isolated case rather than a systematic development trend. In multifunctional platforms that utilize magnetic separation for enhanced efficiency, residual magnetic particles can interfere with fluorescence signals through the IFE, complicating the readout and introducing systematic measurement errors. Furthermore, achieving high-resolution pixel arrays for large-area display or sensor array applications remains a “formidable obstacle” because QDs tend to degrade during common industrial processes like inkjet printing or photolithography, limiting their integration into mass-manufactured devices. The lack of interlaboratory comparisons using identical sample sets precludes objective performance benchmarking. Real-sample validation spans an eclectic range of matrices, from cosmetics and rice to cerebrospinal fluid, without consensus on priority applications or matrix-specific performance requirements. This fragmentation impedes regulatory approval pathways and hinders end-user adoption by environmental monitoring agencies, clinical diagnostics providers, or industrial quality control laboratories.^259^

Advancing QD-based lead detection from academic demonstrations to operational environmental sensors requires focused attention on several critical areas. First, mechanistic studies employing time-resolved spectroscopy, temperature-dependent quenching analyses, and thermodynamic modeling must replace qualitative quenching classifications to enable rational sensor design and performance prediction. Second, standardized interference testing protocols incorporating simultaneous multi-ion challenges at environmentally relevant concentrations should be established and adopted across the research community, with particular attention to defining masking agent requirements and selectivity coefficients. Third, long-term stability assessments under field-relevant conditions, including UV exposure, temperature cycling, oxygen and humidity exposure, and extended storage exceeding six months, must become mandatory reporting requirements with standardized accelerated aging protocols. Fourth, the development and validation of cadmium-free alternatives must accelerate to align sensor performance with regulatory constraints, accompanied by full life-cycle toxicity assessments that consider both precursor and end-of-life disposal risks. Fifth, scalable synthesis methodologies compatible with continuous manufacturing, rigorous quality control, and batch-to-batch reproducibility must be prioritized over exotic techniques offering marginal performance gains, with explicit demonstration of kilogram-scale production feasibility. Sixth, standardized definitions for critical performance metrics including “device lifetime”, “stability”, and “reproducibility” must be established through inter-laboratory consensus efforts, potentially coordinated through international standards organizations. Finally, cross-disciplinary collaboration between materials scientists, analytical chemists, process engineers, and regulatory agencies is essential to define application-specific performance criteria, establish certified reference materials, create validation frameworks, and develop practical sensor formats that bridge the gap between laboratory innovation and real-world impact. Only through systematic resolution of these foundational gaps can QD-based Pb^2+^ sensors fulfill their considerable promise as next-generation environmental monitoring tools capable of deployment in complex field scenarios.

### 5.2. Detection of mercury (Hg)

Mercury contamination represents a critical global health threat, with the WHO estimating that over 15 million people worldwide are exposed to hazardous mercury levels through artisanal gold mining alone, while many more face exposure through contaminated fish consumption and industrial emissions.^261,262^ The unique chemical properties of mercury, including its volatility, multiple oxidation states, and tendency to form organometallic compounds, distinguish it from other heavy metals and create specific analytical challenges that have driven the development of specialized sensing technologies. Mercury exists in the environment in three principal forms, each with distinct physicochemical behavior and toxicological profiles. Elemental mercury (Hg^0^) exists as a liquid metal at room temperature with high volatility, representing the primary route of occupational exposure and dental amalgam-related concerns. Inorganic mercury ions (Hg^2+^ and Hg_2_^2+^) are water-soluble species that exhibit nephrotoxic properties and primarily originate from industrial discharge and mining operations. Organomercury compounds, particularly methylmercury (CH_3_Hg^+^) and ethylmercury (C_2_H_5_Hg^+^), are lipophilic species that bioaccumulate extensively in aquatic food chains and pose the most severe neurological risks.^263^

Inorganic mercury (Hg^2+^) binds to sulfhydryl groups in proteins, disrupting enzymatic function and causing acute kidney damage, gastrointestinal distress, and immune system dysfunction. The particularly insidious threat, however, comes from methylmercury (CH_3_Hg^+^), formed by anaerobic bacteria in aquatic sediments through biomethylation of inorganic mercury. Methylmercury readily crosses biological barriers including the blood–brain barrier and placenta, causing irreversible neurological damage, particularly during fetal development.^264^ The Minamata disaster in Japan (1956) and more recent incidents in Iraq (1971) demonstrated the catastrophic consequences of methylmercury exposure, including severe neurological disorders, developmental delays, and death, prompting international regulatory action and intensive research into mercury detection and remediation strategies.^265^ The WHO establishes guideline values of 6 µg L^−1^ (30 nM) for inorganic mercury and 1 µg L^−1^ (5 nM) for total mercury in drinking water, though the U.S. EPA adopts a more stringent limit of 2 µg L^−1^ (10 nM).^266^ These exceptionally low regulatory thresholds necessitate analytical methods capable of detecting mercury at nanomolar to sub-nanomolar concentrations in diverse sample matrices.

Conventional analytical techniques including cold vapor AAS (CV-AAS), cold vapor atomic fluorescence spectroscopy (CV-AFS), and ICP-MS achieve the requisite sensitivity for regulatory compliance monitoring but suffer from significant practical limitations including extensive sample pretreatment requirements (acid digestion, chemical reduction, purge-and-trap concentration), expensive instrumentation with capital costs ranging from $50 000 to $500 000, dependence on trained personnel and centralized laboratory facilities, lengthy analysis times of 30–60 minutes per sample, and the use of hazardous reagents such as tin(ii) chloride, bromine monochloride, and aqua regia.^267–269^ These constraints severely limit real-time monitoring capabilities and point-of-use testing applications, particularly in developing regions where mercury contamination from artisanal gold mining poses the greatest public health risk. Mercury presents several unique analytical challenges that distinguish it from other heavy metals and complicate detection efforts. The exceptionally high affinity of Hg^2+^ for sulfur-containing functional groups results in the formation of extremely stable mercury–thiol complexes with stability constants (log *K*) exceeding 20, creating both opportunities for selective binding in sensor design and challenges from matrix interference by endogenous biological thiols such as GSH, cysteine, and metallothioneins. Speciation analysis represents a critical but often overlooked requirement, as total mercury measurements provide insufficient information for accurate risk assessment; the ability to distinguish toxic methylmercury (CH_3_Hg^+^) from less hazardous inorganic mercury (Hg^2+^) is essential for proper exposure evaluation, yet few sensors address this challenge.

QD-based fluorescence sensors have emerged as powerful analytical tools for Hg^2+^ detection, offering distinct advantages such as exceptional sensitivity, rapid response times typically ranging from 1–5 minutes compared to 30–60 minutes required by conventional methods, and compatibility with portable and smartphone-integrated sensing formats. The soft Lewis acid character of Hg^2+^ makes it particularly amenable to detection by chalcogenide QDs, where it interacts strongly with surface chalcogen atoms including sulfur, selenium, and tellurium through favorable soft–soft acid–base interactions. Thiol-functionalized QD surfaces exploit the Hg–S affinity to enable sub-nanomolar LODs, often achieving sensitivities well below regulatory thresholds. However, selective discrimination of Hg^2+^ from chemically similar soft metal ions such as Ag^+^, Pb^2+^, and Cd^2+^ remains non-trivial, as these ions can also interact with chalcogenide surfaces. Consequently, ratiometric fluorescence strategies that incorporate internal signal referencing have gained increasing attention due to their ability to mitigate environmental fluctuations, probe instability, and matrix-dependent signal variations.^270^

Tang *et al.* developed a dual-mode ratiometric fluorescence platform using amino-functionalized NH_2_-MIL-101(Fe) MOF as a stable blue reference (433 nm, unaffected by analyte) and 3-MPA-capped CdTe QDs as the Hg^2+^-responsive green component, quenched *via* surface metal-sulfide formation.^270^ The platform supports both down-conversion (330 nm) and up-conversion (700 nm) excitation, with up-conversion mode suppressing background autofluorescence and scattering in complex matrices. The sensor achieved an ultralow LOD of 0.053 nM across a 0.12 to 1222 nM linear range, with negligible cross-reactivity from Pb^2+^ (response identical to blank), a useful selectivity margin given the frequent co-occurrence of Hg^2+^ and Pb^2+^ in environmental samples (Fig. 14). Field deployment used a smartphone-assisted 3D dark box for RGB-based visual quantification, with a response time of approximately 2 minutes. Recovery in lake water, human urine, and food matrices ranged 98.1 to 105.3%. Signal recovery for reuse required a longer 10-minute incubation.

**Fig. 14:**
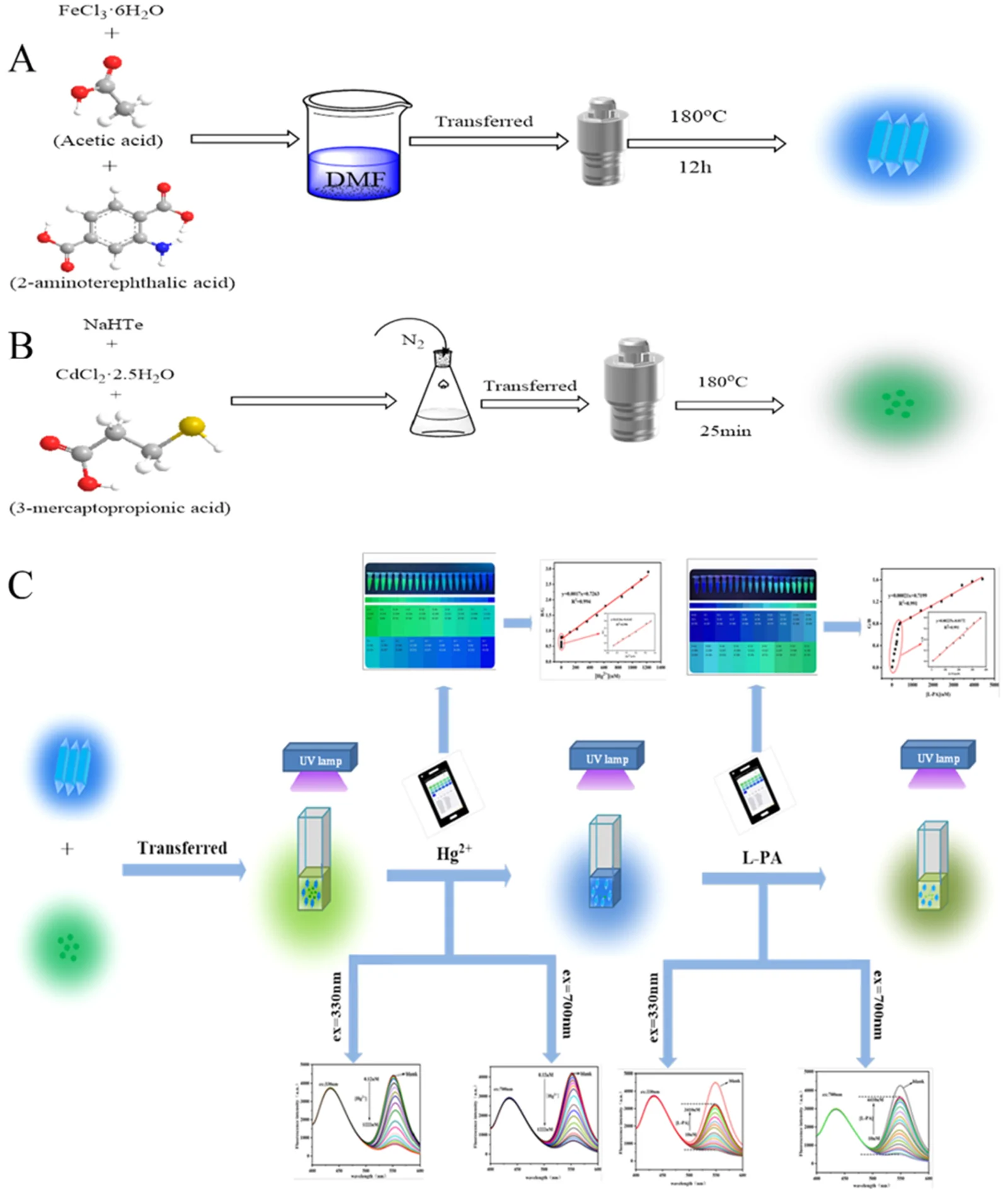
Schematic illustration of the synthesis routes for the metal–organic framework (A) and CdTe QDs (B), together with the smartphone-based RGB signal analysis for Hg^2+^ and l-penicillamine (l-PA). The scheme also depicts the fluorescence quenching and recovery mechanisms under both down-conversion and up-conversion excitation modes (C).^270^

Guo *et al.* developed a ratiometric dual-emission system combining graphitic carbon nitride nanoparticles (CNNPs, green reference emission) with red-emissive CdTe_0.16_S_0.84_ QDs (one-pot solvothermal synthesis) for ultrasensitive Hg^2+^ detection.^261^ Hg^2+^ selectively quenches the QD emission *via* combined electron transfer and electrostatic interactions, while the CNNP reference signal remains unchanged, compensating for environmental and background variability (Fig. 15). The platform achieved an exceptionally low LOD of 0.009 nM across a 0.05 to 2525 nM linear range, with negligible response to Pb^2+^, an advantage for analysis of samples containing multiple co-occurring metal ions. For field deployment, the probe was immobilized in a sodium alginate hydrogel and paired with a smartphone-based 3D dark box for RGB quantification, achieving an even lower LOD of 0.001 nM in this format. Visual detection accuracy may be affected by matrix complexity in real samples such as lake water.

**Fig. 15:**
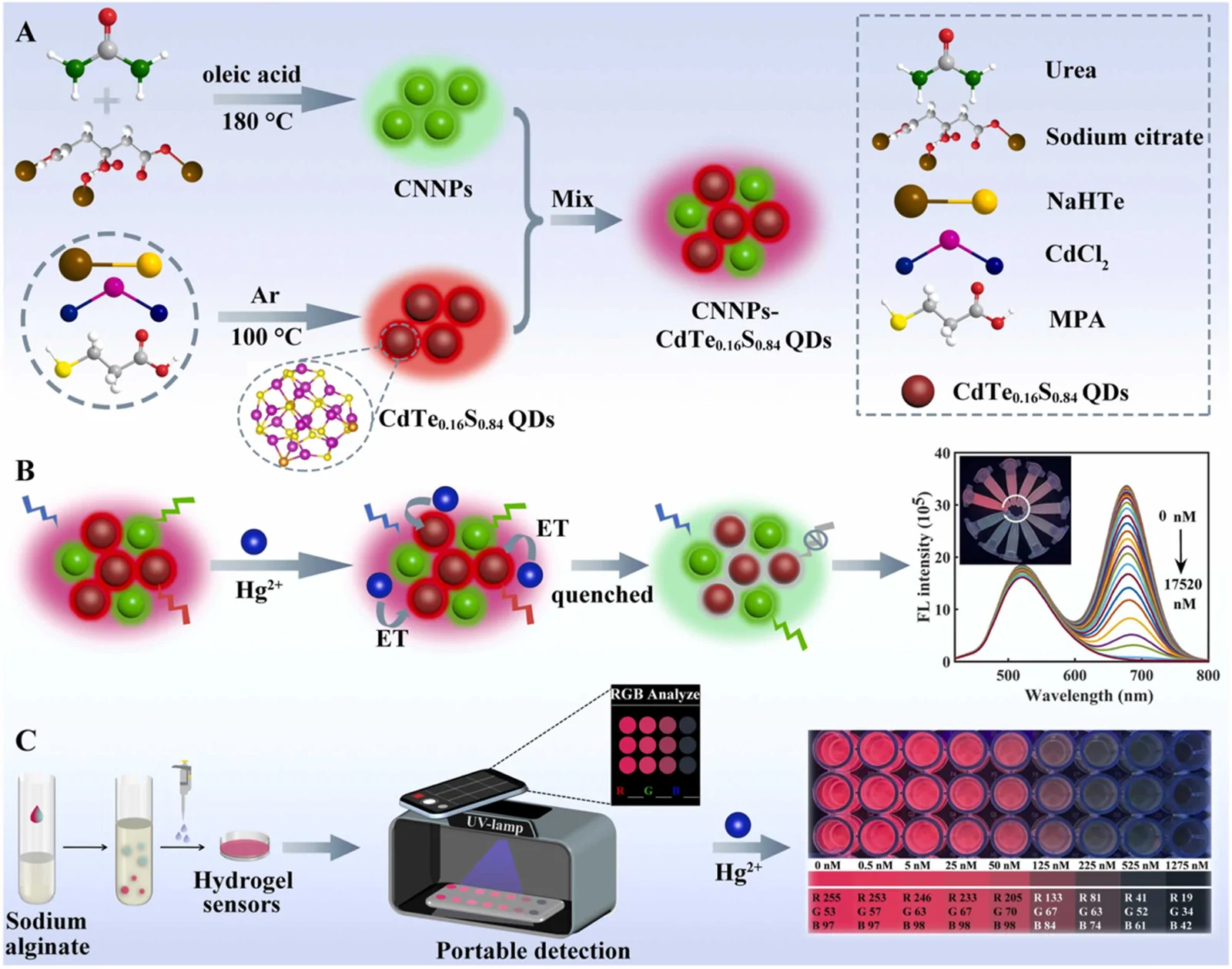
(A) Schematic representation of the synthetic route used to prepare the CNNPs-CdTe_0.16_S_0.84_ QD nanocomposite. (B) Illustration of the electron transfer-driven fluorescence quenching mechanism of the CNNPs-CdTe_0.16_S_0.84_ system in the presence of Hg^2+^ ions. (C) Schematic depiction of the portable, hydrogel-based sensing platform for on-site Hg^2+^ detection.^261^

Wang *et al.* developed a dual-emission ratiometric system combining silica-encapsulated CDs (CQDs@SiO_2_, blue reference at 450 nm) with d-penicillamine-stabilized copper nanoclusters (DPA-CuNCs, orange-red analyte-responsive emission at 650 nm), both excited at 350 nm, for Hg^2+^ detection. The *I*_650_/*I*_450_ ratio remained unchanged in the presence of Pb^2+^ up to 100 µM, confirming selectivity that arises from mercury's specific interaction with the CuNC surface groups. The sensor achieved a LOD of 1 µM across a 0 to 5 µM linear range, with a visually distinguishable purplish-red to blue color shift under 365 nm UV illumination as Hg^2+^ concentration increases (Fig. 16). Recovery in lake and tap water ranged 93.40 to 106.40%. Operational stability is optimized for alkaline conditions (pH 6 to 14) and temperatures of 10 to 50 °C, potentially limiting performance in acidic industrial effluents or extreme temperatures.^271^

**Fig. 16:**
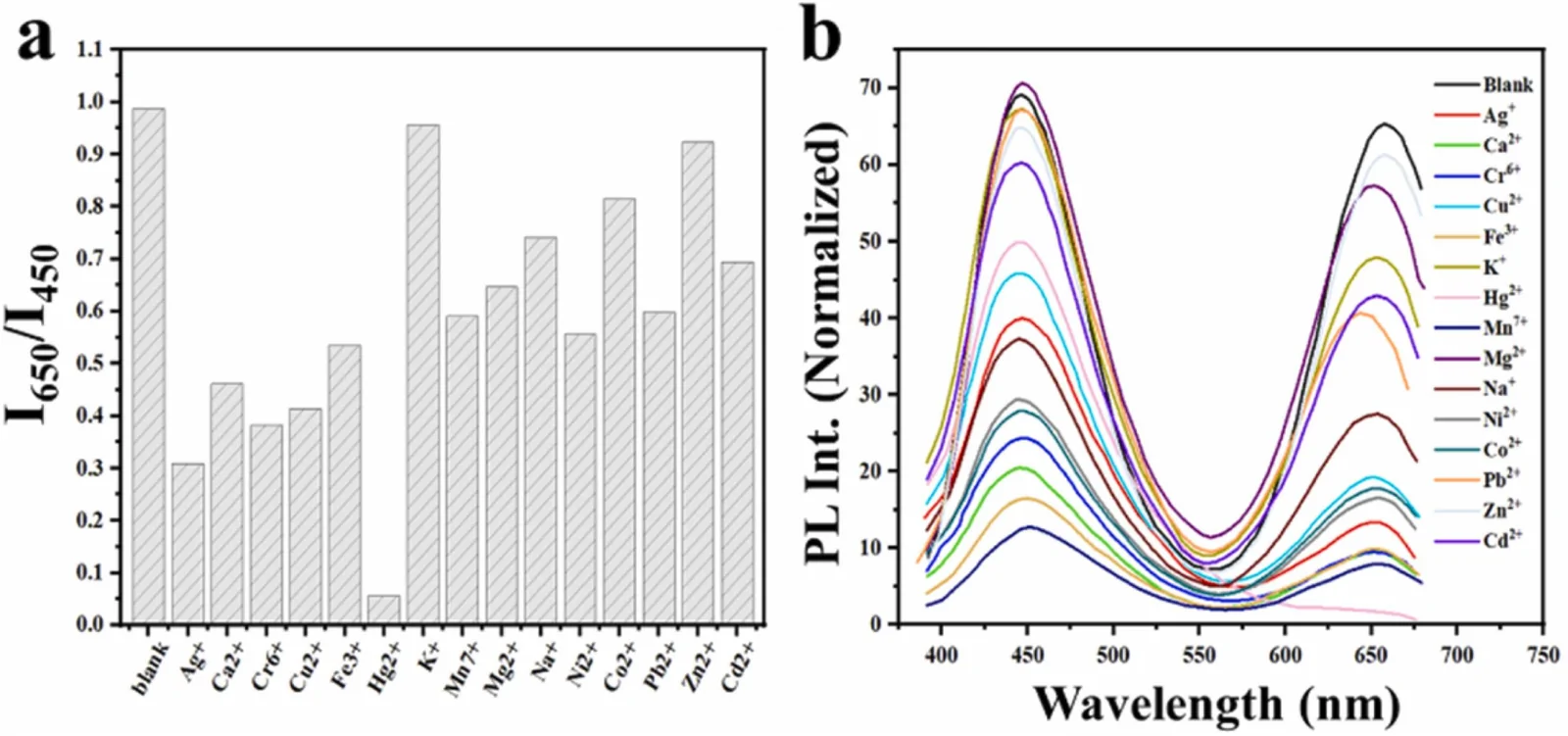
(a) Fluorescence intensity ratio responses and (b) corresponding emission spectra of CQDs@SiO_2_/DPA-CuNCs dual-emission nanocomposites in the presence of various metal ions (Ag^+^, Ca^2+^, Cr^6+^, Cu^2+^, Fe^3+^, Hg^2+^, K^+^, Na^+^, Co^2+^, Mg^2+^, Mn^7+^, Ni^+^, Pb^2+^, Zn^2+^, and Cd^2+^).^271^

Chaghaghazardi *et al.* developed nitrogen- and sulfur-*co*-doped CDs (N,S-CQDs) *via* one-pot hydrothermal synthesis (chitosan, thiourea, and citric acid precursors), yielding a biocompatible probe with 33.0% QY for Hg^2+^ detection *via* fluorescence quenching.^272^ Selectivity was strong against Pb^2+^, which caused negligible quenching even at elevated concentrations, an important property given the frequent co-occurrence of lead and mercury in environmental samples. The sensor achieved an ultralow LOD of approximately 4 nM across a 5 to 160 nM linear range, outperforming several previously reported carbon-based probes. Response time was approximately 5 minutes, with no photobleaching or signal decay over one hour of continuous irradiation. Recovery in lake water ranged 94 to 106%. The synthesis avoided toxic solvents, strong acids, and complex post-synthetic modification, supporting sustainability and scalability.

Zhang *et al.* developed blue-emitting silicon QDs (Si-QDs) *via* rapid microwave-assisted synthesis (trisodium citrate and DAMO precursors) for Hg^2+^ detection *via* dynamic quenching, confirmed by UV-vis absorption, fluorescence lifetime, and zeta potential measurements (Fig. 17).^126^ Pb^2+^ caused no appreciable fluorescence attenuation, confirming selectivity. Intrinsic up-conversion behavior suppressed background autofluorescence and scattering in complex matrices. Paired with smartphone-based grayscale image analysis, the portable system achieved a LOD of 3 nmol L^−1^ across a 0 to 5 µmol L^−1^ linear range, meeting international environmental monitoring requirements. Recovery in tap water, river water, and seawater ranged 80.3 to 113%, with strong robustness against Pb^2+^ interference. Response time was approximately 15 minutes.

**Fig. 17:**
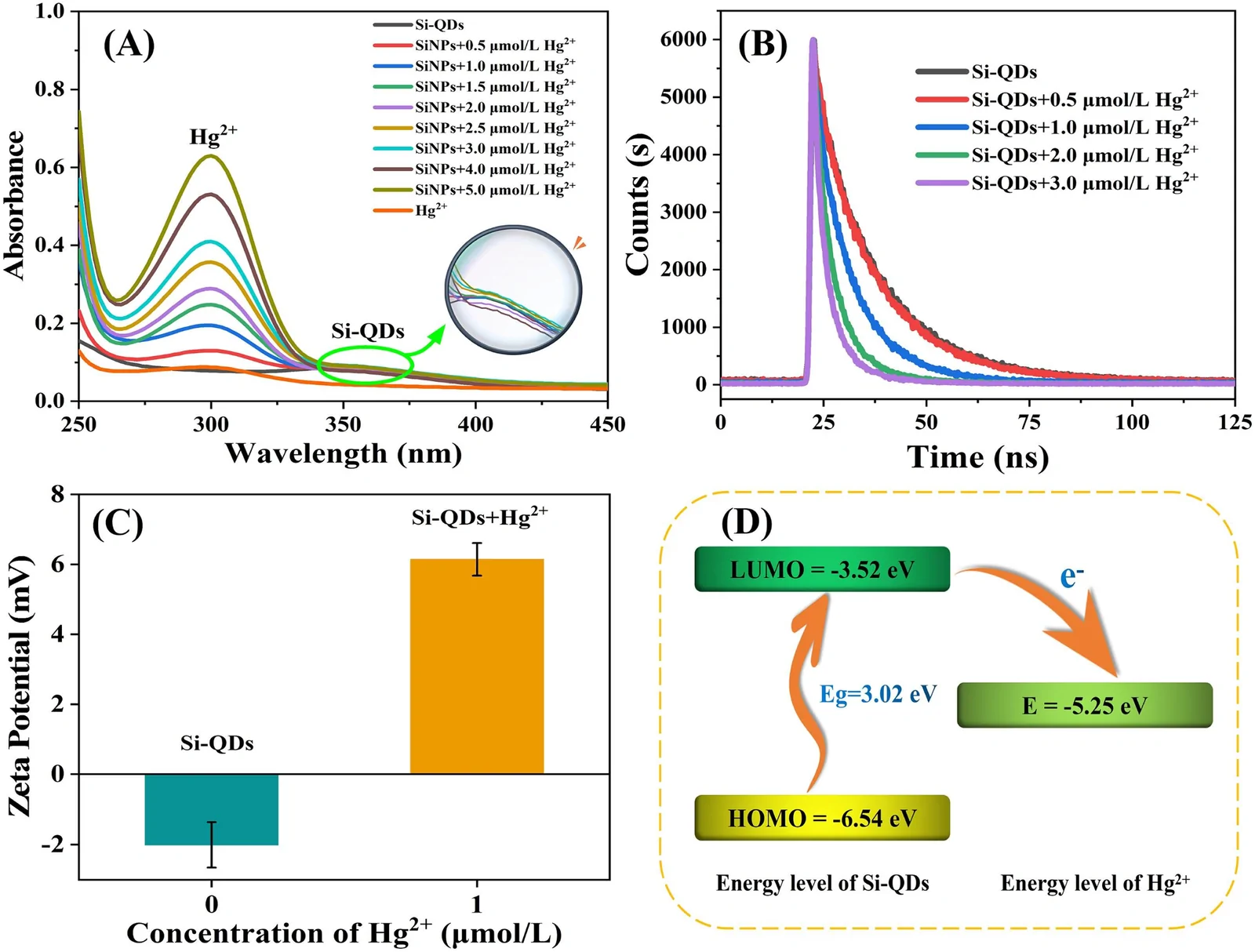
(A) UV-visible absorption spectra of Si-QDs, Hg^2+^, and the Si-QDs–Hg^2+^ system. (B) Fluorescence lifetime decay profiles of pristine Si-QDs and the Si-QDs–Hg^2+^ complex. (C) Zeta potential measurements of Si-QDs before and after interaction with Hg^2+^. (D) Schematic illustration of the proposed electron transfer-mediated quenching mechanism between Si-QDs and Hg^2+^.^126^

Han *et al.* developed a paper-based fluorescence sensor by immobilizing CdTe QDs onto amino-functionalized silica nanospheres (QDs-SiO_2_ composite, green emission at 525 nm), avoiding non-uniform distribution and coffee-ring effects common to direct QD deposition on paper, for Hg^2+^ detection *via* electron-transfer quenching.^273^ Selectivity was strong, with negligible response to Pb^2+^. The chip achieved a visual LOD of 2.83 µg L^−1^*via* smartphone grayscale analysis (0.38 µg L^−1^ by conventional fluorescence spectroscopy) across a 0 to 100 µg L^−1^ linear range, with a response time of approximately 90 seconds. Recovery in seawater, lake water, and river water ranged 96.8 to 105.4%. Dissolved organic matter in natural waters may bind free metal ions, potentially requiring optimized sample pretreatment for complex aquatic environments.

Molaei *et al.* developed thioglycolic acid (TGA)-stabilized CdTe/CdS/ZnS core/multishell QDs *via* photochemical synthesis (QY ∼45%, reduced toxicity relative to bare CdTe) for Hg^2+^ detection *via* “off-type” quenching, involving electron transfer and TGA-mediated metal-sulfide formation and aggregation (Fig. 18).^274^ Pb^2+^ produced no measurable interference even at high concentrations. The sensor achieved a LOD of 16.32 nM across a 20 to 70 nM linear range, with instantaneous response supporting real-time monitoring. For field use, QDs were immobilized on WM41 micro pads for UV-visible detection without instrumentation, validated in tap, river, and agricultural water with recovery of 98.2 to 101.3%. Stability was maintained for up to five months, though acidic conditions (pH < 6) promote aggregation and strongly alkaline conditions (pH > 9) cause structural degradation *via* metal hydroxide formation.

**Fig. 18:**
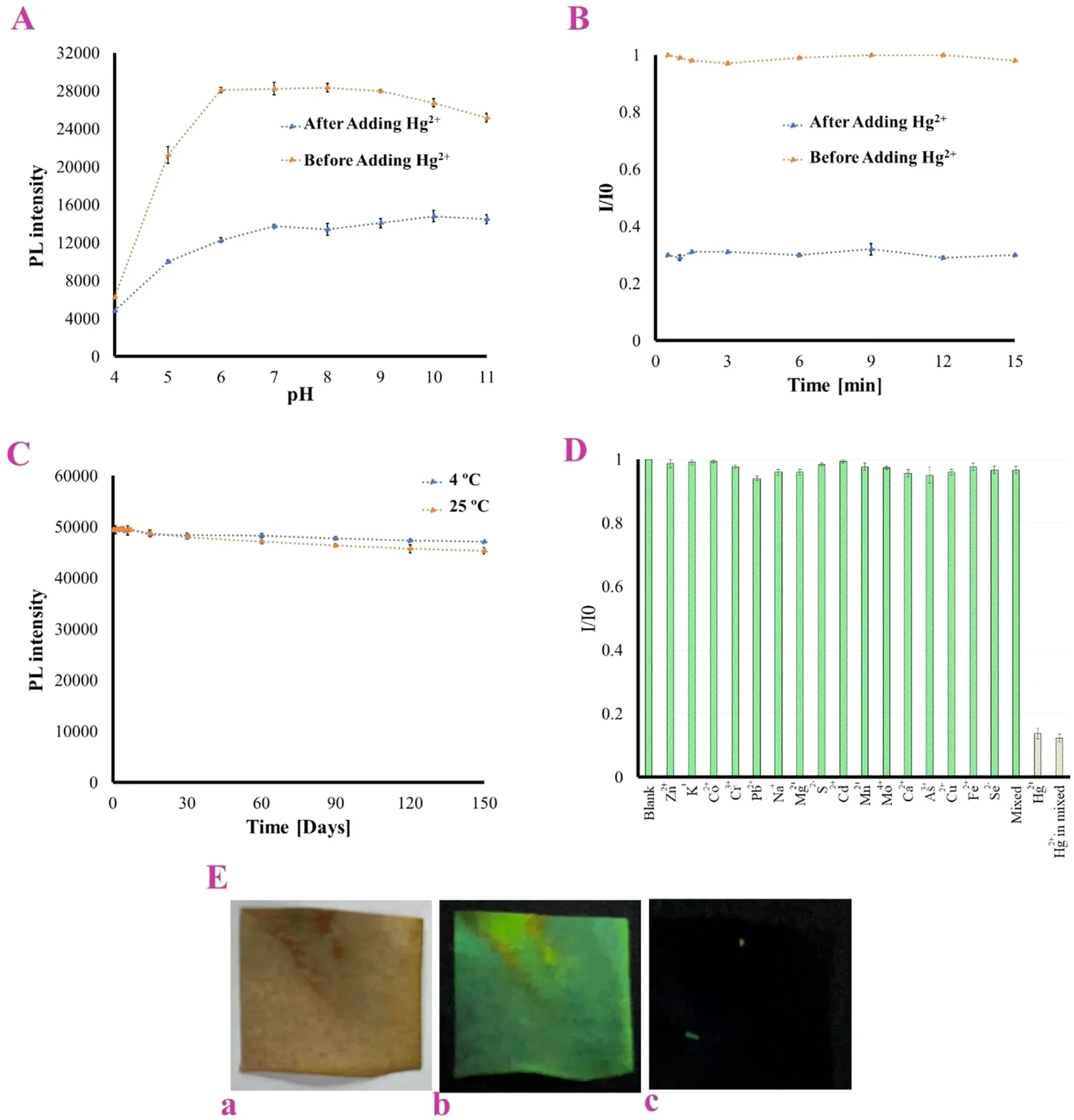
(A) Effect of pH on the fluorescence response of CdTe/CdS/ZnS QDs. (B) Response time optimization of the CdTe/CdS/ZnS QD sensor. (C) Long-term stability profile of CdTe/CdS/ZnS QDs. (D) Selectivity of the CdTe/CdS/ZnS QD-based sensor toward various metal ions. (E) Immobilization of CdTe/CdS/ZnS QDs on WM41 micro pads: (a) QD-immobilized WM41 under ambient light, (b) QD-immobilized WM41 under UV illumination, and (c) fluorescence quenching of the QD-immobilized WM41 after exposure to Hg^2+^ solution (20 µM) under UV light.^274^

Nair *et al.* developed silanized CdSe QDs *via* reverse microemulsion synthesis, coated with an ultrathin (∼1.5 nm) mesoporous silica shell that enables metal-ion diffusion to the core while enhancing colloidal stability, for Hg^2+^ detection.^275^ The fluorescence “off” response is accompanied by a bathochromic absorption/emission shift, arising from surface metal-selenide formation that alters band structure and promotes nonradiative recombination. Pb^2+^ caused negligible interference even at 100 µM, an important property for complex environmental matrices where lead commonly co-occurs. The sensor achieved a LOD of 1.5 ppm with naked-eye detection under UV illumination on a glass slide, requiring no biomolecule or polymer functionalization (label-free). The platform maintained excellent shelf stability, supporting portable, cost-effective monitoring.

Haque *et al.* developed biogenic CdSe-HSA core and CdSe@ZnSe-HSA core–shell QDs using human serum albumin as a biocompatible surface-engineering agent, yielding ultrasmall (2 to 3.5 nm) nanocrystals stable for up to 150 days; the ZnSe shell raised QY to 44.48%.^276^ Sensing relies on dynamic quenching *via* metal-ion affinity for HSA thiol groups and QD-surface selenium sites (Fig. 19), for Hg^2+^ detection. Pb^2+^ produced no measurable fluorescence change. The sensor achieved a LOD of 1.3 nM across a 0 to 2 µM linear range, with label-free, naked-eye UV detection. Validation in artificial cerebrospinal fluid gave recoveries of 102 to 109%. The synthesis avoided hazardous reducing agents like sodium borohydride. Optimal stability occurs at alkaline pH (∼9.0); acidic conditions (pH 3.0 to 4.0) degrade the protein coating or QD core.

**Fig. 19:**
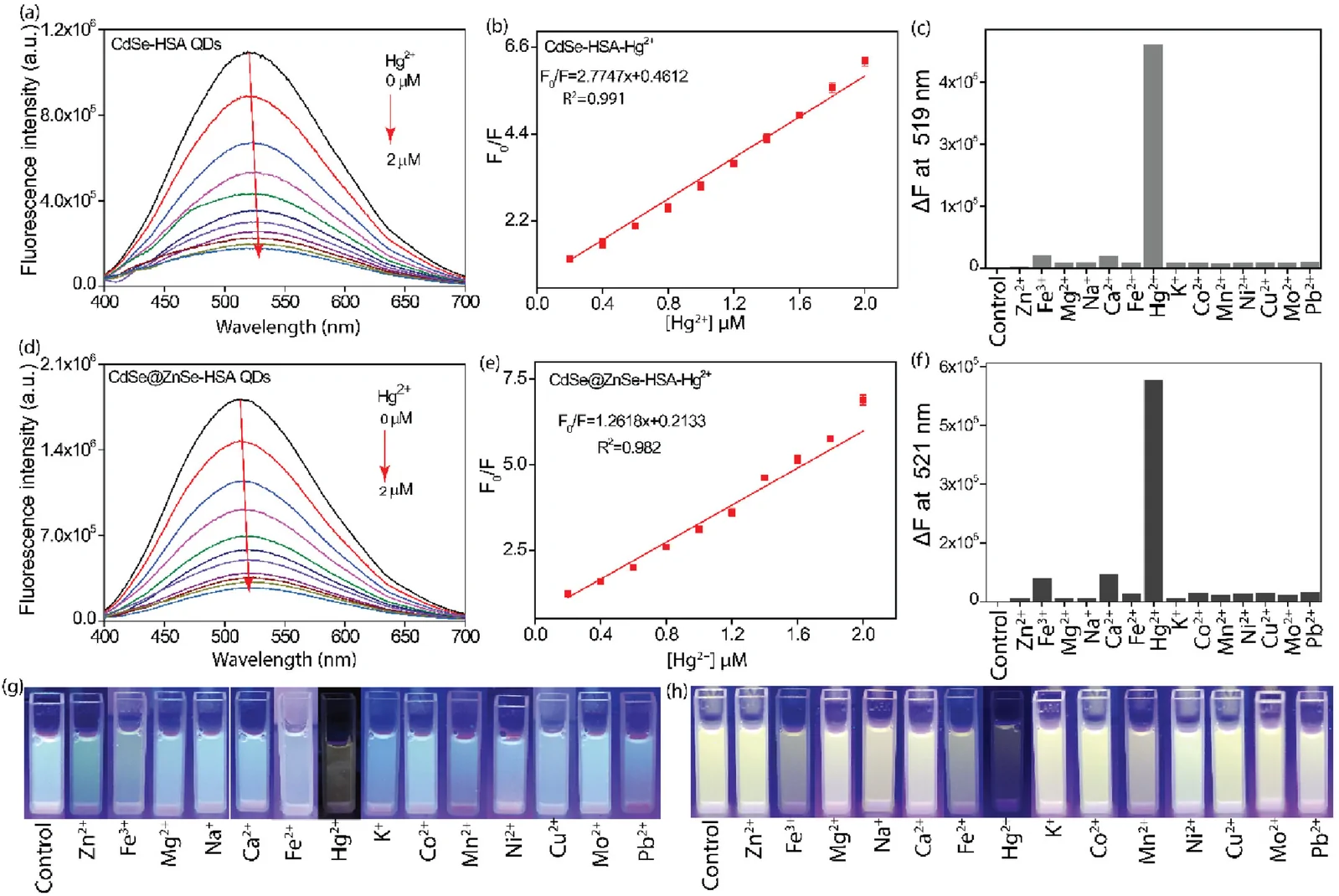
(a) Fluorescence quenching behavior of CdSe-HSA QDs (25 µM) in the absence and presence of Hg^2+^ (0–5 µM). (b) Linear calibration plot for Hg^2+^ detection using CdSe-HSA QDs (0–2 µM). (c) Selectivity evaluation of CdSe-HSA toward Hg^2+^ in the presence of competing metal ions ([metal ions] = 2 µM). (d) Fluorescence quenching profiles of CdSe@ZnSe-HSA QDs in response to Hg^2+^ (0–2 µM). (e) Corresponding linear calibration plot for CdSe@ZnSe-HSA QDs. (f) Selectivity of CdSe@ZnSe-HSA QDs toward Hg^2+^ in the presence of various metal ions ([metal ions] = 2 µM). (g and h) Naked-eye fluorescence images of CdSe-HSA and CdSe@ZnSe-HSA QDs, respectively, showing selective fluorescence quenching in the presence of Hg^2+^ under UV illumination (*λ*_ex_ = 365 nm; [QDs] : [metal ions] = 25 : 2).^276^

#### 5.2.1. Critical analysis

The analytical performance and real-world applicability of diverse nanomaterial-based sensing platforms, including ratiometric hydrogels, paper-based devices, and biogenic core–shell nanostructures, are systematically summarized in Table 15, highlighting substantial improvements in sensitivity (with LODs reaching as low as 0.001 nM) and a clear shift toward smartphone-assisted, on-site analytical methodologies. The rapid expansion of QD-based mercury sensing platforms reflects both the urgency of Hg^2+^ monitoring and the intrinsic suitability of nanostructured fluorophores for soft-metal recognition. However, beyond incremental improvements in LODs and response times, a deeper examination of the field reveals that many reported advances remain constrained by fundamental physicochemical trade-offs that have yet to be systematically addressed. The central challenge is no longer achieving sensitivity, but rather reconciling selectivity, robustness, mechanistic transparency, and sustainability within a single deployable sensing architecture. A defining characteristic of Hg^2+^ sensing is the metal's extraordinarily strong affinity for sulfur- and selenium-containing ligands, a feature that underpins the success of chalcogenide QDs while simultaneously introducing critical vulnerabilities. In controlled environments, Hg–S and Hg–Se interactions yield highly efficient fluorescence quenching through surface reconstruction, exciton trapping, or NP aggregation. In realistic matrices, however, this same affinity renders Hg^2+^ highly susceptible to sequestration by dissolved organic sulfur species, proteins, and thiol-rich biomolecules. As a result, many sensors that perform optimally in ultrapure or weakly buffered solutions experience attenuated or delayed responses in natural waters, biological fluids, or food samples. The frequent reliance on spiked recovery experiments, rather than direct comparison against independently quantified mercury speciation limits confidence in sensor accuracy under field conditions. Another underexplored dimension is mercury speciation. While most reported platforms are nominally designed for Hg^2+^ detection, environmental and biological toxicity is dominated by organomercury species, particularly methylmercury. The assumption that Hg^2+^ sensing alone provides sufficient risk assessment is increasingly untenable, especially in aquatic systems where rapid biotransformation occurs. Yet, only a small fraction of QD-based sensors attempt to distinguish inorganic mercury from organomercury compounds, and even fewer address dynamic interconversion between species. Without explicit speciation capability or selective recognition strategies, current platforms risk systematic underestimation of toxic exposure despite impressive analytical figures of merit.^277,278^

**Table 15:** Comparative analysis of nanomaterial-based sensing platforms for Hg^2+^ detection

Nanomaterial/sensing platform	Synthesis method	Doping/functionalization	Detection technique	Target analyte	Sensing mechanism	Linear detection range	LOD	Selectivity/interfering ions	Optimal working conditions	Real-sample validation (recovery)	Key advantages	Reported limitations
NH_2_-MIL-101(Fe)/CdTe QDs^270^	Solvothermal	3-Mercaptopropionic acid	Dual-mode ratiometric fluorescence; smartphone	Hg^2+^ and l-penicillamine	Dynamic quenching (Hg^2+^); recovery (l-PA)	0.12–1222 nM (Hg^2+^)	0.053 nM (Hg^2+^)	16 ions; Cu^2+^/Ag^+^ interference (masked by DDTC)	pH 7.0 (PBS), 35 °C	Lake/river water, food, urine (97.4–105.3%)	Anti-autofluorescence; rapid (2 min)	Requires masking agent for Cu^2+^/Ag^+^
CNNPs/CdTe_0.16_S_0.84_ QDs^261^	Solvothermal/redox	Carboxyl functionalization; Te-doping	Hydrogel-based ratiometric; smartphone	Hg^2+^	Electron transfer & electrostatic interactions	0.05 to 2525 nM	0.009 nM	Strong selectivity; Cu^2+^/Ag^+^ resolved by MCR-ALS	pH > 7.0, 25 °C	Lake water (linear correlation validated)	High QY; simple preparation	Solutions sensitive to environment (mitigated by hydrogel)
CQDs@SiO_2_/CuNCs^271^	Microwave/assembly	Silica coating; d-penicillamine	Dual-emission ratiometric; visual	Hg^2+^	Chemical reaction with surface groups	0–5 µM	1 µM	14 metal ions; insensitive to Pb^2+^, Cu^2+^, *etc.*	pH 6–14, 10–50 °C	Tap and lake water (92.40–106.40%)	Visual transition (fuchsia to blue); stable	Unstable in acidic conditions (pH 1–5)
N,S-CQDs^272^	Facile one-pot hydrothermal	Nitrogen and sulfur co-doping	Fluorescence quenching assay	Hg^2+^	Affinity of N/S functional groups for Hg^2+^	∼5–160 nM	∼4 nM	11 ions; no quenching from Pb^2+^, Fe^2+^, *etc.*	pH 7.0 (PB), 30 °C	Lake water (94–106%)	High QY (33.0%); green synthesis	Intensity decreases with high salt (NaCl)
Silicon QDs (Si-QDs)^126^	Microwave-assisted	DAMO functionalization	Mono-fluorescence quenching; smartphone	Hg^2+^	Electron transfer (LUMO to Hg^2+^)	0.5–5.0 µmol L^−1^	3 nmol L^−1^ (smartphone)	High selectivity (28+ ions tested)	pH 7.2 (PBS), 15 min	Tap, surface, river, and seawater (80.3–113%)	Eco-friendly; low toxicity; salt resistant	Laboratory LOD (28 nM) higher than smartphone LOD
QDs-SiO_2_ paper-based chip^273^	Sol–gel/grafting	Amino-modified silica nanospheres	Visual sensing; smartphone	Hg^2+^	Electron transfer	0–100 µg L^−1^	2.83 µg L^−1^ (visual)	14 ions; optimal at 525 nm emission	HEPES buffer, pH 7.5, 90 s	Seawater, lake, river, tap water (96.8–105.4%)	Rapid (90 s); no “coffee ring” effect	Organic matter may sequester Hg^2+^
CdTe/CdS/ZnS core/multi-shell QDs^274^	Photochemical	Thioglycolic acid stabilizer	Off-fluorescence sensor; micro pad	Hg^2+^	Electron transfer and binding to TGA	20–70 nM	16.32 nM	17 ions tested; insignificant interference	pH 6.0, immediate response	Tap, river, agricultural water (98.2–101.3%)	Reduced toxicity (ZnS shell); 5-month stability	Aggregation at pH < 6; decomposition at pH > 9
Silanized CdSe QDs^275^	Reverse microemulsion	Mesoporous silica silanized (APS)	Fluorescence switch-off; visual	Hg^2+^	Formation of HgSe trap states	Not reported (quenching 0–14 µM)	1.5 ppm	15 cations tested; permanent switch-off only by Hg^2+^	pH 7.3 (PBS), instant	Raw water (visual detection)	Label-free; rapid; excellent shelf life	LOD significantly higher than other materials
CdSe@ZnSe-HSA QDs^276^	Biogenic (HSA protein-mediated)	Human serum albumin decoration	Ultrasensitive fluorescence; naked eye	Hg^2+^	Dynamic quenching *via* Se–Hg/Hg–SH affinity	0–2 µM	1.3 nM	15 ubiquitous ions (Pb^2+^, Ag^+^, *etc.*)	pH 9.0 (PB), 5 min	Artificial cerebrospinal fluid (102–109%)	Biocompatible; highly stable (150 days)	Fluorescence lost in acidic conditions (pH 3–4)

Selectivity claims, although frequently substantiated against Pb^2+^, often overlook broader competitive landscapes. Soft metal ions such as Ag^+^ and Cu^2+^ share overlapping coordination chemistry with Hg^2+^ and are ubiquitous in industrial and urban effluents. While ratiometric and dual-emission strategies improve signal reliability, they do not inherently resolve competitive binding unless coupled to chemically orthogonal recognition motifs. The prevalent use of single-ion interference testing obscures this limitation and creates a false impression of robustness. More critically, few studies report binding constants or kinetic selectivity coefficients that would allow predictive assessment of sensor behavior under mixed-ion exposure. Mechanistic ambiguity further constrains rational design. Terms such as “electron transfer”, “dynamic quenching”, or “aggregation-induced quenching” are often invoked interchangeably, despite representing fundamentally distinct physical processes with different sensitivities to temperature, concentration, and matrix composition. In many cases, fluorescence lifetime measurements are either absent or insufficiently resolved to distinguish between excited-state deactivation and ground-state complex formation. This lack of mechanistic resolution is particularly problematic in multi-component systems, such as core–shell QDs or protein-capped architectures, where multiple quenching pathways may coexist. Without quantitative mechanistic models, sensor optimization remains largely empirical, limiting reproducibility and scalability.^201,279^

From a materials perspective, the dominance of cadmium-based QDs in high-performance Hg^2+^ sensing presents an unresolved ethical and regulatory paradox. Sensors designed to detect environmental toxins increasingly rely on materials that are themselves subject to stringent regulatory restriction. Although encapsulation strategies mitigate acute toxicity, they do not eliminate long-term risks associated with degradation, disposal, or accidental release. Cadmium-free alternatives, including silicon QDs, CDs, and polymeric fluorophores, have demonstrated promising advances in matrix tolerance and biocompatibility, yet often suffer from broader emission bands, lower QYs, or reduced photostability. Bridging this performance gap represents one of the most pressing challenges for sustainable mercury sensing. Equally critical is the issue of operational longevity. Environmental monitoring demands sensors capable of maintaining calibration and signal fidelity over extended periods under fluctuating temperature, humidity, and illumination conditions. Yet, long-term stability studies remain sporadic, with most reports limited to short-term photostability under laboratory storage. Protein-based and hydrogel-embedded systems improve dispersion and handling but introduce new degradation pathways, including enzymatic breakdown, microbial growth, and pH-induced denaturation. The absence of standardized aging protocols makes it difficult to assess whether reported stability metrics translate to meaningful operational lifetimes.^280,281^

Device integration, while increasingly emphasized through smartphone-assisted platforms, introduces additional layers of complexity. Variability in camera sensors, illumination geometry, and image-processing algorithms can introduce systematic bias that rivals chemical uncertainty. Few studies report calibration transferability across devices or address the need for internal optical standards beyond ratiometric emission alone. Without such considerations, the promise of decentralized mercury monitoring risks being undermined by inconsistent readouts across users and environments. Advancing the field will require a strategic shift from sensitivity-driven innovation toward systems-level optimization. Priority areas include the development of chemically orthogonal recognition motifs capable of discriminating mercury species under competitive conditions; mechanistic validation using time-resolved and *in situ* techniques; accelerated aging and matrix stress testing as standard reporting criteria; and the intentional design of cadmium-free platforms that meet regulatory and sustainability benchmarks. Equally important is the alignment of sensor development with realistic deployment scenarios, including artisanal mining regions, coastal monitoring stations, and clinical screening contexts, each with distinct performance requirements. In summary, QD-based mercury sensing has reached a level of analytical sophistication where further progress will depend less on incremental improvements in LODs and more on resolving foundational challenges in selectivity, stability, sustainability, and translational validation. Addressing these issues holistically will determine whether the field evolves into a mature environmental monitoring technology or remains confined to laboratory demonstrations of technical elegance without practical reach.^282,283^

### 5.3. Detection of cadmium (Cd)

Cadmium (Cd) represents a particularly pressing target for environmental monitoring and biosensing applications due to its dual nature as both a widespread contaminant and a persistent bioaccumulative toxin. In recent decades, cadmium residues have emerged as a critical driver of environmental and food chain contamination, with profound implications for human health across multiple organ systems. The bioaccumulation of cadmium in agricultural crops and aquatic organisms poses significant risks to ecosystem stability and food security.^284,285^ Upon human exposure, cadmium exhibits a troubling propensity for accumulation in vital organs, particularly the kidneys and liver, where it triggers chronic pathological changes including tubular dysfunction and hepatic damage. The systemic consequences of prolonged cadmium exposure extend beyond organ-specific injury; long-term exposure compromises vascular integrity and disrupts critical physiological systems, substantially elevating carcinogenic risk across multiple tissue types. Given these multifaceted health and environmental threats, the development of sensitive, selective, and field-deployable analytical methods for quantitative detection of Cd^2+^ has become essential for both regulatory compliance and public health surveillance.^286^

Recent advances in nanomaterial-based sensing platforms have demonstrated the potential to address this analytical challenge. Polymer-based QD nanoprobes offer a compelling combination of chemical selectivity and practical robustness. Chen *et al.* developed an “OFF–ON” fluorescent nanosensor for Cd^2+^ using CdTe/CdS/SiO_2_ core/shell/shell QDs modified with 3-MPA, in which ammonium pyrrolidine dithiocarbamate (APDC) quenches fluorescence (94.06% efficiency) by disrupting the surface passivation layer.^287^ Cd^2+^'s superior affinity for the MPA sulfhydryl groups displaces APDC, restoring passivation and fluorescence; Pb^2+^ does not trigger this recovery (∼10% efficiency), giving strong discrimination against a common environmental and food-matrix interferent. The sensor achieved a LOD of 0.3451 µmol L^−1^ across two linear ranges (0 to 20 and 20 to 160 µmol L^−1^), with a 10-minute response time. The SiO_2_ shell (Stöber method) improved photostability (87.26% QY), biocompatibility, and reduced metal leakage relative to single-core structures. Visual detection under 365 nm UV was validated in seawater, freshwater, and milk, with recovery of 90.73 to 111.60%. Performance is pH-dependent (optimal at pH 7.0), with competitive interference from Hg^2+^ and Co^2+^, which form low-solubility tellurides on the QD surface.

Islam *et al.* developed a long-wavelength emissive ZnO/CD nanocomposite *via* chemical synthesis and hydrothermal treatment of citric acid, yielding a rod-like morphology (∼33 nm crystallite size) with strong yellow-orange emission (570 nm) and high color purity (94.3%), for Cd^2+^ detection.^288^ Sensing proceeds *via* static quenching, confirmed by time-resolved fluorescence, involving non-radiative electron transfer to vacant d-orbitals of the target ion and surface complex formation *via* carboxyl/hydroxyl groups on the CQD surface (Fig. 20). The sensor achieved a LOD of 0.14 µM across a 0 to 150 µM linear range, with stable fluorescence across pH 3 to 12 and ionic strength up to 2.0 M NaCl. Response time was 3 minutes. Recovery in tap and drinking water ranged 100.5 to 106%. Selectivity against divalent cations including Ni^2+^, Cu^2+^, and Mg^2+^ was addressed through surface functionalization design.

**Fig. 20:**
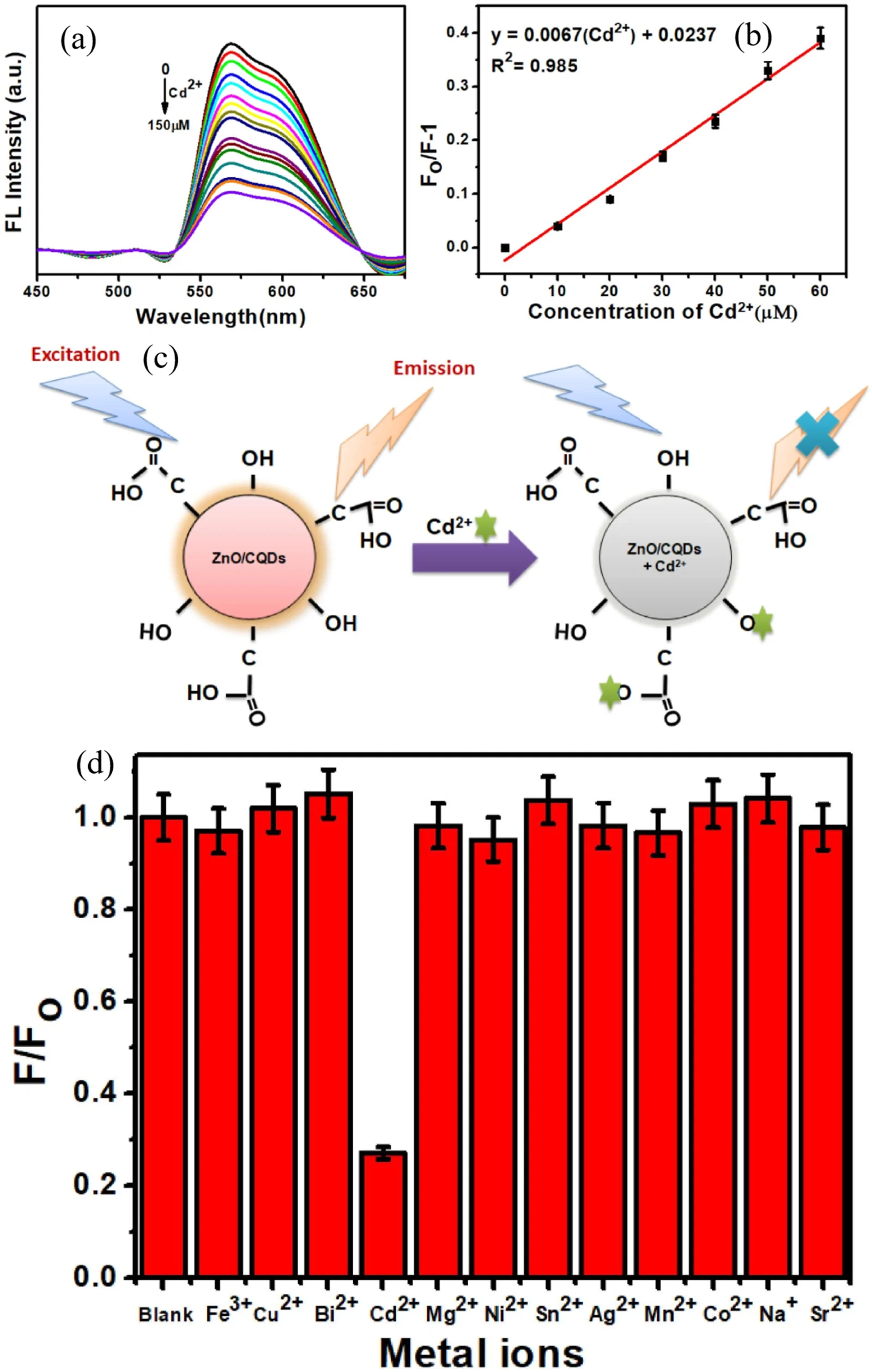
Analytical performance, selectivity, and fluorescence quenching mechanism of the ZnO/CQD sensor. (a) Fluorescence emission spectra showing progressive quenching of the yellow–orange emission upon increasing target metal ion concentration (0–150 µM). (b) Linear calibration curve of fluorescence quenching efficiency *versus* ion concentration in the range of 0–60 µM, with a correlation coefficient (*R*^2^) of 0.985. (c) Selectivity and interference study illustrating the relative fluorescence intensity (*F*/*F*_0_) of the ZnO/CQD platform in the presence of various divalent and trivalent metal ions, demonstrating high specificity toward the target analyte. (d) Schematic illustration of the proposed fluorescence quenching mechanism, where surface complex formation between the ZnO/CQD nanocomposite and the target ion induces an “OFF” state *via* non-radiative electron transfer to vacant d-orbitals of the metal ion.^288^

Karabıyık and Ebil developed a reusable, solid-state fluorescent sensor for Cd^2+^ by immobilizing a CdTe QD-4-amino TEMPO (4AT) complex onto a cross-linked p(GMA-*co*-V4D4) copolymer *via* initiated CVD, operating through an electron-shuttle/nitroxide interaction mechanism.^289^ The sensor achieved a LOD of 0.195 µM across a 0.0784 to 2.5 µM linear range under pH 8.0 (PBS buffer), with a 10-minute reaction time. Selectivity was demonstrated against 12 competing ions, including Cu^2+^, Cr^3+^, Ag^+^, Hg^2+^, Pb^2+^, and Zn^2+^. A key advantage is reusability: the iCVD polymer substrate offered high mechanical strength and could be regenerated for at least four cycles. Recovery in tap, bottled, and sea water ranged 89.6 to 98.6%. Non-uniform bonding or isolated pockets on the polymer surface were noted as a limitation.

Ai *et al.* developed a ratiometric, smartphone-integrated dual-emission probe combining blue-emitting CDs with red-emitting l-cysteine-capped, EDTA-etched CdTe QDs for Cd^2+^ detection *via* a surface defect repair (turn-on) mechanism.^290^ The sensor achieved a LOD of 0.018 µM across a 0.1 to 23 µM linear range under pH 9.0 (Tris buffer), with a 10-minute recovery time. Selectivity was demonstrated against Na^+^, K^+^, Ca^2+^, Mg^2+^, Zn^2+^, Fe^3+^, Cu^2+^, Ba^2+^, Pb^2+^, and Mn^2+^. Visual detection produced a blue-to-pink color transition under UV illumination, quantified *via* smartphone RGB analysis (Fig. 21). Recovery in rice samples ranged 84.1 to 105.4%. QD instability at acidic pH (<6) was noted as a limitation.

**Fig. 21:**
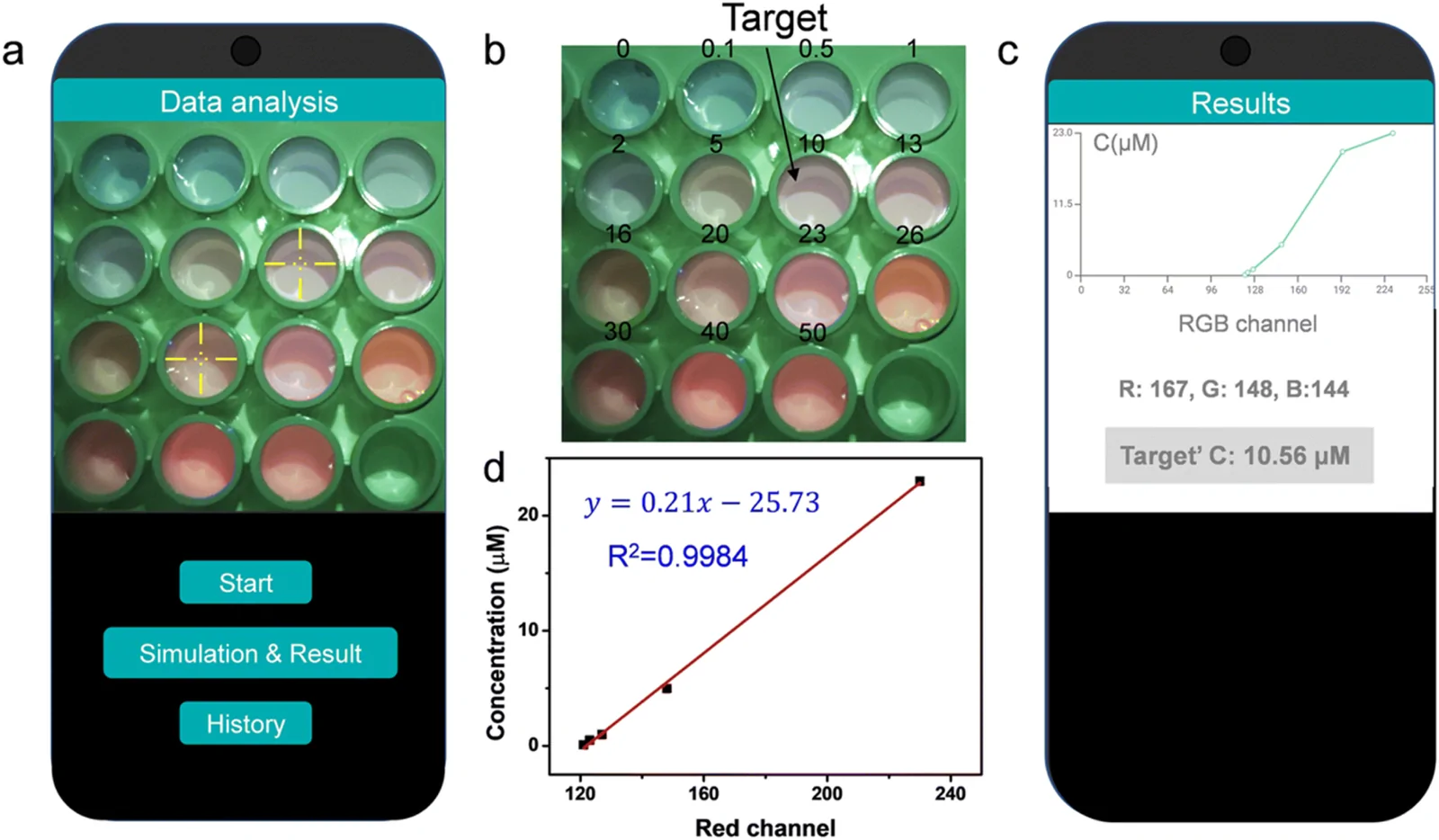
(a) Schematic illustration of the smartphone-assisted detection strategy for Cd^2+^ ions. (b) Photographs of the CQDs/CdTe QDs sensing system exposed to increasing concentrations of Cd^2+^ under 365 nm UV illumination. (c) Smartphone-based analysis showing the calculated concentrations of Cd^2+^ derived from image processing. (d) Linear calibration plot correlating Cd^2+^ concentration with the red-channel intensity over the range of 0.1–23 mM.^290^

Cao *et al.* developed a portable, paper-based fluorescent sensor for Cd^2+^ using EDTA-etched, TGA-capped CdTe@CdS QDs, operating through an “OFF–ON” surface defect repair (turn-on) mechanism, with EDTA-induced surface defects acting as selective ion-recognition sites that restore fluorescence upon target-ion recombination.^291^ The sensor achieved a LOD of 0.092 µM across a 0.44 to 6.67 µM linear range under pH 8.0 (Tris–HCl), with a 9-minute incubation and a visual cut-off concentration of 0.44 µM (Fig. 22). Selectivity was demonstrated against Fe(iii), Hg(ii), K(i), Mg(ii), Mn(ii), and Na(i). Coupled with a smartphone-based electronic-eye readout, recovery in rice samples ranged 76.2 to 104.2%. Etching times exceeding 4 minutes irreversibly damage the QD core, and excessive EDTA concentrations cause competitive chelation that inhibits target-ion recovery.

**Fig. 22:**
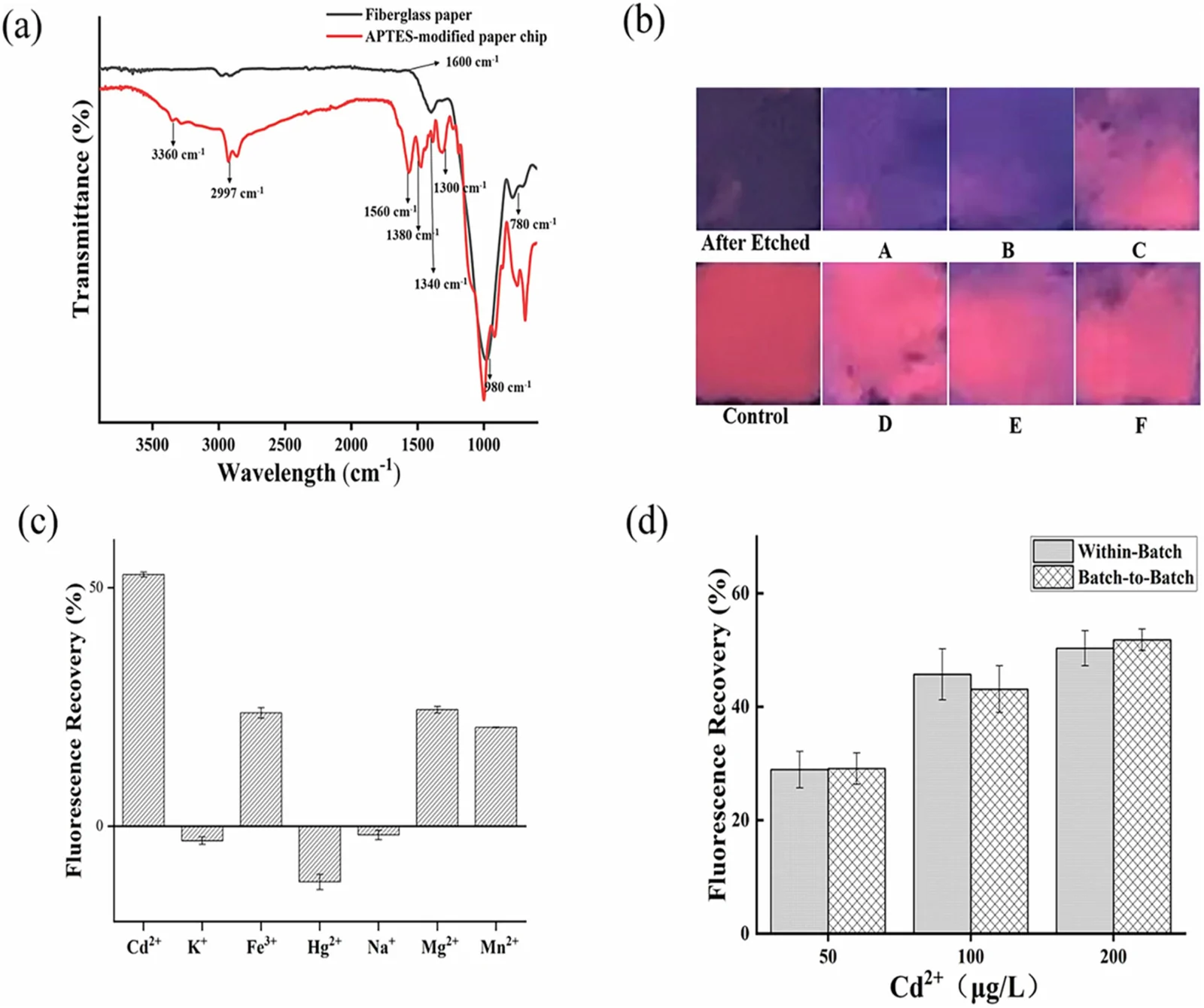
(a) FTIR spectra of pristine fiberglass paper and APTES-modified paper chips. (b) Visual determination of the cut-off concentration for Cd(ii) detection using EDTA-etched CdTe@CdS QD paper chips in rice samples, corresponding to Cd(ii) concentrations of 0 µg L^−1^ (A), 10 µg L^−1^ (B), 50 µg L^−1^ (C), 100 µg L^−1^ (D), 500 µg L^−1^ (E), and 1000 µg L^−1^ (F). (c) Selectivity evaluation of the EDTA-etched CdTe@CdS QD chips toward Cd(ii) (0.1 mg L^−1^) in the presence of competing ions, including Fe(iii), Hg(ii), K(i), Mg(ii), Mn(ii), and Na(i) (each at 5 mg L^−1^). (d) Stability assessment of the EDTA-etched CdTe@CdS QD paper-based sensor.^291^

Yan *et al.* developed nitrogen and boron co-doped CDs (N,B-CQDs) *via* one-pot hydrothermal synthesis (citric acid, 2-amino-3-hydroxypyridine, and sodium borohydride precursors), achieving a fluorescence QY of 21.2%, for Cd^2+^ detection *via* a CHEF mechanism in which Cd^2+^ coordination with surface nitrogen and oxygen groups suppresses internal charge transfer and promotes radiative recombination.^140^ The sensor achieved a LOD of 0.45 mM across a 2.5 to 22.5 mM linear range under pH 7.0 (ultrapure water), with a rapid 1-minute response. Selectivity was demonstrated against Zn^2+^, K^+^, Na^+^, Ag^+^, Ba^2+^, Mg^2+^, Mn^2+^, Ca^2+^, Cr^6+^, Co^2+^, and Cr^3+^; Pb^2+^ was not included in the interference studies, leaving potential cross-sensitivity to lead unaddressed. Recovery in tap water, lake water, and human urine ranged 97.9 to 105.9%. Fluorescence intensity fluctuates significantly across pH 2.0 to 11.0 due to protonation/deprotonation of surface amide groups.

Hong *et al.* developed a DNA aptamer-based fluorescence “turn-on” sensor for Cd^2+^, using a Cy5-labeled Cd^2+^-specific aptamer hybridized to an Iowa Black-quenched complementary strand; Cd^2+^ coordination triggers a conformational change that disrupts hybridization and restores fluorescence.^292^ The sensor achieved a LOD of 8.36 nM across a 0 to 5 µM linear range under pH 7.4 (Tris–HAc buffer) at 37 °C, with signal stabilization within approximately 20 minutes. Selectivity was demonstrated against Na^+^, K^+^, Li^+^, Zn^2+^, Mg^2+^, Mn^2+^, Ca^2+^, and Ce^3+^; Pb^2+^ was not evaluated, leaving its effect on the quenched state unresolved. The platform enabled real-time spatiotemporal monitoring of QD ion leaching in living HeLa cells, distinguishing CdTe QD leaching behavior from more stable core–shell CdSe/CdS QDs, and performance was noted as comparable to ICP-MS. Rigorous probe purification is required, and non-specific signal interference is possible in complex intracellular environments.

#### 5.3.1. Critical analysis

Despite sustained interest in QD-based fluorescent sensors for cadmium (Cd^2+^) detection, driven by the metal's widespread environmental occurrence and its well-documented nephrotoxic and carcinogenic effects, the field remains largely confined to proof-of-concept demonstrations. Translation into robust, deployable analytical technologies has been minimal. As summarized in Table 16, sensing platforms reported between 2019 and 2026 frequently claim impressive analytical performance, including nanomolar to low-micromolar LODs and apparent selectivity against common interferents. However, closer examination reveals persistent scientific ambiguities, insufficient validation in realistic matrices, and a fundamental disconnect between laboratory-scale performance and real-world monitoring requirements. A recurring pattern in the literature is the emphasis on ultra-low LODs, often between 8 and 200 nM, that are obtained exclusively under idealized conditions, typically in buffered aqueous solutions with minimal matrix complexity. For example, Hong *et al.* reported an LOD of 8.36 nM using a Cy5-labeled DNA aptamer sensor,^292^ while Ai *et al.* achieved 0.018 µM with a ratiometric CQDs/CdTe probe.^290^ In most cases, however, these values represent instrumental LODs rather than true method LODs that reflect performance in environmentally relevant samples. When applied to real matrices such as rice extracts, seawater, or drinking water, reported recovery ranges (76.2–111.6%) indicate substantial matrix effects that are not captured by spiked LOD determinations. This limitation is particularly concerning given that regulatory thresholds for Cd^2+^, approximately 27 nM in drinking water under WHO guidelines and 0.3–3 mg kg^−1^ in agricultural soils, require not only sensitivity but also quantitative accuracy in the presence of dissolved organic matter, humic substances, competing metal ions, and variable ionic strength. Notably, Islam *et al.* reported a comparatively higher LOD (0.14 µM) for their ZnO/CQDs nanocomposite sensor, yet achieved excellent recoveries in real water samples, suggesting that analytical robustness may be more valuable than nominal sensitivity alone.^288^ Critically, none of the studies summarized in Table 16 provide systematic evaluations of LOD degradation when transitioning from buffered systems to authentic environmental matrices, nor do they employ matrix-matched calibration, standard-addition validation, or benchmarking against established techniques such as ICP-MS or AAS.

**Table 16:** Comparative performance of QD-based platforms for Cd^2+^

Nanomaterial/sensing platform	Synthesis method	Doping/functionalization	Detection technique	Target analyte	Sensing mechanism	Linear detection range	LOD	Selectivity/interfering ions	Optimal working conditions	Real-sample validation (recovery)	Key advantages	Reported limitations
EDTA-etched CdTe@CdS QDs^291^	Hydrothermal (autoclave)	TGA-capped; EDTA-etched	Fluorescence (electronic eye/paper chip)	Cd(ii)	Surface defect repair (turn-on)	0.44–6.67 µM	0.092 µM	Fe(iii), Hg(ii), K(i), Mg(ii), Mn(ii), Na(i)	pH 8.0 (Tris–HCl); 9 min incubation	Rice (76.2–104.2%)	On-site visual detection; paper-based portability	Etching >4 min damages QDs; high EDTA inhibits recovery
Cy5-labeled DNA aptamer sensor^292^	Hybridization	Cy5 (fluorophore); Iowa Black (quencher)	Fluorescence	Cd^2+^	Conformational change/dehybridization (turn-on)	0–5 µM	8.36 nM	Na^+^, K^+^, Li^+^, Zn^2+^, Mg^2+^, Mn^2+^, Ca^2+^, Ce^3+^	pH 7.4 (Tris-HAc); 37 °C	HeLa cells (live imaging)	Spatiotemporal monitoring in living cells; comparable to ICP-MS	Requires careful probe purification; potential intracellular instability
ZnO/CQDs nanocomposite^288^	Chemical route (ZnO); hydrothermal (CQDs)	Oxygen-rich functional groups (carboxyl/hydroxyl)	Fluorescence (phosphor)	Cd^2+^	Static quenching/non-radiative electron transfer	0–150 µM (linear 0–60 µM)	0.14 µM	Fe^3+^, Cu^2+^, Bi^2+^, Mg^2+^, Ni^2+^, Sn^2+^, Ag^2+^, Mn^2+^, Co^2+^, Na^+^, Sr^2+^	pH 7.0 (Na–Ac); 3 min incubation	Tap and drinking water (100.5–106%)	High color purity (94.3%); rapid response	Emission restricted to long-wavelength yellow-orange zone
APDC-etched CdTe/CdS/SiO_2_ QDs^287^	Stöber method	MPA-capped; APDC-etched	Fluorescence (OFF–ON)	Cd^2+^	Surface passivation recovery (turn-on)	0–20 µmol L^−1^ and 20–160 µmol L^−1^	0.3451 µmol L^−1^	15 metal ions (K^+^, Na^+^, Ba^2+^, Pb^2+^, Zn^2+^, *etc.*)	pH 7.0 (PBS); 10 min reaction	Seawater, freshwater, milk (90.73–111.60%)	SiO_2_ shell improves stability and reduces metal leakage	Hg^2+^ and Co^2+^ interfere *via* accumulation on surface
N,B-CQDs (nitrogen/boron co-doped)^140^	One-pot hydrothermal	N and B co-doping	Fluorescence (on–off)	Cd^2+^ and l-cysteine	CHEF	2.5 mM to 22.5 mM	0.45 mM	Zn^2+^, K^+^, Na^+^, Ag^+^, Ba^2+^, Mg^2+^, Mn^2+^, Ca^2+^, Cr^6+^, Co^2+^, Cr^3+^	pH 7.0 (ultrapure water); 1 min incubation	Tap water, lake water, human urine (97.9–105.9%)	High QY (21.2%); extremely rapid 1 min response	Significant pH sensitivity (pH 2.0–11.0 fluctuation)
CQDs/CdTe QDs ratiometric probe^290^	Hydrothermal/autoclave	l-Cysteine capped; EDTA-etched	Ratiometric fluorescence (smartphone)	Cd^2+^	Surface defect repair (turn-on)	0.1 µM to 23 µM	0.018 µM	Na^+^, K^+^, Ca^2+^, Mg^2+^, Zn^2+^, Fe^3+^, Cu^2+^, Ba^2+^, Pb^2+^, Mn^2+^	pH 9.0 (Tris buffer); 10 min recovery	Rice samples (84.1–105.4%)	Visual blue-to-pink color change; smartphone integration	QD instability at acidic pH (<6)
CdTe QD-4AT on p(GMA-*co*-V4D4) polymer^289^	iCVD (polymer); hydrothermal (QD)	4-Amino TEMPO (4AT) ligand	Solid-state fluorescence	Cd^2+^	Electron shuttle/nitroxide interaction	0.0784–2.5 µM	0.195 µM	12 ions (Cu^2+^, Cr^3+^, Ag^+^, Hg^2+^, Pb^2+^, Zn^2+^, *etc.*)	pH 8.0 (PBS buffer); 10 min reaction	Tap, bottled, sea water (89.6–98.6%)	Reusable (4+ cycles); high mechanical strength	Non-uniform bonding/isolated pockets on surface

Selectivity claims further highlight the gap between laboratory testing and real-world applicability. Although many sensors are reported to tolerate 8–15 common ions, selectivity is typically evaluated using single-ion challenges or binary mixtures with Cd^2+^ at equimolar or modest excess concentrations. Such testing protocols fail to reflect the complexity of natural waters, where multiple metal ions coexist at widely varying concentration ratios. Yan *et al.*, for instance, evaluated their N,B-CQDs sensor against individual interferents but did not assess simultaneous multi-ion exposure or competitive binding effects.^288^ Recurrent interference from Cu^2+^ and Hg^2+^ exposes deeper limitations rooted in coordination chemistry, as these ions often outcompete Cd^2+^ due to higher Lewis acidity and favorable positioning within the Irving–Williams series. Similarly, Fe^3+^-induced nonspecific quenching is frequently acknowledged despite Fe^3+^ concentrations in groundwater often exceeding those of Cd^2+^ by several orders of magnitude. While HSAB theory is commonly invoked to rationalize selectivity, it fails to account for critical factors such as ligand accessibility, coordination geometry, hydration effects, and kinetic competition. Notably, none of the reviewed studies employ chemometric or multivariate analytical approaches to resolve overlapping spectral responses, methods that could potentially transform selectivity limitations into multi-analyte sensing capabilities.

Mechanistic interpretation represents another major source of uncertainty. Across the literature, diverse sensing mechanisms are proposed, including static or dynamic quenching, surface passivation, CHEF, electron transfer, and defect-mediated emission recovery. However, experimental evidence supporting these assignments is often circumstantial. Rigorous mechanistic validation through temperature-dependent Stern–Volmer analysis, time-resolved fluorescence spectroscopy, or complementary absorption studies is rarely performed. In etching–repair-based systems, such as those employing EDTA or APDC, fluorescence recovery is attributed to selective passivation of surface defects by Cd^2+^, yet the chemical basis for this selectivity, particularly over structurally similar ions such as Zn^2+^, remains poorly justified. The absence of direct structural characterization using techniques such as XPS, extended X-ray absorption fine structure (EXAFS), or nuclear magnetic resonance (NMR) leaves the molecular origins of selectivity largely speculative. This issue is especially pronounced in doped or ternary QD systems, where the relative contributions of dopant-induced emissive states, intrinsic defects, and surface complexation remain unresolved, severely limiting rational sensor optimization.

Operational stability and reproducibility further constrain translational potential. Many QD-based Cd^2+^ sensors function only within narrow pH windows, typically between pH 7 and 9, excluding acidic environments commonly encountered in mining-impacted or industrial regions. Several studies explicitly report fluorescence instability at lower pH values but do not propose mitigation strategies. Photostability is another critical yet largely unaddressed issue, despite the inevitability of continuous excitation in fluorescence-based sensing. Claims of reusability or long-term stability are rarely supported by quantitative degradation metrics, operational lifetime assessments, or shelf-life studies. Equally concerning is the lack of batch-to-batch reproducibility data. Hydrothermal synthesis methods are highly sensitive to reaction parameters, yet no study reports statistical process control, interlaboratory validation, or use of certified reference materials. Narrow processing windows, such as the strict etching-time dependence reported by Cao *et al.*, further complicate scale-up and reproducibility.^291^

A fundamental regulatory contradiction also pervades the field. Many sensors designed to detect cadmium rely on cadmium-based QDs themselves, creating an inherent conflict with environmental and health regulations such as RoHS and REACH. While encapsulation strategies have been proposed to mitigate metal leakage, long-term shell integrity under environmental stressors remains unvalidated. Cadmium-free alternatives, including CDs, InP/ZnS, and AgInS_2_ systems, offer promising pathways forward but currently lag in QY, surface chemistry control, and mechanistic understanding. Importantly, none of the reviewed studies conduct life-cycle assessments, leaching studies, or environmental fate modelling, representing a significant barrier to regulatory approval and public acceptance. Manufacturing and scalability challenges further limit translational viability. The synthesis routes commonly employed, hydrothermal autoclaving, batch one-pot reactions, and post-synthetic functionalization, are inherently ill-suited for industrial-scale production. These methods suffer from poor parameter control and high batch-to-batch variability, while post-synthesis modification steps introduce additional sensitivity to timing and reagent concentration. Continuous-flow or microfluidic synthesis approaches, which could dramatically improve reproducibility and scalability, remain largely unexplored. Moreover, economic considerations, including cost per test and comparison with existing analytical technologies, are almost entirely absent from the literature.^112,293^ Finally, the lack of standardized performance metrics and system-level integration severely hampers progress toward field deployment. Definitions of stability, selectivity, and sensitivity vary widely across studies, making inter-comparison unreliable. Device integration efforts, such as paper-based platforms or smartphone readouts, represent promising steps but remain insufficiently robust for deployment by non-specialist users. Most systems lack calibration stability, environmental durability, and user-centered design. The scarcity of QD-based sensors integrated into microfluidic systems, multiplexed arrays, or IoT-enabled monitoring platforms underscores the field's continued emphasis on materials development at the expense of systems engineering.^294,295^

Addressing these challenges will require a coordinated, multidisciplinary research effort. Mechanistic studies using advanced spectroscopic techniques must be prioritized to establish clear structure–property relationships. Standardized validation protocols incorporating realistic multi-ion challenges, matrix-matched calibration, and stability benchmarks are urgently needed. Parallel development of cadmium-free QD materials, scalable manufacturing strategies, and interlaboratory validation studies will be essential for regulatory acceptance. Ultimately, meaningful translation of QD-based Cd^2+^ sensors will depend on close collaboration among chemists, environmental engineers, toxicologists, regulators, and industry partners, enabling these technologies to evolve from laboratory demonstrations into practical tools for environmental monitoring and public health protection.

### 5.4. Detection of chromium (Cr)

Chromium is a widely distributed transition metal whose environmental and biological impact is strongly dependent on its oxidation state. Among its various valence forms, trivalent chromium (Cr^3+^) is considered an essential trace element involved in glucose and lipid metabolism, whereas hexavalent chromium (Cr^6+^) is highly toxic and poses severe risks to human health and ecosystems. Owing to its strong oxidizing nature, Cr^6+^ exhibits pronounced teratogenic and carcinogenic properties and is classified as a priority pollutant in environmental regulations worldwide. Exposure to Cr^6+^ can occur through dermal contact, inhalation, or ingestion, leading to a wide range of adverse effects, including skin ulceration, allergic reactions, respiratory damage, renal and hepatic dysfunction, and an increased risk of gastrointestinal and pulmonary cancers. In addition to its acute toxicity, Cr^6+^ is environmentally persistent, non-biodegradable, and prone to bioaccumulation, allowing even trace concentrations to pose long-term threats to human health and aquatic and terrestrial organisms. Industrial activities such as electroplating, leather tanning, textile processing, and pigment manufacturing are major sources of Cr^6+^ contamination in water and soil, underscoring the urgent need for sensitive, selective, and field-deployable monitoring technologies. Conventional analytical methods, including AAS and inductively coupled plasma-based techniques, offer high sensitivity but are often limited by high cost, complex sample preparation, and lack of portability. In this context, fluorescence-based sensing platforms, particularly those employing carbon nanomaterials, have emerged as promising alternatives for Cr^6+^ detection due to their high sensitivity, rapid response, operational simplicity, and potential for on-site analysis. Recent advances in CD-based sensors highlight their tunable surface chemistry, low toxicity, and compatibility with smartphone-assisted detection systems, making them attractive candidates for real-world environmental monitoring. The following section discusses recent progress in Cr^6+^ detection using CD-based fluorescent sensors, with particular emphasis on portable and environmentally benign sensing strategies.^296–298^

Feng *et al.* developed iron-doped carbon dots (CS-CDs) *via* one-step hydrothermal synthesis of chitosan and urea, yielding a graphitized core with amino, hydroxyl, and carboxyl surface groups for high water dispersibility, integrated into a PVA/chitosan hydrogel for Cr(vi) detection *via* inner filter effect (IFE) quenching (*λ*_ex_ = 330 nm, *λ*_em_ = 408 nm).^299^ Selectivity for Cr(vi) over Cr^3+^ and other ions was achieved by optimizing pH to 1 to maximize spectral overlap-driven IFE. The liquid-phase sensor achieved a LOD of 0.59 µM across a 1 to 400 µM range, with a robust dual-range linear response (1 to 70 and 70 to 400 µM) (Fig. 23). The hydrogel-integrated format enabled instrument-free, smartphone-based grayscale detection with a LOD of 2.83 µM. Recovery in river and tap water ranged 98.22 to 103.91%. Scalability is constrained by the requirement for highly acidic conditions and a 30-minute hydrogel incubation period.

**Fig. 23:**
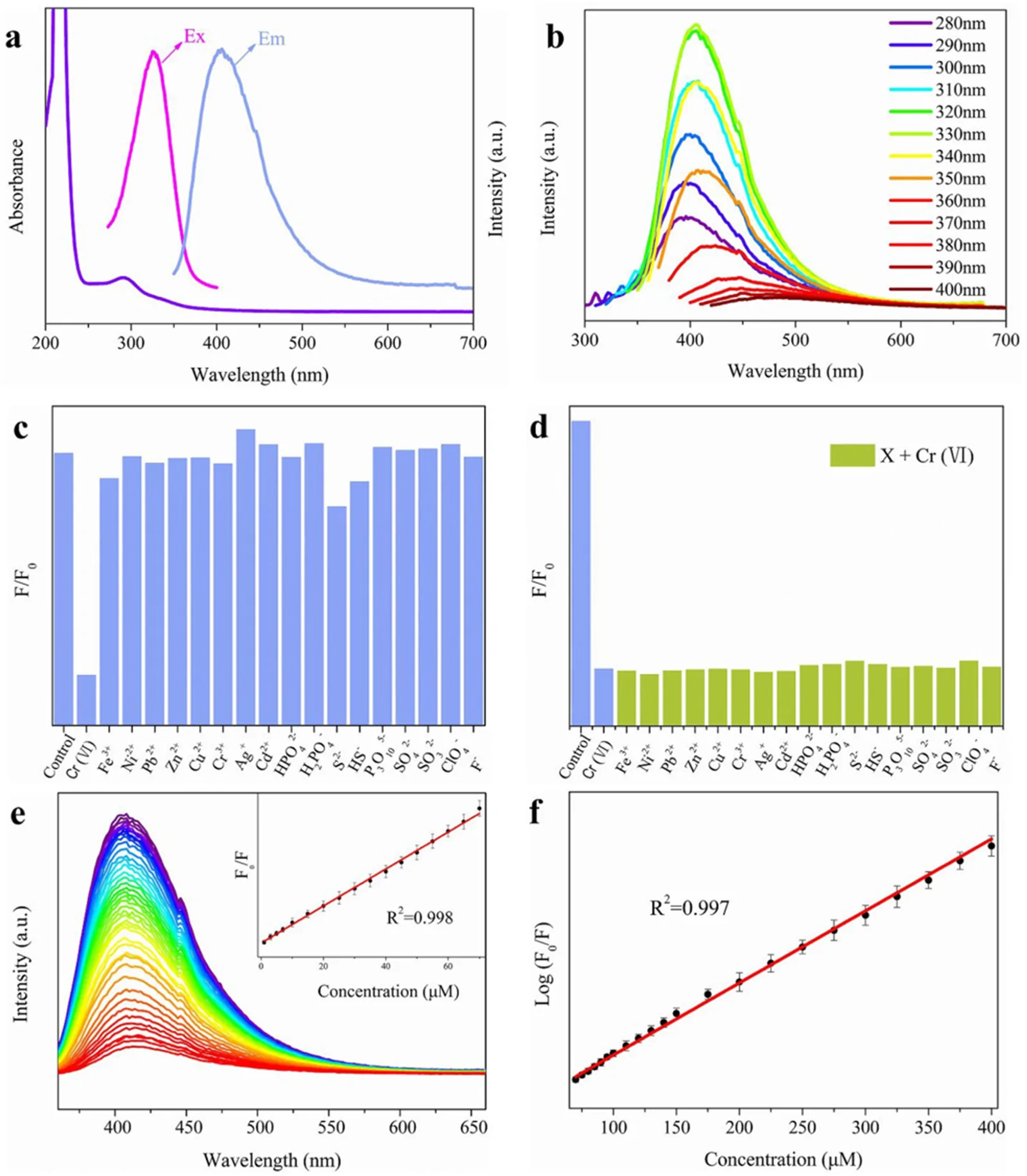
(a) UV-visible absorption profile and corresponding fluorescence emission characteristics of CS-CDs. (b) Emission spectra of CS-CDs recorded under varying excitation wavelengths, demonstrating excitation-dependent fluorescence behaviour. (c and d) Comparative fluorescence responses of CS-CDs toward a range of metal ions in the absence and presence of Cr(vi), highlighting the selective response to chromium. (e) Evolution of the fluorescence emission spectra of CS-CDs with increasing Cr(vi) concentrations. Inset: Calibration plot of *F*_0_/*F* as a function of Cr(vi) concentration over the 1–70 µM range. (f) Logarithmic calibration curve of log(*F*_0_/*F*) *versus* Cr(vi) concentration spanning 70–400 µM.^299^

Zhao *et al.* developed a PEC sensor for Cr(vi) using a layer-by-layer Ag_2_S/MnO_2_/Ag_2_S architecture on an ITO electrode, where the staggered bandgaps of Ag_2_S QDs (1.90 eV) and MnO_2_ nanosheets (2.23 eV) suppress electron–hole recombination and enhance baseline photocurrent.^300^ Sensing relies on a target-induced variation strategy: l-ascorbic acid (AA) normally boosts photocurrent by scavenging photogenerated holes, but Cr(vi) consumes AA *via* a spontaneous redox reaction not shared by Cr^3+^, quenching the photocurrent and providing inherent selectivity for the hexavalent species. The sensor achieved an exceptional LOD of 6.46 pM across a 100 pM to 30 µM linear range under pH 6.5. Interference from strong oxidants Fe^3+^ and MnO_4_^−^ was addressed using EDTA and Fe^2+^ as masking agents, respectively. Recovery in river water, tap water, and industrial wastewater ranged 87.26 to 111.6%. Stability of the MnO_2_ nanosheets and AA electron-donating efficiency require the optimized pH, potentially limiting direct analysis of highly acidic or alkaline samples without buffering.

John *et al.* developed nitrogen-doped carbon quantum dots (N-CQDs) *via* green, one-step hydrothermal synthesis using Ruta graveolens leaf extract, yielding 4.5 nm spherical particles with a graphitic core and amino/hydroxyl surface groups for aqueous dispersibility and metal-ion coordination.^301^ Selective quenching of the N-CQDs (*λ*_ex_ = 380 nm, *λ*_em_ = 458 nm) by Cr(vi) proceeds *via* combined static quenching and inner filter effect (IFE), driven by spectral overlap between Cr(vi) absorption and the N-CQD optical profile, a feature absent for Cr^3+^, enabling discrimination between chromium oxidation states. The sensor achieved a fluorescence LOD of 300 nM across a 0 to 15 µM linear range, with a robust Stern–Volmer relationship confirmed for quantification (Fig. 24). N-CQD-coated paper strips enabled instrument-free, visual detection of Cr(vi) in both solution and solid-state formats, with recovery in river and tap water ranging 98 to 101%. Fluorescence stability requires pH 4 to 8, potentially necessitating buffering for highly acidic or alkaline industrial effluents.

**Fig. 24:**
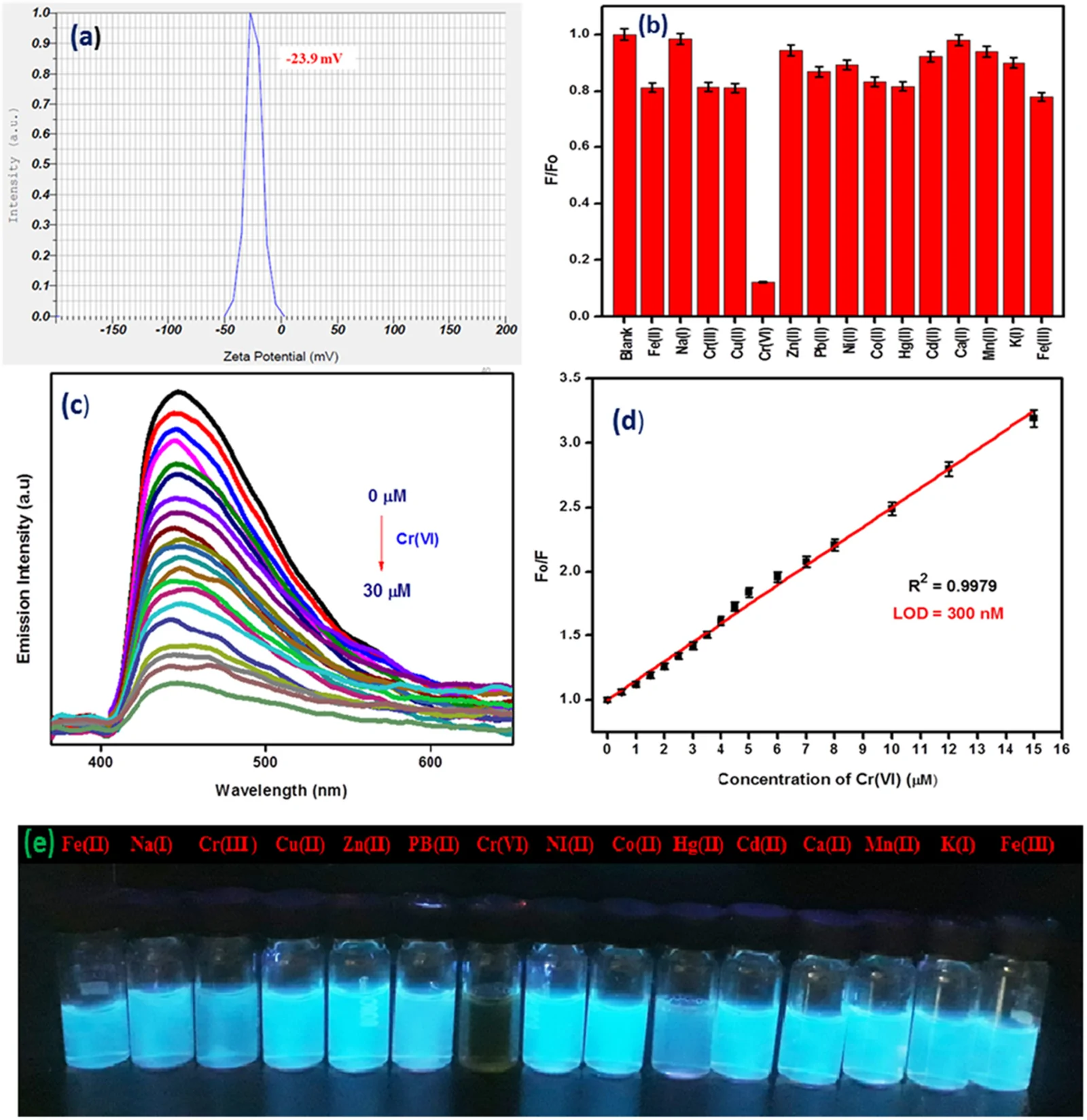
(a) Zeta potential of N-CQDs. (b) Fluorescence response of N-CQDs toward different metal ions. (c) Concentration-dependent fluorescence quenching of N-CQDs upon exposure to Cr(vi). (d) Calibration curve of *F*_0_/*F versus* Cr(vi) concentration in the 0–15 µM range. (e) Visual fluorescence response of N-CQD solutions containing various metal ions (20 µM) under UV illumination.^301^

Tan *et al.* developed heavy-metal-free sulfur quantum dots (SQDs) *via* top-down hydrogen peroxide-assisted etching of sublimed sulfur, PEG-400 passivated, with a polycrystalline sulfur phase and sulfonate/hydroxyl surface groups, for Cr(vi) detection *via* IFE-dominated fluorescence quenching (*λ*_ex_ = 360 nm, *λ*_em_ = 440 nm), confirmed by spectral overlap and negligible fluorescence lifetime change.^302^ The sensor achieved a LOD of 0.36 µM across a 10 to 120 µM linear range under pH 6.6, with an ultrafast response time (<5 minutes). A fluorescence recovery feature allows signal restoration *via* ascorbic acid-mediated reduction of Cr(vi) to Cr(iii). Recovery in lake water ranged 98.3 to 102.8%. The moderate QY (11%) and sensitivity, while robust for routine assays, are surpassed by some nitrogen-doped or hybrid QD systems in recent literature; redox-based recovery stability requires the specified pH, limiting direct use in highly acidic industrial wastewater.

Cilamkoti and Dutta developed a hybrid probe combining SiO_2_ quantum dots (3 to 10 nm) anchored on carbon nanodiscs (SiO_2_-QDs@CND), synthesized hydrothermally with APTES and trisodium citrate, with hydroxyl, carboxyl, and amide surface groups, for Cr(vi) detection *via* ground-state complexation (with an 18% IFE contribution) quenching the blue fluorescence (*λ*_em_ = 440 nm).^303^ The sensor achieved a LOD of 0.78 ± 0.01 µM across a 1 to 75 µM linear range at neutral pH (7.0), with a rapid 30-second response, notable since many biomass-derived sensors require strongly acidic conditions. Selectivity against Cr^3+^ and a range of interfering cations and anions was confirmed *via* stable *I*_0_/*I* quenching ratios (Fig. 25). Recovery in river and tap water ranged 78 to 118%. Sensitivity is markedly reduced at pH < 4, limiting direct application to certain industrial effluents without pH adjustment.

**Fig. 25:**
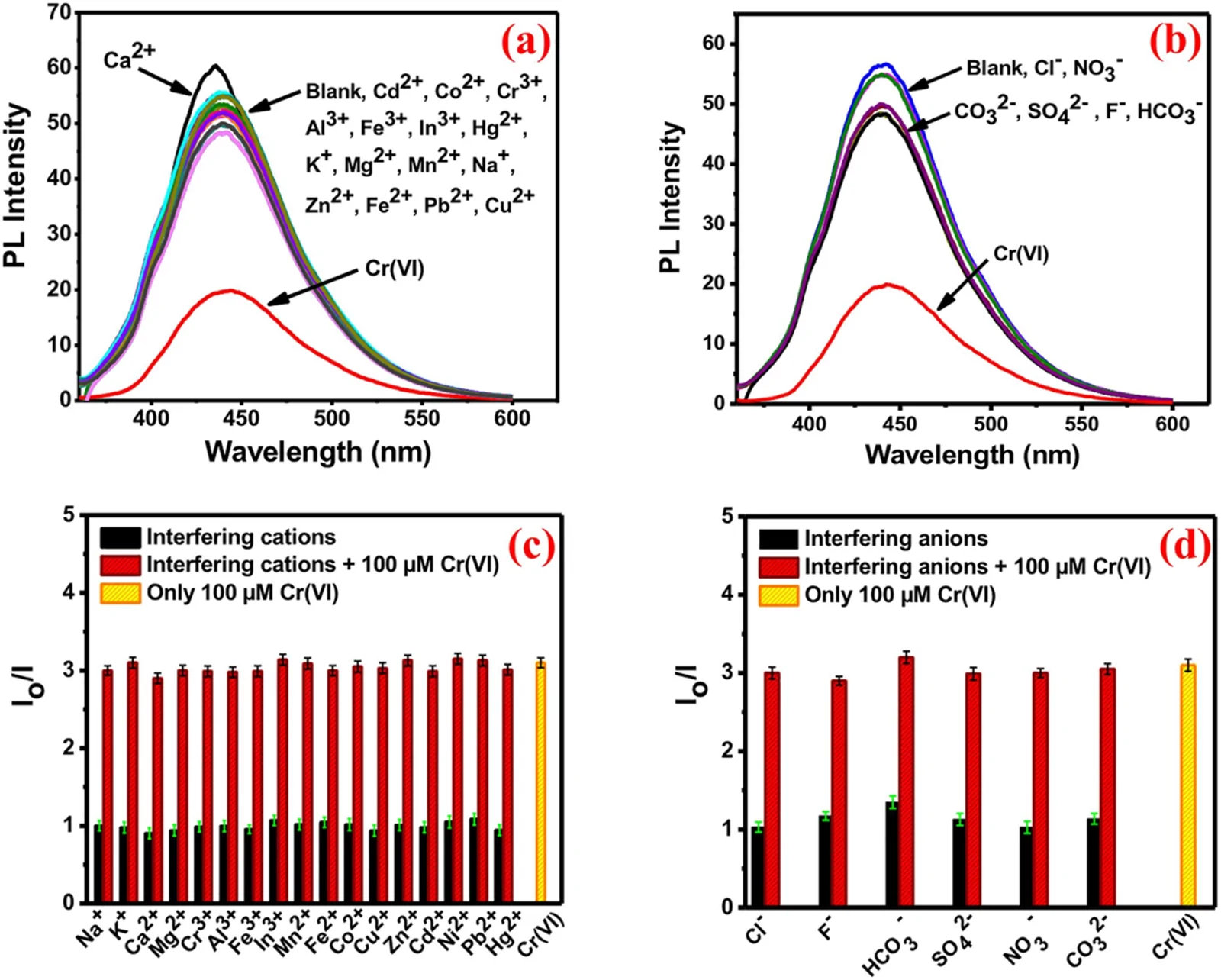
Photoluminescence spectra of SiO_2_-QDs@CND in the presence of interfering (a) cations and (b) anions. (c and d) Bar plots of photoluminescence quenching (*I*_0_/*I*) for SiO_2_-QDs@CND upon treatment with individual interfering (c) cations or (d) anions in comparison with Cr(vi).^303^

Guo *et al.* developed nitrogen and sulfur co-doped carbon dots (NSCDs) *via* one-step hydrothermal treatment of glycine and 4-sulfophthalic acid, yielding ∼3.14 nm spherical particles with amino, hydroxyl, carboxyl, and sulfonate surface groups, for Cr^3+^ detection *via* fluorescence quenching (*λ*_ex_ = 413 nm, *λ*_em_ = 492 nm) driven by inhibition of n to π* transitions and enhanced non-radiative decay.^304^ Specificity for Cr^3+^ was demonstrated against 17 other common ions, with stable optical performance across pH 1 to 14. The sensor achieved a LOD of 7.8 nM across a 0 to 40 µM linear range. Biological validation confirmed high biocompatibility and negligible cytotoxicity, enabling use as an intracellular probe with visible fluorescence attenuation in Kyse-150 cells upon Cr^3+^ exposure (Fig. 26). Recovery in tap water ranged 99 to 106.4%. Steric hindrance and multiple oxygen bonds limit Cr^3+^ access to sulfur binding sites, preventing complete fluorescence quenching and potentially affecting the absolute signal range in highly concentrated samples.

**Fig. 26:**
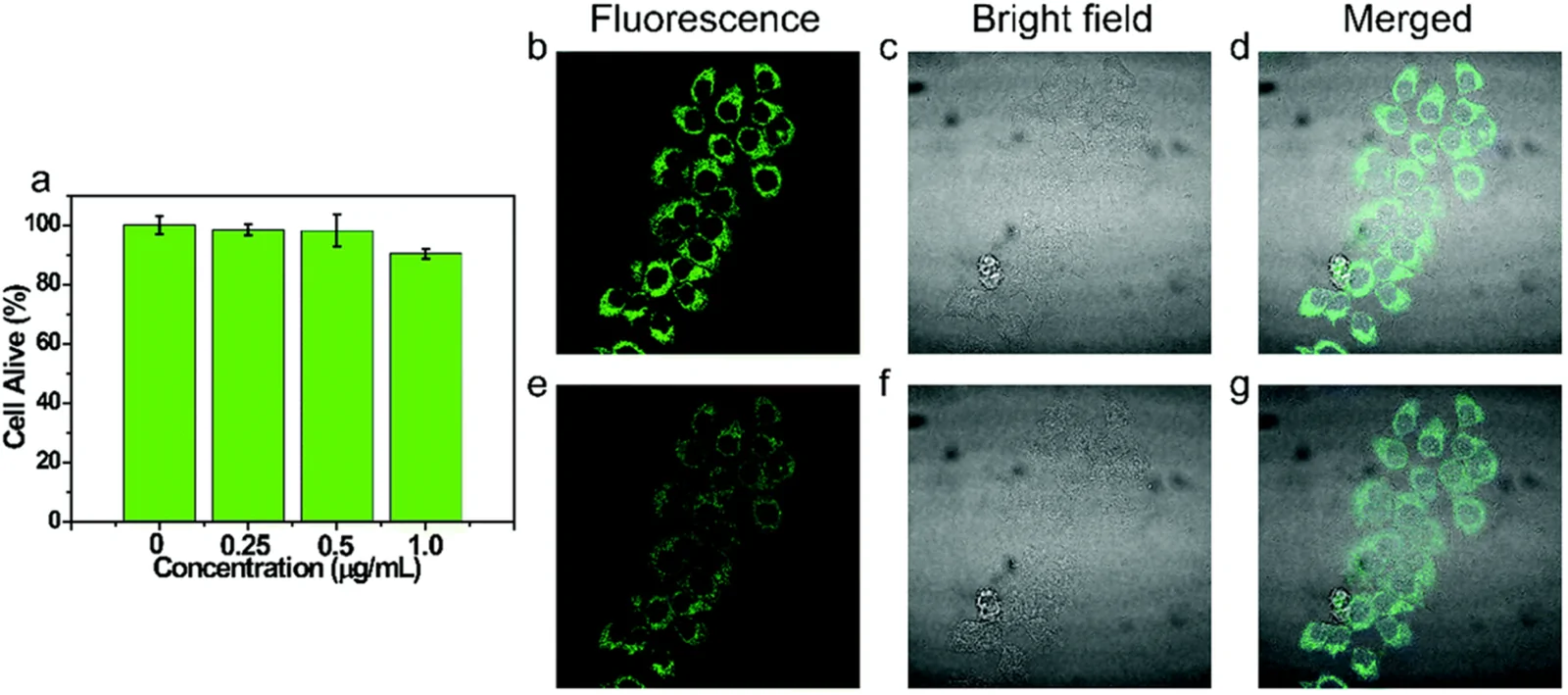
(a) Cell viability of Kyse-150 cells following exposure to varying concentrations of NSCDs. (b–d) Confocal fluorescence images of live Kyse-150 cells incubated with NSCDs (25 µg mL^−1^). (e–g) Confocal fluorescence images of live Kyse-150 cells treated with NSCDs (25 µg mL^−1^) after the introduction of Cr^3+^, illustrating the intracellular fluorescence response to chromium.^304^

Xia *et al.* developed heavy-metal-free sulfur quantum dots (SQDs, 5 to 10 nm) *via* a top-down “assembling-fission” method using sublimed sulfur and PEG-400, with a polycrystalline sulfur core and sulfonyl, carboxyl, and hydroxyl surface groups, for a reversible “ON–OFF–ON” Cr(vi) sensing platform *via* IFE-dominated quenching (*λ*_ex_ = 395 nm, *λ*_em_ = 500 nm) at physiological pH 7.0.^305^ Selectivity for Cr(vi) over Cr^3+^ and common matrix cations was demonstrated. The sensor achieved a LOD of 1.5 µM across a dual-linear range (5 to 100 and 200 to 1500 µM). Bioimaging in zebrafish embryos and larvae visualized Cr(vi) bioaccumulation and its reduction by endogenous ascorbic acid (AA), which restores fluorescence unless inhibited by ascorbic acid oxidase (Fig. 27). Sequential Cr(vi)/AA detection was validated in river water and human serum, with recoveries exceeding 97%. Precise incubation times (1 minute quenching, 15 minutes recovery) and a moderate QY may limit sensitivity in ultra-trace detection scenarios.

**Fig. 27:**
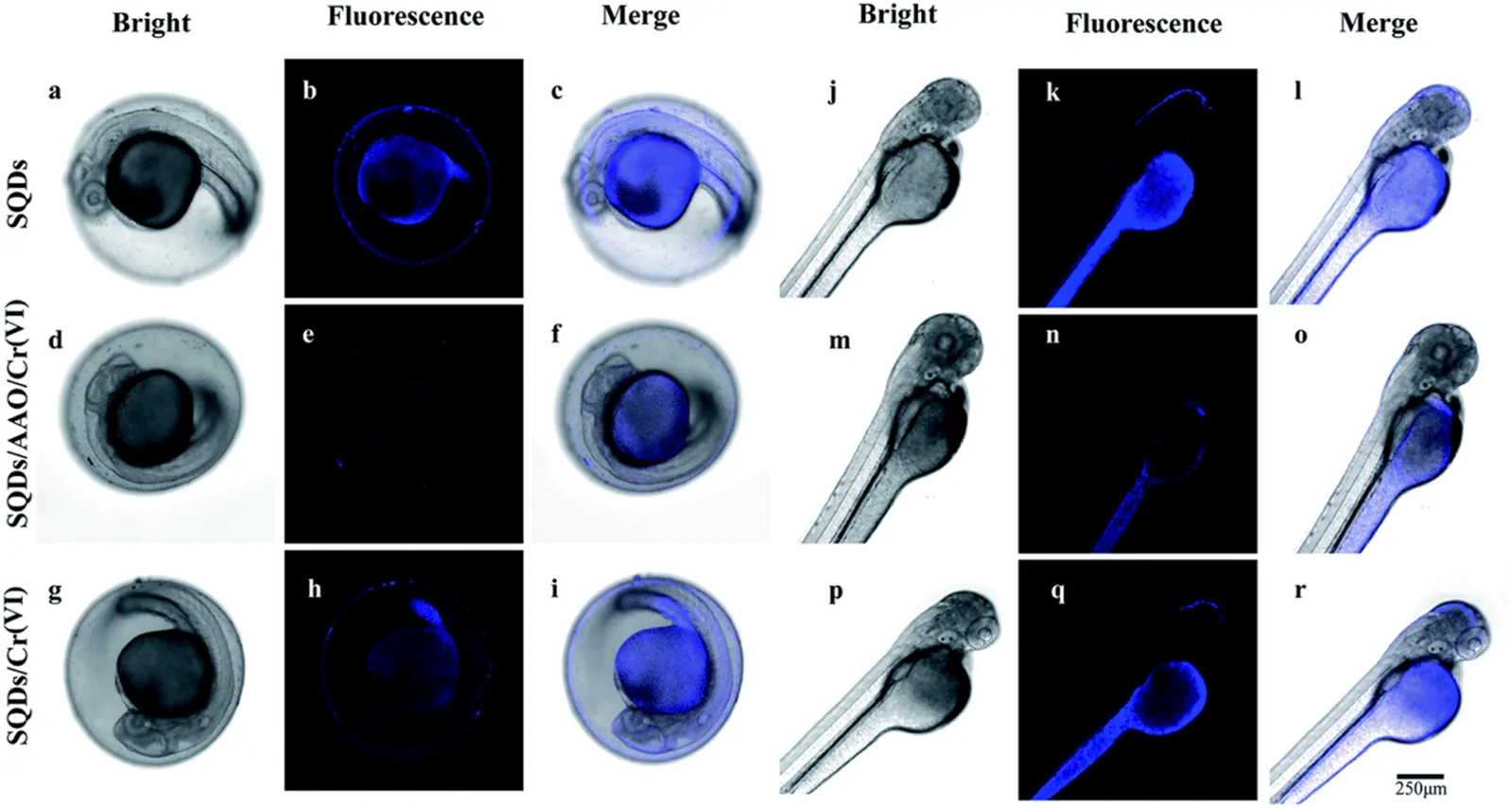
(a–c and j–l) Fluorescence micrographs of zebrafish embryos and larvae following incubation with SQDs. (d–f and m–o) Fluorescence images of zebrafish embryos and larvae treated with ascorbic acid oxidase (AAO, 2 U mL^−1^) in the SQDs/Cr(vi) sensing system. (g–i and p–r) Fluorescence images of zebrafish embryos and larvae exposed to the SQDs/Cr(vi) system. Images are shown across different acquisition modes: bright-field (first column), blue fluorescence channel (second column; excitation at 345 nm), and merged images (third column) combining bright-field and fluorescence signals.^305^

#### 5.4.1. Critical analysis

QD-based sensors for chromium detection have emerged as a rapidly expanding research domain, motivated primarily by the acute toxicity and carcinogenicity of hexavalent chromium [Cr(vi)], which poses severe threats to public health through contaminated groundwater, industrial effluents, and occupational exposures. Unlike many heavy metal contaminants that exist predominantly in a single oxidation state, chromium's environmental chemistry is dominated by the interconversion between Cr(vi) and the far less toxic trivalent form [Cr(iii)], introducing a speciation challenge that most QD-based sensing platforms inadequately address. As detailed in Table 17, recent developments have produced sensors with ostensibly remarkable performance characteristics, LODs spanning from picomolar to low micromolar concentrations, rapid response times as short as 30 seconds, and claims of high selectivity. Yet these advances remain overwhelmingly confined to controlled laboratory environments, with minimal progress toward the speciation-selective, matrix-tolerant, and operationally robust systems required for meaningful environmental and industrial monitoring.^306^

**Table 17:** Comparative analysis of QD-based sensors for chromium detection

Material	Synthesis method	Surface groups/passivation	Signal mechanism	Target analyte	Sensing mechanism	Linear range	LOD	Interferences tested	Conditions	Sample types	Advantages	Limitations
Sulfur QDs (SQDs)^305^	Top-down “assembling-fission”	Sulfonyl, carboxyl, and hydroxyl groups; PEG-400 passivated	Fluorescence (“ON–OFF–ON”)	Cr(vi)	IFE; coordination interaction	5–100 µM and 200–1500 µM	1.5 µM	Ca^2+^, Fe^3+^, Zn^2+^, K^+^, NH_4_^+^, Co^2+^, Mn^2+^, Cu^2+^, Mo^2+^, Al^3+^, Mg^2+^, Ag^+^	pH 7.0; 1 min (quenching); 15 min (recovery)	Tap water, river water, human serum (97.0–105.6%)	Reversible sensing; low toxicity; bioimaging in HeLa cells and zebrafish	Moderate QY; requires precise incubation for recovery
Ag_2_S QDs/MnO_2_ nanosheets^300^	Microwave-assisted (Ag_2_S); BSA exfoliating (MnO_2_)	BSA (stabilizer); d-penicillamine (sulfur source)	PEC	Cr(vi)	Target-induced redox reaction consuming AA (electron donor)	1.0 × 10^−10^ to 3.0 × 10^−5^ M	6.46 × 10^−12^ M	Fe^3+^ and MnO_4_^−^ interfere (require masking with EDTA/Fe^2+^)	pH 6.5; 0.1 M PBS; 0.1 M AA	River, tap water, wastewater (87.26–111.6%)	Exceptional sensitivity (pM level); low background noise	MnO_2_ nanosheets unstable in strong acid; requires electron donor (AA)
Iron-doped CDs (CS-CDs)/PVA-chitosan hydrogel^299^	One-step hydrothermal	Fe-doped; amino, hydroxyl, and carboxyl groups	Fluorescence; smartphone grayscale	Cr(vi)	IFE; auxiliary electron transfer	1–70 µM and 70–400 µM (liquid); 10–300 µM (gel)	0.59 µM (liquid); 2.83 µM (gel)	Fe^3+^, Ni^2+^, Pb^2+^, Zn^2+^, Cu^2+^, Cr^3+^, Ag^+^, Cd^2+^, *etc.*	pH 1.0 (for quenching); pH 2–11 stability	River water and tap water (98.22–103.91%)	Portable, instrument-free quantification; sustainable biomass source	Optimal sensitivity requires highly acidic conditions (pH 1.0)
N-doped CDs (N-CQDs)^301^	One-step hydrothermal (*R. graveolens* extract)	N-doped; hydroxyl and amine groups	Fluorescence; electrochemical (DPV)	Cr(vi)	Static quenching and IFE	0–15 µM (FL); 20–70 nM (DPV)	300 nM (FL); 5 nM (DPV)	Na^+^, K^+^, Mn^2+^, Zn^2+^, Fe^2+^, Ca^2+^, Hg^2+^, Ni^2+^, Cd^2+^, Cr^3+^, Pb^2+^, Cu^2+^, Co^2+^, Fe^3+^	pH 7.0 (FL); pH 4.0 (DPV); 4 min interaction	Tap water and river water (98–101%)	Green synthesis; dual-mode detection; solid-state paper strips	Fluorescence stability restricted to pH 4–8 window
SiO_2_-QDs@CND (silicon dots on carbon nanodiscs)^303^	Hydrothermal	Hydroxyl, carboxyl, and amide groups	Fluorescence	Cr(vi)	Ground-state complexation (static quenching); minor IFE (18%)	1–75 µM	0.78 µM	Na^+^, K^+^, Ca^2+^, Mg^2+^, Cr^3+^, Al^3+^, Fe^3+^, Mn^2+^, Pb^2+^, Hg^2+^, various anions	pH 7.0; 30 s response time	River water and municipality tap water (78–118%)	Rapid response (30 s); operates at neutral pH; heavy-metal-free	Quenching efficiency significantly decreases at pH < 4
Sulfur QDs (SQDs)^302^	H_2_O_2_-assisted top-down etching	Sulfonate and hydroxyl groups; PEG-400 passivated	Fluorescence	Cr(vi)	IFE	10–120 µM	0.36 µM	Fe^3+^, Mn^2+^, Ni^2+^, Zn^2+^, Cd^2+^, Pb^2+^, Ag^+^, Cr^3+^, Al^3+^, *etc.*	pH 6.6; 5 min response time	Lake water (98.3–102.8%)	Eco-friendly; fast response; reversible signal recovery with AA	Moderate QY (11%); sensitivity lower than some doped CDs
N,S co-doped CDs (NSCDs)^304^	One-step hydrothermal	N,S co-doped; amino, hydroxyl, carboxyl, sulfonate groups	Fluorescence	Cr^3+^	Inhibition of n–π* transition; non-radiative carrier depopulation	0–40 µM	7.8 nM	F^−^, Cl^−^, Br^−^, SO_4_^2−^, CO_3_^2−^, NO_3_^−^, K^+^, Na^+^, Mg^2+^, Ba^2+^, Ca^2+^, Cu^2+^, Zn^2+^, Ag^+^, Ni^2+^, Pb^2+^, Mn^2+^	pH 1.0–14.0 stability	Tap water (99.0–106.4%)	Highly selective for Cr^3+^; intracellular bioimaging; broad pH stability	Surface steric hindrance prevents 100% signal quenching

The chromium speciation problem represents the most critical yet consistently underaddressed challenge in the field. Cr(vi) and Cr(iii) exhibit drastically different toxicological profiles: Cr(vi) is a well-established human carcinogen that readily penetrates biological membranes, whereas Cr(iii) is an essential micronutrient at trace levels. Regulatory frameworks, including the U.S. EPA's Maximum Contaminant Level of 100 µg L^−1^ for total chromium and California's far stricter 10 µg L^−1^ limit specifically for Cr(vi), explicitly recognize this distinction. However, the majority of QD sensors listed in Table 17 report detection of “Cr(vi)” based solely on differential reactivity rather than true speciation capability. For instance, while Feng *et al.*,^299^ John *et al.*,^301^ and Xia *et al.*^305^ all claim Cr(vi) selectivity, their discrimination relies on the stronger oxidizing power of Cr(vi) relative to Cr(iii) under specific pH conditions, not on genuine chemical speciation. This approach fails catastrophically in real environmental samples where pH fluctuations, redox-active organic matter, and microbial activity drive continuous Cr(vi) ↔ Cr(iii) interconversion. Critically, only Guo *et al.* explicitly designed their sensor for Cr(iii) detection, underscoring the field's overwhelming bias toward Cr(vi) despite the analytical necessity of measuring both species independently. None of the reviewed studies incorporate sequential extraction protocols, online speciation preservation, or coupling with separation techniques such as ion chromatography, leaving the fundamental question of whether reported signals truly reflect Cr(vi) concentrations largely unanswered.^301,307^

pH-dependent performance introduces a second layer of complexity that severely restricts practical applicability. The data in Table 17 reveal a troubling dichotomy: sensors optimized for maximum sensitivity often operate under extreme pH conditions incompatible with environmental monitoring. Feng *et al.* achieved their lowest LOD (0.59 µM) only at pH 1.0, a condition that not only perturbs chromium speciation but also accelerates fluorophore degradation and corrodes sensor materials.^299^ While the same system exhibited pH 2–11 stability, optimal quenching efficiency was confined to highly acidic regimes, raising questions about whether the sensor detects Cr(vi) or merely responds to pH-induced changes in chromium chemistry. Conversely, Cilamkoti & Dutta reported severe quenching efficiency loss below pH 4, effectively limiting their SiO_2_-QDs@CND platform to near-neutral conditions. This restriction is particularly problematic for industrial applications, where chromium contamination frequently occurs in acidic mine drainage (pH 2–4), electroplating wastewaters (pH 1–3), and tannery effluents with highly variable pH.^303^ Guo *et al.*'s claim of pH 1.0–14.0 stability for their N,S co-doped CDs represents an outlier, yet even this system provides no data on whether sensing performance remains constant across this pH range or merely whether the fluorophore survives.^304^ The absence of systematic pH-titration studies correlating speciation, sensing mechanism, and analytical performance reflects a critical gap that prevents rational sensor design for specific application contexts.

Mechanistic ambiguity further undermines the credibility of performance claims. As shown in Table 17, proposed mechanisms span a broad spectrum: IFE, static quenching, ground-state complexation, electron transfer, PEC redox reactions, and coordination interactions. However, the experimental rigor supporting these assignments varies dramatically. The widespread invocation of IFE is often based solely on spectral overlap between QD emission and Cr(vi) absorption, without time-resolved fluorescence measurements to exclude dynamic quenching contributions. Zhao *et al.*'s PEC system introduces additional mechanistic complexity, attributing response to target-induced consumption of ascorbic acid (the electron donor) rather than direct Cr(vi)–QD interaction.^300^ While this indirect sensing strategy achieved exceptional sensitivity (6.46 pM LOD), it raises fundamental questions about specificity: any oxidizing agent capable of consuming ascorbic acid would produce an analogous signal. The dependence on external redox mediators also introduces calibration drift, as ascorbic acid undergoes spontaneous oxidation even in the absence of analyte. More troubling is the lack of direct spectroscopic evidence, XPS, EXAFS, or Raman spectroscopy, confirming proposed chromium–QD binding modes or oxidation state changes. Feng *et al.* attribute sensing to “auxiliary electron transfer” without providing electron paramagnetic resonance (EPR) data or cyclic voltammetry evidence for electron transfer events. This mechanistic vagueness is not merely an academic concern; it prevents systematic optimization, limits predictive modeling, and obscures whether sensors respond to total chromium, Cr(vi) specifically, or redox potential more generally.^299^

Interference management strategies reveal a critical disconnect between claimed selectivity and practical matrix complexity. Table 17 uniformly reports tolerance toward 8–15 common cations and anions, yet interference testing protocols remain superficial. Zhao *et al.* explicitly acknowledge interference from Fe^3+^ and MnO_4_^−^, requiring pretreatment with EDTA and Fe^2+^ as masking agents, a two-step procedure that negates the simplicity advantage of optical sensing and introduces additional sources of error.^300^ The recurrence of Fe^3+^ interference across multiple studies is particularly concerning given that iron concentrations in groundwater typically exceed chromium by 100–1000-fold, especially in reducing environments where Cr(iii) predominates. While HSAB principles predict moderate affinity between Cr(iii) and nitrogen/sulfur donor groups on QD surfaces, they fail to explain the reported selectivity of Cr(vi) sensors, as hexavalent chromium exists predominantly as oxyanions (CrO_4_^2−^, Cr_2_O_7_^2−^, HCrO_4_^−^) that interact through electrostatic or hydrogen-bonding mechanisms rather than coordination. More fundamentally, selectivity is almost universally assessed through sequential single-analyte addition rather than simultaneous multi-ion competition under environmentally realistic concentration ratios. Real industrial effluents contain complex mixtures where Ni^2+^, Cu^2+^, Zn^2+^, and Fe^3+^ coexist at concentrations orders of magnitude higher than chromium, creating competitive binding scenarios never replicated in published studies. The absence of principal component analysis (PCA), partial least squares (PLS) regression, or other chemometric approaches capable of deconvoluting multi-analyte responses represents a missed opportunity to transform a limitation into a multiplexed sensing capability.

Response kinetics and reversibility introduce operational constraints often glossed over in performance summaries. Cilamkoti & Dutta's 30-second response time appears exceptional, yet the study provides no data on whether this reflects true equilibration or merely an arbitrary measurement endpoint.^303^ Feng *et al.*^299^ and John *et al.* report 4-minute interaction times without kinetic modeling to establish rate constants or reaction orders.^301^ Xia *et al.*'s “ON–OFF–ON” reversible sensing system, while conceptually attractive for reusable sensors, requires 15 minutes for signal recovery following addition of GSH, a timescale incompatible with continuous monitoring or high-throughput screening.^305^ The chemical basis for reversibility, attributed to competitive coordination, remains unvalidated through binding affinity measurements or equilibrium constant determinations. More critically, repeated sensing cycles invariably induce photodegradation, surface oxidation, and ligand loss, yet no study quantifies signal drift over multiple use cycles or provides operational lifetime projections. The lack of accelerated aging studies, continuous-operation stress tests, or quantitative photobleaching assessments leaves the practical durability of these sensors entirely speculative.

Real-sample validation, while universally conducted, reveals systematic weaknesses that question translational readiness. Recovery percentages ranging from 78–118% (ref. 303) and 87.26–111.6% (ref. 300) fall well outside the 95–105% window considered acceptable for regulatory methods. These deviations indicate significant matrix effects, interference from dissolved organic carbon, colloid formation, or competitive binding, that are not adequately modeled or corrected. Critically, all validation studies employ the standard addition method, spiking known chromium concentrations into real samples, rather than analyzing certified reference materials with established chromium content. This approach cannot distinguish between matrix-induced signal suppression and genuine analytical bias. Furthermore, sample pretreatment protocols, filtration, pH adjustment, dilution, are inconsistently reported and vary dramatically between studies, preventing meaningful inter-study comparison. Feng *et al.*'s application to river and tap water,^299^ John *et al.*'s validation in tap and river samples,^301^ and Xia *et al.*'s testing in human serum all lack critical details, chromium speciation in the original samples,^305^ background chromium levels prior to spiking, interfering ion concentrations, and dissolved organic carbon content. The absence of head-to-head comparisons with ICP-MS, graphite furnace AAS (GFAAS), or validated colorimetric methods means that reported recoveries cannot be independently verified. Moreover, no study addresses the temporal stability of spiked samples or the potential for Cr(vi) reduction during storage and analysis, a well-documented artifact in chromium speciation analysis.

The QY and fluorescence stability constraints highlighted in Table 17 expose fundamental photophysical limitations. Tan *et al.*^302^ and Xia *et al.*^305^ both report “moderate QY” (∼11%), a value that severely limits signal-to-noise ratios and necessitates high excitation intensities that accelerate photobleaching. Carbon-based QDs, while advantageous for their heavy-metal-free composition, inherently suffer from lower QY compared to traditional semiconductor QDs due to defect-mediated non-radiative recombination. Doping strategies, Fe in Feng *et al.*,^299^ N in John *et al.*,^301^ N/S co-doping in Guo *et al.*,^304^ are intended to enhance emission through defect passivation and heteroatom-induced electronic state modification. However, none of these studies provide TCSPC measurements to quantify radiative *versus* non-radiative decay pathways or assess how chromium binding alters excited-state dynamics. The consequence is that “quenching efficiency” remains an empirical parameter rather than a mechanistically understood property, preventing rational design to maximize signal change per unit analyte concentration. Photostability data are conspicuously absent across all studies despite the inherent vulnerability of carbon-based fluorophores to singlet oxygen-mediated oxidation under continuous illumination. For sensors intended for field deployment or smartphone-based readout platforms, applications requiring extended light exposure, this omission is particularly glaring.

Device integration and user-interface development, while receiving increasing attention, remain at rudimentary stages. Feng *et al.*'s incorporation into a PVA-chitosan hydrogel for smartphone-based grayscale analysis^299^ and John *et al.*'s solid-state paper strips represent important steps toward point-of-use testing.^301^ However, these platforms suffer from critical limitations: the hydrogel system exhibited elevated LOD (2.83 µM) compared to solution-phase measurements, while the paper strip format provides no quantitative data on storage stability, humidity tolerance, or batch-to-batch reproducibility. Smartphone-based quantification, though conceptually appealing, introduces additional variables, ambient lighting conditions, camera calibration drift, color balance variations, that are not systematically controlled or validated. None of the studies provide user-testing data, false-positive/false-negative rates under field conditions, or comparisons with handheld spectrophotometers or portable XRF analyzers already deployed for chromium monitoring. The absence of microfluidic integration, automated sample handling, or multiplexed sensor arrays capable of simultaneous speciation and total chromium determination reflects the field's continued focus on materials synthesis over systems engineering.

Synthesis reproducibility and scalability constraints pose underappreciated barriers to commercialization. Hydrothermal methods dominate Table 17, yet these batch processes are notoriously sensitive to heating rate, maximum temperature, dwell time, precursor purity, and autoclave geometry. Feng *et al.*'s one-step hydrothermal synthesis^299^ and John *et al.*'s green synthesis using *Ruta graveolens* extract introduce biological variability that is difficult to control across production batches.^301^ None of the studies report statistical process control data, control charts for QY, emission wavelength, or particle size across multiple synthesis runs, nor do they provide coefficients of variation for key performance metrics. Post-synthetic functionalization steps, such as surface passivation with PEG-400 (ref. 302) or BSA,^300^ introduce additional timing dependencies and reagent concentration sensitivities. The lack of continuous-flow synthesis platforms, which could enable real-time monitoring and feedback control, leaves the field reliant on methods fundamentally unsuited for the reproducibility and throughput required for commercial sensor production. Economic analysis is similarly absent: no study estimates cost per test, compares sensor material costs with consumables for established methods, or projects manufacturing costs at production scales of 10^4^–10^6^ units.

Regulatory and environmental fate considerations introduce a final layer of translational barriers. Unlike cadmium sensors, which face direct regulatory conflicts when employing cadmium-based QDs, chromium sensors utilizing carbon, silicon, or sulfur-based QDs avoid heavy metal toxicity concerns. However, this advantage has led to complacency regarding environmental impact assessment. None of the studies conduct ecotoxicity testing of sensor materials, despite the likelihood of sensor disposal into the same aquatic environments they are designed to monitor. The environmental persistence of carbon nanomaterials, potential bioaccumulation in aquatic food webs, and interactions with microbial communities remain entirely uncharacterized. Furthermore, the use of Ag_2_S QDs by Zhao *et al.*, while achieving exceptional sensitivity, reintroduces heavy metal concerns (silver) without corresponding life-cycle assessment or leaching studies.^300^ Regulatory approval pathways for point-of-use diagnostic devices typically require ISO 13485 quality management, extensive stability databases, and clinical/environmental validation trials, requirements that no current QD-based chromium sensor approaches meeting. The absence of standardized performance metrics complicates this landscape further: without consensus on reporting units (molar *vs.* mass concentration), matrix specifications (synthetic *vs.* real samples), and acceptance criteria (recovery ranges, precision limits), comparative evaluation across studies remains impossible.

Advancing QD-based chromium sensing from laboratory demonstrations to deployable technologies demands a paradigm shift in research priorities. First, speciation-selective platforms capable of independently quantifying Cr(vi) and Cr(iii) must be developed, potentially through dual-mechanism sensors or integration with solid-phase extraction cartridges. Second, mechanistic validation using *operando* spectroscopy, *in situ* XPS during chromium binding, time-resolved fluorescence under analytical conditions, electrochemical impedance spectroscopy for PEC sensors, is essential to move beyond phenomenological descriptions. Third, standardized validation protocols incorporating certified reference materials, matrix-matched calibration across diverse water types (surface, ground, industrial), and interlaboratory reproducibility studies must become mandatory. Fourth, systems engineering efforts, microfluidic integration, multiplexed sensor arrays for simultaneous multi-metal detection, wireless data transmission for distributed monitoring networks, should receive equal emphasis to materials development. Fifth, manufacturing partnerships to develop scalable synthesis platforms with statistical process control and to conduct techno-economic analyses comparing QD sensors with established methods (ICP-MS, ion chromatography, colorimetric kits) are urgently needed. Finally, proactive engagement with regulatory agencies, environmental consultants, and industrial end-users during the development phase, rather than after publication, will ensure that sensor performance targets align with actual monitoring requirements. Only through such coordinated, translational research can QD-based chromium sensors transcend their status as laboratory curiosities to become practical tools addressing a critical global environmental health challenge.

### 5.5. Detection of arsenic (As)

Arsenic contamination of groundwater represents one of the most severe and geographically widespread public health crises, affecting an estimated 140 million people across more than 70 countries, with particularly acute exposure documented in South and Southeast Asia, Latin America, and parts of the United States.^308,309^ The element's intrinsic toxicity manifests through diverse and often irreversible health consequences, including chronic respiratory dysfunction, peripheral vascular disease, hypertension and cardiovascular complications, neurological impairment, developmental defects, and malignancies of the skin, bladder, lung, and liver. Unlike many environmental contaminants that pose risks primarily through acute exposure, arsenic exerts its most devastating effects through chronic, low-level consumption over years to decades, making sensitive, field-deployable detection technologies critical for prevention rather than remediation. The environmental chemistry of arsenic is dominated by complex speciation that varies dramatically with redox potential, pH, microbial activity, and the presence of complexing ligands, creating substantial analytical challenges for selective detection. Under oxidizing conditions typical of surface waters and well-aerated aquifers, arsenate [As(v)] predominates, existing primarily as oxyanions H_2_AsO_4_^−^ and HAsO_4_^2−^ at environmentally relevant pH values (6–8). Conversely, in reducing environments characteristic of anaerobic groundwater, waterlogged soils, and sediment pore waters, arsenite [As(iii)] becomes the dominant species, present predominantly as the uncharged H_3_AsO_3_ molecule below pH 9.2. This speciation distinction carries profound toxicological and analytical implications: As(iii) exhibits 25–60 times greater acute toxicity than As(v), penetrates biological membranes more readily due to its neutral charge and structural mimicry of glycerol, and resists conventional treatment processes such as coagulation and adsorption that effectively remove As(v). Complicating this binary classification, methylated arsenic species, monomethylarsonic acid (MMA) and dimethylarsinic acid (DMA), arise through microbial biomethylation and metabolic detoxification pathways in both environmental and biological systems. While historically considered less toxic than inorganic forms, recent evidence demonstrates that MMA(iii) and DMA(iii), the trivalent intermediates in arsenic methylation, exhibit genotoxicity and cytotoxicity comparable to or exceeding that of inorganic As(iii). Additionally, thioarsenates, generated through sulfide-mediated ligand exchange reactions in sulfate-reducing environments, introduce further complexity: monothioarsenate (H_3_AsO_3_S), dithioarsenate (H_3_AsO_2_S_2_), trithioarsenate (H_3_AsOS_3_), and tetrathioarsenate (H_3_AsS_4_) exhibit distinct stability constants, mobility patterns, and bioavailability compared to their oxygen analogs. The detection of dimethyldithioarsenate (DMDTA) and dimethylmonothioarsenate (DMMTA) in rice paddy pore waters at concentrations sufficient to impact crop uptake underscores that arsenic speciation extends far beyond the conventional As(iii)/As(v) dichotomy.^310–314^

Regulatory frameworks worldwide recognize arsenic's severe health threat through stringent exposure limits, yet analytical enforcement remains inconsistent. The WHO guideline value of 10 µg L^−1^ (approximately 133 nM) for total arsenic in drinking water, adopted by the European Union, Japan, and the United States, represents a pragmatic compromise between health protection and analytical/treatment feasibility rather than a truly safe threshold.^315^ Epidemiological evidence suggests that even concentrations below 10 µg L^−1^ contribute to increased cancer incidence, cardiovascular mortality, and neurodevelopmental deficits, with some jurisdictions considering lower limits (5 µg L^−1^ in New Jersey, USA) or differentiated standards for As(iii) *versus* total arsenic. However, current regulatory monitoring relies almost exclusively on laboratory-based techniques, ICP-MS, hydride generation AAS (HGAAS), and atomic fluorescence spectroscopy (AFS), that require centralized facilities, skilled operators, expensive instrumentation, and sample preservation protocols that often fail to maintain arsenic speciation during transport and storage. This infrastructure-intensive approach creates critical monitoring gaps in resource-limited settings, where arsenic exposure is often most severe, and prevents real-time risk assessment necessary for dynamic aquifer management or treatment system optimization. The confluence of severe health impacts, complex environmental speciation, stringent regulatory requirements, and inadequate field-monitoring capabilities establishes a compelling need for sensitive, selective, speciation-capable, and operationally simple arsenic detection technologies, a niche that QD-based sensors ostensibly aim to fill.^316^

Sadjadi *et al.* developed bisphosphonate-functionalized CDs for turn-on fluorescence detection of As(iii), with bisphosphonate moieties providing chelating sites that leverage phosphonate's established affinity for arsenite.^316^ Unlike conventional quenching-based arsenite sensors, this platform enhances fluorescence upon binding, offering advantages for visual detection. Performance was validated in rice grain and seawater samples, relevant given arsenic bioaccumulation in rice crops and coastal water contamination.

Behera *et al.* developed a manganese dioxide-CD (MnO_2_CD) nanobionic sensor *via* one-step hydrothermal synthesis from polydopamine and citric acid, comprising a nitrogen-doped CD core (425 nm fluorescence) FRET-quenched by a monoclinic k-birnessite MnO_2_ shell.^317^ As(iii) reduces the MnO_2_ shell to soluble Mn, halting FRET and restoring “turn-on” fluorescence; detection extends to As(v)-containing organoarsenic species (*e.g.*, DMA, DMDTA), where the CDs first reduce As(v) to As(iii) *in situ* before triggering MnO_2_ degradation. The sensor achieved a LOD of 5.75 nM (0.76 ppb, below the WHO 10 ppb threshold) across a 0 to 100 nM linear range, with strong selectivity against common interfering ions. *In vivo* imaging (Fig. 28) visualized arsenic uptake and translocation in rice and mung plant roots, extending beyond water analysis to agricultural monitoring. The sensor was robust in saline matrices and physiological pH (5.0 to 9.0), though sensitivity may be affected by extreme pH, limiting use in highly acidic or alkaline industrial effluents. Recovery in tap, river, and seawater ranged 99 to 103%.

**Fig. 28:**
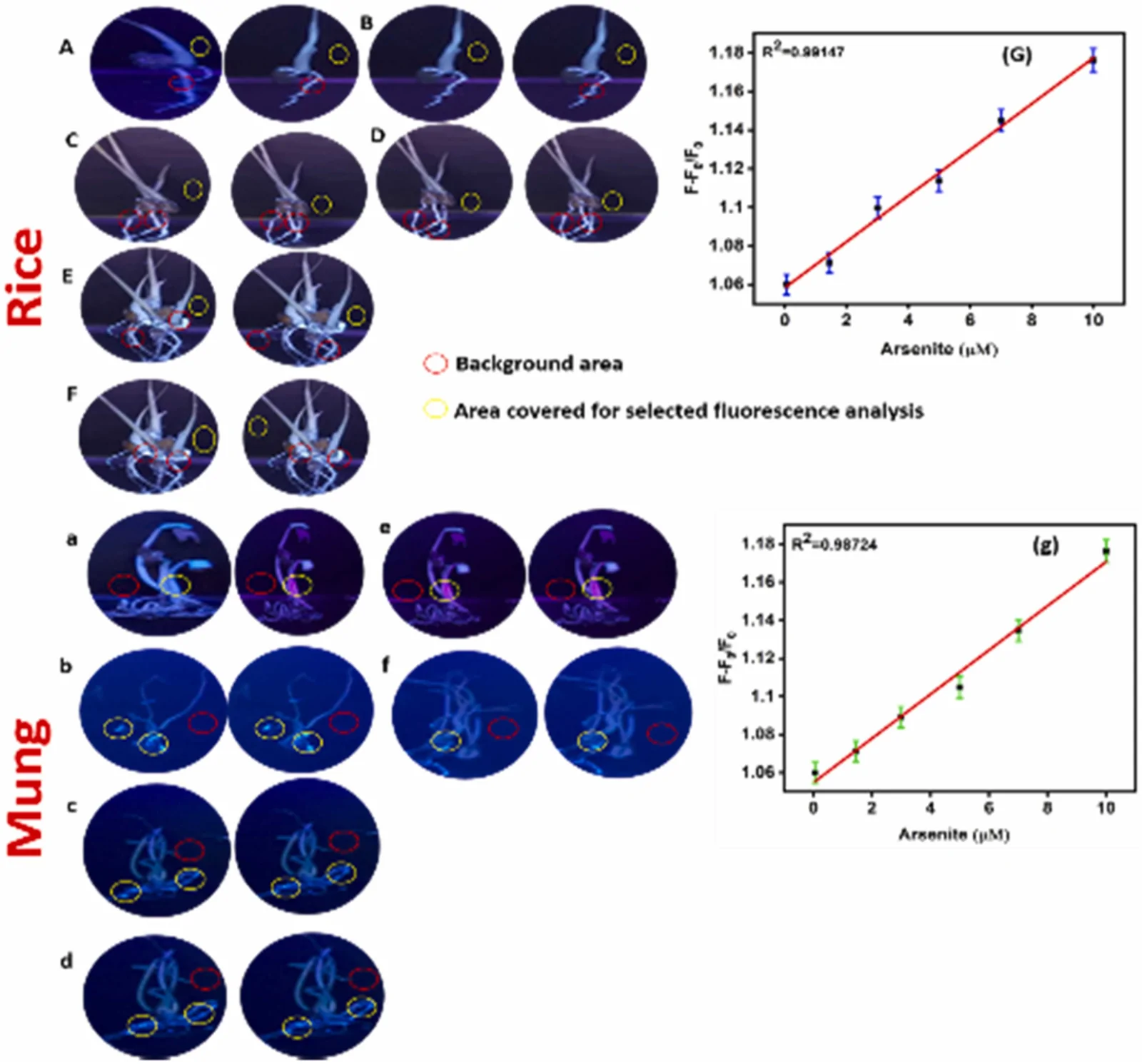
(A–F) Digital fluorescence images of rice roots recorded under UV irradiation following exposure to varying arsenic concentrations. (G) Linear correlation between fluorescence intensity and arsenic concentration in rice root samples. (a–f) Fluorescence images of mung bean sprouts acquired under UV light at different arsenic levels. (g) Corresponding linear calibration plot of fluorescence intensity as a function of arsenic concentration in mung bean sprouts. In all experiments, the MnO_2_CD probe was used at a fixed concentration of 0.4 mg mL^−1^.^317^

Rahimi *et al.* developed a sustainable, one-pot aqueous synthesis for l-methionine (Meth)-capped PbS QDs for turn-off fluorescence detection of As(iii), where Meth's sulfur and nitrogen donor atoms are preferentially displaced by As(iii) to form an *S*,*N*-bidentate chelate, triggering aggregation-induced quenching confirmed by TEM (monodisperse particles to clusters upon 30 ppb As(iii) addition).^318^ The sensor achieved a LOD of 3.7 ppb, below the WHO's 10 ppb threshold, across a 5 to 150 ppb linear range, with negligible interference from common metal ions and, notably, from As(v), which lacks comparable affinity for the sulfur-based capping agent. Synthesis avoided organic solvents and complex surface modification, with a rapid 3-minute response time and robust storage stability. Recovery in tap and river water ranged 96.6 to 100.8%. Optimal sensing occurs at physiological pH 7.4; extreme acidity or alkalinity hinders ligand coordination or induces As(iii) hydroxide formation.

Soni *et al.* developed a label-free aptamer-based sensor combining mercaptopropionic acid-capped CdTe@CdS core–shell QDs with a guanylated copolymer, poly(HPMA-*s*-GPMA), as a programmable quencher, for As(iii) detection.^319^ In the competitive “off–on” mechanism, the copolymer is normally sequestered by the As(iii)-selective aptamer, preserving QD emission (626 nm); As(iii) binding displaces the copolymer, which then aggregates with the negatively charged QDs to quench fluorescence. The sensor achieved a LOD of 249.77 pM (0.018 ppb) across a 0.01 to 100 nM linear range, surpassing previously reported colorimetric and AuNP-based arsenic sensors, with approximately threefold selectivity for As(iii) over competing metal ions *via* aptamer binding affinity. Recovery in mineral water, soil water, and apple juice ranged 91 to 108%, with consistent linear quenching response across all matrices (Fig. 29), including UV-illuminated visual detection. Deployment may be limited by relatively long incubation times and the requirement for controlled pH to preserve guanidinium protonation.

**Fig. 29:**
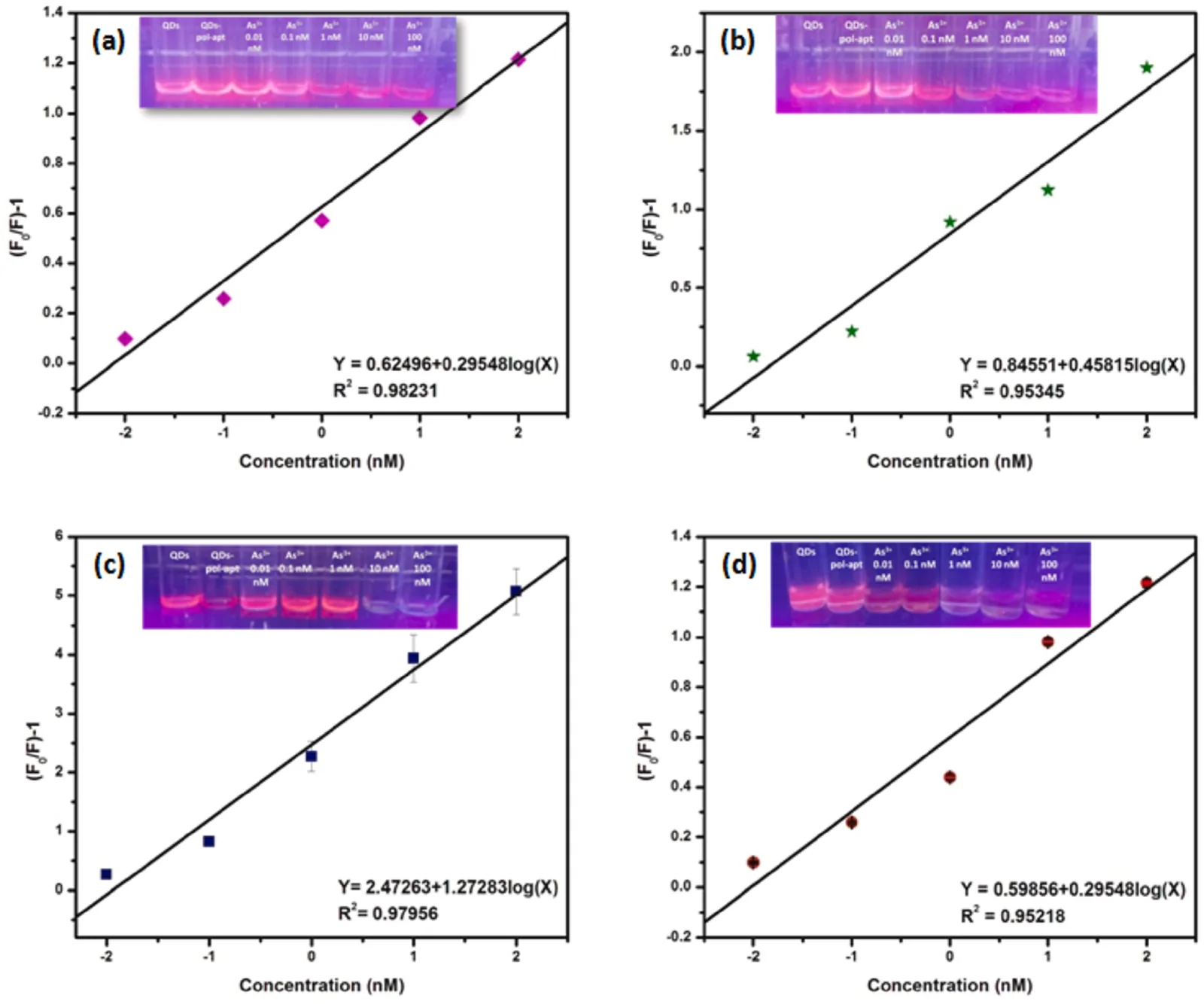
Calibration plots illustrating the fluorescence response to varying As^3+^ concentrations in different real matrices, including mineral water (a), soil water (b), tap water (c), and apple juice (d). Insets depict the corresponding changes in QD fluorescence intensity under UV illumination.^319^

Adegoke and Daeid developed an OFF–ON hybrid plasmonic–fluorometric nanobiosensor combining cadmium-free AuFeZnSe alloyed QDs with gold nanorods (AuNRs) for As(iii) detection, with QD emission (485 nm) initially suppressed *via* nano-metal surface energy transfer to the AuNRs.^320^ A thiolated As(iii)-selective DNA aptamer confers target specificity; arsenic binding induces localized surface plasmon resonance in the AuNRs, enhancing radiative QD decay and restoring fluorescence with approximately 20-fold signal amplification relative to non-plasmonic aptamer-QD controls. The sensor achieved a LOD of 0.01 µg L^−1^ (69.12 pM) across a 0.01 to 100 µg L^−1^ linear range, substantially exceeding WHO drinking-water sensitivity requirements, with high selectivity for As(iii) over As(v) and other coexisting ions *via* aptamer-mediated recognition. The cadmium-free alloyed QD composition addresses toxicity concerns associated with conventional II–VI semiconductor systems. Recovery in blood and river water ranged 93 to 105%. Performance is highly pH-dependent, with optimal operation near neutral conditions, limiting deployment in highly acidic or alkaline wastewater.

#### 5.5.1. Critical analysis

QD-based sensors for arsenic detection have proliferated in recent years, driven by the urgent need for field-deployable alternatives to laboratory-based techniques for monitoring this globally distributed carcinogen. As documented in Table 18, developments showcase diverse material platforms, from aptamer-functionalized CdTe@CdS core–shell structures to plasmonic nanocomposites and CD hybrids, each claiming exceptional sensitivity, with limits of detection ranging from sub-picomolar to low parts-per-billion concentrations.^319^ These platforms invoke increasingly sophisticated sensing modalities, including competitive aptamer displacement, redox-triggered fluorescence recovery, and LSPR amplification. However, despite these ostensible advances, the field exhibits critical deficiencies that severely limit translational potential, inadequate speciation discrimination in chemically complex arsenic systems, reliance on biomolecular recognition elements with unvalidated stability, mechanistic interpretations lacking rigorous physicochemical validation, and real-sample testing protocols that fail to capture the matrix complexity and temporal dynamics of arsenic-contaminated waters.

**Table 18:** Comparative characteristics and analytical performance of QD-based arsenic sensors

Nanomaterial/sensing platform	Synthesis method	Doping/functionalization	Detection technique	Target analyte	Sensing mechanism	Linear detection range	LOD	Selectivity/interfering ions	Optimal working conditions	Real-sample validation (recovery)	Key advantages	Limitations
MPA-capped CdTe@CdS QDs and poly(HPMA-*s*-GPMA) copolymer^319^	Hot injection aqueous synthesis (QDs); aRAFT polymerization (copolymer)	2-MPA; guanidinium-bearing copolymer	Fluorescence	As(iii)	Competitive “on–off” strategy; analyte binds to aptamer, releasing copolymer to quench QD fluorescence *via* electrostatic aggregation	0.01–100 nM	249.77 pM	Hg^2+^, Cd^2+^, Na^+^, Pb^2+^, Cu^2+^, Ni^2+^, Zn^2+^, Mg^2+^, Co^2+^, Fe^3+^, Ca^2+^, Al^3+^, Cr^3+^, Au^3+^, Bi^3+^	pH 7.0; 1.0 M Tris buffer; room temperature	Tap (91.87–101.03%), mineral (91.41–99.89%), soil water (107.92–108.72%); apple juice (73.39–102.11%)	Picomolar sensitivity; cost-effective; label-free; high specificity *via* aptamer	Specific incubation times (up to 60 min) required
Manganese dioxide-CD (MnO_2_CD) composite^319^	One-step hydrothermal decomposition	Polydopamine and citric acid precursors; MnO_2_ shell	Fluorescence	As(iii), DMA, DMDTA, DMMTA	Signal-on (turn-on); redox-mediated shell disintegration halts FRET, restoring CD fluorescence	0–100 nM (As^3+^)	5.75 nM (0.76 ppb)	Cu^2+^, Pb^2+^, Cd^2+^, Ca^2+^, Cr^3+^, Ba^2+^, Hg^2+^, Co^2+^, K^+^, NO_3_^−^, SO_4_^2−^, ClO_4_^−^, Cl^−^, Br^−^, PO_4_^3−^	pH 5.0–9.0; high salinity stability (2.5 M NaCl)	Tap (101.16–103.0%), pond (99.37–103.25%), river (100.41–101.87%), sea water (99.0–100.66%)	Turn-on signal; reversible temperature sensing; first *in vivo* plant imaging of arsenic	Not reported
l-Methionine (Meth)-capped PbS QDs^318^	One-pot aqueous chemical approach	l-Methionine capping	Fluorescence	As(iii)	Turn-off (static quenching); ligand stripping and QD aggregation *via S*,*N*-bidentate chelate formation	5–150 ppb	3.7 ppb	Na^+^, K^+^, Li^+^, Ni^2+^, Co^2+^, Cu^2+^, Ca^2+^, Mg^2+^, Fe^2+^, Fe^3+^, Zn^2+^, Cr^2+^, Cr^3+^, As(v), Al^3+^, Ba^2+^, Sr^2+^, Pb^2+^	pH 7.4; 3 min contact time; room temperature	Tap water (96.6–100.2%); river water (99.2–100.8%)	Simple aqueous synthesis; no organic solvents; rapid 3-min response; high storage stability (4 months)	Sensitivity to ligand protonation in acidic media and hydroxide formation at pH > 8
AuFeZnSe alloyed QDs @ gold nanorod (AuNR) nanocomposite^320^	Hot-injection organometallic pyrolysis (QDs); seed-mediated approach (AuNRs)	Amphiphilic polymer (Amp-P); thiolated anti-As(iii) DNA aptamer	Fluorescence (OFF–ON)	As(iii)	LSPR-amplified fluorescence; NSET-based quenching (OFF) followed by plasmonic signal amplification upon analyte binding (ON)	0.01–100 µg L^−1^	0.01 µg L^−1^ (69.12 pM)	Zn^2+^, Mg^2+^, Co^2+^, Fe^2+^, Ag^+^, Ca^2+^, Hg^2+^, Ni^2+^, Ba^2+^, Cu^2+^, Pb^2+^, Na^+^, K^+^, As(v)	pH 7.0; MES-Trisma acetate buffer	Cow blood (∼95.8–104.9%); river water (∼93.1–103.4%)	Ultra-high sensitivity (1000× below WHO limit); cadmium-free alloyed QDs; high signal contrast	Sensitivity to chemical environment (pH fluctuations)

The speciation selectivity problem in arsenic sensing represents a far more intractable challenge than for cadmium or chromium yet receives remarkably superficial treatment in the reviewed literature. As detailed in Table 18, three of four sensors explicitly target As(iii), while Behera *et al.* claim broader recognition encompassing As(iii), dimethylarsenate (DMA), and thioarsenates DMDTA and DMMTA.^317^ This speciation bias toward As(iii) reflects practical analytical considerations, As(iii) exists predominantly as the uncharged H_3_AsO_3_ molecule near neutral pH, enabling hydrophobic partitioning and soft-donor (sulfur, nitrogen) coordination that are exploited by surface-functionalized QDs. Conversely, As(v) exists as oxyanions (H_2_AsO_4_^−^, HAsO_4_^2−^) that interact weakly with most QD surface chemistries and require electrostatic preconcentration or ion-exchange mechanisms rarely implemented in fluorescence-based platforms. However, this analytical convenience obscures a critical environmental reality, natural waters contain dynamic mixtures of As(iii) and As(v) whose relative proportions fluctuate with redox conditions, microbial activity, photochemical processes, and seasonal variations. Reporting As(iii)-selective measurements as representative of “arsenic contamination” without concurrent As(v) determination yields incomplete and potentially misleading risk assessments, as total arsenic, not individual species, governs regulatory compliance in most jurisdictions. More troubling, none of the reviewed sensors demonstrate true speciation capability through differential response mechanisms; instead, they exploit thermodynamic or kinetic selectivity that may collapse under environmentally realistic conditions. For instance, Rahimi *et al.* claim As(iii) selectivity over As(v) based on bidentate chelation through methionine's sulfur and nitrogen donors, yet provide no binding constant measurements, no demonstration of selectivity under varying As(iii):As(v) ratios, and no assessment of whether As(v) gradually displaces surface ligands over extended exposure periods.^318^ Behera *et al.*'s broader arsenic species recognition, while conceptually valuable, lacks quantitative differentiation: the MnO_2_CD platform reportedly responds to DMA, DMDTA, and DMMTA through the same MnO_2_ reduction mechanism that detects As(iii), providing no pathway to distinguish these species or measure their individual concentrations.^317^ This fundamental limitation, sensors that detect multiple arsenic species but cannot differentiate them, severely restricts their utility for understanding arsenic biogeochemistry, optimizing treatment processes, or assessing species-specific health risks.

Aptamer-based recognition, employed by Soni *et al.*^319^ and Adegoke *et al.*,^320^ introduces additional layers of complexity and vulnerability that are inadequately characterized. DNA aptamers selected for As(iii) binding offer theoretical advantages over small-molecule ligands, higher binding specificity through three-dimensional structural complementarity, rational sequence engineering for affinity tuning, and biocompatibility for potential *in vivo* applications. However, these benefits depend critically on maintaining aptamer tertiary structure, which is exquisitely sensitive to ionic strength, pH, temperature, and the presence of nucleases, conditions that vary dramatically in real environmental samples. Soni *et al.*'s competitive displacement strategy, wherein As(iii) binding to the aptamer releases a guanidinium-bearing copolymer that subsequently quenches QD fluorescence, achieved impressive 249.77 pM sensitivity but required precise control of Tris buffer concentration (1.0 M) and extended incubation times up to 60 minutes.^319^ These operational constraints raise fundamental questions about field applicability: natural waters exhibit ionic strengths from <0.001 M (rainwater) to >0.5 M (brackish groundwater), potentially destabilizing aptamer secondary structure or altering copolymer-QD electrostatic interactions in unpredictable ways. Moreover, the study provides no data on aptamer stability during storage, resilience to nuclease contamination from environmental microorganisms, or signal drift during prolonged field deployment. Adegoke *et al.*'s thiolated anti-As(iii) aptamer, immobilized on gold nanorods for LSPR-enhanced sensing, achieved even lower LODs (69.12 pM) but operates through an “OFF–ON” mechanism whose physicochemical basis remains poorly defined.^320^ The claim that aptamer-As(iii) binding reverses nanoenergy transfer (NSET)-mediated quenching to restore fluorescence requires validation through distance-dependent energy transfer measurements, aptamer conformational studies, and demonstration that signal recovery specifically correlates with As(iii) binding rather than nonspecific environmental perturbations. Critically, neither aptamer-based sensor benchmarks performance against conventional ligand-based approaches using identical testing protocols, preventing objective assessment of whether the added complexity of biological recognition elements translates into meaningful analytical advantages.

Mechanistic ambiguity pervades the field, with proposed sensing mechanisms ranging from static quenching and ligand displacement to FRET modulation and plasmonic enhancement, yet experimental validation remains largely correlative rather than definitive. Rahimi *et al.* attribute As(iii)-induced fluorescence quenching of methionine-capped PbS QDs to “ligand stripping and QD aggregation *via S*,*N*-bidentate chelate formation”, supported by transmission electron microscopy showing particle clustering.^318^ However, this interpretation conflates two distinct processes, surface ligand exchange (a molecular-scale event) and NP aggregation (a colloidal phenomenon), without establishing their causal relationship or temporal sequence. Static quenching, as claimed, should produce linear Stern–Volmer plots and concentration-independent fluorescence lifetimes, neither of which are reported. Alternative explanations, As(iii)-induced oxidative etching of PbS surfaces, electron transfer quenching, or inner-sphere charge transfer complex formation, remain unexplored. The absence of time-resolved photoluminescence spectroscopy, XPS to confirm As-QD binding modes, or isothermal titration calorimetry to quantify binding thermodynamics leaves the “mechanism” as phenomenological description rather than molecularly validated understanding. Behera *et al.*'s MnO_2_CD composite presents an equally complex system wherein As(iii) reduction of the MnO_2_ shell terminates FRET from CDs to MnO_2_, restoring CD emission.^317^ While conceptually elegant, this “turn-on” mechanism introduces multiple uncontrolled variables: the stoichiometry of As(iii) oxidation to As(v), the kinetics of MnO_2_ dissolution, potential catalytic cycling that could amplify or dampen signal, and the fate of generated Mn^2+^ ions (which themselves might quench fluorescence). The study provides no evidence distinguishing whether DMA, DMDTA, and DMMTA detection proceeds through identical MnO_2_ reduction pathways or involves alternative mechanisms, nor does it explain why these methylated and thioarsenate species, with drastically different reduction potentials, yield comparable sensor responses. Most critically, the claimed pH stability across pH 5.0–9.0 and salinity tolerance up to 2.5 M NaCl, while impressive for CD fluorescence retention, do not guarantee that the As(iii)–MnO_2_ redox reaction kinetics and selectivity remain constant across these conditions.

Plasmonic enhancement strategies, exemplified by Adegoke *et al.*'s AuFeZnSe QD@AuNR nanocomposite, represent the cutting edge of QD sensor design but introduce extraordinary mechanistic and synthetic complexity that undermines reproducibility.^320^ The system integrates four distinct nanomaterials, quaternary alloyed QDs (AuFeZnSe) synthesized *via* high-temperature organometallic pyrolysis, gold nanorods grown through seed-mediated approaches with aspect-ratio-dependent LSPR properties, amphiphilic polymer phase-transfer agents, and sequence-specific DNA aptamers. Each component contributes to the overall sensing response through mechanisms operating at different length scales, aptamer-As(iii) binding (molecular), NSET between QDs and AuNRs (1–10 nm), and LSPR field enhancement (10–50 nm). Disentangling these coupled phenomena to establish which dominates the analytical signal, and under what conditions, requires systematic studies varying each parameter independently, experiments not performed in the published work. The claim of 1000-fold sensitivity enhancement over non-plasmonic systems lacks quantitative justification, LSPR enhancement factors depend exquisitely on QD-AuNR spacing, spectral overlap between QD emission and AuNR plasmon resonance, and local refractive index, yet none of these parameters are systematically characterized or controlled. More fundamentally, the synthesis requires two multi-step procedures (QD and AuNR preparation) followed by aptamer conjugation and QD-AuNR assembly, a workflow vulnerable to batch-to-batch variations at each stage. The study reports no statistical analysis of synthesis reproducibility, no quantification of QD-to-AuNR stoichiometry in the final nanocomposite, and no assessment of nanocomposite stability during storage or repeated use cycles. Given that hot-injection QD synthesis exhibits notorious sensitivity to heating rates, ligand ratios, and precursor purity, while seed-mediated AuNR growth produces aspect ratio distributions that critically affect LSPR properties, expecting consistent analytical performance from such a complex assembly border on optimistic.

Real-sample validation protocols reveal systematic deficiencies that call into question the environmental relevance of reported performance metrics. Recovery studies, universally employed across all four sensors, exhibit troubling inconsistencies and inadequacies. Soni *et al.* report recovery ranges of 91.87–101.03% (tap water), 91.41–99.89% (mineral water), 107.92–108.72% (soil water), and 73.39–102.11% (apple juice), with the soil water and apple juice results falling far outside acceptable analytical ranges.^319^ A recovery of 107–108% in soil water suggests either matrix enhancement effects, co-extraction of reducing agents that partially reduce As(v) to As(iii) during sample preparation, or baseline arsenic contamination that was not properly quantified before spiking. Even more concerning, the 73% recovery in apple juice indicates severe matrix interference, likely from ascorbic acid, polyphenols, or pectins, that is neither explained nor addressed through sample pretreatment. Behera *et al.*'s validation across tap, pond, river, and seawater (recoveries 99.0–103.25%) appears superficially robust, yet the study provides no information on background arsenic levels, dissolved organic carbon concentrations, iron and manganese content (both of which could reduce MnO_2_ independently of As(iii)), or the stability of MnO_2_CD in these matrices over the measurement timescale.^317^ The remarkable claim of seawater applicability deserves particular scrutiny: marine waters contain millimolar concentrations of bromide, iodide, and sulfide (in anoxic basins) that are strong reducing agents potentially capable of nonspecific MnO_2_ reduction. Without concurrent blank measurements, standard addition validation, or comparison against certified reference seawater, these recovery values provide limited confidence. Rahimi *et al.*'s validation in tap and river water (96.6–100.8% recovery) meets analytical acceptability criteria but tested only two matrix types, both freshwater, and provides no data on sample pretreatment, filtration cutoffs, or preservation methods, critical omissions given that particulate arsenic, colloidal species, and biological transformations can dramatically alter speciation during storage.^318^ Most critically, none of the studies perform temporal stability assessments: arsenic speciation in water samples changes within hours due to microbial activity and oxidation, yet all validation protocols assume static, stable arsenic concentrations during extended measurement periods (up to 60 minutes for Soni *et al.*).^319^

The absence of head-to-head comparisons with established analytical techniques represents a glaring validation gap that pervades the arsenic QD sensor literature. While all four studies claim superior or comparable performance to existing methods, none provide direct comparative measurements on identical samples using validated reference techniques such as ICP-MS, HGAAS, or anodic stripping voltammetry. This omission is particularly problematic for sensors claiming ultra-low LODs (249.77 pM for Soni *et al.*,^319^ 69.12 pM for Adegoke *et al.*^320^) that approach or exceed the sensitivity of standard laboratory methods. Without parallel analysis of real samples by both QD sensors and certified reference methods, reported recoveries cannot distinguish between accurate quantification and coincidental agreement. Furthermore, the regulatory acceptance of any new analytical technique requires interlaboratory validation studies demonstrating reproducibility across different operators, instruments, and environmental matrices, a standard that no QD-based arsenic sensor has approached. The reliance on spiked recovery studies, rather than analysis of certified reference materials with known arsenic concentrations and speciation, represents a fundamental departure from established validation protocols in environmental analytical chemistry.

Operational constraints and practical deployment barriers receive minimal attention despite representing major translational hurdles. Incubation time requirements ranging from 3 minutes to 60 minutes present significant challenges for high-throughput screening or continuous monitoring applications.^318^ While 3 minutes appears reasonable for grab sampling, it far exceeds the response time of electrochemical sensors already deployed for field arsenic testing (typically <30 seconds), negating claimed advantages of optical detection. The 60-minute requirement for aptamer-based systems renders them unsuitable for any scenario requiring rapid decision-making; emergency response, real-time treatment optimization, or point-of-use testing in resource-limited settings. Moreover, these timescales assume controlled laboratory conditions; field deployment introduces temperature fluctuations, sample turbidity, and operator variability that may significantly extend equilibration times. Buffer requirements present another practical limitation frequently glossed over: Soni *et al.* require 1.0 M Tris buffer, Adegoke *et al.* specify MES-Trisma acetate buffer,^320^ and Rahimi *et al.* operate at pH 7.4, all conditions requiring sample pH adjustment and buffer addition that increase cost, complexity, and potential for contamination.^319^ The claim by Behera *et al.* of pH 5.0–9.0 operational range represents significant progress, yet even this range excludes acidic mine drainage and alkaline industrial effluents where arsenic contamination is prevalent.^317^ None of the sensors address sample turbidity, a ubiquitous characteristic of natural waters that scatters excitation and emission light, potentially causing both false positives (light scattering detected as fluorescence) and false negatives (attenuation of signal). The absence of built-in turbidity correction, internal standards, or ratiometric referencing represents a critical oversight for sensors intended for field deployment.

Photostability and sensor reusability, while essential for practical deployment, receive only cursory attention. Rahimi *et al.* report 4-month storage stability for PbS QDs, a valuable finding, yet provide no data on fluorescence stability under continuous illumination, the operational mode for most fluorescence-based sensors.^318^ CDs, employed by Behera *et al.*, are generally more photostable than semiconductor QDs but still undergo gradual photooxidation, particularly in oxygenated aqueous environments, leading to blue-shifted emission and QY degradation.^317^ The lack of accelerated photostability testing (continuous illumination at elevated temperature) or quantification of photobleaching rates under realistic measurement conditions leaves operational lifetime entirely uncertain. Claims of sensor reversibility or reusability are notably absent: all sensors appear to function as single-use or limited-reuse devices, with no discussion of regeneration protocols, signal recovery after analyte binding, or quantitative assessment of signal drift over multiple measurement cycles. This limitation contrasts sharply with electrochemical arsenic sensors, many of which offer hundreds of measurements per electrode, and represents a significant economic and waste-generation concern for deployment in resource-limited settings.

Interference from environmentally relevant species, while ostensibly addressed through selectivity studies, remains incompletely characterized. All four sensors report tolerance toward a broad panel of metal ions, yet these selectivity assessments follow the standard but inadequate protocol of sequential single-ion addition at equimolar or modest excess concentrations relative to arsenic. Real groundwater contains complex mixtures where calcium and magnesium (0.1–10 mM) exceed arsenic (0.1–10 µM) by three to four orders of magnitude, iron and manganese (1–1000 µM) by two to three orders, and phosphate (1–100 µM) by one to three orders. Phosphate presents a particularly vexing interference: as an oxyanion with similar charge and geometry to arsenate, it competes for surface binding sites, while its ubiquitous presence in natural waters (from agricultural runoff and wastewater discharge) ensures it will be present in virtually all field samples. Behera *et al.* list PO_4_^3−^ among tested interferents but provide no quantitative data on response suppression at environmentally relevant phosphate:arsenic ratios.^317^ Dissolved organic matter, present in surface waters at 1–50 mg L^−1^ as dissolved organic carbon, presents another major interference pathway rarely addressed: humic and fulvic acids contain thiol, carboxyl, and phenolic groups that coordinate metal ions, potentially sequestering As(iii) in non-bioavailable complexes that do not interact with QD sensors. The absence of selectivity testing in the presence of natural organic matter isolates, or using certified reference water samples with defined matrix complexity, represents a critical gap in validation rigor.

Synthesis reproducibility and scalability constraints mirror those identified for cadmium and chromium sensors but are compounded by the multi-component nature of arsenic sensing platforms. Soni *et al.*'s system requires three separately synthesized components: MPA-capped QDs *via* hot injection, guanidinium-bearing copolymer *via* reversible addition–fragmentation chain transfer (RAFT) polymerization, and DNA aptamer either through commercial purchase or oligonucleotide synthesis.^319^ Each synthesis step introduces variability, QD size distribution and QY, copolymer molecular weight and polydispersity, aptamer purity and secondary structure, that propagates into the assembled sensor's analytical performance. The study provides no statistical analysis of inter-batch reproducibility, no quantification of component-to-component stoichiometric variations, and no assessment of sensor-to-sensor performance variations when assembled from different synthesis batches. Adegoke *et al.*'s AuFeZnSe@AuNR nanocomposite presents even greater complexity: quaternary alloyed QDs require precise control of four metal precursor ratios, reaction temperature, and ligand stoichiometry to achieve the desired composition and optical properties; gold nanorod aspect ratios (which determine LSPR wavelength) vary with seed concentration, growth solution composition, and aging time; and final nanocomposite assembly depends on aptamer surface density, QD-AuNR ratio, and amphiphilic polymer concentration.^320^ The probability of achieving consistent analytical performance across batches produced by different operators, in different laboratories, using different reagent lots approaches zero without rigorous standardization protocols, none of which are reported. Economic considerations are similarly absent: no cost-per-test analysis, no comparison with commercially available arsenic test kits (such as those based on the Gutzeit reaction or field arsenic test strips), and no projection of manufacturing costs at scales required for widespread deployment.

Environmental and toxicological impacts of QD-based sensors themselves constitute an underexplored concern with particular relevance to arsenic detection, where sensor deployment targets the same aquatic environments vulnerable to contamination. Soni *et al.*^319^ and Adegoke *et al.*^320^ both employ cadmium-containing QDs (CdTe@CdS and Cd-free alloyed QDs, respectively), with Adegoke *et al.* explicitly highlighting the cadmium-free nature of AuFeZnSe as an advantage. However, “cadmium-free” does not equate to “environmentally benign”: selenium, zinc, iron, and gold all pose ecotoxicity concerns at sufficient concentrations, while gold nanorods have documented cytotoxicity linked to aspect ratio and surface chemistry. Behera *et al.*'s MnO_2_CD composite, while based on relatively benign carbon and manganese, undergoes deliberate MnO_2_ dissolution during sensing, releasing Mn^2+^ into the sample matrix, a potential concern for sensors deployed in drinking water or used for food testing (apple juice).^317^ Rahimi *et al.*'s PbS QDs present the most obvious environmental hazard: lead toxicity rivals or exceeds that of arsenic, making deployment of lead-based sensors to detect arsenic contamination ethically questionable and potentially illegal under many environmental regulations.^318^ None of these studies conduct leaching assessments, measure QD release rates under environmental conditions, or perform ecotoxicity testing on aquatic organisms, standard requirements for nanomaterials intended for environmental release or water contact.

Advancing QD-based arsenic sensors beyond their current proof-of-concept status toward genuine field deployment demands a fundamental reorientation of research priorities. First and most critical, speciation-capable platforms that independently quantify As(iii), As(v), and key methylated/thioarsenated species must be developed, potentially through multiplexed sensor arrays combining multiple recognition chemistries or integration with pre-separation techniques such as solid-phase extraction cartridges or ion-exchange columns. Second, aptamer-based systems require comprehensive stability validation including nuclease resistance testing, performance across realistic ionic strength ranges (0.001–1.0 M), temperature cycling (5–40 °C), and shelf-life assessments under field storage conditions. Third, mechanistic validation using advanced spectroscopic techniques, time-resolved fluorescence spectroscopy, X-ray absorption spectroscopy to confirm arsenic oxidation states and binding modes, surface-enhanced Raman spectroscopy to monitor real-time QD-arsenic interactions, must become standard practice. Fourth, real-sample validation protocols must advance beyond spiked recovery studies to include analysis of certified reference materials, head-to-head comparisons with ICP-MS or HGAAS on identical field samples, and interlaboratory studies establishing method precision and accuracy. Fifth, operational robustness testing, photostability under continuous illumination, performance degradation over repeated use cycles, quantification of interference from dissolved organic matter and high-abundance ions at environmentally realistic ratios, must be systematically conducted and reported. Sixth, synthesis standardization through statistical process control, continuous-flow microfluidic production platforms, and quality control metrics (QY, size distribution, functional group density) is essential for commercialization. Seventh, techno-economic analysis comparing cost-per-test with existing field methods (commercial test strips, portable XRF, handheld electrochemical analyzers) and life-cycle environmental impact assessment of sensor materials must inform development priorities. Finally, regulatory pathway engagement, including submission of validation packages to relevant agencies (EPA, FDA for blood testing applications) and alignment of performance targets with regulatory monitoring requirements, should occur early in development rather than as an afterthought. Only through such comprehensive, translational research can QD-based arsenic sensors evolve from materials science demonstrations into practical tools addressing one of the world's most pressing environmental health challenges.

### 5.6. Detection of other metals

While the majority of QD-based sensor development has concentrated on the “priority pollutant” heavy metals, cadmium, chromium, arsenic, lead, and mercury, mandated for regulatory monitoring under frameworks such as the U.S. EPA Priority Pollutant List and the European Water Framework Directive, a growing body of research addresses the detection of additional metal ions whose environmental, industrial, and biological significance warrants analytical attention. These “other metals” encompass a diverse array of elements with distinct toxicological profiles, environmental occurrence patterns, and analytical challenges: antimony (Sb^3+^), a metalloid with applications in flame retardants and semiconductor alloys that exhibits carcinogenic and immunotoxic effects; manganese (Mn^2+^ and Mn^7+^), an essential micronutrient that becomes neurotoxic at elevated exposures and appears as permanganate oxidant in industrial processes; cobalt (Co^2+^), a component of vitamin B_12_ that poses occupational hazards in mining and battery manufacturing; zinc (Zn^2+^), simultaneously an essential trace element and a toxic contaminant at high concentrations in aquatic ecosystems; and nickel (Ni^2+^), a widespread industrial metal and common allergen with established carcinogenic potential.

The analytical detection of these metals presents challenges that differ substantially from those encountered with the extensively studied cadmium–chromium–arsenic–lead–mercury group, primarily due to their distinct coordination chemistries, redox behaviors, and the frequent necessity to discriminate among multiple oxidation states with divergent toxicological properties. Antimony's speciation between trivalent Sb(iii) and pentavalent Sb(v) mirrors arsenic's complexity, with the trivalent form exhibiting significantly greater acute toxicity and environmental mobility.^321^ Manganese exists in oxidation states ranging from +2 to +7, with permanganate (Mn^7+^) serving as a powerful oxidizing agent in water treatment that requires monitoring to prevent excessive residuals, while Mn^2+^ accumulation in drinking water distribution systems causes aesthetic problems and potential neurodevelopmental effects.^322^ Cobalt's biological essentiality as the metallic center in vitamin B_12_ necessitates sensors capable of operating in complex biological matrices, while its increasing use in lithium-ion batteries creates new exposure pathways requiring environmental monitoring.^323^ Zinc presents the dual challenge of being both nutritionally essential and ecotoxic, demanding sensors with wide dynamic ranges spanning deficiency to toxicity thresholds.^324^

The QD platforms developed for these “other metals”, while often employing similar core materials and surface chemistries as those designed for priority pollutants, exhibit distinctive sensing mechanisms tailored to exploit specific metal–ligand coordination preferences, redox reactivities, or photophysical interactions. Strategies include protein-mediated recognition (BSA-functionalized QDs for antimony), fluorescence turn-on mechanisms based on surface defect passivation (Zn^2+^-induced enhancement), oxidation-reduction sensing for multiple manganese oxidation states, and chiral recognition elements for enantioselective detection. These approaches reflect both the maturation of QD sensor design principles and the persistent challenges of achieving selectivity in complex matrices where target analytes may be present alongside structurally similar interferents or at concentrations orders of magnitude lower than major matrix components. However, the relative scarcity of research attention devoted to these metals, compared to the extensively studied priority pollutants, results in less comprehensive validation, limited inter-laboratory reproducibility data, and minimal advancement toward commercial deployment or regulatory acceptance. The following section surveys representative developments in QD-based sensors for antimony, manganese, cobalt, zinc, and nickel, highlighting innovative detection mechanisms while critically examining their analytical performance, real-sample validation, and translational potential within the broader context of heavy metal monitoring technologies.

Ge *et al.* developed a receptor-fluorophore nanosensor for Sb^3+^ using thioglycolic acid-stabilized CdTe QDs functionalized with bovine serum albumin (BSA) *via* EDC/NHS coupling, where BSA acts as both recognition element and fluorescence enhancer through amino-acid-mediated charge transfer.^321^ Sb^3+^ coordination to BSA's nitrogen and oxygen residues disrupts this charge transfer, quenching fluorescence. Selectivity against Pb^2+^, As^3+^, Hg^2+^, and other transition-metal cations relies on BSA's intrinsic Sb^3+^ affinity. The sensor achieved a LOD of 2.94 × 10^−8^ g L^−1^ across a 0.10 to 22.0 mg L^−1^ linear range, with a 5-minute response time. Validation in artificial pond water and domestic sewage required chemical pre-reduction to convert Sb^5+^ to detectable Sb^3+^. A narrow neutral pH window is required, as acidic conditions cause receptor protonation and alkaline conditions promote antimony precipitation.

Mohagheghpour *et al.* developed histidine-modified CDs *via* hydrothermal carbonization of sodium citrate and histidine, with histidine serving as both passivating agent and recognition ligand, for CHEF-based Mn^2+^ detection (534 nm emission enhancement).^322^ DFT calculations showed Mn^2+^ preferentially adopts tetrahedral coordination with the histidine surface, unlike competing ions favoring square-planar arrangements; optimal performance occurs at alkaline pH 10.0. The sensor achieved a LOD of 1.85 µg L^−1^ across a 3.50 to 35.5 µg L^−1^ linear range. The signal-on mode mitigates false positives from nonspecific quenching. Recovery in whole blood control samples exceeded 97%, supporting clinical and point-of-care applications. Strong alkaline pH dependence and partial Co^2+^ interference are limitations requiring careful sample control.

Deng *et al.* developed l-cysteine-functionalized, manganese-doped ZnS QDs for Mn(vii) detection *via* long-lived phosphorescence (^4^T_1_ to ^6^A_1_ Mn^2+^ transition), enabling time-resolved readout that suppresses autofluorescence without oxygen removal.^325^ MnO_4_-oxidizes surface thiol groups to sulfite species, causing ligand detachment and defect-mediated quenching; selectivity relies on Mn(vii)'s distinct oxidative reactivity *versus* other anions and transition-metal ions. The sensor achieved a LOD of 0.24 µM across a 0.5 to 100 µM linear range, with a 10-minute response time. Compared to cadmium-based probes, this system offers larger Stokes shifts and improved interference resistance. Recovery in tap and pond water ranged 95.3 to 106.2%. Performance requires near-neutral to mildly alkaline pH (∼8.0), as acidic conditions promote surface dissolution and intrinsic quenching.

Li *et al.* developed a label-free assay using l-cysteine-derived CDs (∼3.6 nm) with intrinsic thiol, carboxyl, and amino functionality for Co^2+^ detection *via* Co_*x*_S_*y*_ formation and subsequent aggregation-induced quenching.^323^ Selectivity relies on cysteine–Co^2+^ coordination affinity, absent in CDs made from glycine or EDTA. The sensor achieved a LOD of 5 nM across a 10 nM to 100 µM linear range, an improvement over thioglycolic acid-capped CdTe and Mn-doped ZnS QD sensors. Validation in vitamin B12 formulations and lake/stream water agreed closely with ICP-MS. Minor matrix effects slightly raised the effective LOD in environmental samples, and mildly basic conditions (pH ∼ 9.0) are required.

Geng *et al.* developed β-cyclodextrin-functionalized ZnO QDs (β-CD@ZnO, yellow-green emission at 537 nm) *via* modified sol–gel synthesis, a low-toxicity cadmium-free alternative for Co^2+^ detection *via* host-guest-mediated aggregation-induced quenching (FRET excluded due to lack of spectral overlap).^324^ Selectivity against Fe^3+^, Hg^2+^, and Pb^2+^ relies on β-cyclodextrin host–guest chemistry. The sensor achieved a LOD of 0.34 µM across a 1.0 to 10 µM linear range, with a 3-minute response time. Recovery in spiked tap and lake water ranged 98.2 to 113.0%. A narrow near-neutral pH window (∼7.5) and interference from elevated Cu^2+^ (>1 mM) may require preliminary separation in complex samples. Boonta *et al.* developed nitrogen and sulfur co-doped GQDs (N,S-GQDs) *via* one-step hydrothermal synthesis of citric acid and cysteamine hydrochloride, incorporating amino, carboxyl, and thiol groups without post-synthetic modification, for Co^2+^ detection *via* combined static and aggregation-induced quenching, confirmed by minimal exciton lifetime change and UV-vis absorption shifts.^326^ The sensor achieved a LOD of 1.25 µM across a 0 to 40 µM linear range under alkaline pH 9.0, with a rapid 3-minute response (Fig. 30). The system enabled both quantitative analysis and paper-strip visual screening under UV. Recovery in tap and commercial drinking water ranged 91.2 to 108.2%. Strong alkaline pH dependence limits sensitivity in neutral or acidic environments.

**Fig. 30:**
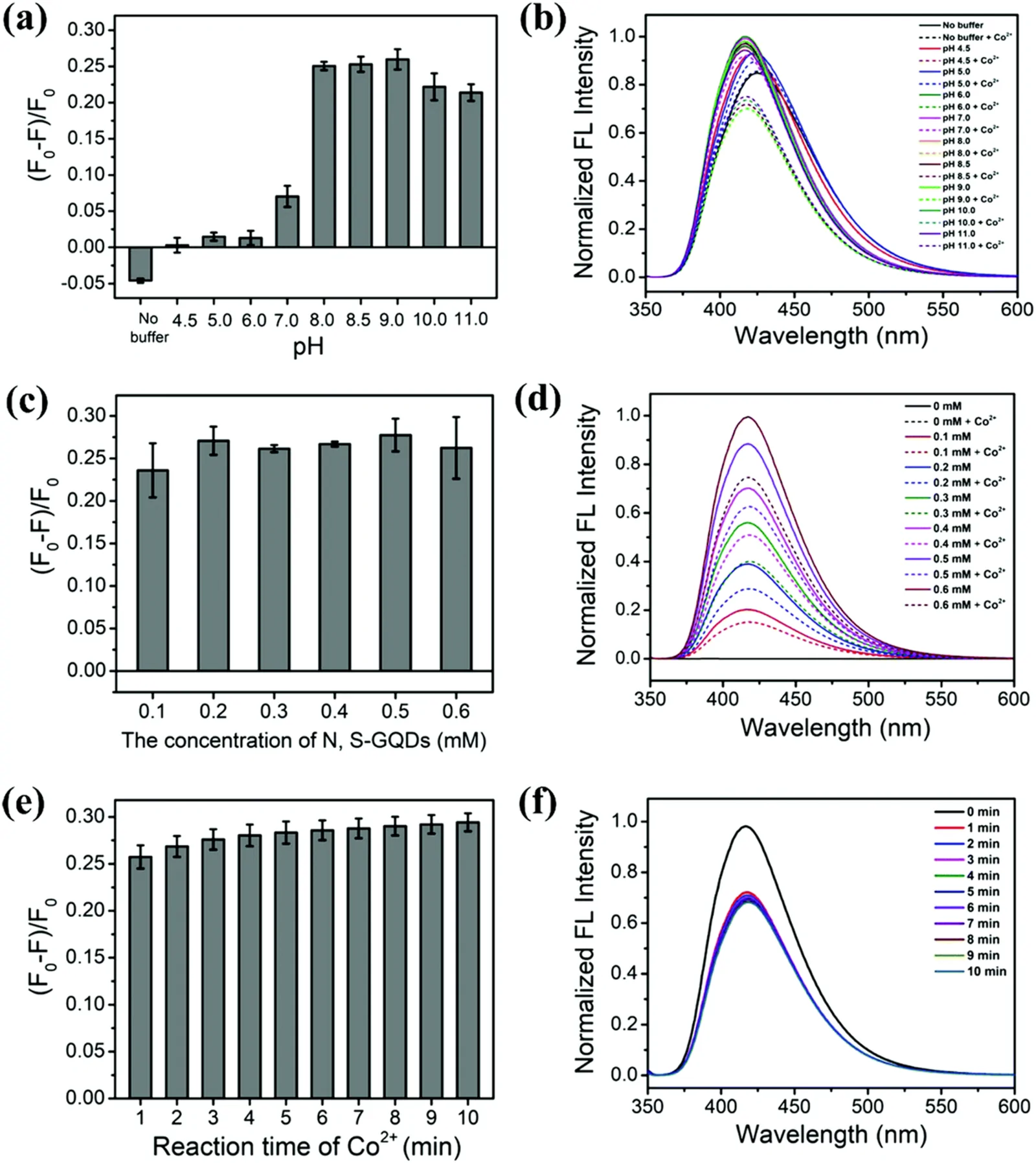
Optimization of Co^2+^ sensing using N,S-GQDs. (a and b) pH-dependent fluorescence quenching behaviour of N,S-GQDs (0.4 mM) in the presence of Co^2+^ (30 µM), demonstrating enhanced quenching efficiency under alkaline conditions due to increased deprotonation of surface functional groups. (c and d) Effect of N,S-GQD concentration on fluorescence quenching in Tris–HCl buffer (50 mM, pH 9.0), illustrating concentration-dependent modulation of the optical response. (e and f) Time-resolved quenching kinetics of N,S-GQDs (0.2 mM) upon Co^2+^ exposure, indicating rapid signal stabilisation within minutes. Corresponding fluorescence emission spectra accompany each dataset, collectively highlighting the optimized sensing conditions and fast response characteristics of the platform.^326^

Wu *et al.* developed the first NIR-II (1.0 to 1.7 µm) fluorescence platform for turn-on Zn^2+^ and Cd^2+^ detection, using water-dispersible, TGA-functionalized Ag_2_S QDs synthesized hydrothermally with ligand exchange.^327^ Unlike conventional quenching probes, signal enhancement arises from surface defect passivation: metal-thiolate shell formation heals surface traps and suppresses nonradiative decay, extending emission lifetime from 3.25 ns (metal-free) to 10.42 ns upon Cd^2+^ binding (Fig. 31). Selectivity for Group IIB ions arises from TGA coordination chemistry, discriminating against Cu^2+^ and Hg^2+^ (which cause quenching) and alkaline earth metals (negligible response). The sensor achieved LODs of 760 nM (Zn^2+^) and 546 nM (Cd^2+^), with a 2-minute response time and emission stability exceeding 1 hour. NIR-II operation minimizes autofluorescence and scattering relative to visible-emitting QD systems, using a low-toxicity, cadmium-free Ag_2_S core. Applications included Cd^2+^ quantification in tap and lake water and real-time Zn^2+^ imaging in living cells. The platform cannot distinguish Zn^2+^ and Cd^2+^ in mixed samples, and operates within a constrained pH window (∼7.4 to 9.0).

**Fig. 31:**
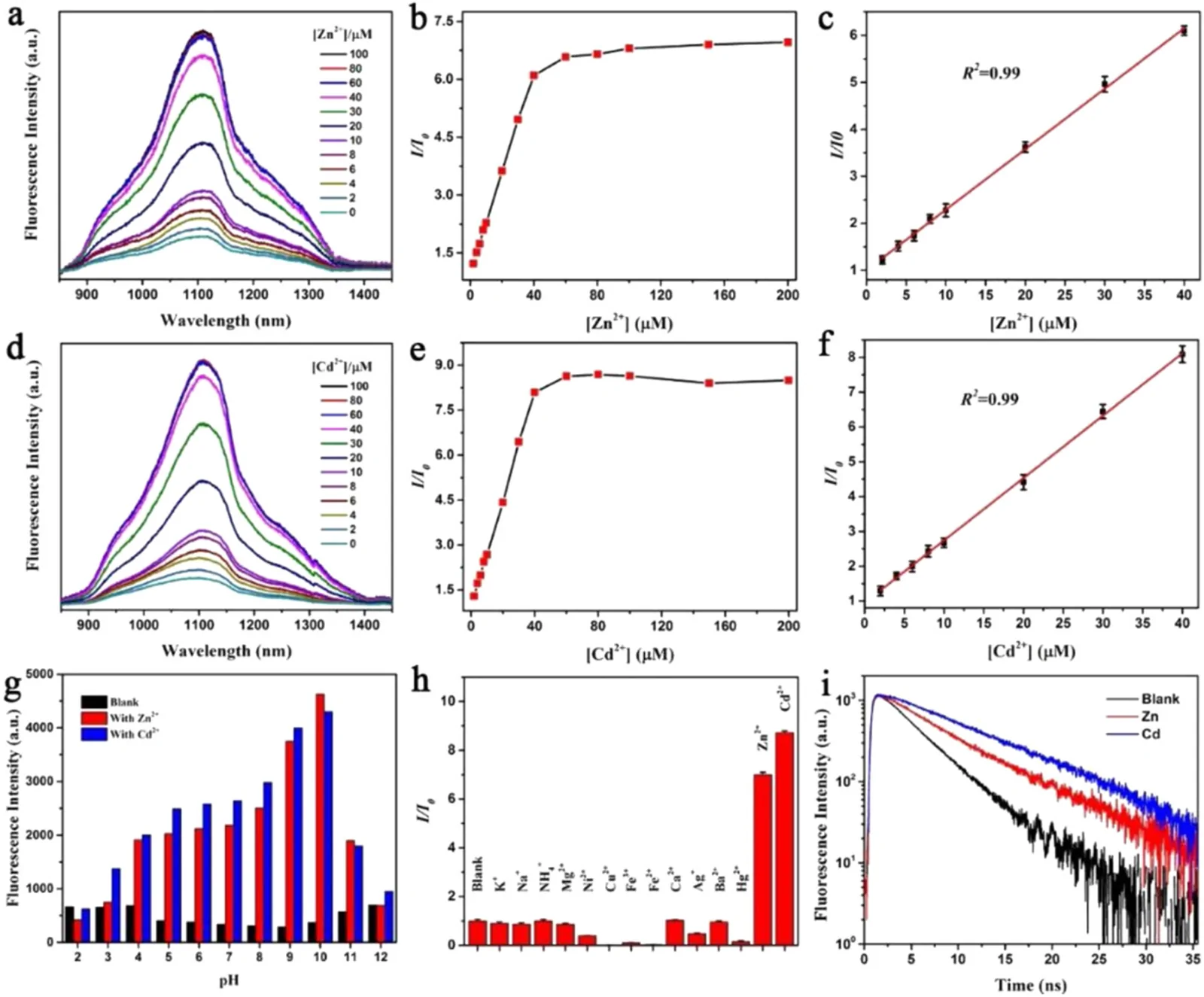
NIR-II fluorescence-based detection of Zn^2+^ and Cd^2+^ using Ag_2_S QDs. (a and d) Progressive enhancement of Ag_2_S QD fluorescence emission with increasing concentrations of Zn^2+^ (a) and Cd^2+^ (d), consistent with metal-induced surface passivation. (b, c, e and f) Linear calibration relationships between normalized fluorescence intensity (*I*/*I*_0_) and Zn^2+^ (b and c) or Cd^2+^ (e and f) concentration, demonstrating quantitative sensing over the relevant analytical range. (g) Effect of solution pH on fluorescence intensity in the absence and presence of Zn^2+^ or Cd^2+^, highlighting the optimal operational window for stable metal–ligand coordination. (h) Selectivity profile of the Ag_2_S QD sensor against competing metal ions, confirming preferential response toward Group IIB cations. (i) Fluorescence lifetime analysis before and after metal ion binding, evidencing suppression of nonradiative decay pathways as the dominant signal-enhancement mechanism. Here, *I* and *I*_0_ represent the fluorescence intensities of Ag_2_S QDs at 480 nm in the presence and absence of target metal ions, respectively.^327^

Tedsana *et al.* developed the first circular dichroism (CD)-based QD sensor for Ni^2+^ and Co^2+^ detection using l-cysteine-capped CdS QDs, where chiral surface ligands transduce metal binding into optical activity changes rather than fluorescence intensity changes.^328^ Ni^2+^ and Co^2+^ coordination with cysteine's amine and carboxylate groups produces distinct metal-specific chiroptical signatures: a sign inversion at 324 nm for Ni^2+^ and enhancement at 352 nm for Co^2+^, enabling differentiation between the two analytes. Selectivity against Pb^2+^, Hg^2+^, Zn^2+^, and Cu^2+^, which produce no comparable CD response, arises from distinct metal-specific coordination geometries. The sensor achieved LODs of 7.33 µM (Ni^2+^, 10 to 60 µM range) and 1.13 µM (Co^2+^, 4 to 80 µM range). This chiroptical approach avoids the non-specific quenching that limits conventional fluorescence-based differentiation. Recovery in tap, drinking, and pond water ranged 90 to 104%, in strong agreement with ICP-OES. The method requires near-neutral pH and short incubation and cannot simultaneously quantify both metals in mixed samples due to overlapping chiroptical responses.

#### 5.6.1. Critical analysis

The QD-based sensors developed for antimony, manganese, cobalt, zinc, and nickel detection, while demonstrating innovative sensing strategies and occasionally impressive analytical figures of merit, suffer from fundamental limitations that are amplified rather than mitigated by the metals' secondary status in environmental monitoring priorities. As documented in Table 19, these sensors span nearly 15 years of development, yet exhibit minimal evolution in validation rigor, operational robustness, or translational readiness compared to early proof-of-concept demonstrations. The field's fragmented nature, with individual research groups developing isolated platforms for specific metals without systematic comparison, standardization, or advancement of prior work, has resulted in a collection of disparate technologies that collectively fail to address the practical analytical needs driving metal monitoring in industrial, environmental, or clinical contexts. More critically, the absence of compelling use-case justification for many of these sensors raises fundamental questions about research prioritization: why develop QD-based sensors for metals where existing analytical techniques are adequate, inexpensive, and widely deployed, while neglecting critical validation that might enable translation of more promising platforms?

**Table 19:** Comparative analysis of QD-based heavy metal sensors

Material	Synthesis method	Functionalization	Detection method	Target ion	Response type	Linear range	LOD	Sample matrix	Real sample application	Additional features	Limitations
TGA-capped CdTe QDs^321^	Hydrothermal	TGA capping; BSA conjugation	Fluorescence	Sb^3+^	“Switch-off” quenching (charge transfer disruption)	0.10–22.0 mg L^−1^	2.94 × 10^−8^ g L^−1^	High selectivity over various cations (*e.g.*, Fe^3+^, Pb^2+^, As^3+^)	pH 6.5 (PBS); 5 min reaction time	Artificial pond, spring water, sewage (97.6–104.3%)	First QD Sb sensor; photostable; rapid response
CDs (C-dots)^323^	Hydrothermal (300 °C)	Inherent thiol, carboxyl, and amino groups	Photoluminescence (PL)	Co^2+^	Dynamic PL quenching (formation of Co_*x*_S_*y*_ NPs)	10 nM to 100 µM	∼5 nM	Specific toward Co^2+^ against 11 common metal ions	pH 9.0; 1 h incubation	Vitamin B_12_, stream and lake water (recoveries not explicitly quantified in table format)	Label-free; simple; superior sensitivity
l-Cyst-CdS QDs^328^	Not reported	l-Cysteine capping	Circular dichroism (CD)	Ni^2+^, Co^2+^	Chiroptical response (chiral complex formation)	Ni^2+^: 10–60 µM; Co^2+^: 4–80 µM	Ni^2+^: 7.33 µM; Co^2+^: 1.13 µM	Highly selective over 17 competing species	pH 7.0 (PBS); 10 min incubation	Tap, drinking, and pond water (90–104%)	First CD metal sensor; discriminates ions by wavelength
l-Cys-MnZnS QDs^325^	Aqueous route	Mn-doped; l-cysteine capping	Phosphorescence	MnO_4_^−^	“Switch-off” quenching (l-cys oxidation and surface defects)	0.5–100 µM	0.24 µM	Selective over 15 anions (*e.g.*, Cl^−^, SO_4_^2−^)	pH 8.0 (Tris–HCl); 10 min reaction time	Tap and pond water (95.3–106.2%)	Background-free (no autofluorescence); low toxicity
β-CD@ZnO QDs^326^	Sol–gel	β-Cyclodextrin capping	Fluorescence	Co^2+^	Aggregation-induced quenching (AIQ)	1.0–10 µM	0.34 µM	Selective over 16 ions; Cu^2+^ interferes if >1 mM	pH 7.54 (BR buffer); 3 min reaction time	Tap and lake water (98.2–113.0%)	Cadmium-free; inexpensive; non-toxic
Ag_2_S QDs^327^	Hydrothermal	Thioglycollic acid (TGA)	NIR-II fluorescence	Zn^2+^, Cd^2+^	“Turn-on” enhancement (surface passivation shell)	Up to 40 µM	Zn^2+^: 760 nM; Cd^2+^: 546 nM	High selectivity; Hg^2+^ and Cu^2+^ quench	pH 7.4 (Tris–HCl); 2 min incubation	Peptone, intracellular fluid, tap/lake water (94.8–104.2%)	NIR-II window (deep tissue penetration); low core toxicity
N,S-GQDs^326^	Hydrothermal	Nitrogen and sulfur co-doped	Fluorescence	Co^2+^	Static and aggregation-induced quenching	0–40 µM	1.25 µM	Selective over 14 metal cations (*e.g.*, Na^+^, Fe^3+^)	pH 9.0 (Tris–HCl); 3 min reaction time	Drinking and tap water (91.2–108.2%)	Biocompatible; visual paper strip sensing
Histidine-CQDs^322^	Hydrothermal pyrolysis	Histidine functionalization	Fluorescence	Mn^2+^	“Turn-on” response	3.50–35.5 µg L^−1^	1.85 µg L^−1^	Not specified in detail	Not specified in detail	Not specified	Water-soluble; highly selective

The analytical performance claims, while superficially impressive, reveal troubling inconsistencies and inadequate contextualization relative to regulatory requirements and existing monitoring capabilities. LODs span an enormous range, from 5 nM (Co^2+^) to 7.33 µM (Ni^2+^), yet no study provides meaningful justification for why the achieved sensitivity represents a significant advancement or meets a previously unmet analytical need.^328^ For antimony, Ge *et al.* report an LOD of 2.94 × 10^−8^ g L^−1^ (approximately 0.24 nM), orders of magnitude below typical environmental concentrations (1–10 µg L^−1^ in contaminated waters) and regulatory limits (6 µg L^−1^ under U.S. EPA standards), raising questions about whether such extreme sensitivity provides practical value or merely represents materials optimization pursued for its own sake.^321^ The linear detection range (0.10–22.0 mg L^−1^) begins three orders of magnitude above the regulatory limit, suggesting the sensor cannot actually quantify antimony at environmentally relevant concentrations despite its ultra-low LOD, a fundamental disconnect that undermines the platform's regulatory utility. Similarly, permanganate detection by Deng *et al.* (LOD 0.24 µM, linear range 0.5–100 µM) targets a narrow analytical niche, monitoring residual oxidant in treated water, where simple spectrophotometric methods already provide adequate sensitivity at far lower cost and complexity.^325^ The absence of comparative performance data against established techniques (ICP-MS for metals, UV-vis spectrophotometry for permanganate) prevents objective assessment of whether QD-based approaches offer genuine advantages beyond novelty.

Selectivity claims, while universally made, rest on testing protocols that systematically avoid the interference scenarios most likely to be encountered in real applications. The recurring pattern of evaluating selectivity against 10–17 individual metal ions at equimolar or modest excess concentrations fails to address the hierarchical concentration relationships characteristic of real matrices. For cobalt sensors, which typically claim selectivity over common cations including Ca^2+^, Mg^2+^, Na^+^, and K^+^, none of the reviewed studies test performance when these major matrix components are present at their typical concentrations in natural waters: Ca^2+^ and Mg^2+^ at millimolar levels (1–10 mM), Na^+^ at tens of millimolar in brackish waters, vastly exceeding target cobalt concentrations (typically nanomolar to low micromolar). Li *et al.*'s CD sensor for Co^2+^ operates through dynamic quenching attributed to formation of Co_*x*_S_*y*_ NPs at pH 9.0, yet provides no mechanistic justification for why other divalent transition metals (Ni^2+^, Cu^2+^, Zn^2+^) with similar sulfide affinities do not produce analogous quenching.^323^ The claim of “specific toward Co^2+^ against 11 common metal ions” lacks quantitative validation through competitive binding studies, Stern–Volmer constant determinations, or demonstration of constant selectivity coefficients across concentration ranges. More fundamentally, the 1-hour incubation requirement renders the sensor incompatible with time-sensitive applications, while the strict pH 9.0 requirement excludes the vast majority of environmental and industrial samples without extensive pretreatment.

The emergence of multi-analyte sensors, exemplified by Tedsana *et al.*'s chiral QD platform for simultaneous Ni^2+^ and Co^2+^ detection *via* circular dichroism, represents an important conceptual advance yet highlights severe practical limitations.^328^ While the ability to discriminate metal ions based on induced chiroptical responses (Ni^2+^ at 320 nm, Co^2+^ at 360 nm) offers elegant speciation capability, the reported limitation that the sensor “cannot simultaneously determine ions in mixtures” fundamentally negates its multi-analyte potential, the very advantage circular dichroism detection ostensibly provides. This contradiction suggests that the differential wavelength responses are insufficiently orthogonal to enable deconvolution of overlapping signals, or that competitive binding effects alter the chiroptical signatures in unpredictable ways. The lack of quantitative mixture analysis, mathematical modeling of signal overlap, or chemometric approaches for spectral deconvolution leaves the platform as a proof-of-principle demonstration rather than a practical analytical tool. Similarly, Wu *et al.*'s Ag_2_S QD sensor detects both Zn^2+^ (LOD 760 nM) and Cd^2+^ (LOD 546 nM) through the same “turn-on” surface passivation mechanism, providing no pathway to differentiate these metals in mixtures, a critical limitation given that zinc and cadmium frequently co-occur in industrial wastewaters and biological samples.^327^

The exploitation of unconventional detection modalities, phosphorescence, circular dichroism, and NIR-II fluorescence, while scientifically interesting, introduces operational complexity and instrumentation requirements that contradict claims of simplicity and field-deployability. Deng *et al.*'s phosphorescent Mn-doped ZnS QDs for permanganate detection offer the advantage of temporal separation from background fluorescence (background-free detection), yet require time-gated measurements with microsecond-to-millisecond delay times that necessitate specialized instrumentation rarely available outside research laboratories.^325^ The pH 8.0 requirement and complete signal loss below pH 7.0 restrict application to a narrow subset of water types, excluding acidic industrial effluents where permanganate is commonly used as oxidant. Wu *et al.*'s NIR-II fluorescent Ag_2_S QDs, while offering potential advantages for biological imaging due to reduced tissue scattering and autofluorescence in the 1000–1700 nm window, require expensive InGaAs cameras and customized optical setups unavailable in most analytical laboratories, infrastructure investments difficult to justify for routine metal analysis when ICP-MS provides superior sensitivity, selectivity, and multi-element capability using standardized instrumentation already deployed in regulatory labs worldwide.^327^

Mechanistic ambiguity pervades the field, with proposed sensing mechanisms ranging from simple charge transfer and surface coordination to NP formation and aggregation-induced quenching, yet experimental validation remains largely phenomenological. Ge *et al.* attribute Sb^3+^-induced quenching of BSA-conjugated CdTe QDs to “charge transfer disruption” based solely on fluorescence intensity changes, without time-resolved spectroscopy to confirm static *versus* dynamic quenching, electron paramagnetic resonance to detect charge transfer intermediates, or XPS to verify antimony oxidation state and binding mode.^321^ The invocation of BSA as a recognition element introduces biological variability (BSA purity, aggregation state, conformational heterogeneity) that is neither characterized nor controlled, while the mechanism by which Sb^3+^ coordination to BSA's nitrogen and oxygen lone pairs specifically quenches conjugated QDs, rather than merely altering protein conformation, remains speculative. Li *et al.*'s proposed Co^2+^ quenching mechanism through Co_*x*_S_*y*_NP formation requires validation through transmission electron microscopy demonstrating particle nucleation and growth kinetics, X-ray diffraction confirming cobalt sulfide phase identity, and quantitative correlation between Co_*x*_S_*y*_ formation and fluorescence quenching, none of which are provided.^323^ The 1-hour incubation time suggests slow NP nucleation kinetics, yet no kinetic studies establish reaction orders, rate constants, or whether equilibrium is achieved within this timeframe.

Aggregation-induced quenching mechanisms, invoked by Geng *et al.* for Co^2+^ detection with β-CD@ZnO QDs^324^ and by Boonta *et al.* for N,S-GQD sensors,^326^ suffer from inherent non-specificity that undermines selectivity claims. Any factor promoting NP aggregation, increased ionic strength, pH changes, temperature fluctuations, surfactant contamination, will produce analogous fluorescence quenching independent of target analyte presence. Geng *et al.* report that Cu^2+^ interferes at concentrations above 1 mM, yet provide no data on interference from other aggregation-promoting species such as multivalent cations (Fe^3+^, Al^3+^), polyelectrolytes, or natural organic matter, all ubiquitous in environmental samples.^324^ The rapid 3-minute response time, while operationally attractive, raises questions about whether true equilibrium aggregation is achieved or whether the measurement captures a kinetic snapshot whose magnitude depends on mixing conditions, temperature, and sample matrix composition. The claimed advantage of “visual paper strip sensing” by Boonta *et al.* lacks quantitative validation: no color change photographs under realistic lighting conditions, no inter-observer reproducibility studies, and no demonstration that visual detection provides sufficient accuracy for quantitative compliance monitoring rather than merely qualitative screening.^326^

Turn-on sensing mechanisms, while offering superior signal-to-noise ratios compared to quenching-based approaches, introduce unique validation challenges that are inadequately addressed. Wu *et al.*'s Zn^2+^/Cd^2+^ turn-on sensor attributes fluorescence enhancement to formation of a Zn-thiol or Cd-thiol passivation shell that suppresses non-radiative recombination pathways, a mechanism originally demonstrated for CdSe and CdTe QDs.^327^ However, the study provides no direct evidence for shell formation, no XPS confirming surface metal–sulfur bonding, no quantification of metal uptake stoichiometry, and no demonstration that fluorescence enhancement correlates with surface defect passivation rather than alternative mechanisms such as aggregation-induced emission or changes in local dielectric environment. More troubling, the inability to discriminate Zn^2+^ from Cd^2+^, both of which produce similar fluorescence enhancement through presumably identical passivation mechanisms, severely limits the sensor's utility given these metals' vastly different toxicological profiles and regulatory limits. The validation in “intracellular fluid” lacks critical details: cell type, lysis method, protein content, potential interferences from other thiophilic metals (Cu^+^, Hg^2+^, Ag^+^), or comparison with established analytical techniques for intracellular metal quantification.

Real-sample validation, while universally conducted, exhibits quality ranging from marginally acceptable to deeply problematic. Recovery percentages spanning 90–113% fall outside the 95–105% window considered acceptable for regulatory analytical methods, indicating significant matrix effects that are neither explained nor corrected through internal standardization or matrix-matched calibration.^328^ Ge *et al.*'s validation in “artificial pond, spring water, sewage” raises immediate questions: were these authentic samples or laboratory-prepared matrices?^321^ What were the background antimony concentrations prior to spiking? How were samples preserved to prevent antimony speciation changes or adsorptive losses? The recovery range of 97.6–104.3% appears acceptable, yet sewage represents one of the most complex analytical matrices, high dissolved organic carbon, suspended solids, surfactants, variable pH, redox-active species, making these results either remarkably robust or insufficiently challenged by the specific samples tested. The absence of certified reference material analysis, standard addition validation, or head-to-head comparison with ICP-MS prevents independent verification of accuracy claims.

The validation of Li *et al.*'s cobalt sensor in vitamin B_12_ samples exemplifies missed opportunities for demonstrating genuine analytical utility.^323^ Vitamin B_12_ contains cobalt as an integral component of the corrin ring, providing a well-defined sample matrix with known cobalt content that could serve as an excellent validation standard. However, the study provides no quantitative data on cobalt recovery from vitamin B_12_, no comparison with the known cobalt content, and no assessment of whether the sensor detects total cobalt (including organically bound forms) or only free Co^2+^ ions. Given that the sensor operates through sulfide-mediated quenching, it almost certainly does not detect organically complexed cobalt without prior acid digestion; a pretreatment requirement that negates claimed advantages of simplicity and direct detection. The validation in “stream and lake water” lacks essential matrix characterization: dissolved organic carbon content, hardness (Ca^2+^/Mg^2+^ concentrations), pH, temperature, and most critically, background cobalt levels.

pH dependencies, while acknowledged as limitations, receive insufficient mechanistic investigation or mitigation strategy development. Multiple sensors require strongly alkaline conditions, restricting application to basic waters and requiring pH adjustment for acidic or neutral samples, an additional sample manipulation step that increases cost, complexity, and contamination risk.^325^ Ge *et al.* explicitly note that antimony precipitates as Sb(OH)_3_ or Sb_2_O_3_ at pH > 7.2, creating a narrow operational window (pH 6.5–7.2) incompatible with the pH variability characteristic of environmental samples (typically pH 4–9).^321^ Yet the study proposes no buffering strategy, no investigation of alternative pH ranges through ligand modification, and no assessment of whether pH adjustment affects antimony speciation or stability. Deng *et al.*'s permanganate sensor exhibits complete fluorescence loss below pH 7.0 attributed to “quenching in acidic pH”, yet no mechanistic explanation is provided, does acidity protonate surface ligands, alter manganese dopant oxidation states, or induce aggregation?^325^ Without understanding pH-dependent failure modes, rational sensor redesign becomes impossible.

Incubation time requirements ranging from 2 minutes (Wu *et al.*) to 1 hour (Li *et al.*) highlight kinetic limitations that are rarely investigated or optimized.^327^ The 1-hour requirement for Li *et al.*'s cobalt sensor likely reflects slow Co_*x*_S_*y*_ NP nucleation and growth, yet no kinetic studies establish whether this timeframe represents approach to equilibrium or an arbitrary measurement endpoint.^323^ Temperature dependence of reaction kinetics, critical for field deployment across seasonal temperature variations (5–40 °C), receives no attention. Wu *et al.*'s rapid 2-minute response for Zn^2+^/Cd^2+^ detection appears operationally attractive, yet raises questions about reproducibility: is signal magnitude sensitive to mixing efficiency, sample volume, or precise incubation time?^327^ The absence of detailed procedural protocols, vortexing *versus* gentle mixing, temperature control, QD-to-sample volume ratios, prevents interlaboratory reproducibility and suggests these parameters were not systematically optimized.

Photostability and long-term operational stability, while critical for sensor deployment, receive minimal characterization. Only Ge *et al.* explicitly claim photostability for their antimony sensor, yet provide no quantitative data on photobleaching rates under continuous illumination, maximum cumulative light exposure before signal degradation, or comparison with unstabilized QDs to quantify improvement.^321^ Storage stability data are similarly scarce: Wu *et al.* provide no information on Ag_2_S QD shelf life, aggregation propensity during storage, or signal drift over time.^327^ The lack of accelerated aging studies (elevated temperature storage) prevents operational lifetime projection, will sensors function after 1 week, 1 month, or 1 year of storage under field conditions? Claims of reusability are notably absent across all platforms, suggesting these are single-use sensors whose manufacturing and disposal costs must be justified against per-test analytical value. Interference from structurally or chemically similar species reveals fundamental selectivity limitations that are obscured by careful choice of interferent panels. Tedsana *et al.*'s chiral sensor claims selectivity over 17 competing species, yet the composition of this panel is not specified, are they the 17 most likely interferents based on environmental occurrence and coordination chemistry, or a convenience sample selected to demonstrate apparent selectivity?^328^ The circular dichroism approach inherently discriminates based on chiral complex formation, yet many divalent metals (Cu^2+^, Zn^2+^, Cd^2+^, Mn^2+^) form chiral complexes with l-cysteine, raising questions about orthogonality of CD signals. Deng *et al.*'s permanganate sensor claims selectivity over 15 anions including chloride, sulfate, and phosphate, yet permanganate's strong oxidizing power means any reducing agent present in the sample, organic matter, Fe^2+^, sulfide, nitrite, will consume permanganate before it reaches the sensor surface.^325^ This chemical interference, rather than sensor selectivity, determines analytical specificity in real samples.

Synthesis reproducibility and batch-to-batch variability, while briefly mentioned, receive no systematic quantification. The temporal span of sensor development suggests minimal inter-study reproducibility assessment: no independent laboratories have validated or extended these platforms, no interlaboratory comparisons have been conducted, and no sensors have transitioned to commercial availability, strong indicators of irreproducibility or insufficient performance to warrant further investment. Hydrothermal synthesis methods are notoriously sensitive to heating rate, maximum temperature, dwell time, precursor purity, and autoclave geometry, yet no study reports statistical process control data across multiple synthesis batches.^325^ The consequence is that reported analytical performance may represent best-case results from optimally prepared batches rather than reproducible capabilities achievable by independent operators. The absence of economic analysis and commercialization pathway assessment is particularly striking for sensors targeting metals with established, low-cost analytical alternatives. AAS provides multi-element capability for Zn, Mn, Co, and Ni using widely available commercial instruments and standardized protocols. Portable X-ray fluorescence (XRF) analyzers enable field-based metal screening at similar or superior LODs without sample preparation. Colorimetric test kits for permanganate cost pennies per test and require no instrumentation beyond visual color comparison. Against these established alternatives, QD-based sensors must demonstrate not merely comparable performance but substantial advantages, lower cost, superior selectivity, reduced sample preparation, enhanced portability, or multi-analyte capability, to justify development and regulatory validation costs. Yet no study provides cost-per-test projections, manufacturing scalability assessments, or total cost of ownership calculations including instrumentation, consumables, training, and quality control.

Environmental and toxicological implications of sensor materials themselves receive insufficient scrutiny. Ge *et al.*^321^ and Tedsana *et al.*^328^ employ cadmium-based QDs for antimony and nickel/cobalt detection, creating the same regulatory contradiction identified for cadmium sensors: deploying toxic heavy metals to detect other toxic heavy metals. While encapsulation strategies and bovine serum albumin conjugation may reduce acute cadmium leaching, long-term environmental fate following sensor disposal remains uncharacterized. Wu *et al.*'s Ag_2_S QDs, while avoiding cadmium, introduce silver, itself an antimicrobial biocide with documented aquatic toxicity.^327^ The claim of “low core toxicity” lacks supporting data from standardized ecotoxicity assays (Daphnia magna, algal growth inhibition, fish embryo toxicity), leaching studies under environmental pH and redox conditions, or life-cycle assessment of manufacturing and disposal impacts.

The utilization of biological recognition elements (BSA)^321^ or biomolecule-derived precursors introduces protein stability concerns rarely addressed. BSA conjugation enhances antimony selectivity through specific coordination sites, yet protein conformation is exquisitely sensitive to temperature, pH, ionic strength, and chemical denaturants. Storage of BSA-QD conjugates requires refrigeration, protection from microbial contamination, and stability monitoring for protein aggregation or degradation, operational requirements incompatible with field deployment in resource-limited settings lacking cold chain infrastructure. The study provides no data on BSA-QD conjugate shelf life, optimal storage conditions, or whether freeze–thaw cycles degrade analytical performance. Histidine functionalization introduces similar concerns regarding amino acid oxidation, racemization, or leaching during storage and measurement.^322^

Regulatory acceptance pathways and validation standards appropriate for metal analysis receive no consideration. EPA Method 200.7 and 200.8 for metals by ICP-AES and ICP-MS specify detailed quality control requirements: method LOD determination through replicate analysis of spiked samples, linear dynamic range verification, accuracy assessment using certified reference materials, precision quantification through replicate measurements, and interference studies with element-specific interferent lists. None of the QD sensor studies approach this level of validation rigor. The absence of certified reference material analysis is particularly problematic, NIST, NRCC, and other metrology institutes provide water, sediment, and biological tissue reference materials with certified metal concentrations that enable unambiguous accuracy assessment. Reliance on spiked recovery studies in real samples cannot distinguish between accurate quantification and fortuitous error cancellation.

The lack of standardized performance metrics and reporting formats severely hampers inter-study comparison and meta-analysis. LODs are reported in inconsistent units (g L^−1^, M, ppb, mg L^−1^) without conversion to molar concentrations that enable direct comparison. Linear ranges vary in definition, some report concentration ranges over which linearity is demonstrated, others the maximum range tested regardless of linearity quality. Selectivity is assessed through different protocols (individual interferent addition, interferent cocktails, binary mixtures) at varying concentration ratios, preventing quantitative comparison of selectivity coefficients. Recovery studies use different sample types (tap water, lake water, sewage, vitamin B_12_) with uncharacterized background metal levels, making recovery percentages difficult to interpret. The field would benefit enormously from consensus reporting standards specifying minimum validation requirements, standardized interferent panels for each target metal, reference materials for accuracy assessment, and quality metrics (*R*^2^ for linearity, relative standard deviation for precision). Critical evaluation of the claimed advantages reveals a pattern of overstated novelty and inadequate competitive benchmarking. Li *et al.* claim “superior sensitivity” for cobalt detection (5 nM LOD) yet provide no comparison with GFAAS (LOD typically 0.1–1 µg L^−1^ or 1.7–17 nM for Co), electrochemical methods (anodic stripping voltammetry LOD ∼10 nM), or established fluorescence sensors.^323^ Ge *et al.* tout their platform as the “first QD Sb sensor”, a claim of dubious value absent demonstration that antimony monitoring represents an unmet analytical need or that QD-based detection offers advantages over established techniques.^321^ Tedsana *et al.*'s “first CD metal sensor” represents a genuine methodological innovation, yet the inability to perform mixture analysis negates the primary advantage circular dichroism could provide.^328^ Wu *et al.* emphasize NIR-II window detection for “deep tissue penetration”, yet the validation focuses on water samples and intracellular fluids rather than *in vivo* imaging where this advantage would be realized.^327^

The reported limitations, while acknowledged, are presented as minor constraints rather than fundamental barriers to deployment. Ge *et al.* note pH sensitivity and antimony precipitation above pH 7.2, yet dismiss this as a manageable issue despite it excluding most environmental waters (typical pH 7.0–8.5).^321^ Li *et al.* acknowledge “minor matrix effects in natural water samples” without quantifying their magnitude or demonstrating correction strategies.^325^ Boonta *et al.* note “strict pH dependence (requires basic media)” yet provide no investigation of pH-insensitive alternatives or buffering strategies.^326^ This pattern of limitation acknowledgment without mitigation attempts suggests acceptance of fundamental design flaws rather than commitment to developing deployable technologies. The temporal distribution of publications, concentrated heavily in 2015–2017 with sparse recent activity, suggests declining research interest or recognition that these platforms offer limited translational potential.^326^ The absence of follow-up studies extending or validating earlier work, lack of independent replication by other research groups, and failure of any platform to achieve commercial availability or regulatory acceptance indicate that the scientific community has implicitly concluded these sensors do not warrant further investment. This stands in stark contrast to QD sensors for priority pollutants (Cd, Pb, Hg, As, Cr), where sustained research activity, iterative improvements, and occasional commercialization attempts reflect ongoing interest and perceived value.

Advancing QD-based sensors for “other metals” beyond proof-of-concept demonstrations requires fundamental reorientation toward use-case-driven development and rigorous competitive benchmarking. First, clear articulation of unmet analytical needs, specific monitoring scenarios where existing techniques are inadequate, prohibitively expensive, or operationally impractical, must precede sensor design. Generic claims of “environmental monitoring” or “water quality assessment” are insufficient; specific applications such as real-time cobalt monitoring in battery manufacturing wastewater, portable manganese detection in well-water screening, or antimony speciation in mining-impacted groundwater should drive performance target definition. Second, head-to-head performance comparisons with established analytical techniques (ICP-MS, AAS, electrochemical sensors, colorimetric kits) using identical samples and testing protocols must become mandatory, enabling objective assessment of whether QD sensors offer genuine advantages. Third, validation using certified reference materials, matrix-matched calibration, and interlaboratory reproducibility studies should be required before publication, raising the evidence bar from proof-of-concept to analytically validated methods. Fourth, mechanistic studies employing advanced spectroscopic techniques, time-resolved fluorescence, X-ray absorption spectroscopy, *in situ* surface-enhanced Raman spectroscopy, must replace phenomenological mechanism assignments with molecularly validated understanding. Fifth, techno-economic analysis comparing sensor production costs, instrumentation requirements, and operational expenses with existing alternatives should inform development prioritization, preventing investment in platforms unlikely to achieve cost-competitiveness. Sixth, environmental impact assessment including ecotoxicity testing, leaching studies, and life-cycle analysis must address the environmental implications of deploying nanomaterials for environmental monitoring. Finally, engagement with potential end-users, industrial environmental managers, regulatory agencies, water utilities, during early development phases will ensure that performance targets, operational constraints, and validation protocols align with actual monitoring needs rather than academic publication criteria. Only through such disciplined, translational research can QD sensors for other metals transcend their status as laboratory curiosities to become practical analytical tools, or alternatively, reveal that existing technologies adequately serve these monitoring needs and research efforts should be redirected toward more promising applications.

## 6. Design and fabrication of QD-based sensors for heavy metal detection

The design and fabrication of QD-based sensors for heavy metal detection constitute a critical interface between fundamental nanomaterial properties and practical analytical deployment. While the unique photophysical characteristics of QDs underpin their sensing capability, their translation into reliable, field-deployable platforms requires the coordinated integration of surface functionalisation strategies, hybrid nanomaterial architectures, device miniaturisation, and interface engineering tailored to complex environmental matrices.^24^ The design phase involves the rational selection of QD composition, size, and surface chemistry to achieve metal-ion selectivity while preserving high QYs and photostability under operational conditions. Subsequent fabrication steps incorporate these optimised nanomaterials into transduction platforms ranging from benchtop optical systems to portable microfluidic devices and smartphone-integrated sensors, each imposing distinct constraints on sensor architecture, sample handling, and signal acquisition.^329^ This section therefore focuses on the key design principles and fabrication strategies that enable the transition of QD-based heavy metal sensors from laboratory-scale concepts to practical tools for environmental monitoring.

### 6.1. Surface modification and functionalisation strategies

Surface functionalisation represents the most critical design parameter governing the selectivity, sensitivity, and operational stability of QD-based heavy metal sensors. The native surface of as-synthesised QDs, typically passivated by hydrophobic ligands such as trioctylphosphine oxide, oleylamine, or oleic acid employed during colloidal synthesis, requires deliberate modification to achieve aqueous dispersibility and introduce analyte-specific recognition sites. Ligand exchange procedures replace native capping agents with bifunctional molecules featuring both QD-anchoring groups (thiols, carboxylates, phosphonates, amines) and metal-coordinating moieties selected according to HSAB principles to impart selectivity for target heavy metal ions. The choice of surface ligand profoundly influences multiple sensor characteristics including hydrodynamic size, colloidal stability, QY retention, accessible surface area for metal binding, and resistance to non-specific interactions with matrix components.^330^

Thiol-containing ligands including mercaptopropionic acid, mercaptoacetic acid, cysteine, GSH, and penicillamine constitute the most extensively employed functionalisation agents for cadmium-based QDs owing to their strong affinity for cadmium surface atoms and simultaneous provision of carboxylate groups for metal ion coordination.^331^ These bifunctional thiols impart aqueous dispersibility whilst creating coordination pockets with preferential affinity for soft Lewis acids including Hg^2+^, Pb^2+^, and Cu^+^ according to Pearson's HSAB theory. Mercaptopropionic acid-capped CdTe QDs have demonstrated exceptional sensitivity for mercury detection, achieving limits of detection in the low nanomolar range through fluorescence quenching mechanisms mediated by electron transfer from photoexcited QDs to bound Hg^2+^ ions. The terminal carboxylate groups additionally enable further conjugation with biomolecular recognition elements through carbodiimide-mediated coupling chemistry.^332^ Polyethyleneimine (PEI) functionalisation has emerged as an effective strategy for enhancing heavy metal detection capabilities of carbon-based QDs through introduction of abundant amine groups that serve as metal coordination sites. PEI-modified CQDs exhibit concentration-dependent fluorescence quenching in the presence of Cu^2+^ ions spanning concentrations from 0 to 38 ppm, establishing quantitative relationships suitable for environmental monitoring applications. The high density of amine groups on branched PEI molecules provides multiple metal binding sites per functionalised QD, amplifying sensor response and enabling detection at environmentally relevant concentrations. Infrared spectroscopic analysis confirms successful PEI grafting through appearance of characteristic N–H stretching and bending vibrations, whilst preservation of QD photoluminescence following functionalisation validates retention of optical properties essential for sensing applications.^333^

Functionalisation of GQDs with thiol groups (–SH) has been demonstrated through computational studies to enhance heavy metal detection capabilities through increased total dipole moment and improved electronic interaction capacity with metal ions. DFT calculations reveal that –SH functionalised GQDs exhibit altered electrostatic potential distributions upon metal adsorption, facilitating selective recognition of Pb^2+^, Cd^2+^, and Hg^2+^ through modulation of charge transfer characteristics. The sulfur atoms in thiol groups act as soft Lewis bases with high affinity for soft Lewis acid metal ions, whilst the introduction of functional groups also modifies the HOMO–LUMO gap and electronic structure of GQDs, thereby tuning their optical response to metal binding events. Experimental validation of these computational predictions through systematic synthesis and characterisation of functionalised GQDs remains an important direction for translating theoretical insights into practical sensor designs.^334^

Recent advances have demonstrated nitrogen and sulfur dual-doped CDs functionalised with specific ligands achieving sub-nanomolar LODs for multiple heavy metals in real water samples. Perovskite QDs with tailored surface ligands have shown exceptional stability and selectivity for Cr(vi) detection through IFE mechanisms, with limits of detection reaching 0.1 nM. Computational-guided functionalisation of GQDs with –SH groups enhance dipole moment changes upon metal binding, enabling selective Pb^2+^ detection. Electrochemical sensors using GQDs modified glassy carbon electrodes have demonstrated simultaneous detection of Cd(ii), Pb(ii), and Hg(ii) with LODs of 0.02–0.08 µg L^−1^.^335^

### 6.2. Hybrid nanomaterial architectures

The integration of QDs with complementary nanomaterials including metal NPs, CNTs, graphene, MOFs, and plasmonic nanostructures yield hybrid architectures that exhibit synergistically enhanced sensing performance compared to pristine QD systems. These hybrid platforms exploit distinct physicochemical properties of each constituent material to amplify signal transduction, improve selectivity through orthogonal recognition mechanisms, enhance photostability, and enable ratiometric detection schemes that provide built-in calibration. The design of effective hybrid sensors requires careful consideration of interfacial interactions, energy level alignment, charge transfer pathways, and spatial arrangement of components to achieve optimal performance whilst maintaining fabrication simplicity and cost-effectiveness suitable for practical deployment.^336,337^

Perovskite QD (PQD) composites integrated with MOFs represent an emerging class of hybrid sensors that combine the exceptional photoluminescence properties of PQDs (QYs 50–90%, narrow emission linewidths 12–40 nm) with the structural diversity and tuneable porosity of MOFs. The PQD@MOF architecture addresses critical limitations of pristine PQDs including aqueous instability and susceptibility to environmental degradation whilst simultaneously enhancing selectivity through size-selective analyte access mediated by MOF pore structures. Lead-based PQDs such as CsPbX_3_ and CH_3_NH_3_PbX_3_ exhibit superior photoluminescence QYs and tunable emission across the visible spectrum, whilst lead-free variants including Cs_3_Bi_2_X_9_ and CsSnX_3_ address toxicity concerns and offer improved stability in aqueous environments suitable for water quality monitoring. The detection mechanism in PQD-based sensors operates through fluorescence quenching or enhancement triggered by cation exchange, electron transfer, and FRET processes, driven by the high surface-to-volume ratio and defect-tolerant electronic structure of perovskite nanocrystals.^335^

Noble metal NP/CNT composites constitute another important class of hybrid nanomaterials that effectively combine the catalytic activity and plasmonic properties of metal NPs with the exceptional electrical conductivity and mechanical strength of CNTs. Gold, silver, and platinum NPs deposited on CNT scaffolds exhibit cooperative effects arising from metal–support interactions that enhance charge transfer kinetics and provide multiple transduction pathways including optical (surface-enhanced fluorescence), electrochemical (enhanced electron transfer), and plasmonic (localised surface plasmon resonance) mechanisms. These multi-modal hybrid sensors demonstrate remarkable versatility in heavy metal detection across diverse sample matrices and concentration ranges.^338^ Recent hybrid systems include Ag_2_Se QDs with plasmonic NPs achieving near-infrared detection suitable for tissue penetration. CDs combined with MOFs enable ratiometric sensing with built-in correction for matrix effects. QD-based fluorescent nanosensors integrated with carbon nanomaterials have shown food safety applications with LODs below regulatory limits.^336–338^

### 6.3. Microfluidic integration and lab-on-chip platforms

Microfluidics refers to the science and technology of precisely controlling and manipulating fluids that are geometrically constrained to sub-millimeter dimensions, typically within networks of channels only tens to hundreds of micrometers wide; lab-on-a-chip (LOC) platforms extend this concept by consolidating multiple laboratory functions, including sample injection, mixing, reaction, separation, and detection, onto a single miniaturized device.^339^ Relative to the paper-based and smartphone-integrated platforms discussed elsewhere in this section, microfluidic and lab-on-chip systems offer superior control over reaction kinetics and fluid transport, enabling more precise multiplexed analysis and integrated sample pretreatment, though typically at greater fabrication complexity and cost than simple paper-based formats.

Microfluidic integration of QD sensors represents a transformative approach toward miniaturised, automated, and high-throughput heavy metal analysis with substantially reduced sample and reagent consumption compared to conventional benchtop methodologies. Microfluidic platforms leverage precisely controlled fluid manipulation at the microscale to achieve rapid mixing, shortened diffusion distances, enhanced mass transport, and efficient separation of interfering matrix components, collectively enabling faster analysis times and improved LODs. The integration of QD sensing elements within microfluidic channels facilitates continuous monitoring applications, multiplexed detection of multiple analytes in parallel, and incorporation of sample pretreatment modules including filtration, preconcentration, and chemical derivatisation within a single monolithic device.^329^

Recent advances in microfluidic heavy metal sensing platforms have demonstrated integration of multiple functional modules including sample injection, on-chip mixing, QD-based optical detection, and real-time data acquisition coupled with wireless transmission capabilities. These lab-on-chip systems enable rapid screening of water samples for compliance monitoring, achieving analysis times of 5–30 minutes compared to hours or days required for conventional laboratory methods. The reduced footprint and power requirements of microfluidic QD sensors position them as ideal candidates for distributed sensor networks and IoTs environmental monitoring applications where continuous, autonomous operation is essential.^329,340^ 2024 studies report integrated microfluidic platforms with QD sensors achieving 5-minute multiplexed detection of Pb, Cd, and Hg in drinking water. Innovative microfluidic technologies combine QD fluorescence with electrochemical detection for simultaneous multi-analyte quantification.^329^

### 6.4. Paper-based and smartphone-integrated sensors

Paper-based analytical devices incorporating QD sensing elements represent a disruptive technology for point-of-need heavy metal detection, combining the intrinsic advantages of cellulosic substrates (low cost, disposability, capillary-driven fluid transport, minimal power requirements) with the exceptional optical properties of QDs to create user-friendly, equipment-free sensing platforms. Nitrocellulose membranes have emerged as particularly suitable substrates due to their high surface-to-volume ratio, mechanical strength, ease of handling, and superior QD retention within the three-dimensional fibrous matrix. The porous structure of paper substrates enables passive fluid transport through capillary action, eliminating requirements for external pumps whilst simultaneously providing immobilisation sites for QD sensing probes that maintain optical activity and accessibility to target analytes.^340,341^ GQDs embedded in nitrocellulose paper strips have demonstrated quantitative detection of heavy metals through fluorescence quenching mechanisms readable with smartphone cameras. The paper-based device comprises luminescent GQDs synthesised *via* pyrolysis of citric acid and confined within the nitrocellulose matrix, where resonance energy transfer phenomena mediate the sensing response. Upon exposure to heavy metal-containing samples, dramatic fluorescence quenching occurs proportional to analyte concentration, with the resultant optical signal captured by a mobile phone camera housed in a 3D-printed portable dark chamber equipped with UV-LED excitation. This approach eliminates requirements for sophisticated instrumentation, instead leveraging the ubiquity of smartphones to democratise access to quantitative analytical capabilities in resource-limited settings.^340,341^

Smartphone-based sensing systems integrating carbon nanodot microarrays have achieved simultaneous quantitation of multiple heavy metals including Hg^2+^, Pb^2+^, and Cu^2+^ in environmental water samples with minimal user intervention. The system architecture comprises paper-based microarrays functionalised with fluorescent carbon nanodots for analyte recognition, coupled with a standalone smartphone-based portable reader installed with custom-designed application software that automatically acquires fluorescence changes, processes image data, quantifies analyte concentrations, generates analytical reports, and enables wireless data sharing. End-user operation is limited to pipetting samples onto the microarray chip, inserting the chip into the portable reader, and opening the application to acquire results within 5 minutes, with no requirements for sample pretreatment or expensive laboratory equipment beyond a smartphone. This versatile and cost-effective approach demonstrates promise for decentralised environmental surveillance and citizen science applications in resource-constrained environments.^340^

The integration of molecularly imprinted fluorescence sensors with smartphone detection platforms represents an advanced iteration combining the molecular selectivity of imprinting technology with the operational simplicity of mobile device-based readout. These systems employ ion-imprinted polymers synthesised around target metal ions to create complementary recognition cavities that exhibit selectivity comparable to biological receptors whilst offering superior chemical stability and tolerance to harsh environmental conditions. Smartphone-integrated detection enables real-time on-site monitoring without requirements for laboratory infrastructure, positioning these platforms as ideal candidates for field deployment in drinking water quality assessment, industrial effluent monitoring, and agricultural runoff surveillance.^340^

### 6.5. Performance metrics and validation strategies

The analytical performance of QD-based heavy metal sensors must be rigorously characterised through standardised metrics that enable meaningful comparisons across diverse architectures and enable validation against regulatory requirements. Key performance parameters include LOD, limit of quantification (LOQ), linear dynamic range, selectivity coefficients, response time, photostability, shelf-life stability, and matrix tolerance. The International Union of Pure and Applied Chemistry (IUPAC) defines LOD as the lowest analyte concentration producing a signal distinguishable from background noise at 99% confidence (3*σ* criterion), whilst LOQ represents the lowest quantifiable concentration with acceptable precision and accuracy (10*σ* criterion).^338,342^

#### 6.5.1. Selectivity assessment protocols

Selectivity represents the sensor's ability to discriminate target analytes from potential interferents present at comparable or higher concentrations in complex matrices. Quantitative assessment employs interference studies exposing sensors to 10–1000-fold excess concentrations of common coexisting ions (Na^+^, K^+^, Ca^2+^, Mg^2+^, Fe^3+^, Zn^2+^, Cl^−^, SO_4_^2−^, NO_3_^−^) under optimised conditions. Selectivity coefficients (*K*_sel_) are calculated from competitive binding experiments or mixed-sample analysis, with values >100 indicating excellent discrimination. Advanced strategies including molecular imprinting, aptamer-based recognition, and multi-channel array formats enhance selectivity in highly complex matrices.^340,342^

#### 6.5.2. Matrix effect mitigation and validation

Real-world sample analysis necessitates comprehensive evaluation of matrix effects through recovery studies spiking authentic environmental samples (tap water, river water, industrial effluent, seawater) at environmentally relevant concentrations (1–100 µg L^−1^). Recovery percentages between 90-110% with relative standard deviations <10% validate sensor reliability across diverse matrices. Standard addition protocols compensate for matrix suppression or enhancement effects, whilst sample pretreatment strategies including solid-phase extraction, membrane filtration, and chelation-assisted preconcentration enhance sensor performance in challenging samples.^329^

#### 6.5.3. Stability and reusability assessment

Photostability under continuous excitation (1000–5000 cycles), shelf-life stability (storage at 4 °C, 25 °C for 1–6 months), and operational reusability (>50 cycles with <5% signal degradation) constitute essential metrics for practical deployment. Encapsulation within silica shells, MOF matrices, or polymer hydrogels substantially enhances long-term stability whilst preserving accessibility to target analytes. Regeneration protocols employing chelating agents (EDTA, thiourea) or thermal treatment enable sensor reusability essential for cost-effective field applications.^195^

#### 6.5.4. Standardisation and interlaboratory validation

Interlaboratory validation against certified reference materials (NIST SRM 1640a trace elements in water) and comparison with gold-standard techniques (ICP-MS, AAS) establishes analytical credibility. Participation in proficiency testing schemes and adherence to ISO 17025 accreditation requirements ensures method reliability for regulatory compliance monitoring. The development of standardised protocols for QD sensor characterisation facilitates meaningful performance comparisons and accelerates commercial translation.^338^

#### 6.5.5. Computational design and high-throughput optimisation

Machine learning-guided functionalisation and DFT simulations accelerate sensor design by predicting ligand–metal interactions, electronic structure modifications, and quenching efficiencies prior to experimental synthesis. High-throughput screening platforms enable rapid evaluation of QD size, composition, and surface chemistry combinations, substantially reducing development timelines from months to weeks. By integrating active-learning algorithms with automated experimental workflows, candidate QD-ligand architectures can be iteratively refined based on feedback from optical, electrochemical, and stability readouts, improving both sensitivity and selectivity. Coupling these pipelines with multi-objective optimisation further allows simultaneous tuning of key performance metrics such as limit of detection, photostability, and biocompatibility, enabling rational, data-driven discovery of next-generation QD-based sensors.^334^

## 7. Challenges and future perspectives

QD-based sensors for heavy metal detection have demonstrated extraordinary laboratory performance, achieving sub-nanomolar LODs, rapid response times under five minutes, and impressive selectivity ratios exceeding 100 : 1 against common interferents. However, translating these remarkable capabilities into robust, field-deployable commercial products confronts multifaceted technical, manufacturing, regulatory, and economic challenges that must be systematically addressed to realise the full potential of this technology. The path from laboratory proof-of-concept to widespread commercial adoption requires overcoming fundamental barriers in materials toxicity, synthesis reproducibility, matrix complexity management, fabrication scalability, regulatory validation, and cost reduction, while simultaneously exploring transformative opportunities enabled by artificial intelligence, sustainable manufacturing, and advanced quantum technologies. As illustrated in Fig. 32, the successful translation of QD-based heavy metal sensors from laboratory demonstrations to real-world deployment is constrained by interconnected challenges spanning materials toxicity, synthesis reproducibility, matrix interference, scalable fabrication, regulatory validation, and cost reduction.

**Fig. 32:**
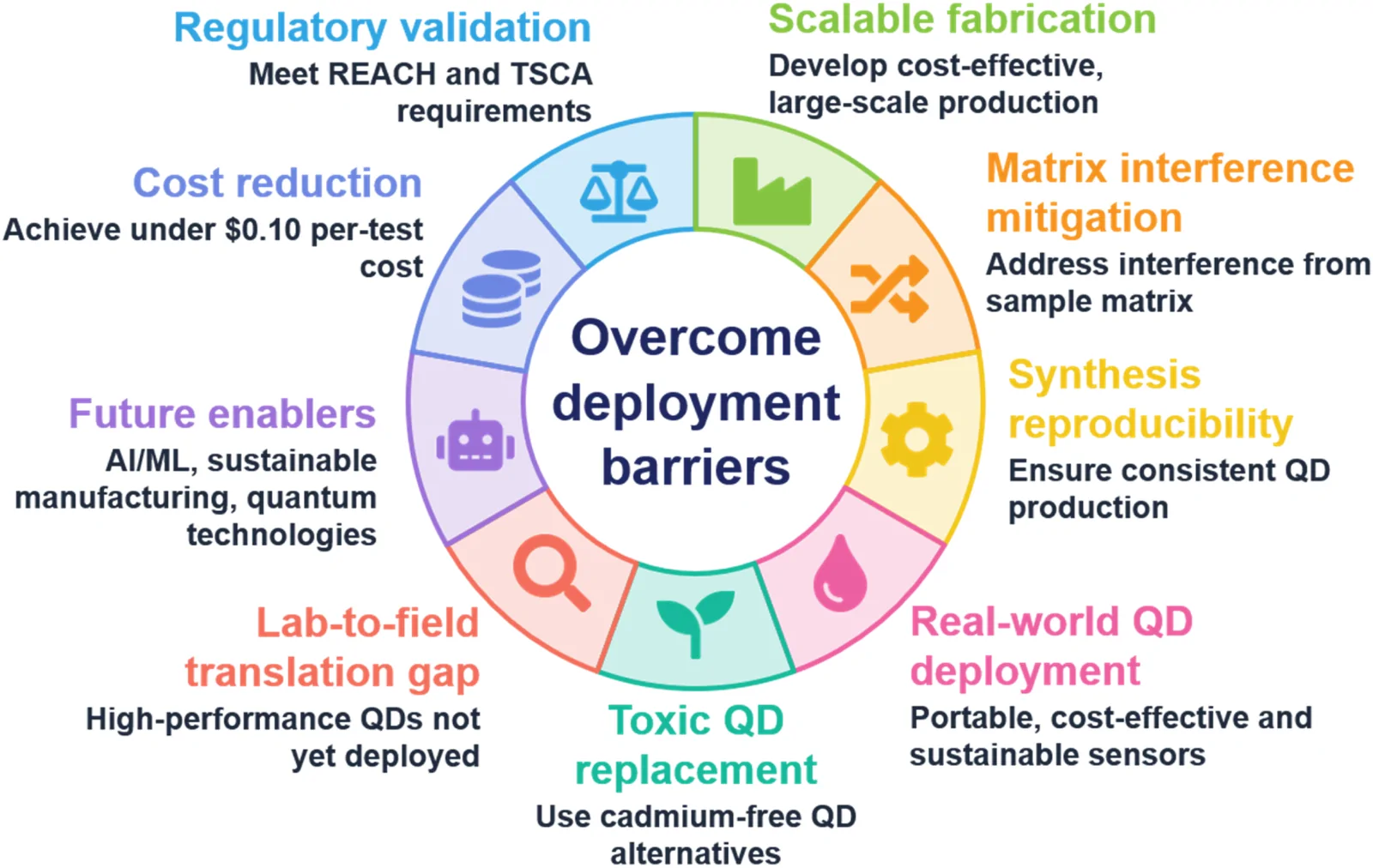
Deployment barriers and future enablers for translating QD-based heavy metal sensors from high-performance laboratory prototypes to scalable, low-cost, and regulatory-compliant field devices.

The most pressing materials challenge centres on the inherent toxicity of cadmium- and lead-based QDs, which paradoxically deliver unmatched optical excellence while raising serious environmental and regulatory concerns. These conventional QDs routinely achieve QYs exceeding 80%, exhibit emission linewidths as narrow as 20 nm, and demonstrate exceptional photostability enabling more than 10 000 excitation cycles without degradation. However, their heavy metal compositions trigger stringent regulatory restrictions under frameworks, including REACH in Europe and TSCA in the United States, while posing genuine environmental risks during kilogram-scale synthesis, involving toxic organometallic precursors and hazardous solvents. The regulatory landscape has become increasingly restrictive, with European directives specifically limiting cadmium use in consumer products and environmental monitoring devices, creating substantial barriers to commercialisation despite superior performance characteristics.^335^

Cadmium-free alternatives represent compelling solutions to these toxicity concerns, yet each candidate material suffers from critical performance deficits that currently prevent direct replacement of conventional QDs.^343^ CDs typically exhibit QYs of only 20–40% with broad, excitation-dependent emission profiles characterised by full-width-half-maximum values around 80 nm, substantially compromising both sensitivity and spectral resolution compared to cadmium-based systems.^344^ Indium phosphide QDs with zinc sulfide shells require complex multi-step synthesis procedures to achieve adequate stability and reasonable QYs, increasing manufacturing complexity and cost. Lead-free perovskites such as Cs_3_Bi_2_I_9_ and CsSnI_3_ demonstrate poor aqueous durability with degradation occurring within 24 hours of water exposure, fundamentally limiting their utility for environmental sensing applications. GQDs, while offering excellent biocompatibility and chemical stability, exhibit lower QYs and less precise emission tunability than their semiconductor counterparts.^345,346^

Recent breakthroughs have begun addressing these performance gaps through innovative materials design and surface engineering strategies. Biomass-derived CDs synthesised from agricultural waste including orange peels, rice husks, and sewage sludge have achieved QYs approaching 65% through nitrogen and phosphorus co-doping, substantially narrowing the performance gap with conventional QDs while offering the additional benefit of sustainable feedstock utilisation. Similarly, halide perovskite QDs functionalised with zwitterionic polymer coatings have demonstrated extended aqueous stability exceeding 30 days, representing a 30-fold improvement over unprotected materials. Despite these advances, comprehensive life-cycle assessments quantifying potential leaching, bioaccumulation in aquatic ecosystems, and long-term ecotoxicity remain essential prerequisites for regulatory approval and widespread deployment. The materials science community must prioritise systematic toxicological studies that establish safe handling protocols, disposal procedures, and environmental fate profiles for all QD materials intended for environmental monitoring applications.^347,348^ Synthesis reproducibility constitutes a fundamental barrier to commercialisation, as conventional colloidal synthesis methods produce exquisitely monodisperse QDs with size variations below 5% only at gram scales suitable for research applications. Scaling to production volumes exceeding one kilogram per batch inevitably introduces increased polydispersity beyond 15%, QY degradation of 20–50%, and surface chemistry inconsistencies that compromise sensor-to-sensor variability and undermine the reliability required for commercial products. These scale-dependent performance losses arise from fundamental limitations in heat and mass transfer within batch reactors, where temperature gradients, concentration inhomogeneities, and temporal variations in precursor injection produce broader size distributions and defect populations that reduce optical quality.^349,350^

Continuous flow microreactor technologies operating at precisely controlled residence times ranging from 10 to 300 seconds and temperatures between 200 and 320 °C promise industrial scalability with automated feedback control systems capable of maintaining size dispersity below 3%.^351,352^ These microfluidic synthesis platforms offer superior heat transfer coefficients, uniform mixing characteristics, and reproducible residence time distributions compared to conventional batch reactors, enabling consistent product quality across extended production runs. However, successful adaptation of established synthetic protocols to continuous flow systems demands fundamental reformulation of air-sensitive organometallic precursors into stable, pumpable inks compatible with long-term storage and automated dispensing systems. This technical challenge currently limits throughput to approximately 100 grams per hour, insufficient for meeting projected demand in large-scale environmental monitoring applications requiring kilogram quantities of high-quality QDs. Achieving true manufacturing readiness requires development and universal adoption of standardised quality control protocols that ensure batch-to-batch consistency and enable meaningful comparison across different production facilities and synthesis methods. These protocols must incorporate ensemble characterisation techniques including dynamic light scattering for hydrodynamic size determination, spectrofluorimetry for QY and emission profile assessment, and inductively coupled plasma optical emission spectroscopy for quantification of residual metal contaminants. Complementary single-particle analysis methods including cryogenic transmission electron microscopy and single-molecule spectroscopy provide critical insights into size distribution tails and defect populations that disproportionately impact sensor performance. Establishing traceable reference materials, inter-laboratory validation studies, and certification procedures analogous to those used in pharmaceutical manufacturing will prove essential for building confidence in QD-based sensors among regulatory agencies and end users.

Real-world matrix complexity represents perhaps the most formidable analytical challenge confronting QD-based heavy metal sensors, as environmental samples routinely contain interfering species at concentrations 1000 to 1 000 000-fold higher than target analytes. These interferents include ubiquitous metal ions such as Fe^3+^ and Mn^2+^, natural organic matter comprising humic and fulvic acids, inorganic anions including phosphates and sulfates, and anthropogenic contaminants such as surfactants and dissolved organic compounds. These matrix components trigger non-specific fluorescence quenching through static and dynamic mechanisms, cause IFEs that reduce excitation and emission intensities, or induce aggregation that fundamentally alters QD optical properties. The cumulative impact of these interference mechanisms can produce signal suppression of 30–70% or generate false positive responses, severely compromising sensor accuracy and reliability in field applications. Advanced mitigation strategies have been developed to address these matrix complexity challenges through multiple complementary approaches. Molecularly imprinted polymers create highly selective steric-exclusion cavities with three-dimensional structures complementary to target metal ions, providing size and shape selectivity that discriminates against interferents while maintaining high affinity for analytes of interest. These synthetic receptors can be integrated directly onto QD surfaces or incorporated into hybrid sensor architectures to provide pre-concentration and sample cleanup capabilities. Dual-transduction platforms that combine fluorescence-based detection with electrochemical techniques such as anodic stripping voltammetry offer orthogonal validation mechanisms, where concordance between independent measurement principles provides confidence in result accuracy even in complex matrices. The electrochemical component provides quantitative determination with minimal matrix interference, while the optical component enables rapid screening and spatial mapping applications.^353,354^

Chemometric algorithms, including partial least squares discriminant analysis and support vector machines, enable deconvolution of overlapping spectral signatures from multi-channel QD arrays, extracting target analyte signals from complex backgrounds through multivariate calibration approaches. These computational methods exploit the full information content of emission spectra rather than relying on single-wavelength measurements, substantially improving selectivity and LODs in challenging matrices. Despite these advances, spectral multiplexing strategies that fully exploit the visible-to-near-infrared emission tunability of mixed QD libraries remain substantially underexplored. Demonstrations of simultaneous five-plex detection for Pb^2+^, Cd^2+^, Hg^2+^, Cu^2+^, and Cr^6+^ using size-gradient CdSe/ZnS populations with distinct emission wavelengths highlight the potential for comprehensive heavy metal profiling using single sensor platforms, yet systematic development of optimised QD combinations and data analysis protocols has not been pursued.^335,355^

Fabrication scalability confronts fundamental materials incompatibilities and processing constraints that prevent direct translation of laboratory deposition methods to industrial manufacturing platforms. Laboratory spin-coating procedures yield uniform QD films with thicknesses of 50–200 nm and QD retention efficiencies exceeding 95%, but these batch processes prove incompatible with high-volume production requirements. Industrial roll-to-roll printing technologies operating at line speeds of 100 metres per minute demand low-viscosity inks with viscosities of 1–20 centipoise, yet such formulations prove prone to dewetting on common substrates, coffee-ring pattern formation during drying, and ligand desorption under the elevated temperatures and mechanical stresses encountered during processing. These materials challenges result in non-uniform QD distribution, reduced fluorescence QYs, and compromised sensor performance compared to laboratory prototypes.^356,357^ Flexible substrate integration presents additional technical hurdles requiring careful materials selection and surface functionalisation strategies. Plasma-treated cellulose and polyethylene terephthalate substrates can achieve peel strengths exceeding 90 N cm^−1^ when properly functionalised with adhesion promoters and coupling agents, providing adequate mechanical robustness for handling and deployment. However, the hygroscopic nature of these polymer substrates and their relatively high oxygen permeability necessitate incorporation of multilayer barrier coatings with water vapour transmission rates below 0.001 g per m^2^ per day to prevent irreversible QD oxidation during storage and field deployment. These protective layers must be engineered to maintain sensor response kinetics under one second while providing hermetic encapsulation against environmental extremes, a materials engineering challenge that currently limits practical shelf-life to 3–6 months compared to the two-year minimum target for commercial viability.^358^ Operational stability under field deployment conditions spanning temperature ranges of 0–50 °C, relative humidity variations from 20–95%, and continuous solar irradiation intensities reaching 10 000 lux requires comprehensive environmental testing and accelerated aging protocols. Current sensor designs exhibit gradual performance degradation under these stress conditions, with particularly rapid deterioration observed under combined high temperature and humidity exposure. Development of robust encapsulation strategies, photostabilising additives, and thermally stable surface ligands remains essential for achieving the multi-year operational lifetimes required for permanent monitoring installations and the stringent performance specifications demanded by regulatory compliance testing.^359^

Regulatory validation gaps critically undermine the credibility and commercial viability of QD-based sensors, as the absence of standardised testing protocols and certified reference materials prevents meaningful comparison with established analytical techniques. Without IUPAC-compliant limit-of-detection and limit-of-quantification determination protocols that account for matrix effects and interference contributions, claimed sensitivity specifications cannot be independently verified or meaningfully compared across different sensor platforms and manufacturers. The development of NIST-traceable reference materials spiked at concentrations spanning regulatory thresholds from 1 to 100 µg L^−1^ in representative matrix compositions proves essential for establishing measurement traceability and enabling proficiency testing programs.^360^ Multi-site validation studies comparing QD sensor performance against reference methods including ICP-MS and AAS with agreement criteria of ±5% provide the evidentiary foundation required for regulatory acceptance. Such validation campaigns must encompass diverse sample types of representative of intended applications, including drinking water, surface water, groundwater, industrial effluents, and soil extracts, with systematic evaluation of accuracy, precision, selectivity, and robustness under field conditions. Only through successful completion of these comprehensive validation studies can QD-based sensors secure certification under frameworks such as the US EPA Environmental Technology Verification Program or EU Water Framework Directive monitoring requirements. Standardisation efforts must address not only analytical performance characteristics but also manufacturing quality control, device calibration procedures, data reporting formats, and inter-laboratory comparison protocols. Professional societies and standards organisations including ASTM International, ISO, and IUPAC should collaborate with sensor developers to establish consensus testing methods, performance specifications, and certification criteria that provide confidence to regulatory agencies and end users. The creation of a robust standardisation framework will prove essential for transitioning QD-based sensors from research curiosities to widely accepted analytical tools suitable for regulatory compliance monitoring and environmental decision-making.^360^

Cost structures reveal critical economic viability thresholds that must be achieved to enable widespread adoption of QD-based sensors for routine environmental monitoring applications. Current prototype devices remain economically uncompetitive with centralized laboratory analysis for most applications, and substantial cost reductions would be needed to reach price points suitable for high-volume routine water quality testing; this remains a goal for the field rather than an established benchmark. Achieving such reductions would require simultaneous advances across multiple dimensions including QD synthesis, device fabrication, and instrumentation development. QD production costs would need to decrease substantially from current research-grade levels toward commodity pricing, through development of scalable synthesis routes using earth-abundant precursors and elimination of costly purification steps. Single-step printing processes that directly pattern QD sensing elements onto low-cost substrates without intermediate transfer or assembly operations could substantially reduce manufacturing complexity and labor costs. Smartphone-based fluorescence readers, whose cost is amortized across many individual tests, offer an attractive alternative to dedicated instrumentation, leveraging the ubiquitous availability of high-quality cameras, processors, and displays in modern mobile devices while providing connectivity for data transmission and cloud-based analysis. The economic analysis must also consider lifecycle costs including calibration requirements, consumable replacement, maintenance procedures, and disposal expenses that substantially impact total cost of ownership beyond initial device purchase price.^361,362^ Business models based on sensor-as-a-service offerings with predictable subscription pricing may prove more attractive to end users than capital equipment purchases requiring significant upfront investment. Government procurement policies, environmental compliance mandates, and public health initiatives will likely play critical roles in creating initial markets that enable manufacturing scale-up and cost reduction through learning curve effects and economies of scale.

Despite the substantial challenges outlined above, emerging technological advances could reshape the landscape of QD-based heavy metal detection over time. Artificial intelligence integration represents one promising avenue for performance enhancement: convolutional neural networks trained on hyperspectral datasets have shown potential for improved discrimination of target ions from common interferents across complex concentration mixtures, though the accuracy achievable in realistic field deployments has yet to be established. Beyond multi-ion discrimination, machine learning approaches are also being explored for spectral deconvolution, separating overlapping fluorescence or absorbance signals from co-occurring metal ions or matrix chromophores that would otherwise confound single-wavelength analysis, and for matrix effect correction, learning to compensate for the ionic strength, pH, and organic matter variability that degrades sensor accuracy in real environmental samples relative to laboratory buffer systems. Chemometric approaches, including partial least squares regression and principal component analysis, offer complementary, more interpretable alternatives to deep learning for multivariate calibration, particularly valuable when training data are limited, a common constraint in heavy-metal QD sensing where individual studies rarely report more than a few hundred calibration points.

These capabilities are of particular relevance to smartphone-based quantification platforms, where on-device or cloud-based machine learning models, employing image recognition techniques to process RGB or grayscale image data, can substitute for dedicated spectrometers, potentially compensating computationally for the reduced spectral resolution and greater susceptibility to ambient lighting variation inherent to camera-based readout. These deep learning models could in principle be refined through federated learning architectures that adapt to local conditions across distributed sensor deployments without centralising sensitive data, addressing privacy concerns while enabling collaborative model improvement. Cloud-based data infrastructure, in combination with the federated learning architectures noted above, could enable aggregation of sensor readings across geographically distributed deployments into centralized environmental monitoring dashboards, supporting real-time contamination mapping and regulatory reporting at a scale not achievable from individually operated, standalone devices.^363^ Machine learning algorithms could also help optimise sensor calibration procedures, predict maintenance requirements based on performance drift patterns, and provide decision support for regulatory compliance and remediation prioritisation. For a broader treatment of AI-assisted and quantum-enhanced sensing architectures, including the role of defect engineering in tuning sensor response.^364^ The deep learning models could in principle be refined through federated learning architectures that adapt to local conditions across distributed sensor deployments without centralizing sensitive data, addressing privacy concerns while enabling collaborative model improvement. Machine learning algorithms could also help optimize sensor calibration procedures, predict maintenance requirements based on performance drift patterns, and provide decision support for regulatory compliance and remediation prioritization.

Self-powered sensing architectures, which eliminate the battery replacement and power infrastructure requirements that constrain continuous field deployment, represent a further avenue for advancing QD-based heavy-metal sensors toward practical environmental monitoring. Energy-harvesting approaches, including piezoelectric and triboelectric generators that convert ambient mechanical energy (water flow, vibration) into electrical power, and photovoltaic elements that harvest ambient or solar illumination, could in principle power the excitation source, signal processing electronics, and wireless transmission components of a QD-based sensor node without requiring battery replacement or grid connection. This capability would be particularly valuable for the remote, distributed sensor deployments discussed elsewhere in this section, where physical access for maintenance is limited or costly. To date, however, self-powered operation has not been demonstrated for QD-based heavy-metal sensors specifically and remains a substantially earlier-stage concept than the other technological directions discussed in this section.

Sustainable manufacturing approaches that leverage agricultural waste streams as renewable feedstocks for carbon dot production offer a route toward substantially lower material costs than conventional CdSe synthesis, though the scale at which this could be achieved commercially remains to be demonstrated. This circular economy approach could simultaneously address the toxicity concerns associated with heavy metal QDs while converting problematic waste streams into valuable sensing materials. Microbial biosynthesis using genetically engineered *Escherichia coli* strains offers a further, more speculative route to QD production, generating chiral QDs with inherent biomolecular recognition capabilities that could eliminate the need for post-synthetic functionalization. These biological manufacturing routes operate at ambient temperature and pressure using aqueous processing conditions, which would reduce energy consumption and solvent waste compared to conventional high-temperature organometallic syntheses if scaled successfully.^365,366^

Internet-of-Things architectures comprising distributed sensor networks could in principle deliver watershed-scale pollution mapping with improved spatial and temporal resolution, though the network scale and resolution ultimately achievable will depend on deployment cost and infrastructure investment. Blockchain-secured data transmission has been proposed as a means of ensuring evidentiary chain-of-custody suitable for regulatory enforcement and legal proceedings, addressing concerns about data tampering and authentication that have previously limited acceptance of distributed sensor networks. Textile-based wearable sensors integrated into clothing or protective equipment could enable personal exposure monitoring by analyzing perspiration for heavy metal content, offering a route toward individual dosimetry data for occupational health and environmental justice applications, though sensitivity under real-world wear conditions remains to be established. Prototype wearable QD sensing platforms reported to date have primarily targeted other analyte classes; for example, a cadmium-free Cu–In–Zn–S quantum dot-grafted cotton fabric sensor has been demonstrated for luminescent anion detection, rather than heavy-metal cations.^367^ Adapting such wearable QD platforms to heavy-metal cation detection introduces additional challenges relative to solution-phase sensing, including maintaining QD photostability and ligand functionality under repeated mechanical flexing, and achieving reliable analyte transport from skin or environmental contact into the sensing layer. As such, wearable heavy-metal QD sensors remain at an earlier stage of development than the smartphone-integrated, paper-based, and microfluidic platforms discussed elsewhere in this review, most of which have progressed to real-sample validation.

Beyond environmental monitoring dashboards, integration of QD-based sensor data with digital health infrastructure represents a further emerging direction, particularly for wearable and point-of-care formats discussed above. Linking personal heavy-metal exposure data to electronic health records or telemedicine platforms could support clinical decision-making in occupational or environmental health contexts, though this would require QD sensor data to meet the accuracy, interoperability, and data privacy standards expected of clinical digital health systems, none of which have yet been established for QD-based heavy-metal sensors specifically.

Satellite-ground sensor fusion, combining high-resolution remote sensing imagery with distributed *in situ* measurements, has been proposed as a means of predicting contamination plume evolution and enabling proactive response to pollution events, though prediction accuracy will depend strongly on local hydrogeological conditions and sensor network density. Quantum technologies herald potentially significant sensitivity gains that may eventually enable detection approaching the thermodynamic limits of measurement. Nitrogen-vacancy diamond sensors, which exploit nuclear spin hyperfine interactions, have demonstrated exceptional sensitivity to metal ions in laboratory settings, representing a sensitivity regime substantially beyond current fluorescence-based approaches. Superconducting quantum interference devices could in principle amplify QD-generated photocurrents toward single-electron resolution, improving signal-to-noise ratios in photon-limited detection scenarios. While these quantum sensing modalities currently require sophisticated cryogenic infrastructure and controlled laboratory environments, ongoing miniaturization efforts may eventually enable field-deployable quantum sensors with substantially improved LODs.^368,369^

Looking toward the coming years, the convergence of advances in materials science, manufacturing technology, artificial intelligence, and quantum sensing could, if realized, enable substantially lower per-test costs integrated into IoT frameworks for continuous monitoring of pollution hotspots at a scale not currently achievable. Such comprehensive monitoring infrastructure could, in principle, help reduce the economic and public health burden of heavy metal contamination, including for populations that currently depend on unsafe water sources; though realizing this potential at meaningful scale remains an aspiration for the field rather than a near-term certainty. Realizing this vision demands unprecedented public-private partnerships that unite nanomaterials expertise from academic research institutions, manufacturing scale and distribution capabilities from industrial partners, artificial intelligence innovation from technology companies, and regulatory pragmatism from government agencies and standards organizations. The pathway to widespread deployment requires systematic attention to technology transfer mechanisms that bridge the gap between laboratory discoveries and commercial products, including pilot-scale demonstrations in representative field environments, economic feasibility studies that account for total lifecycle costs, and stakeholder engagement processes that address concerns from end users, regulators, and affected communities. International collaboration through organisations such as the WHO, United Nations Environment Programme, and International Standards Organisation can accelerate harmonisation of testing protocols, performance specifications, and deployment strategies across diverse regulatory frameworks and environmental conditions. Investment in workforce development and training programs will prove essential for ensuring that personnel in developing regions can effectively deploy, operate, and maintain advanced sensor technologies without requiring extensive analytical chemistry backgrounds.^370,371^Table 20 summarizes these future directions as a roadmap, indicating the current status of each and the near-term and longer-term milestones required to advance QD-based heavy-metal sensing from its present state toward widespread, regulatory-grade field deployment.

**Table 20:** Roadmap of future directions for QD-based heavy-metal sensing

Future direction	Current status	Near-term outlook	Longer-term outlook
Green QD synthesis	Biomass-derived carbon dots demonstrated at lab scale; cost and reproducibility advantages over conventional CdSe synthesis established qualitatively	Standardization of biomass feedstock sourcing; scale-up *via* hydrothermal/microwave routes	Commodity-scale, cost-competitive production integrated into sensor manufacturing
Multiplex sensing	Multiple studies demonstrate simultaneous detection of 2–3 metal ions *via* ratiometric or multi-emission QD architectures	Extension to larger ion panels; standardized cross-reactivity testing protocols	Single-platform, comprehensive heavy-metal panels matching regulatory monitoring requirements
AI-assisted environmental monitoring	Machine learning applied to signal processing, calibration, and image-based smartphone readout in early-stage demonstrations	Chemometric and deep-learning models validated against larger, standardized calibration datasets	Federated, cloud-integrated monitoring networks providing real-time, regulatory-grade contamination mapping
Self-powered sensors	Not yet demonstrated for QD-based heavy-metal sensing specifically; energy-harvesting concepts established in adjacent sensing fields	Proof-of-concept integration of piezoelectric/triboelectric/photovoltaic power with QD sensor nodes	Battery-free, maintenance-minimal sensor nodes for remote, long-duration deployment
Digital environmental surveillance	Individual sensor data reported per-study; no integrated, cross-deployment digital infrastructure demonstrated for QD sensors	Cloud-based aggregation of distributed sensor data into centralized dashboards	Integration with regulatory reporting and public environmental-health infrastructure
Large-scale field deployment	Majority of studies remain at proof-of-concept or real-sample validation stage	Expanded real-world field trials under variable environmental conditions	Widespread, standardized field deployment supported by established manufacturing and regulatory pathways

Ultimately, the success of QD-based heavy metal sensors will be measured not by laboratory performance metrics but by demonstrable improvements in environmental quality, public health outcomes, and ecosystem restoration. Technology must evolve from a promising research concept to a reliable analytical tool that empowers communities to monitor their local water supplies, enables industries to optimize pollution control systems, and provides regulatory agencies with the real-time data needed for evidence-based environmental policy. Achieving this vision requires sustained commitment to addressing the challenges outlined in this section while maintaining focus on the goal: providing accessible, affordable, and accurate heavy metal detection capabilities to all who need them, regardless of geographic location or economic resources.

## 8. Conclusion

QD-based sensors represent a transformative paradigm in heavy metal detection, offering sensitivity, selectivity, and deployability that can surpass conventional laboratory methods under favorable conditions. Over the past decade, advances in surface functionalisation, hybrid architectures, and computational signal processing have propelled laboratory prototypes toward increasingly field-relevant technologies, with individual studies demonstrating sub-nanomolar detection across diverse environmental matrices, smartphone-integrated multiplexed analysis platforms, and molecularly imprinted QD hybrids with strong selectivity against common interferents. Several mechanistic design principles emerge from the body of work reviewed here and should guide future sensor development. First, sensing performance is governed by an inherent trade-off between QD size and surface trap-state density: smaller particles offer greater surface-to-volume ratio and metal-ion binding capacity but correspondingly lower quantum yield, and rational sensor design requires deliberately balancing these competing effects rather than optimizing photophysical brightness in isolation. Second, mechanism selection should be matched to deployment context rather than sensitivity alone: turn-off quenching offers the simplest instrumentation pathway but is most vulnerable to false-positive interference, while ratiometric FRET-based designs trade added architectural complexity for substantially improved reliability in complex, real-world matrices. Third, selectivity is best achieved through deliberate, structurally informed recognition-element design, whether *via* aptamer or DNAzyme conjugation, molecular imprinting, or heteroatom doping, rather than relying on the intrinsic selectivity of the QD material alone.

Advancing these technologies from laboratory demonstration toward scalable, sustainable, and commercially viable products requires several practical priorities. Cadmium-free and green-synthesised QD alternatives must close the remaining quantum yield gap relative to cadmium-based systems while demonstrating batch-to-batch reproducibility at production scale, building on the scalability advantages of hydrothermal and microwave-assisted synthesis routes discussed in this review. Comprehensive life-cycle toxicity and disposal assessments, rather than in-use performance data alone, are essential for regulatory approval, particularly given the regulatory tension inherent in using cadmium-based QDs to detect cadmium contamination itself. Standardized interference-testing, aging, and validation protocols, adopted consistently across the research community, are a prerequisite for meaningful cross-study comparison and for building the evidentiary basis regulatory bodies require. Finally, progression beyond proof-of-concept and real-sample validation toward genuine, field-tested deployment, as distinguished earlier in this review, remains the most significant gap between current research output and practical environmental monitoring capability. Realising this potential will require sustained collaboration between nanomaterials researchers, manufacturing partners, and regulatory bodies, guided by the design principles and practical priorities outlined above, to translate laboratory-demonstrated capability into deployed, standardized environmental monitoring tools.

## Conflicts of interest

The authors affirm that they do not possess any identifiable conflicting financial interests or personal ties that might have potentially influenced the findings provided in this paper.

## Abbreviations

AA: Ascorbic acid
AAS: Atomic absorption spectroscopy
AFM: Atomic force microscopy
AFS: Atomic fluorescence spectroscopy
AgInS_2_ (AIS): Silver indium sulfide
AIF: Apoptosis-inducing factor
δ-ALAD: δ-Aminolevulinic acid dehydratase
APDC: Ammonium pyrrolidine dithiocarbamate
APTES: (3-Aminopropyl)triethoxysilane
ASSURED: Affordable, sensitive, specific, user-friendly, rapid/robust, equipment-free, deliverable
ASTM: American Society for Testing and Materials (ASTM International)
ASV: Anodic stripping voltammetry
4AT: 4-Amino TEMPO
AuNP: Gold nanoparticle
AuNRs: Gold nanorods
B-MOF: Blue-emitting metal–organic framework
Bcl-2: B-cell lymphoma 2 (protein family)
BER: Base-excision repair
BNCDs: Boron/nitrogen-doped carbon dots
BSA: Bovine serum albumin
CAT: Catalase
CD(s)/CQD(s): Carbon dot(s)/carbon quantum dot(s)
cDNA: Complementary DNA
CHEF: Chelation-enhanced fluorescence
CMC: Carboxymethyl chitosan
CNNPs: Graphitic carbon nitride nanoparticles
CNT(s): Carbon nanotube(s)
Cr(vi/v/iv/iii): Chromium oxidation states
CSF: Cerebrospinal fluid
CV-AAS: Cold vapor atomic absorption spectroscopy
CV-AFS: Cold vapor atomic fluorescence spectroscopy
CVD: Chemical vapor deposition
Cy5: Cyanine5 (fluorophore)
DDTC: Diethyldithiocarbamate
DFT: Density functional theory
DLS: Dynamic light scattering
DMA: Dimethylarsinic acid
DMDTA: Dimethyldithioarsenate
DMMTA: Dimethylmonothioarsenate
DNA: Deoxyribonucleic acid
DNAzyme: DNA enzyme
DPA-CuNCs: d-Penicillamine-stabilized copper nanoclusters
DPV: Differential pulse voltammetry
DTPA: Diethylenetriaminepentaacetic acid
DTPAN: Dithienopyrrole (ligand)
ECL: Electrochemiluminescence
EDC/NHS: 1-Ethyl-3-(3-dimethylaminopropyl)carbodiimide/*N*-hydroxysuccinimide
EDTA: Ethylenediaminetetraacetic acid
EPA: (U.S.) Environmental Protection Agency
EPR: Electron paramagnetic resonance
EXAFS: Extended X-ray absorption fine structure
FDA: Food and Drug Administration
FL: Fluorescence
FL/CL: Fluorescence/chemiluminescence
FLIM: Fluorescence lifetime imaging microscopy
FNA: Framework nucleic acid
FRET: Förster (fluorescence) resonance energy transfer
FTIR: Fourier-transform infrared (spectroscopy)
FWHM: Full width at half maximum
GABA: Gamma-aminobutyric acid
GFAAS: Graphite furnace atomic absorption spectroscopy
GO: Graphene oxide
GPx: Glutathione peroxidase
GQD(s): Graphene quantum dot(s)
GR: Glutathione reductase
GSB: Ground-state bleach
GSH: Glutathione
HEPES: 4-(2-Hydroxyethyl)-1-piperazineethanesulfonic acid (buffer)
HGAAS: Hydride generation atomic absorption spectroscopy
HOMO–LUMO: Highest occupied molecular orbital–lowest unoccupied molecular orbital
HPMA/GPMA: *N*-(2-Hydroxypropyl) methacrylamide/3-guanidinopropyl methacrylamide
HSA: Human serum albumin
HSAB: Hard–soft acid–base (theory)
IARC: International Agency for Research on Cancer
ICP-MS: Inductively coupled plasma mass spectrometry
ICP-OES: Inductively coupled plasma optical emission spectrometry
iCVD: Initiated chemical vapor deposition
IFE: Inner filter effect
IoT: Internet of things
ISO: International Organization for Standardization
IUPAC: International Union of Pure and Applied Chemistry
*K*_d_
: Dissociation constant
*K*_sel_
: Selectivity coefficient
*K*_sp_
: Solubility product constant
*K*_SV_
: Stern–Volmer constant
LOC: Lab-on-a-chip
LOD(s): Limit(s) of detection
log *K*: Logarithmic stability constant
LOQ: Limit of quantification
LSPR: Localized surface plasmon resonance
MCR-ALS: Multivariate curve resolution-alternating least squares
MES: 2-(*N*-Morpholino)ethanesulfonic acid (buffer)
MMA: Monomethylarsonic acid
MOF: Metal–organic framework
3-MPA: 3-Mercaptopropionic acid
N,B-CQDs: Nitrogen and boron co-doped carbon quantum dots
N,S-CQDs/NSCDs: Nitrogen and sulfur co-doped carbon (quantum) dots
N-CQDs: Nitrogen-doped carbon quantum dots
NAC: *N*-Acetyl cysteine
NER: Nucleotide-excision repair
NIR/NIR-II: Near-infrared/second near-infrared window
NIST: National Institute of Standards and Technology
NMR: Nuclear magnetic resonance
NP(s): Nanoparticle(s)
PBS: Phosphate-buffered saline
PCA: Principal component analysis
PEC: Photoelectrochemical
PEG: Polyethylene glycol
PEI: Polyethyleneimine
PET: Photoinduced electron transfer
PL: Photoluminescence
PLQY: Photoluminescence quantum yield
PLS: Partial least squares
PQD: Perovskite quantum dot
PVA: Polyvinyl alcohol
QC: Quality control
QD(s): Quantum dot(s)
QY: Quantum yield
RAFT: Reversible addition–fragmentation chain transfer
REACH: Registration, evaluation, authorisation and restriction of chemicals
RGB: Red-green-blue (color model)
RNA: Ribonucleic acid
RoHS: Restriction of hazardous substances
RSD: Relative standard deviation
SELEX: Systematic evolution of ligands by exponential enrichment
Si-QDs: Silicon quantum dots
SILAR: Successive ionic layer adsorption and reaction
SiO_2_-QDs@CND: Silicon dioxide QDs on carbon nanodiscs
SOD: Superoxide dismutase
SPE: Solid-phase extraction
SQDs: Sulfur quantum dots
SRM: Standard reference material
SSQDs: Solid-solution quantum dots
T-QDs: Tangerine-emitting quantum dots
TAS: (Femtosecond) transient absorption spectroscopy
Tb-MOF: Terbium metal–organic framework
TCSPC: Time-correlated single-photon counting
TDN: DNA tetrahedron
TEM: Transmission electron microscopy
TEMPO: (2,2,6,6-Tetramethylpiperidin-1-yl)oxyl
TGA: Thioglycolic acid
Tris–HCl/Tris–HAc: Tris(hydroxymethyl)aminomethane–hydrochloride/–acetate (buffers)
TRPL: Time-resolved photoluminescence
TSCA: Toxic Substances Control Act (US)
US EPA: United States Environmental Protection Agency
UV: Ultraviolet
WHO: World Health Organization
XANES: X-ray absorption near-edge structure
XAS: X-ray absorption spectroscopy
XPS: X-ray photoelectron spectroscopy
XRD: X-ray diffraction
XRF: X-ray fluorescence

## Data Availability

This review article does not report any original research data. All data discussed are drawn from previously published studies, which are cited accordingly throughout the manuscript. No new datasets were generated or analysed during the preparation of this work.
